# Profile Photograph Classification Performance of Deep Learning Algorithms Trained Using Cephalometric Measurements: A Preliminary Study

**DOI:** 10.3390/diagnostics14171916

**Published:** 2024-08-30

**Authors:** Duygu Nur Cesur Kocakaya, Mehmet Birol Özel, Sultan Büşra Ay Kartbak, Muhammet Çakmak, Enver Alper Sinanoğlu

**Affiliations:** 1 Department of Orthodontics, Faculty of Dentistry, Kocaeli University, Kocaeli 41190, Türkiye; duyguucesur@gmail.com (D.N.C.K.); sultanbusraay@gmail.com (S.B.A.K.); 2 Department of Computer Engineering, Faculty of Engineering and Architecture, Sinop University, Sinop 57000, Türkiye; mcakmak@sinop.edu.tr; 3 Department of Oral and Maxillofacial Radiology, Faculty of Dentistry, Kocaeli University, Kocaeli 41190, Türkiye; alpersinanoglu@yahoo.com

**Keywords:** deep learning, artificial intelligence, profile photograph, cephalometry, orthodontics

## Abstract

Extraoral profile photographs are crucial for orthodontic diagnosis, documentation, and treatment planning. The purpose of this study was to evaluate classifications made on extraoral patient photographs by deep learning algorithms trained using grouped patient pictures based on cephalometric measurements. Cephalometric radiographs and profile photographs of 990 patients from the archives of Kocaeli University Faculty of Dentistry Department of Orthodontics were used for the study. FH-NA, FH-NPog, FMA and N-A-Pog measurements on patient cephalometric radiographs were carried out utilizing Webceph. 3 groups for every parameter were formed according to cephalometric values. Deep learning algorithms were trained using extraoral photographs of the patients which were grouped according to respective cephalometric measurements. 14 deep learning models were trained and tested for accuracy of prediction in classifying patient images. Accuracy rates of up to 96.67% for FH-NA groups, 97.33% for FH-NPog groups, 97.67% for FMA groups and 97.00% for N-A-Pog groups were obtained. This is a pioneering study where an attempt was made to classify clinical photographs using artificial intelligence architectures that were trained according to actual cephalometric values, thus eliminating or reducing the need for cephalometric X-rays in future applications for orthodontic diagnosis.

## 1. Introduction

Facial photographs reveal the aesthetic characteristics of the facial shape and its relationship to the teeth. They can be used in conjunction with a combination of radiographic measurements [1,2]. Facial photography allows for evaluation of the patient’s facial aesthetics, visualizing their current condition, creating an appropriate treatment plan and monitoring the progress of the treatment [3].

Artificial intelligence [AI] technology has made a significant contribution on orthodontic treatment planning [4]. AI-based software systems, which play an important and transformative role in orthodontics, are considered the future of dental applications. AI is utilized in every aspect of orthodontics, from patient communication to diagnosis and treatment processes [5]. Recent orthodontic studies investigating AI have focused on two or three-dimensional digital radiographs and tools to provide standard patient care, achieving treatment goals and clinical decision-making goals in the most successful way [6,7,8].

AI is described as the science and engineering of making intelligent machines, especially intelligent computer programs, by McCarthy [9]. Deep learning (DL) is a part of machine learning designed to mimic the recognition system of the human brain while leveraging the computing power of graphics processing units [10,11]. As an important subset of DL techniques, convolutional neural network systems (CNNs) have gained popularity in the field of graphical image analysis. It has been reported that CNNs are widely used among deep learning algorithms and are well suited for image processing, including with medical images [12,13,14]. Accurate diagnosis based on cephalometric measurement is prerequisite for a successful treatment. Lateral cephalometric radiography is widely used as a standard tool in orthodontic evaluation and in dentofacial diagnosis [15,16].

As a medical image, patient profile photographs have not been investigated for their potential to reflect cephalometric data. To the best of our knowledge, this is the first study in which clinical profile photographs were evaluated using DL models in orthodontic planning processes. Therefore, the aim of our study was to compare classifications made solely from photographs by different DL models trained with categorized patient photographs based on cephalometric measurements with actual cephalometric classifications.

The null hypothesis of this study was that extraoral photograph classifications are unrelated to cephalometric measurement classifications.

## 2. Materials and Methods

This study was approved by the Ethics Committee of Kocaeli University (GOKAEK-2023/06.21). The pretreatment cephalometric radiographs and profile photographs of patients were randomly retrieved from the database of Kocaeli University Faculty of Dentistry, Department of Orthodontics, during the period between 2014 and 2018. 

### 2.1. Study Group Selection

The study group consisted of 990 individuals who had not received any orthodontic treatment before and had both cephalometric radiographs and profile photographs taken at the same session. Individuals who had previously received orthodontic treatment, had cleft lip and palate or any other syndromic craniofacial anomaly were excluded from the study. Although missing teeth were not quantified, severe edentulism that may have affected craniofacial morphology was eliminated by the exclusion of syndromic anomalies. Lateral cephalometric radiographs with artefacts or photographs with poor image quality for the assessment were also excluded.

The lateral cephalometric radiographs were taken with a Morita Veraviewepocs 2D (J. Morita MFG. Corp, Kyoto, Japan) device under standard conditions and with a magnification difference of 1.1 mm, as determined by the manufacturer.

The profile photographs were taken with a Nikon D700 digital camera and Nikon AF-S VR Micro Nikkor 105 mm f/2.8 G IF ED lens. In order to fully visualize the face, the hair of the patient was gathered behind the ear, and photographs were taken from the right side to ensure that the patient’s profile view was compatible with the cephalometric radiography.

### 2.2. Cephalometric Measurements

**Figure 1:**
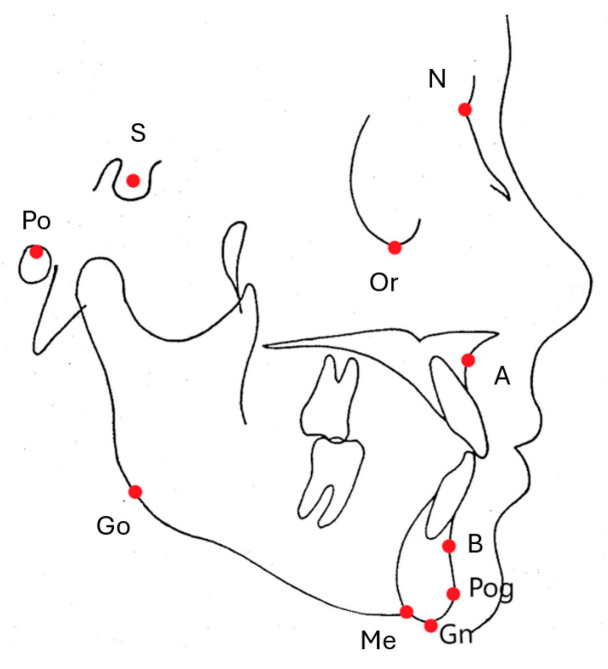
Cephalometric reference points.

A total of 4 angular measurements were performed on cephalometric radiographs after 10 cephalometric landmarks (point A, point B, Sella, Nasion, Orbita, Porion, Pogonion, Gonion, Gnathion, Menthon) were determined (Figure 1).

FH-NA, FH-NPog, FMAand N-A-Pog were measured to evaluate the position of the maxilla, the position of the mandible, the vertical development of the face and profile convexity respectively. Frankfurt Horizontal reference plane was selected to evaluate the relationship between the facial features and the cephalometric measurements.

**Figure 2:**
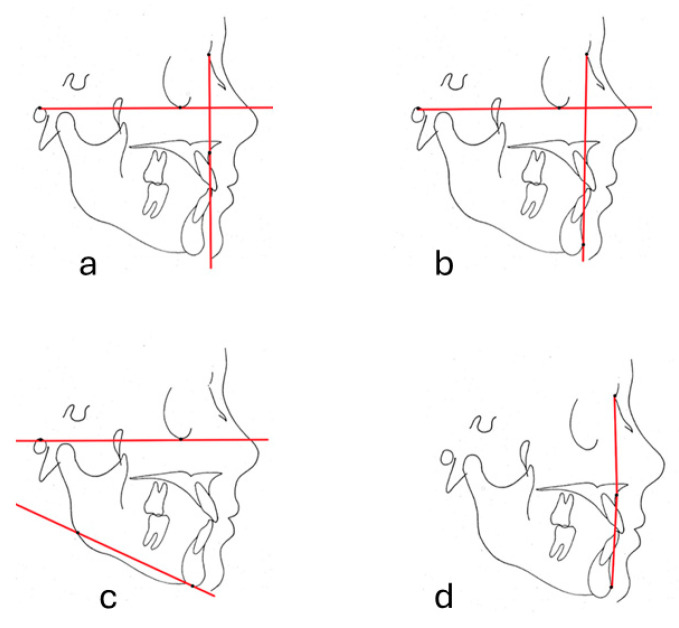
(**a**) FH-NA, (**b**) FH-NPog, (**c**) FMA, (**d**) N-A-Pog.

The Webceph digital cephalometric measurement program (AssembleCircle Corp., Pangyoyeok-ro, Bundang-gu, Seongnam-si, Gyeonggi-do, Republic of Korea) was used for performing below mentioned measurements:

FH-NA was the angle between the Frankfort horizontal plane passing through the Porion and Orbita points and the plane passing through the Nasion and A points, which was used to assess the position of the maxilla in the sagittal direction (Figure 2a). 

FH-NPOG was the angle between the Frankfort horizontal plane passing through the Porion and Orbita points and the plane passing through the Nasion and Pogonion points. This measurement was used to classify the sagittal position of the mandible (Figure 2b).

FMA was constructed as the angle between the Frankfort horizontal plane passing through the Porion and Orbita points and the plane passing through the Gonion and Menthon points. The vertical dimension of the face is classified according to this measurement (Figure 2c).

N-A-Pog was the angle between Nasion, point A and the Pogonion point. This angle was used to evaluate the profile convexity of the face (Figure 2d).

After completion of cephalometric measurements, 50 randomly selected lateral cephalometric radiographs were re-evaluated two weeks later by the same researcher to evaluate repeatability of the measurements. Intraobserver agreement was assessed by the intraclass correlation coefficient and were found to be between 0.85 and 0.99, presenting high reproducibility for assessment of the 4 angles. 

### 2.3. Profile Photograph Group Formation for DL

The study group was divided into 3 subgroups according to cephalometric measurement values. The cephalometric values for the subgroups were as follows: 

For FH-NA; first group (*n* = 330) was formed (FH-NA1) between measurement scores of 79.58 and 90.02. Second group (*n* = 330) was formed (FH-NA2) between measurement scores of 90.02 and 93.00. Third group (*n* = 330) was formed (FH-NA3) between measurement scores of 93.00 and 103.89.

For FH-NPOG; First group (*n* = 330) was formed (FH-NPOG1) between measurement scores of 72.49 and 84.32. Second group (*n* = 300) was formed (FH-NPOG2) between measurement scores of 84.32 and 87.32. Third group (*n* = 300) was formed (FH-NPOG3) between measurement scores of 87.32 and 99.52.

For FMA; first group (*n* = 330) was formed (FMA1) between measurement scores of 10.38 and 22.48. Second group (*n* = 330) was formed (FMA2) between measurement scores of 22.48 and 27.75. Third group (*n* = 330) was formed (FMA3) between measurement scores of 27.75 and 46.68.

N-A-Pog; first group (*n* = 330) was formed (N-A-POG1) between measurement scores of −27.98 and 3.31. Second group (*n* = 330) was formed (N-A-POG2) between measurement scores of 3.31 and 8.9. Third group (*n* = 330) was formed (N-A-POG3) between measurement scores of 8.9 and 26.69.

**Figure 3:**
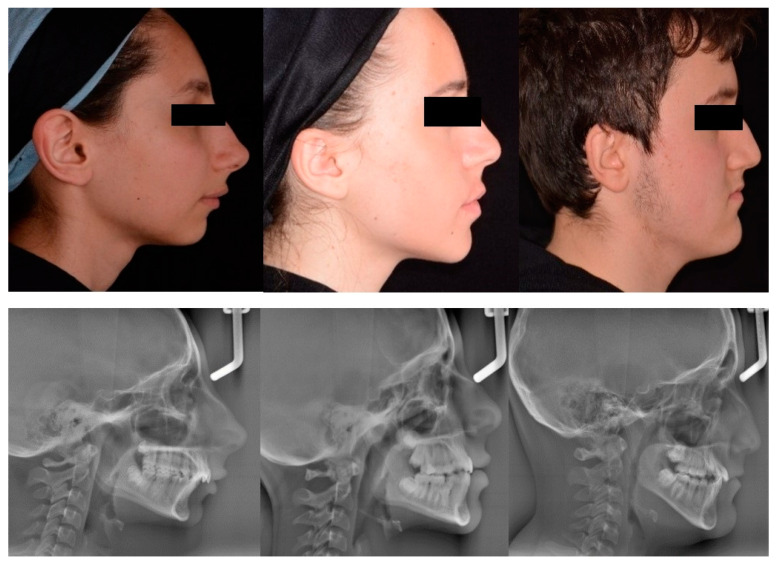
Sample photographs and cephalograms of 3 patients grouped according to cephalometric values.

After formation of subgroups all profile photographs of groups were sent for DL analysis (Figure 3).

### 2.4. Preparation of Deep Learning Models

Image classification procedures were implemented in the laboratory of Karabük University, Department of Electrical and Electronics Engineering. Data augmentation was applied to the grouped profile photographs on all deep learning models to obtain higher accuracy and lower loss values than the deep learning models used for the study. 

The 14 DL models that were employed were as follows:

- MobileNet V2,
- Inception V3,
- DenseNet 121,
- DenseNet 169,
- DenseNet 201,
- EfficientNet B0,
- Xception,
- VGG16,
- VGG19,
- NasNetMobile,
- ResNet101,
- ResNet 152,
- ResNet 50,
- EfficientNet V2

The average number of photographs in each class, which was 330 in the above mentioned original data sets, was increased to 1000 photographs for each group (FH-NA1, FH-NA2 and FH-NA3 for FH-NA; FH-NPOG1, FH-NPOG2 and FH-NPOG3 for FH-NPOG; FMA1, FMA2 and FMA3 for FMA; N-A-POG1, N-A-POG2 and N-A-POG3 for N-A-POG).

Following the data enrichment process, in the first step, training, validation and test data sets were created for all deep learning models. These data sets consist of labeled samples. In all deep learning models, the training data set constitutes 80% of the entire data set, the validation data set constitutes 10%, and the test data set constitutes the remaining 10%. Therefore, the training dataset contains 800 images, and the validation and test datasets contain 100 images.

After the data sets were arranged, a series of filters (kernels) were applied to the input data for the first convolution layer for deep learning. Each filter acts on the input data with a convolution process and multiplies and sums the pixel values in the region to be filtered. This sum forms one pixel of the feature map.

In the pooling layer, the feature maps are reduced, and the features are made more invariant to scale and location changes. After the pooling layers, the feature maps were flattened and transmitted to the dense layers. After the last dense layer, classification was performed in the output layer. For classification, a softmax activation function was used. Experimental study: DL models were trained using a GPU-supported system in the Google cloud environment.

Training was performed on a Tesla T4 GPU and an Intel Xeon CPU with 16 GB RAM running at 2.20 GHz. Python 3 was used to write all programs for transfer learning design, and the Keras 2.3.1 training framework was used to train the models.

After completing learning and training and validation procedures, all photographic images belonging to cephalometric measurement groups were tested (*n* = 100) via 14 DL algorithms (MobileNet V2, Inception V3, DenseNet 121, Densenet 169, Densenet 201, Efficientnet B0, Xception, Vgg16, Vgg19, Nasnetmobile, Resnet101, Resnet152, Resnet50 and Efficientnet V2) for their accuracy in predicting the cephalometric classification. 

Accuracy value was calculated by the percentage of correctly classified subjects using the confusion matrix.

(TP + TN)/(TP + TN + FP + FN)

TP (True Positive): Number of profile photos correctly classified as belonging to the group by the DL model. TN (True Negative): Number of profile photos correctly classified as not belonging to the evaluated group by the DL model. 

FP (False Positive): Number of profile photos incorrectly classified as belonging to the group by the DL model.

FN (False Negative): Number of profile photos incorrectly classified as not belonging to the evaluated group by the DL model.

Precision, recall and F1 score values were also calculated for every successful sequence with a total of 40 instances.

Precision (positive predictive value) is the proportion of true positive results among all the positive results identified by the DL model, and it was calculated by the formula: Precision = TP/(TP + FP).

Recall (sensitivity or true positive rate) refers to the proportion of positive results correctly identified by the DL model out of all the actual positive cases and it was calculated by the formula: Recall = TP/(TP + FN).

F1 score is a score between 0–1, where the value 1 indicates a perfect balance between precision and recall. It provides an overall performance metric of the model, considering both precision and recall. The F1 score is the harmonic mean of precision and recall, which is calculated by the formula: F1 score =2 × (Recall × Precision)/(Recall + Precision). 

## 3. Results

DL algorithms exhibited varying degrees of accuracy in the prediction of the 4 cephalometric traits measured. These results depicted that the null hypothesis was rejected in 40 of 56 prediction sequences where DL algorithms demonstrated acceptable accuracy of prediction. The null hypothesis was accepted in 16 prediction sequences where the prediction accuracy was found to be equal to or lower than 36.37%. Profile photograph classification performances of CNN models using state-of-the-art deep learning models trained according to actual cephalometric measurements were evaluated by accuracy, precision, sensitivity and F1 Scores. The accuracy of the prediction results is shown in Table 1.

FH-NA angle value; from a set of 14 different DL models, Efficient B0 presented a relatively high accuracy rate of 96.67%, with correct results for 290 images out of 300. VGG16 presented the lowest accuracy rate, of 30.67%.

FH-NPOG angle value; from a set of 14 different DL models, DenseNet201 presented the highest accuracy rate of 97.33%, with correct results for 292 images out of 300. VGG19 and Inception V3 presented the lowest accuracy rates, of 31.33%.

FMA angle value; from a set of 14 different DL models, DenseNet 201 presented the highest accuracy rate of 97.67%, with correct results for 293 images out of 300. VGG19 presented the lowest accuracy rate, of 31.33%.

N-A-POG angle value; from a set of 14 different DL models, Efficient V2 presented the highest accuracy rate of 97.00% with correct results for 291 images out of 300. VGG19 presented the lowest accuracy rate of 34.67%.

Precision, recall and F1 score values and confusion matrices of the 40 instances have been provided as Supplementary Data owing to the high volume of information.

## 4. Discussion

The use of medical images with DL training is becoming a point of interest in recent studies. In evaluation of intraoral medical images, Tanriver et al. evaluated six DL algorithms and reported that DL-based approaches could be applied for automatic detection and classification of oral lesions [17]. Warin et al. also investigated DL algorithms to develop a classification and detection model of oral cancer photographs and trained three DL algorithms. They stated that DL-based algorithms offer an acceptable potential for classification and detection of lesions in oral images [18,19]. In our study, cephalometric radiographs and profile photographs of 900 patients were evaluated with 14 DL models which provided a more comprehensive perspective in assessing the potential of available DL algorithms. Additionally, considering the high accuracy rates for each cephalometric analyses, our results also suggest that DL training may offer promising results for orthodontic purposes.

In the literature, it is seen that the DenseNet201 model is used more in image classification studies than other models [20,21,22]. For our study, the highest accuracy values were seen in the DenseNet201 algorithm. Benyahia S. et al. [20], trained DL models to classify medical images containing skin lesions and extract lesion features. DenseNet201 was the DL model that gave the highest accuracy in terms of both medical image classification and feature extraction. Thalakottor et al. [21] used three convolutional neural network (CNN) models for medical image classification in their study. (VGG19, DenseNet201, ResNet50V2). Of these, the DenseNet201 model performed better than other models and achieved the highest accuracy. Mingzhu Meng et al. [22] examined the degree of overlap between the histopathological method for distinguishing benign and malignant breast lesions in their study. They stated that they preferred the DenseNet 201 DL model because of its ability to reuse features, its ability to reduce exploding features, and because it is associated with fewer gradient disappearance problems.

In our study, care was taken to select angles that could correlate with cephalometric radiographs and patients’ photographs. To determine the position of the maxilla and mandible, angles that we could associate with the FH reference plane were used instead of SNA and SNB. In studies comparing the Sella–Nasion reference plane and the Frankfurt Horizontal reference plane for cephalometric orientation, it is stated that clinically visualizing the Frankfurt Horizontal plane provides the opportunity to evaluate its relationship with the face, maxilla and mandible, and for reliability [23].

Considering the necessity of dentofacial photographic records for epidemiological purposes, in cases where initial examinations and irradiation are contraindicated or should be strictly avoided, photographs may provide cephalometric information that may be sufficient for such purposes [24,25,26]. DL trained models that have analyzed photographs may provide cephalometric information which may be adequate for such purposes.

Our limitation is that our study was conducted on a relatively narrow population, especially for algorithms that require large databases such as deep learning. It can also be speculated that, in the pre-processing of the photographs by the software, the differences in eyes, ears and hair of individuals in their profile photos may have presented as excessive data for the DL algorithm. This may have been misleading for accurate classification decisions. Additionally, this may also be the case for parameters such as patient age, gender, hair color, and other facial features that lack a direct relationship with investigated features by DL model.

This dataset, consisting of profile photographs, was obtained only from patients of our faculty’s orthodontics department. In order to develop the model, this problem was solved by using the data augmentation method in the training data set. The key to deep learning is a large data set to develop the performance model. The data set could be expanded by collecting cases from different centers or collecting more image data from a similar program via telemedicine.

Apart from the angular measurements used in our study, there are hundreds of parameters that can be measured in cephalometric X-rays and other DL models that can be trained. With further studies, DL models that can provide higher accuracy values can be developed.

By cropping the cephalometric X-rays and photographs we use, studies can be carried out in more specific regions for the extraction and classification of features, and DL models can be trained.

It is thought that in future applications, automatic measurements of facial and intraoral photographs without the need for cephalometric radiographs will be used in clinical orthodontics as a diagnostic step. 

## 5. Conclusions

The most successful deep learning models were DENSENET 201 and EFFICIENTNET V2.

The classification according to FH-NPog angle exhibited the lowest and N-A-Pog exhibited highest success rate in classification.

The results of this study were promising in predicting cephalometric classifications from profile photographs without using cephalometric X-rays.

## Figures and Tables

**Table 1:** DL algorithm’s prediction accuracy of cephalometric classifications based on profile photographs.

DL Model	FH-NA (%)	FH-NPog (%)	FMA (%)	N-A-Pog (%)
MobileNet V2	90.33	88.33	92.67	89.33
Inception V3	36.37	31.33	89.00	79.00
DenseNet 121	93.00	93.67	95.00	93.00
DenseNet 169	91.67	93.67	92.67	95.00
DenseNet 201	94.67	97.33 *	97.67 *	96.00
EfficientNet B0	96.67 *	96.33	93.33	96.33
XCeption	94.00	33.3	33.67	93.00
VGG16	30.67	32.33	37	35.33
VGG19	34.33	31.33	31.33	34.67
NasNetMobile	77.00	80.33	84.00	81.67
ResNet 101	83.00	34.67	34.33	64.00
ResNet 152	67.67	35.33	33.37	64.33
ResNet 50	84.33	84.67	88.67	81.67
EfficientNet V2	95.67	96.00	97.00	97.00 *

* DL models with the highest accuracy value, accuracy values in italics are not evaluated.

## Data Availability

The raw data supporting the conclusions of this article will be made available by the authors on request.

## Supplementary Materials

The following supporting information can be downloaded at: https://www.mdpi.com/article/10.3390/diagnostics14171916/s1.
