# Supplementary material for: Profile Photograph Classification Performance of Deep Learning Algorithms Trained Using Cephalometric Measurements: A Preliminary Study
Source: Diagnostics (Basel). 2024 Aug 30;14(17):1916. doi: 10.3390/diagnostics14171916 (PMC11394270; doi:10.3390/diagnostics14171916)
Supplement: Supplementary file 1 [file diagnostics-14-01916-s001.zip › FHNA.pdf]

MOBILENET V2

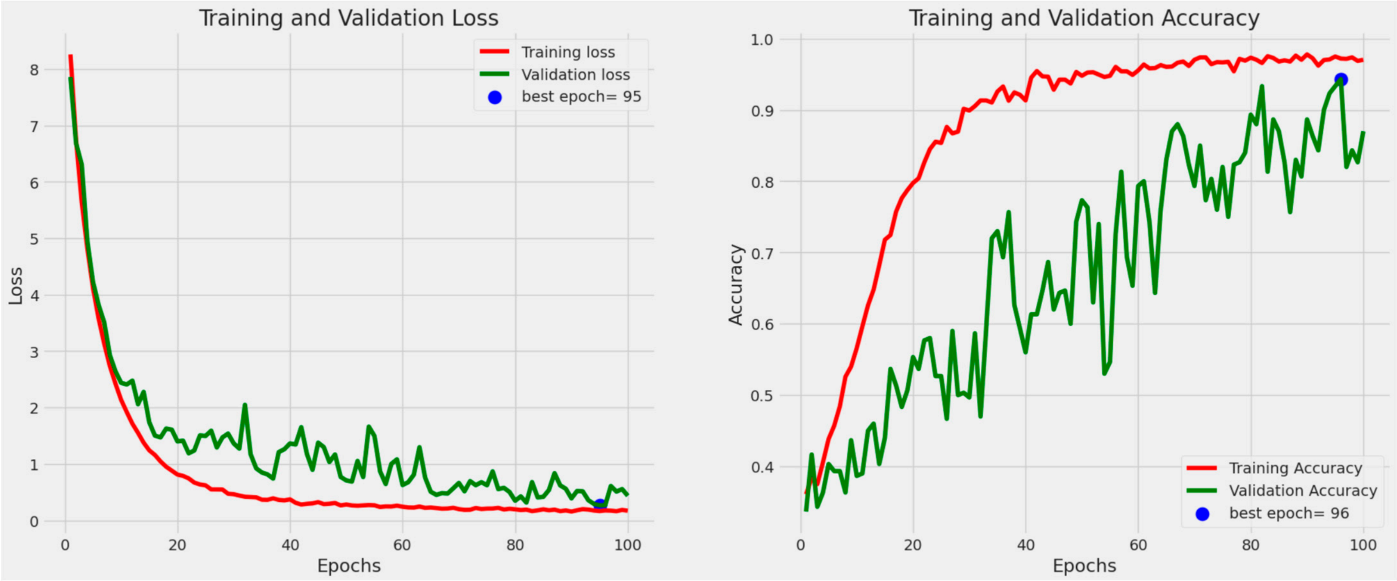

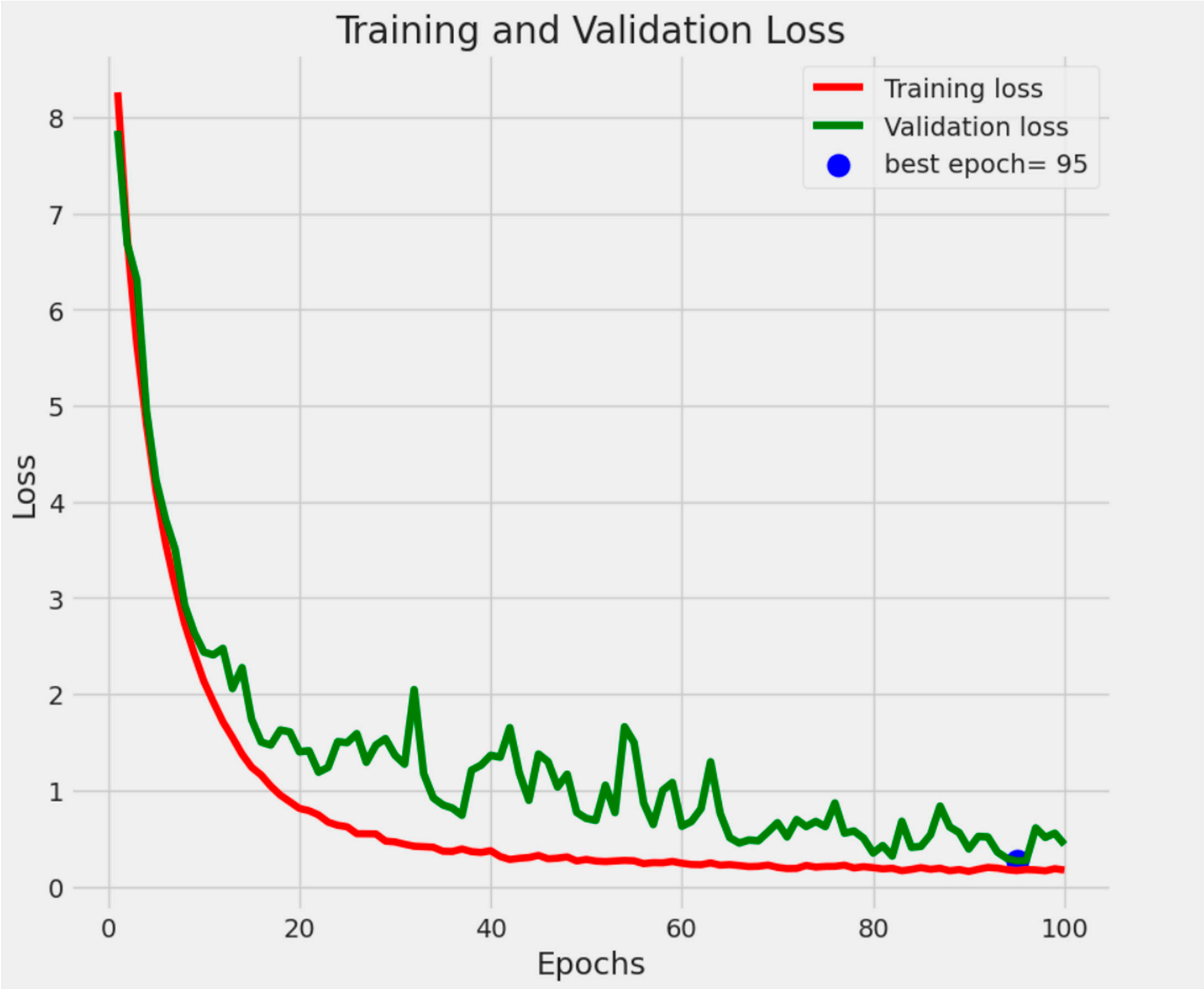

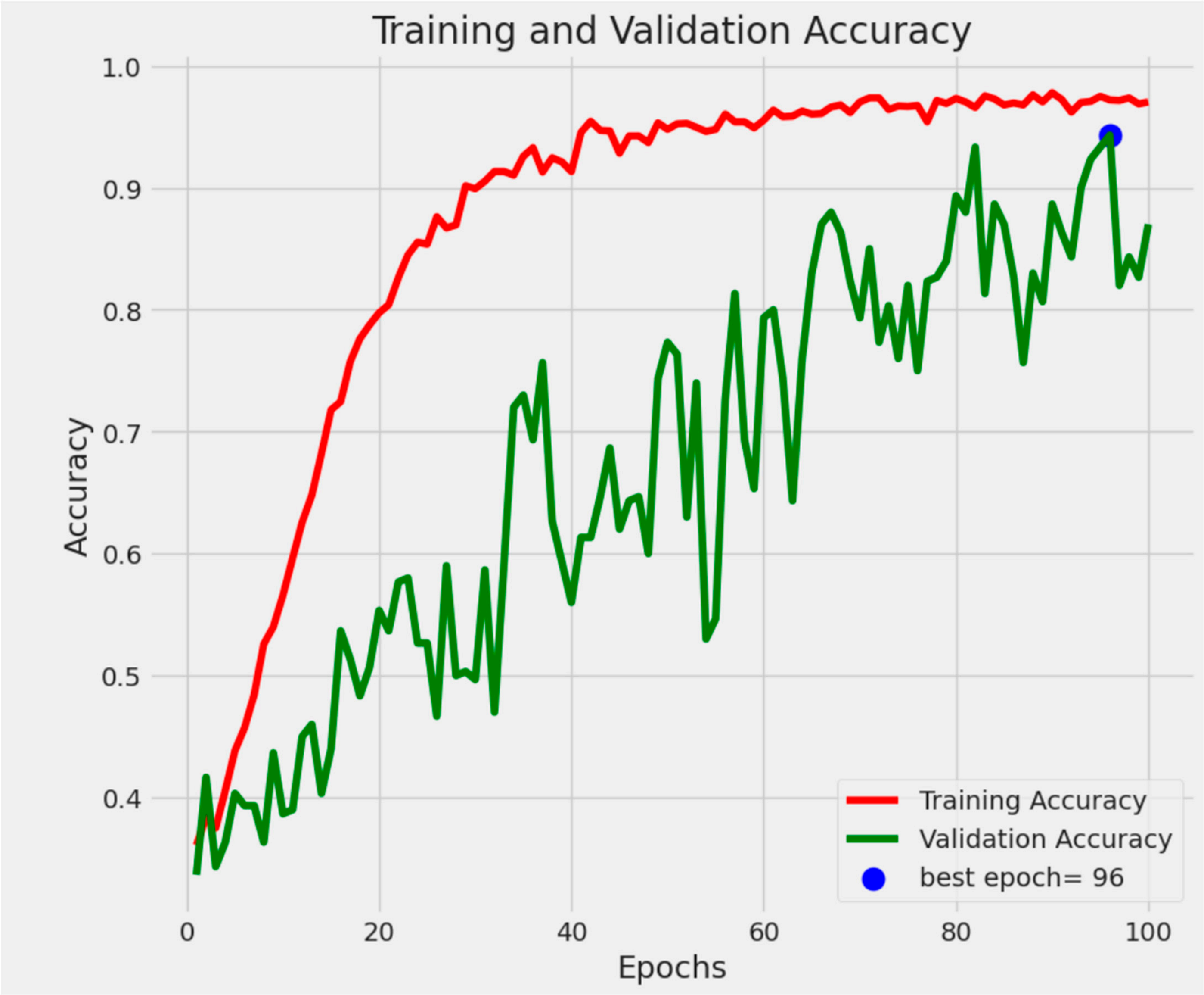

Training and Validation Accuracy
1.0
0.9
0.8
0.7
0.6
0.5
0.4
Accuracy
0
20
40
60
80
100
Epochs
Training Accuracy
Validation Accuracy
best epoch= 96

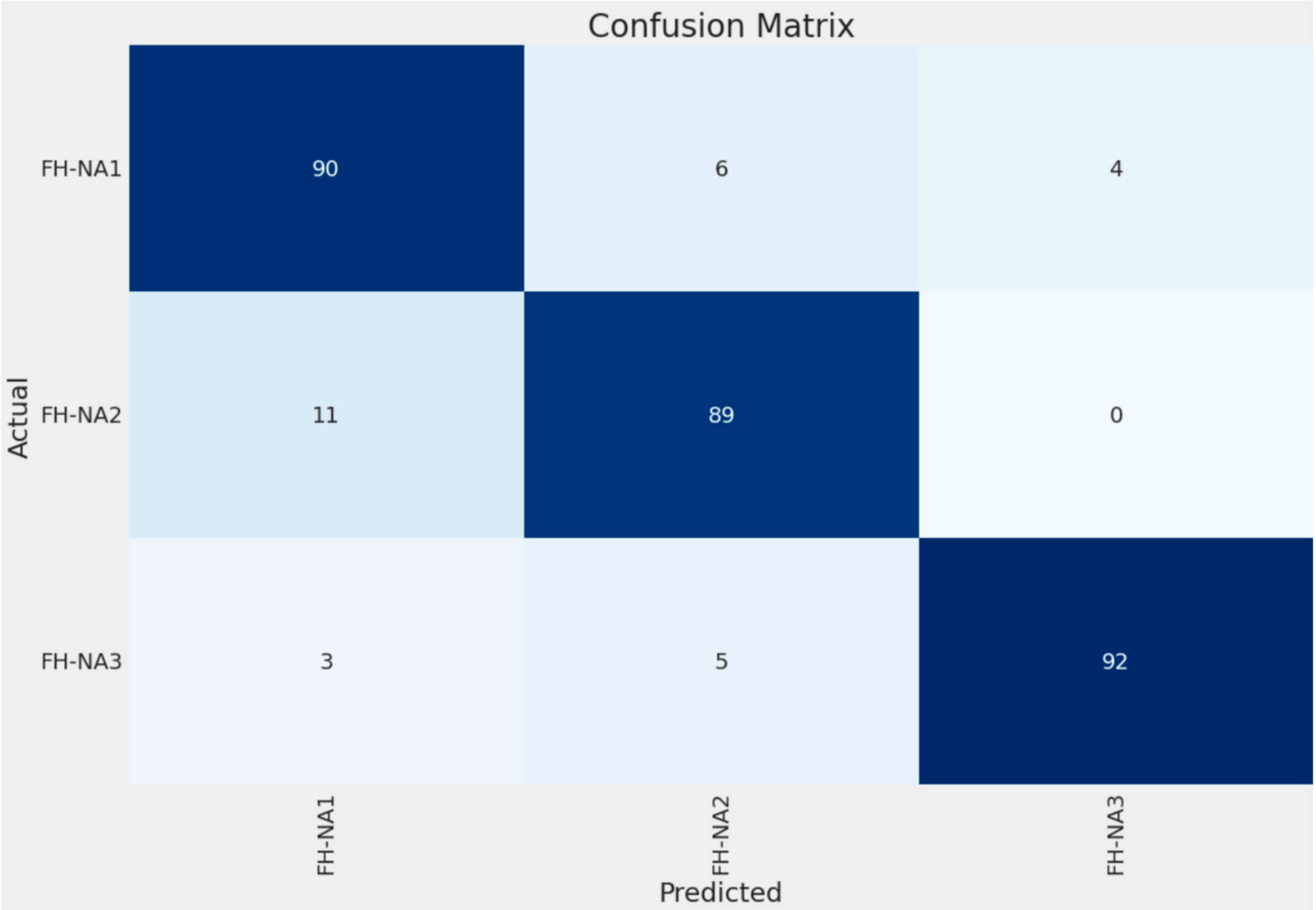

there were 29 errors in 300 tests for an accuracy of 90.33

Classification Report:
----

	precision	recall	f1-score	support
FH-NA1	0.8654	0.9000	0.8824	100
FH-NA2	0.8900	0.8900	0.8900	100
FH-NA3	0.9583	0.9200	0.9388	100
accuracy			0.9033	300
macro avg	0.9046	0.9033	0.9037	300
weighted avg	0.9046	0.9033	0.9037	300

INCEPTION V3: 36.37

DENSENET 121

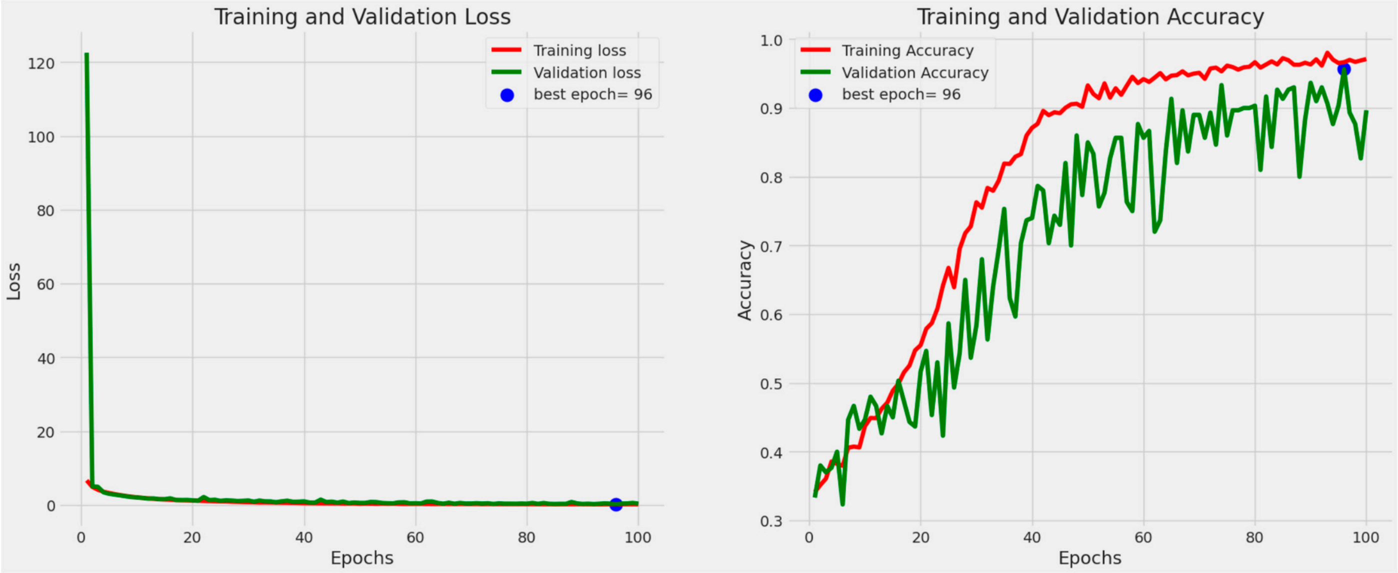

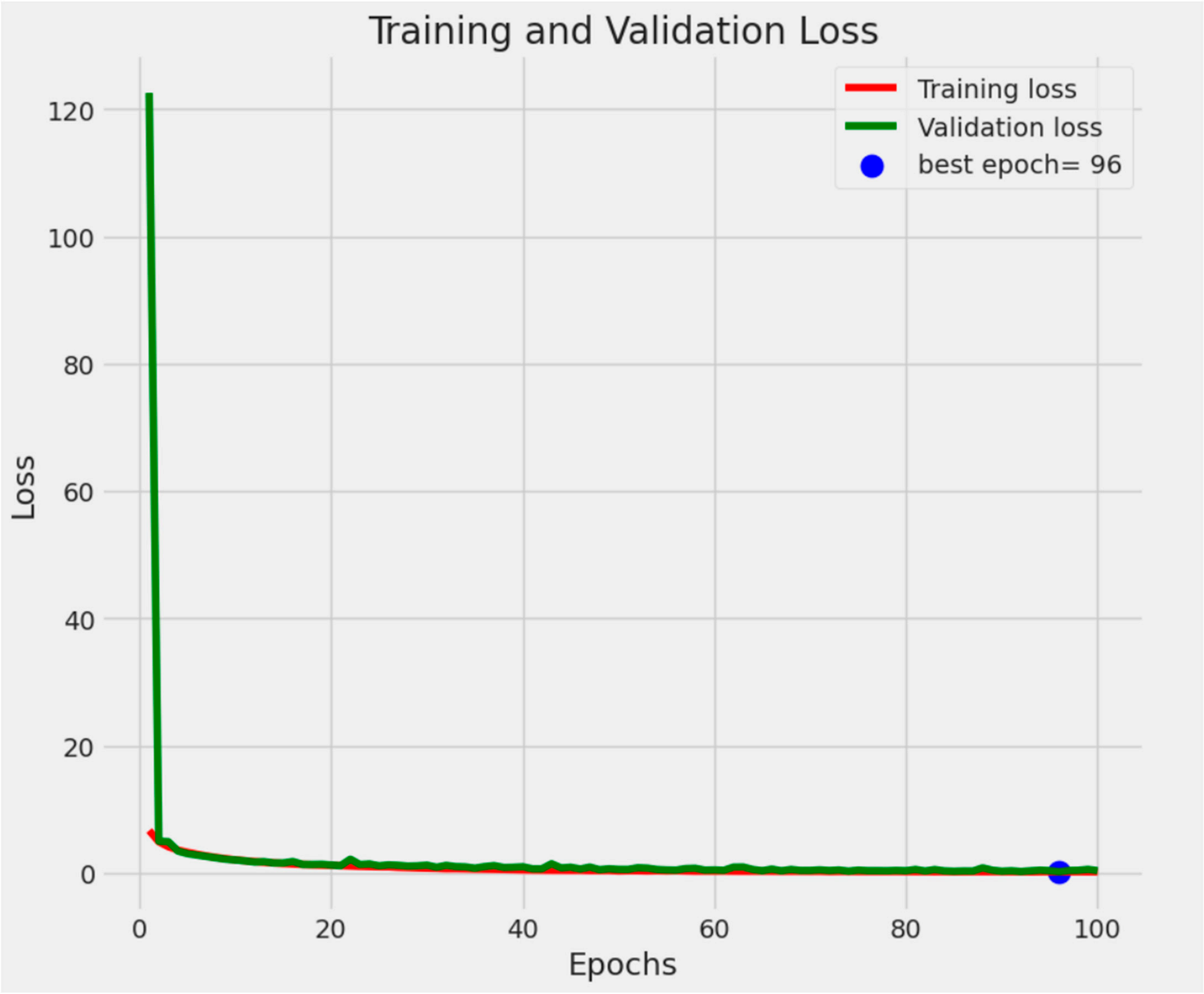

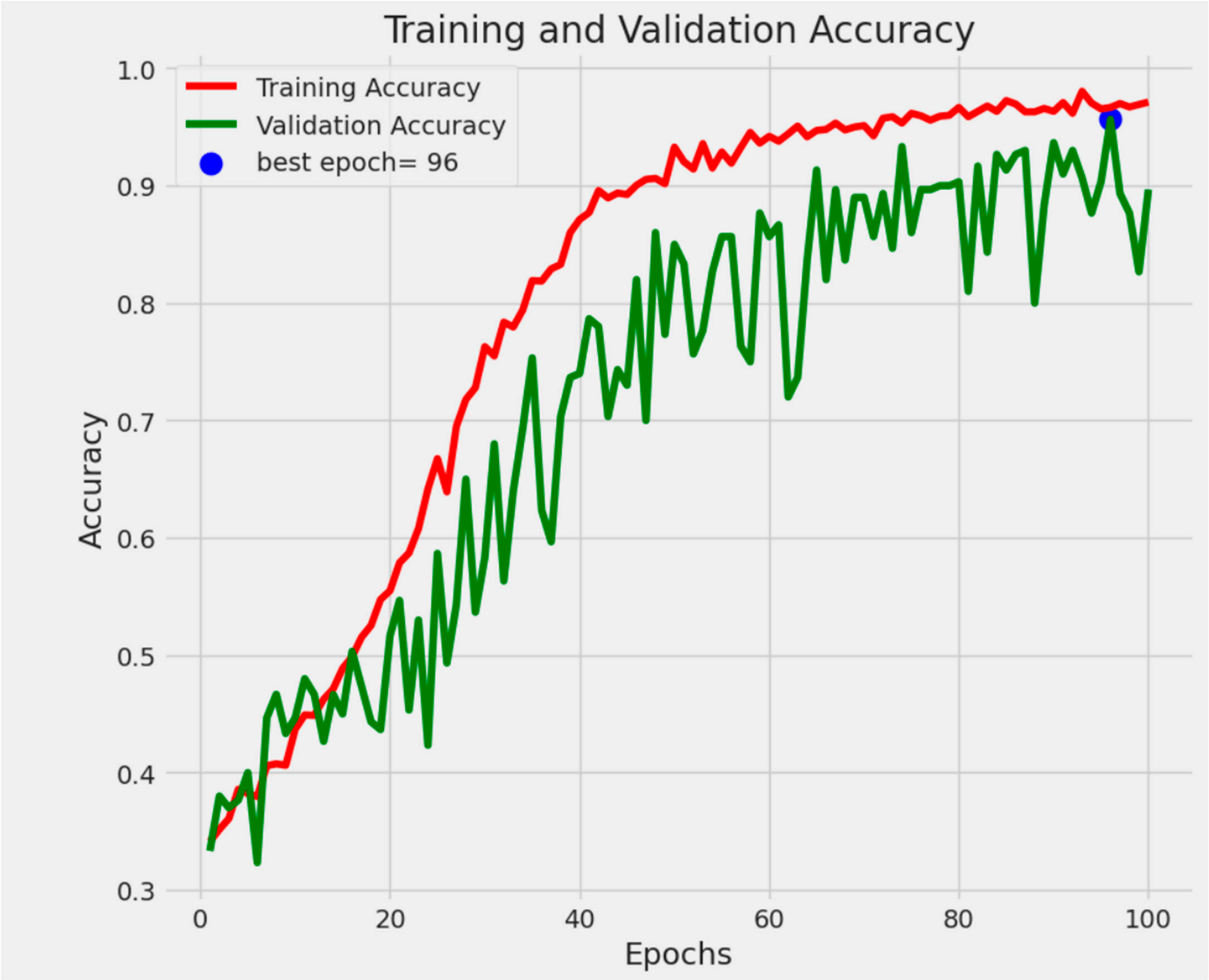

Training and Validation Accuracy
Training Accuracy
Validation Accuracy
best epoch= 96
Accuracy
Epochs
1.0
0.9
0.8
0.7
0.6
0.5
0.4
0.3
0
20
40
60
80
100

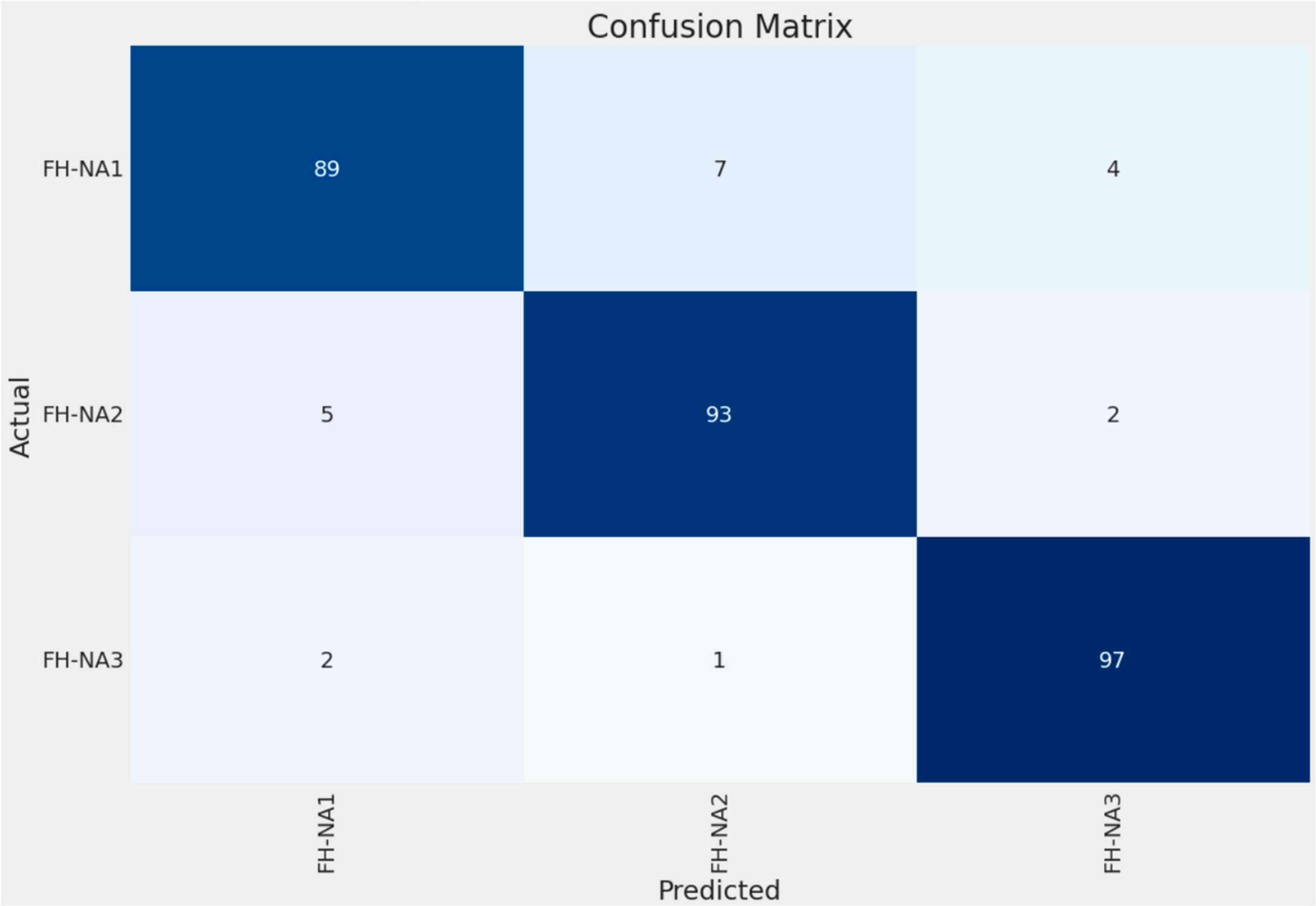


```
there were 21 errors in 300 tests for an accuracy of  93.00

Classification Report:
----------------------
              precision    recall  f1-score   support

      FH-NA1     0.9271    0.8900    0.9082       100
      FH-NA2     0.9208    0.9300    0.9254       100
      FH-NA3     0.9417    0.9700    0.9557       100

    accuracy                         0.9300       300
   macro avg     0.9299    0.9300    0.9297       300
weighted avg     0.9299    0.9300    0.9297       300
```

DENSENET 169

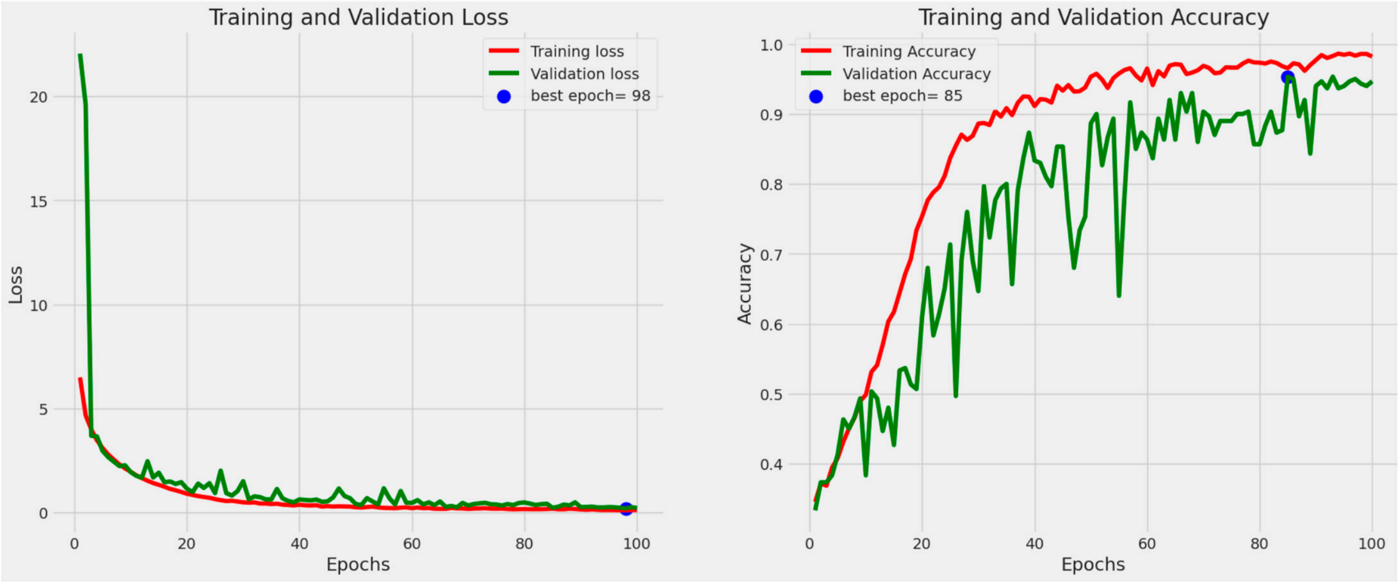

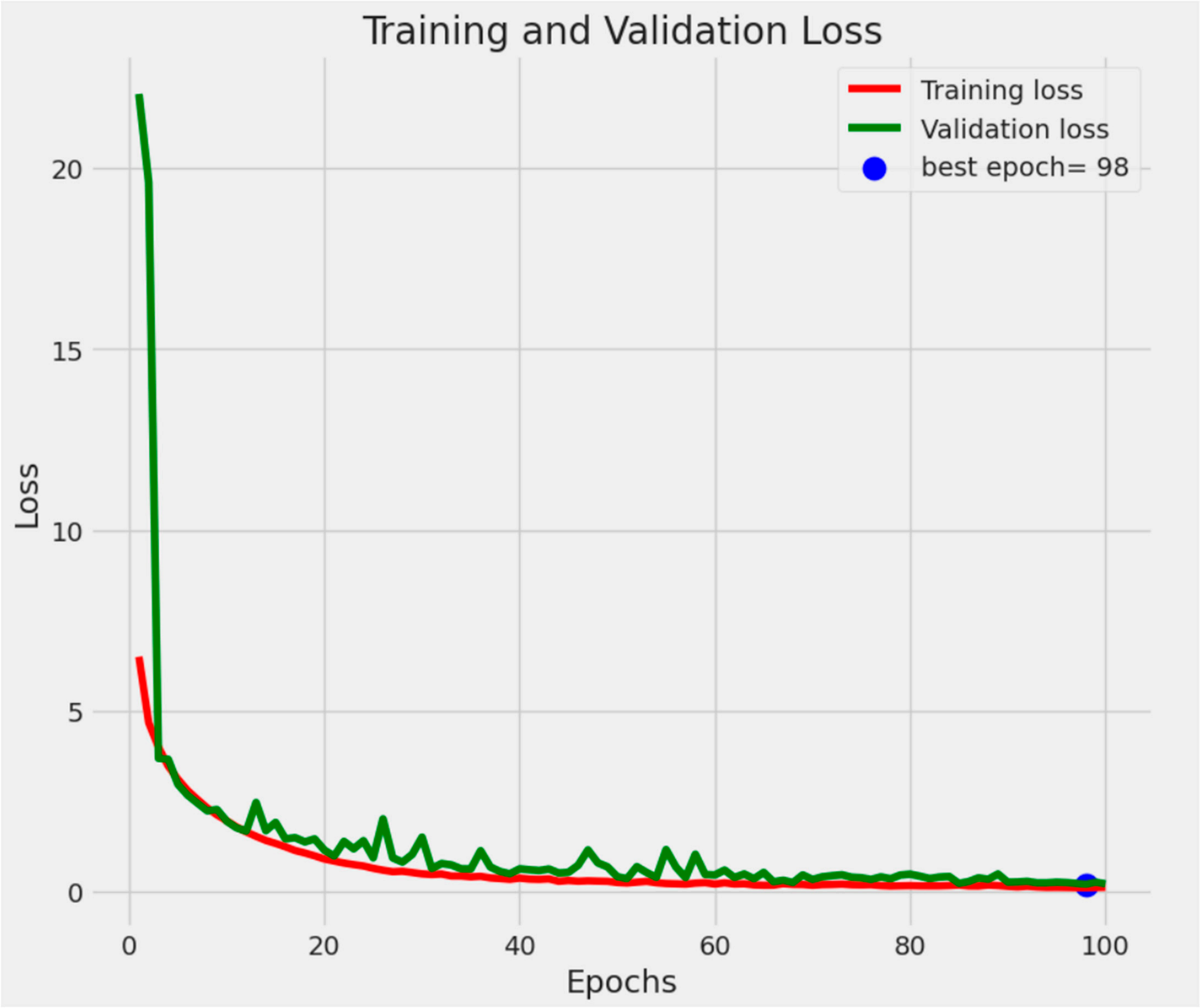
Training and Validation Loss
Training loss
Validation loss
best epoch= 98
Loss
Epochs
0
5
10
15
20
0
20
40
60
80
100

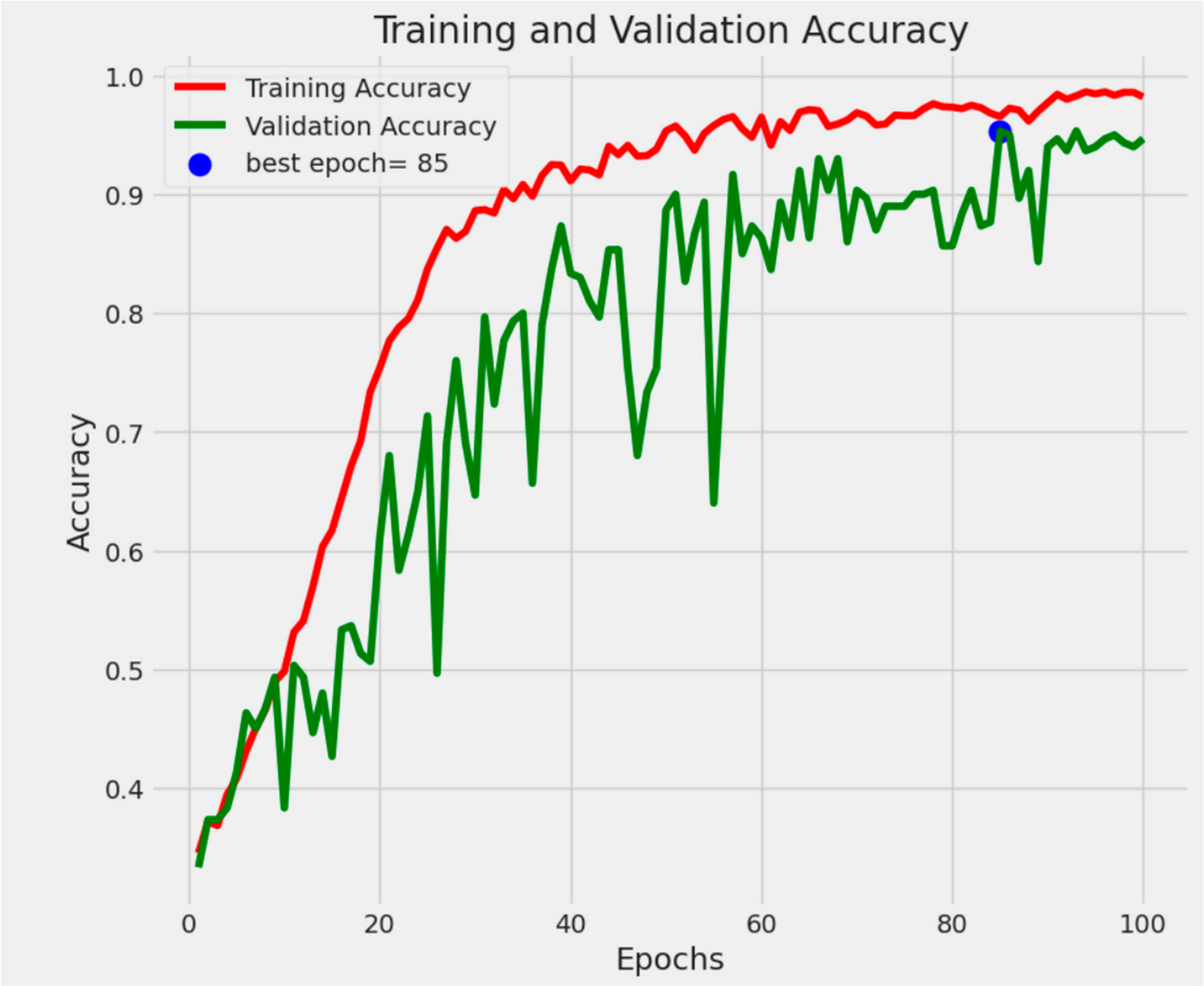

Training and Validation Accuracy
Training Accuracy
Validation Accuracy
best epoch= 85
1.0
0.9
0.8
0.7
0.6
0.5
0.4
Accuracy
0
20
40
60
80
100
Epochs

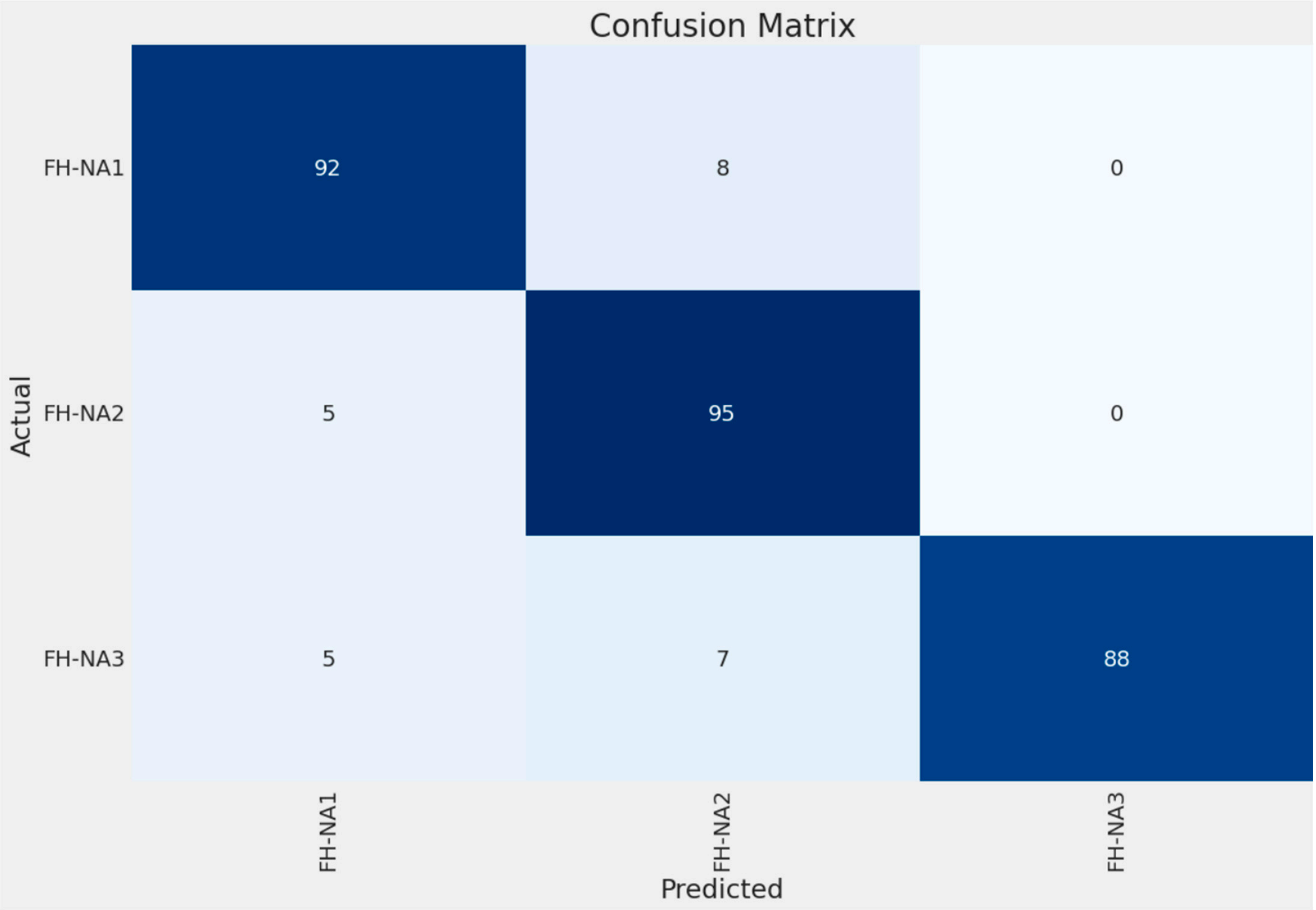

there were 25 errors in 300 tests for an accuracy of 91.67

Classification Report:

----

	precision	recall	f1-score	support
FH-NA1	0.9020	0.9200	0.9109	100
FH-NA2	0.8636	0.9500	0.9048	100
FH-NA3	1.0000	0.8800	0.9362	100
accuracy			0.9167	300
macro avg	0.9219	0.9167	0.9173	300
weighted avg	0.9219	0.9167	0.9173	300

DENSENET 201

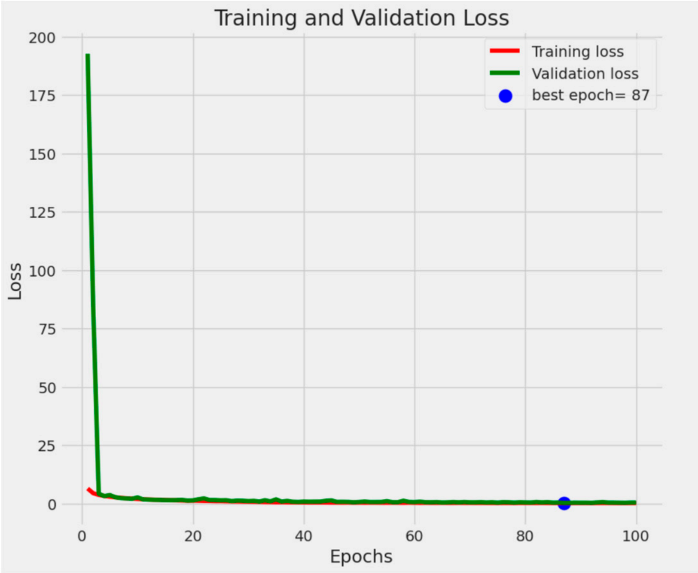

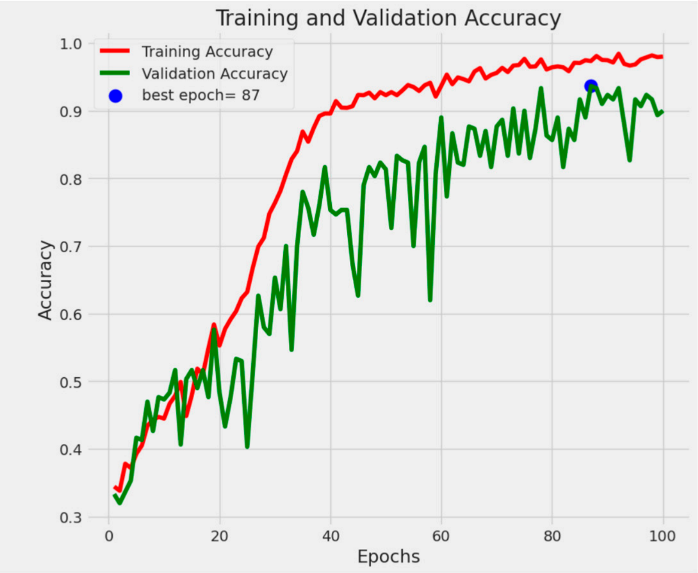

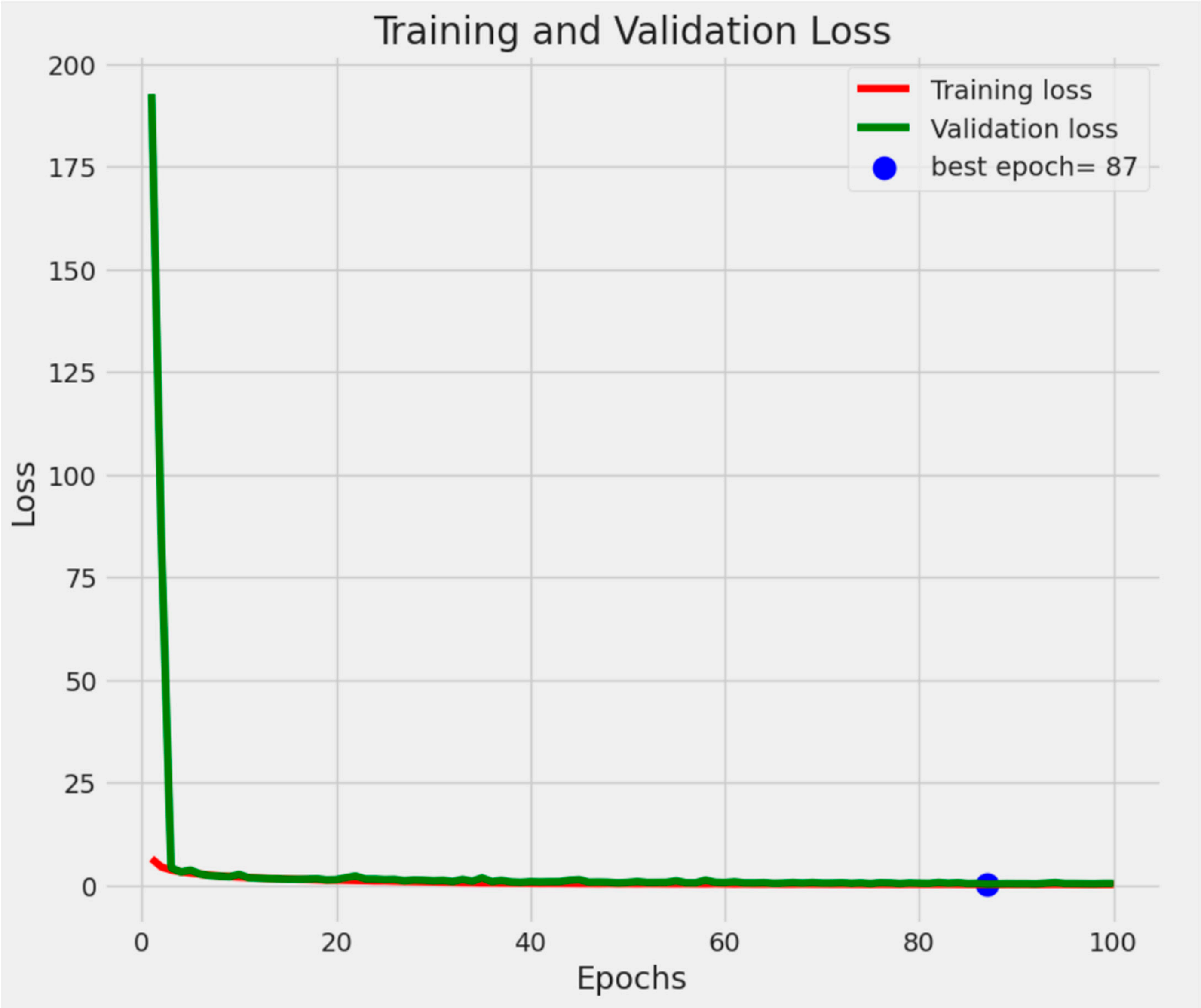

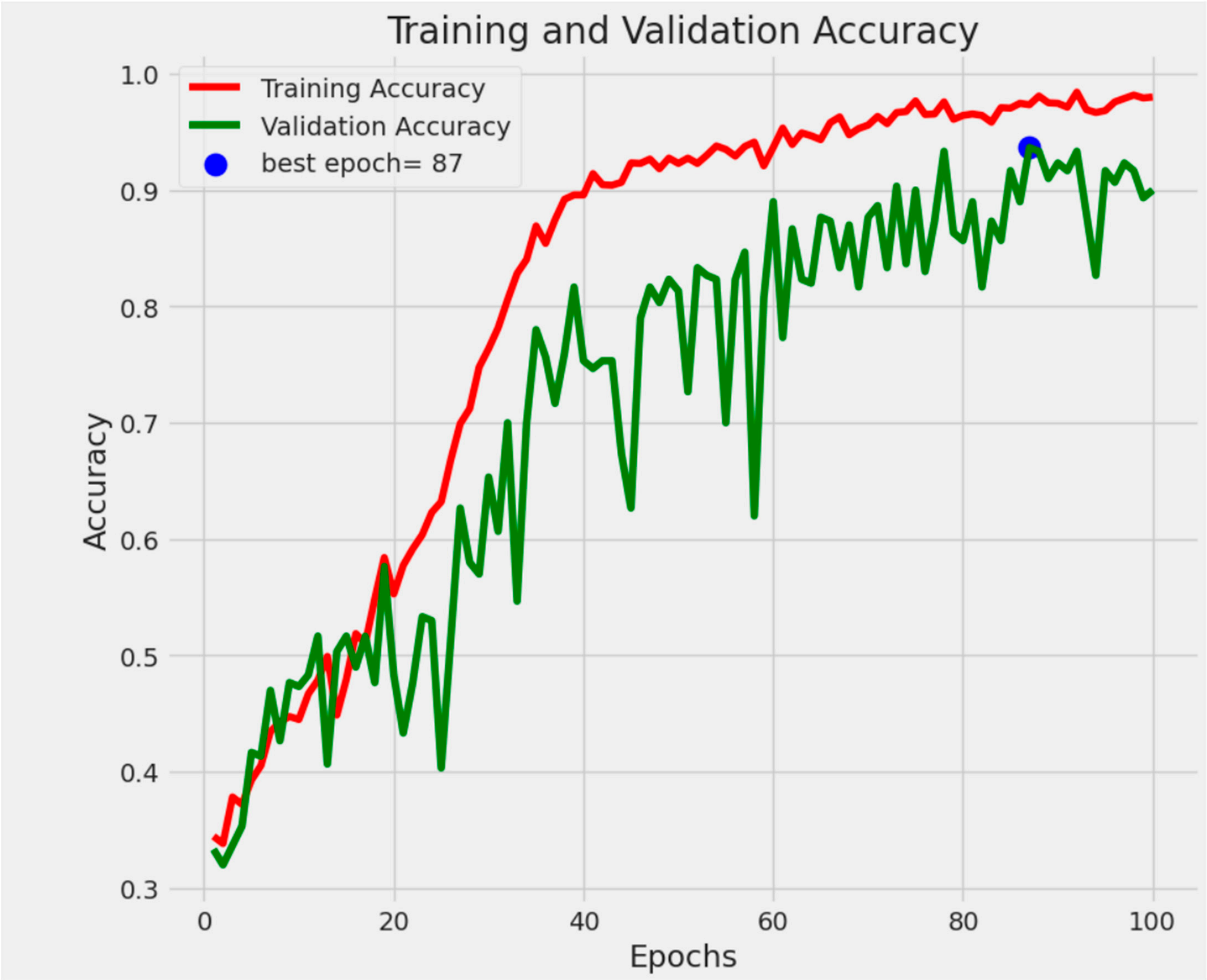
Training and Validation Accuracy
Training Accuracy
Validation Accuracy
best epoch= 87
Accuracy
Epochs
1.0
0.9
0.8
0.7
0.6
0.5
0.4
0.3
0
20
40
60
80
100

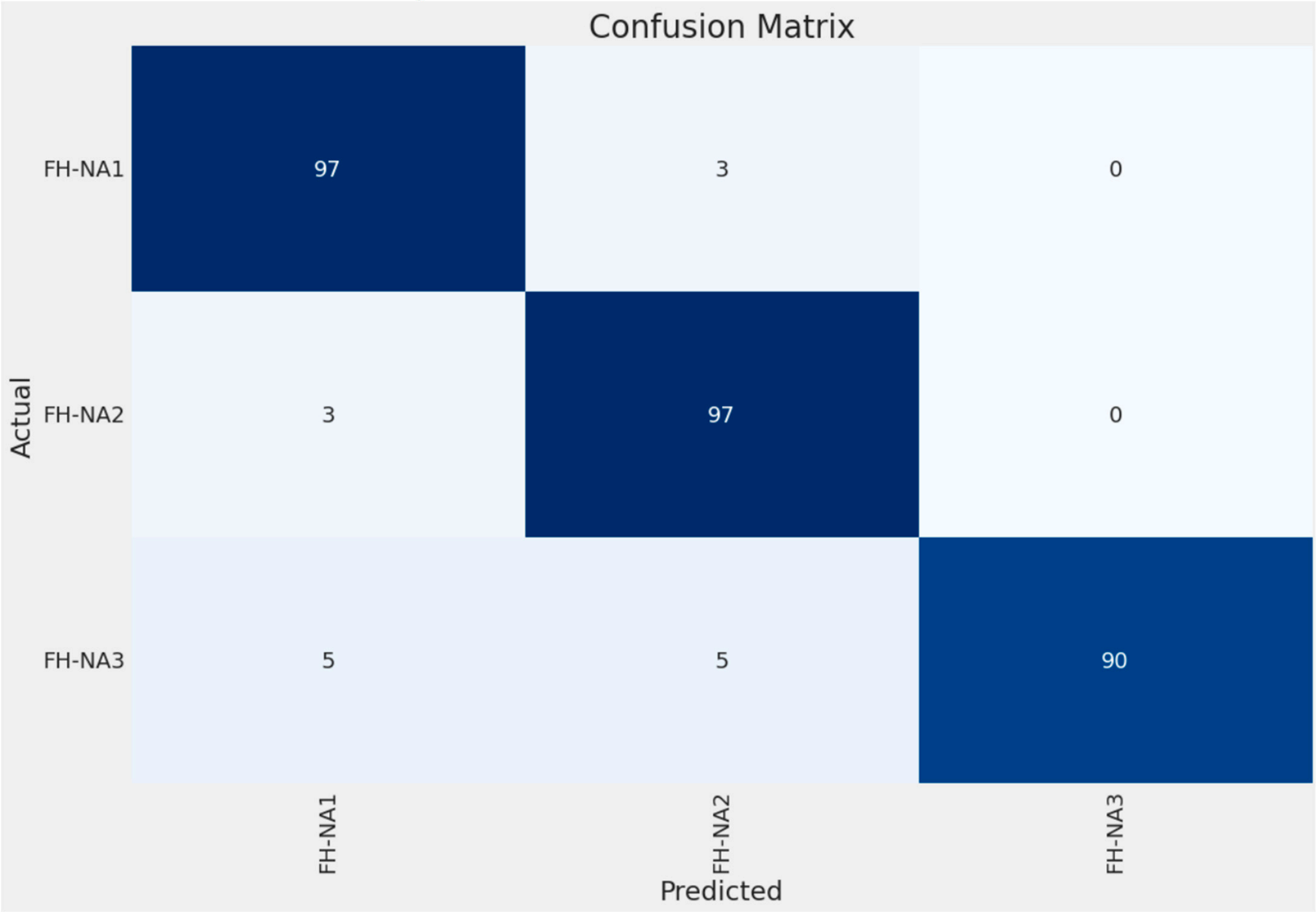

there were 16 errors in 300 tests for an accuracy of 94.67

Classification Report:
----

	precision	recall	f1-score	support
FH-NA1	0.9238	0.9700	0.9463	100
FH-NA2	0.9238	0.9700	0.9463	100
FH-NA3	1.0000	0.9000	0.9474	100
accuracy			0.9467	300
macro avg	0.9492	0.9467	0.9467	300
weighted avg	0.9492	0.9467	0.9467	300

EFFICIENTNET B0

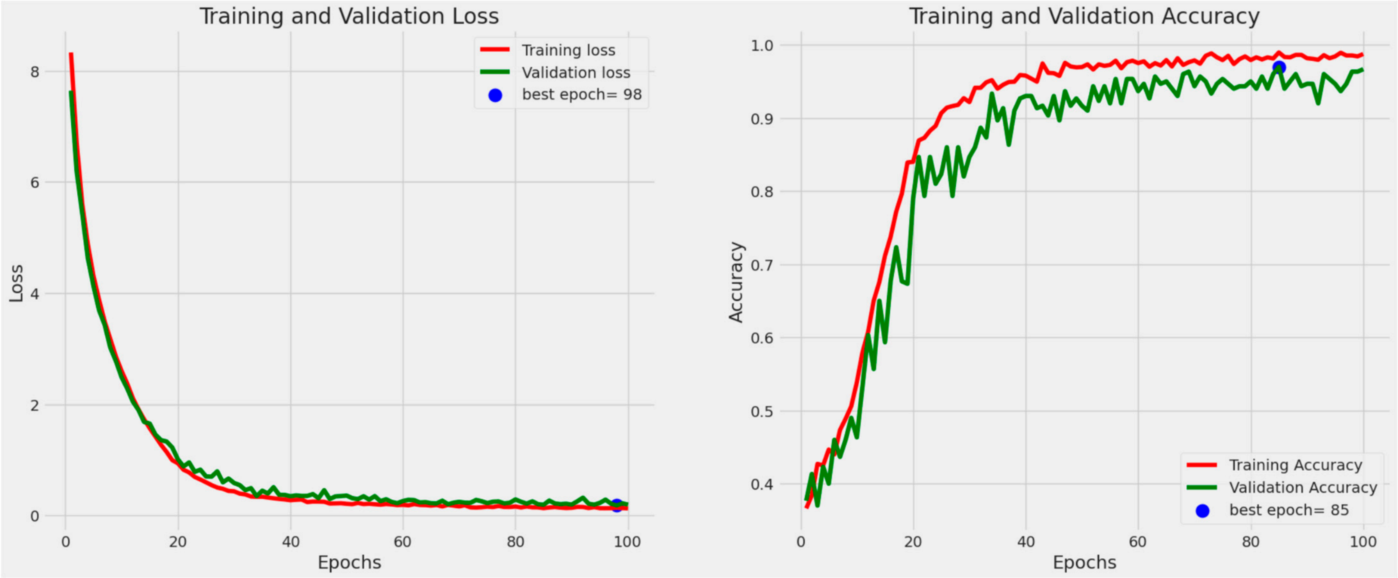

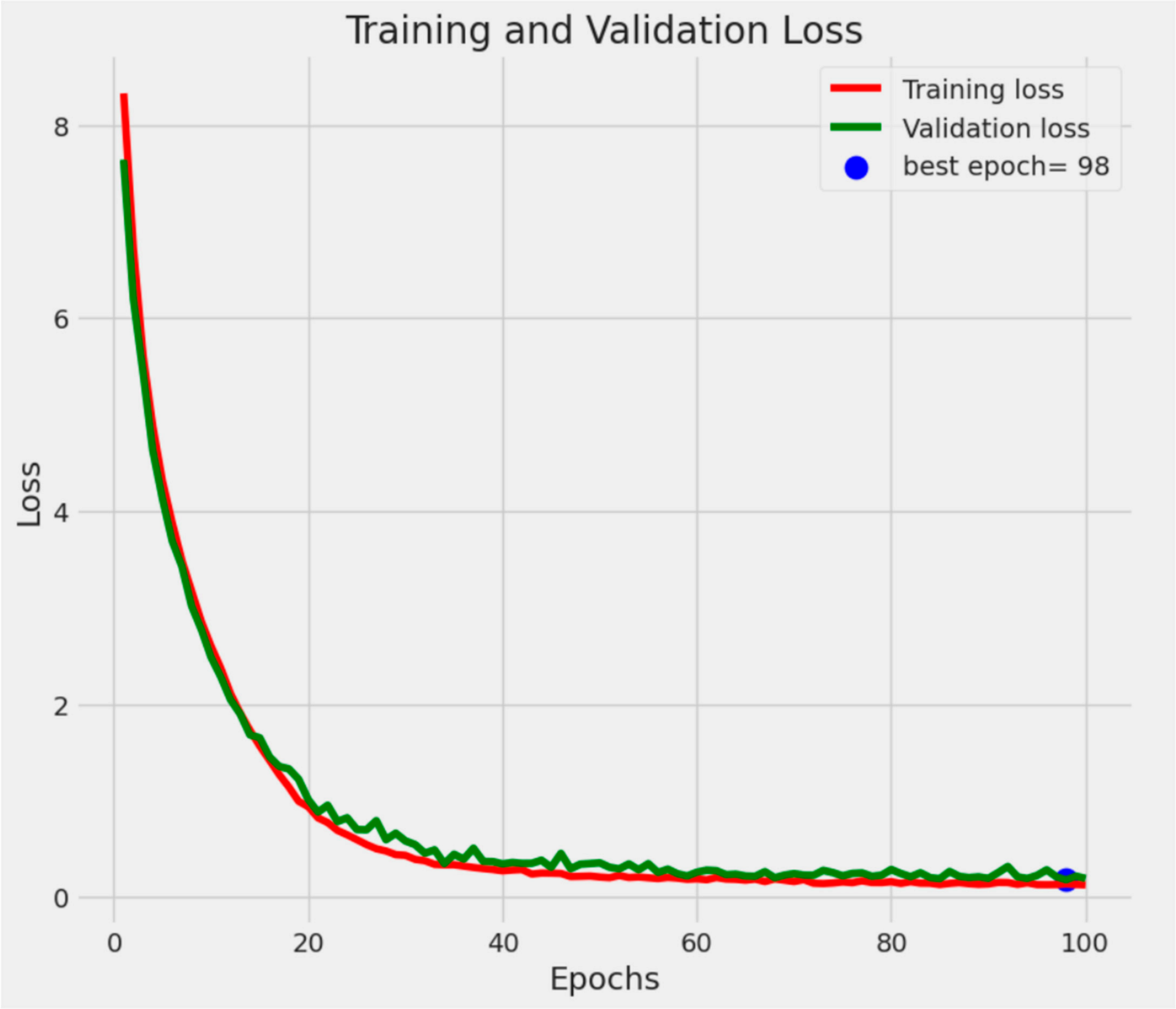

Training and Validation Loss
Training loss
Validation loss
best epoch= 98
8
6
4
2
0
Loss
0
20
40
60
80
100
Epochs

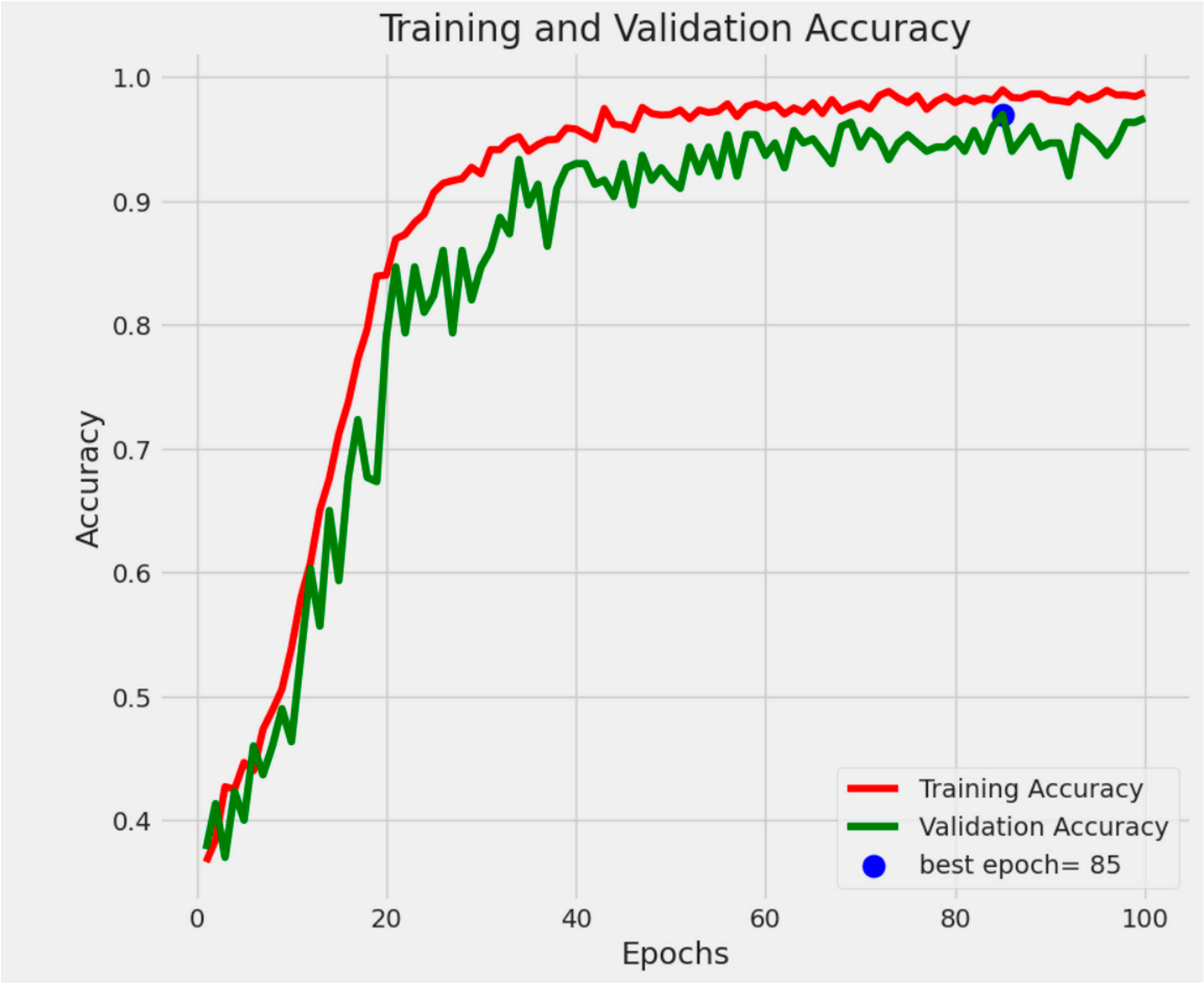

Training and Validation Accuracy
Accuracy
Epochs
1.0
0.9
0.8
0.7
0.6
0.5
0.4
0
20
40
60
80
100
Training Accuracy
Validation Accuracy
best epoch= 85

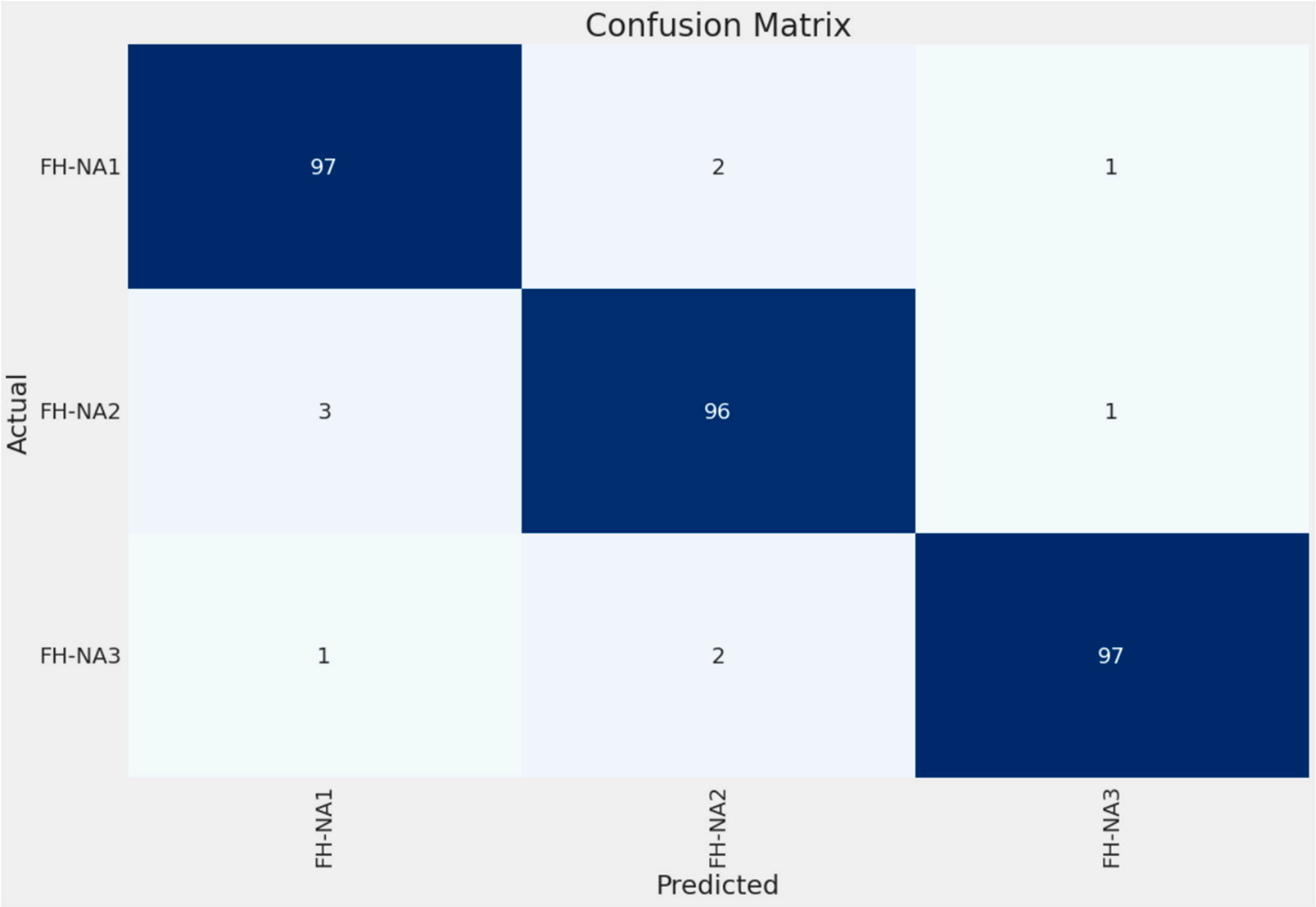

there were 10 errors in 300 tests for an accuracy of 96.67

Classification Report:
----

	precision	recall	f1-score	support
FH-NA1	0.9604	0.9700	0.9652	100
FH-NA2	0.9600	0.9600	0.9600	100
FH-NA3	0.9798	0.9700	0.9749	100
accuracy			0.9667	300
macro avg	0.9667	0.9667	0.9667	300
weighted avg	0.9667	0.9667	0.9667	300

XCEPTION

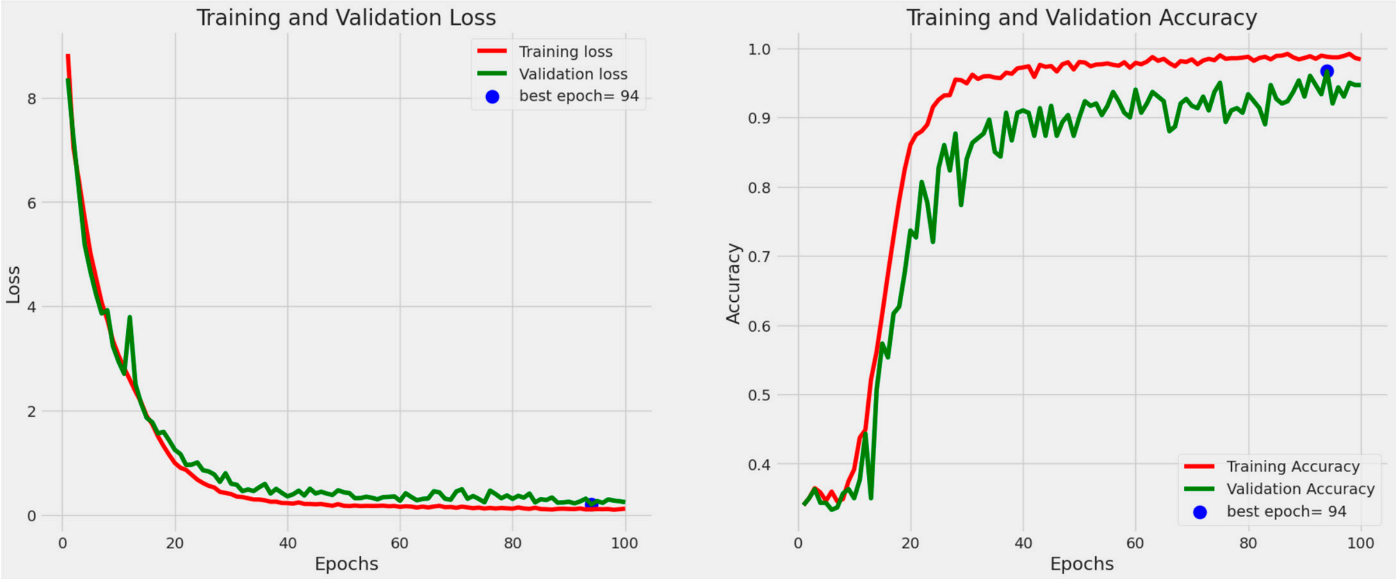
Training and Validation Loss
Training loss
Validation loss
best epoch= 94
Loss
Epochs
Training and Validation Accuracy
Training Accuracy
Validation Accuracy
best epoch= 94
Accuracy
Epochs

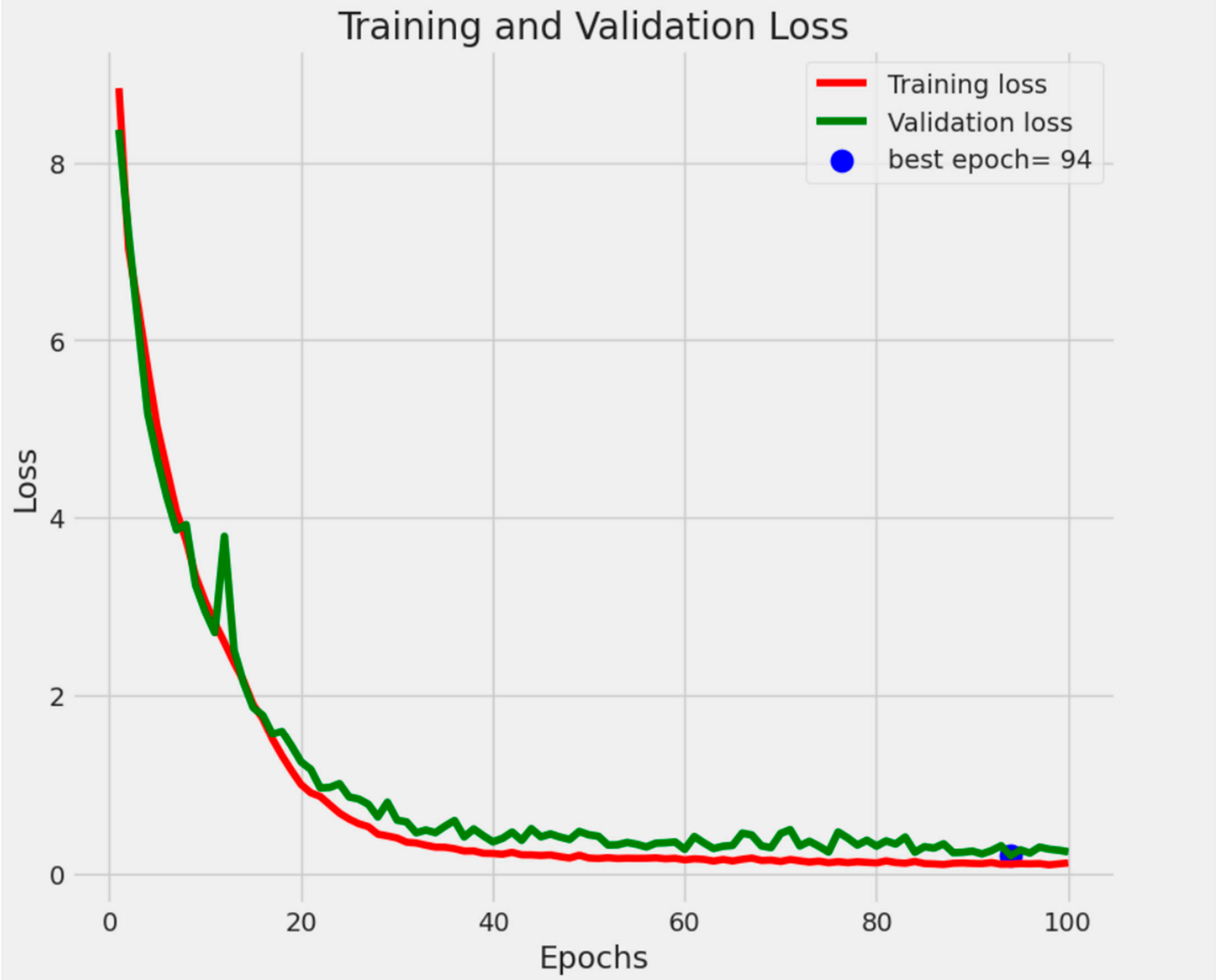
Training and Validation Loss
Training loss
Validation loss
best epoch= 94
Loss
Epochs

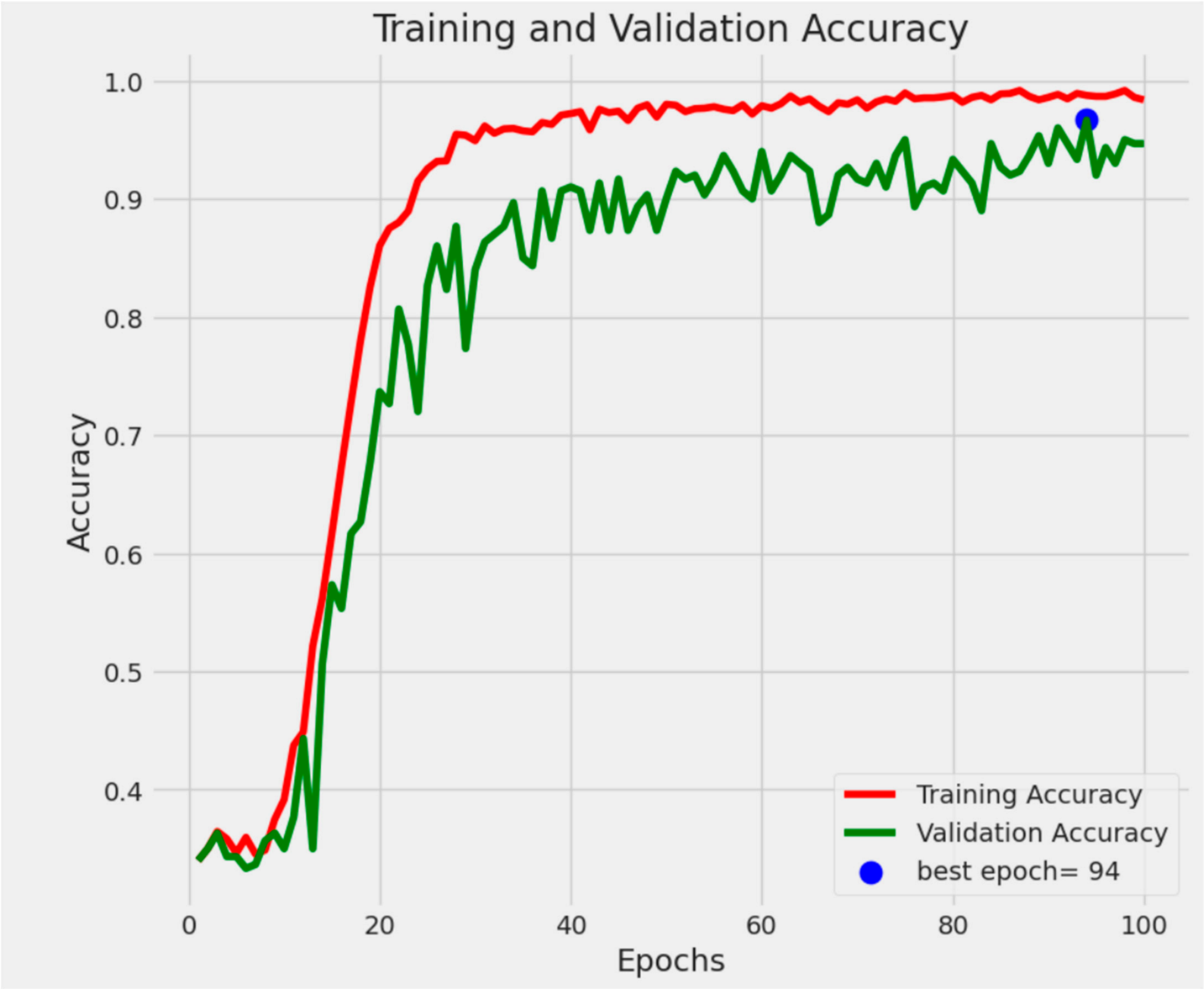

Training and Validation Accuracy
Accuracy
Epochs
1.0
0.9
0.8
0.7
0.6
0.5
0.4
0
20
40
60
80
100
Training Accuracy
Validation Accuracy
best epoch= 94

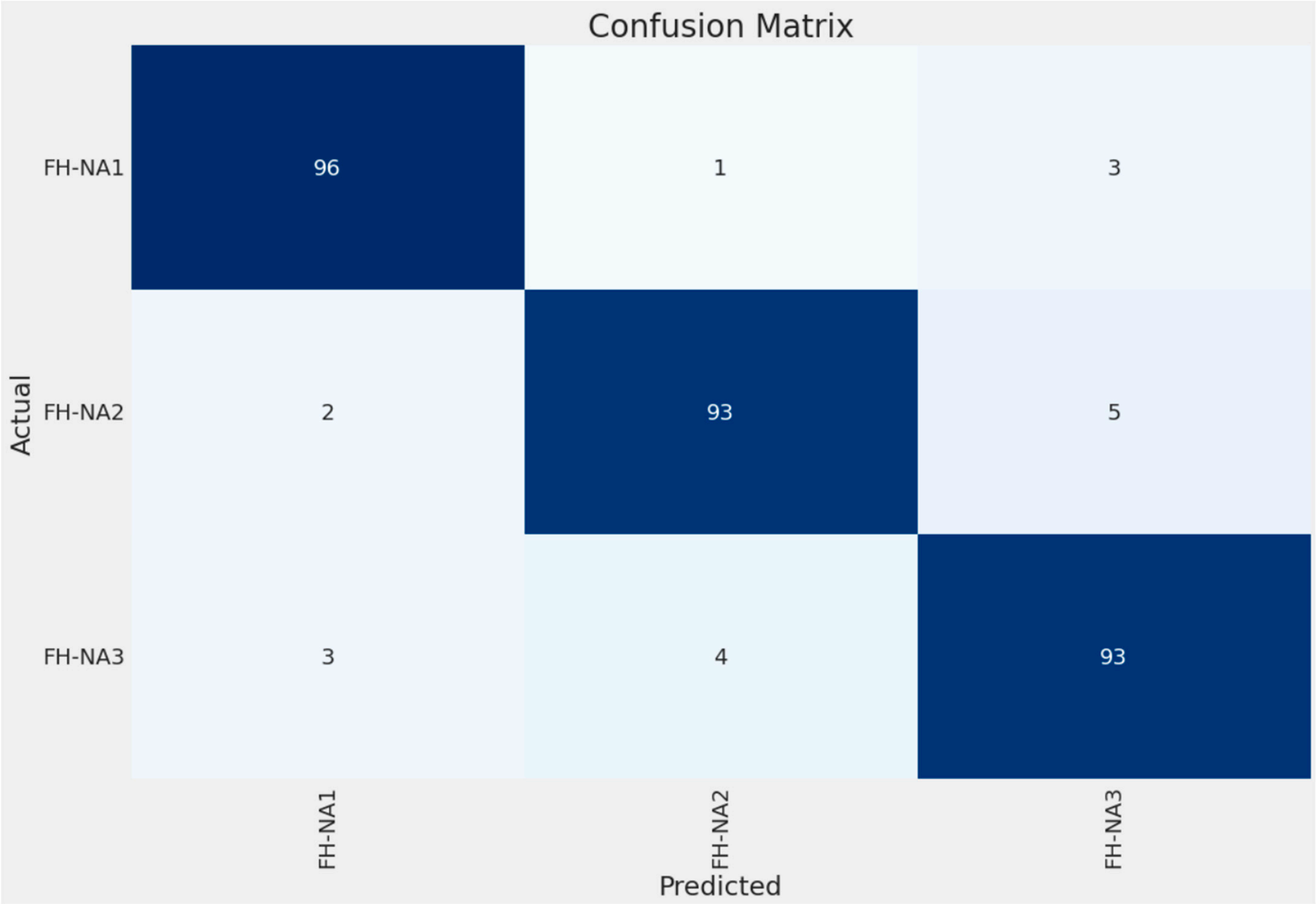


```
there were 18 errors in 300 tests for an accuracy of  94.00

Classification Report:
----------------------
              precision    recall  f1-score   support

      FH-NA1     0.9505    0.9600    0.9552       100
      FH-NA2     0.9490    0.9300    0.9394       100
      FH-NA3     0.9208    0.9300    0.9254       100

    accuracy                         0.9400       300
   macro avg     0.9401    0.9400    0.9400       300
weighted avg     0.9401    0.9400    0.9400       300
```

VGG16: 30.67

VGG19:34.33

NASNETMOBILE

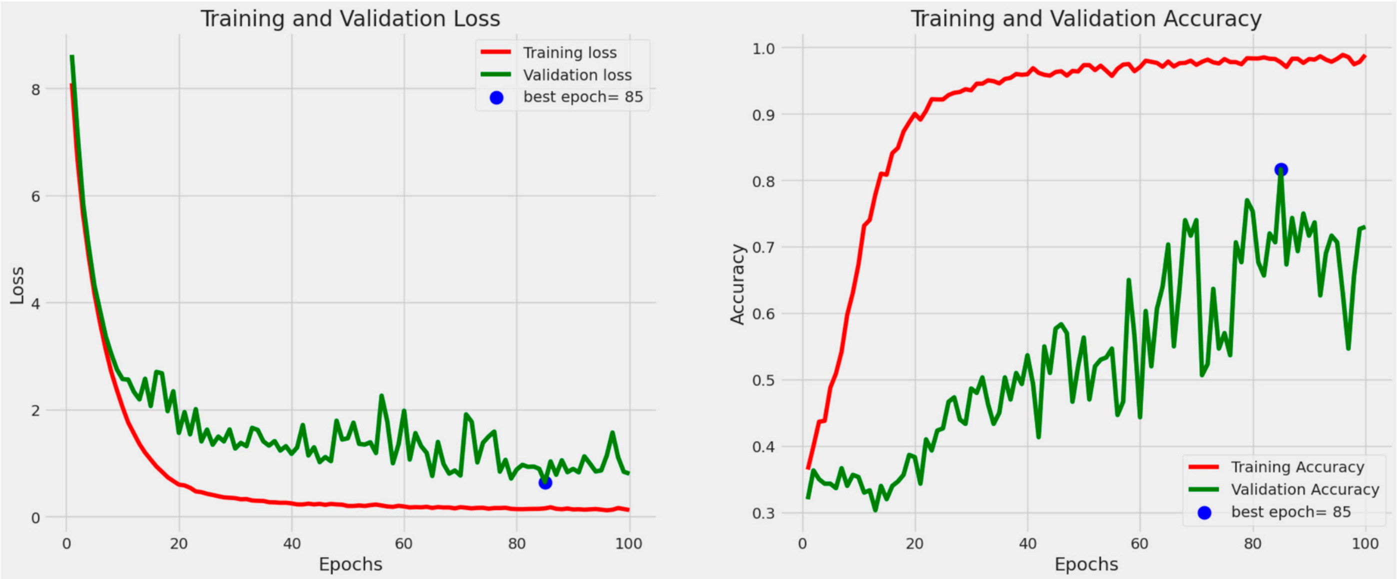

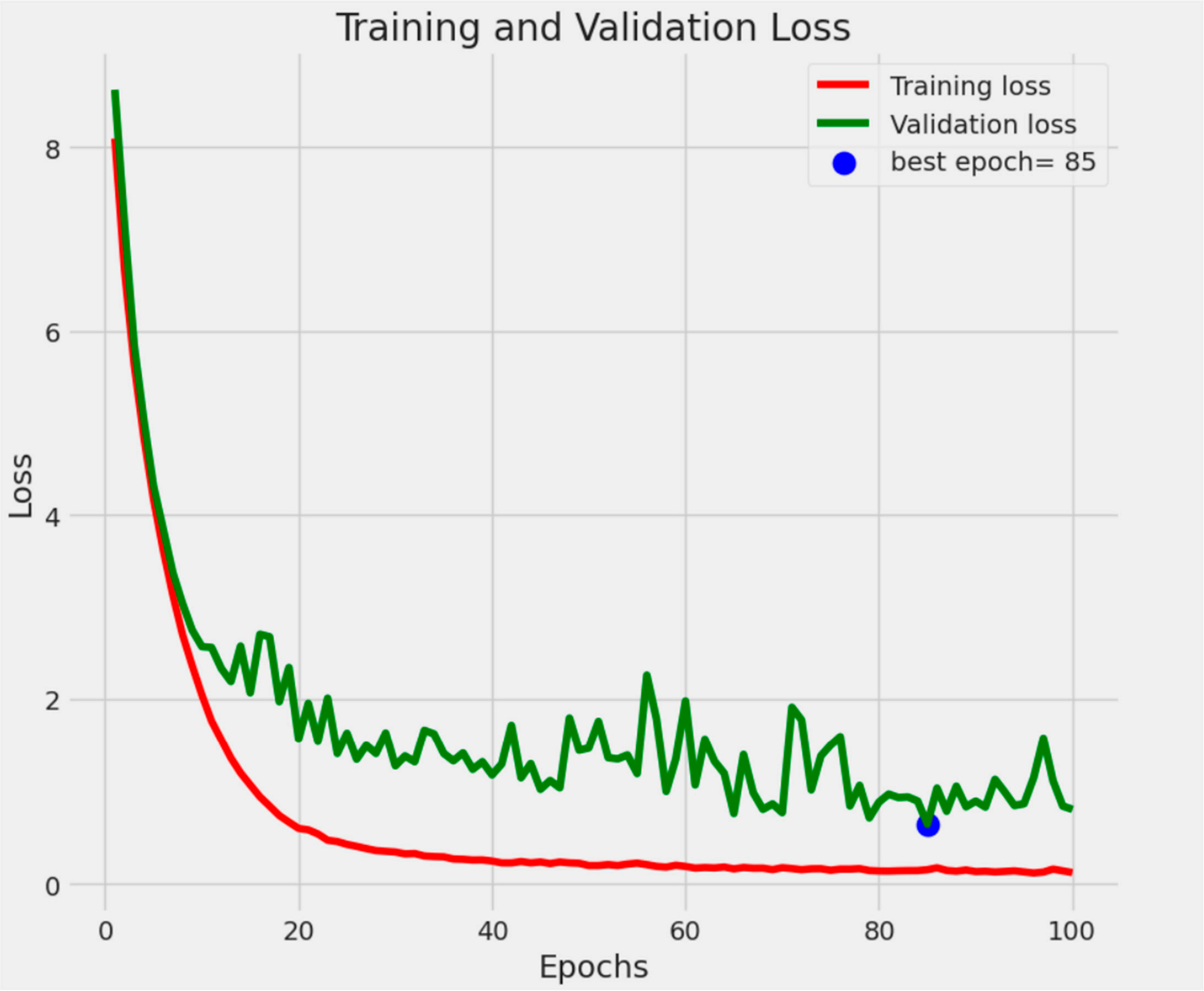

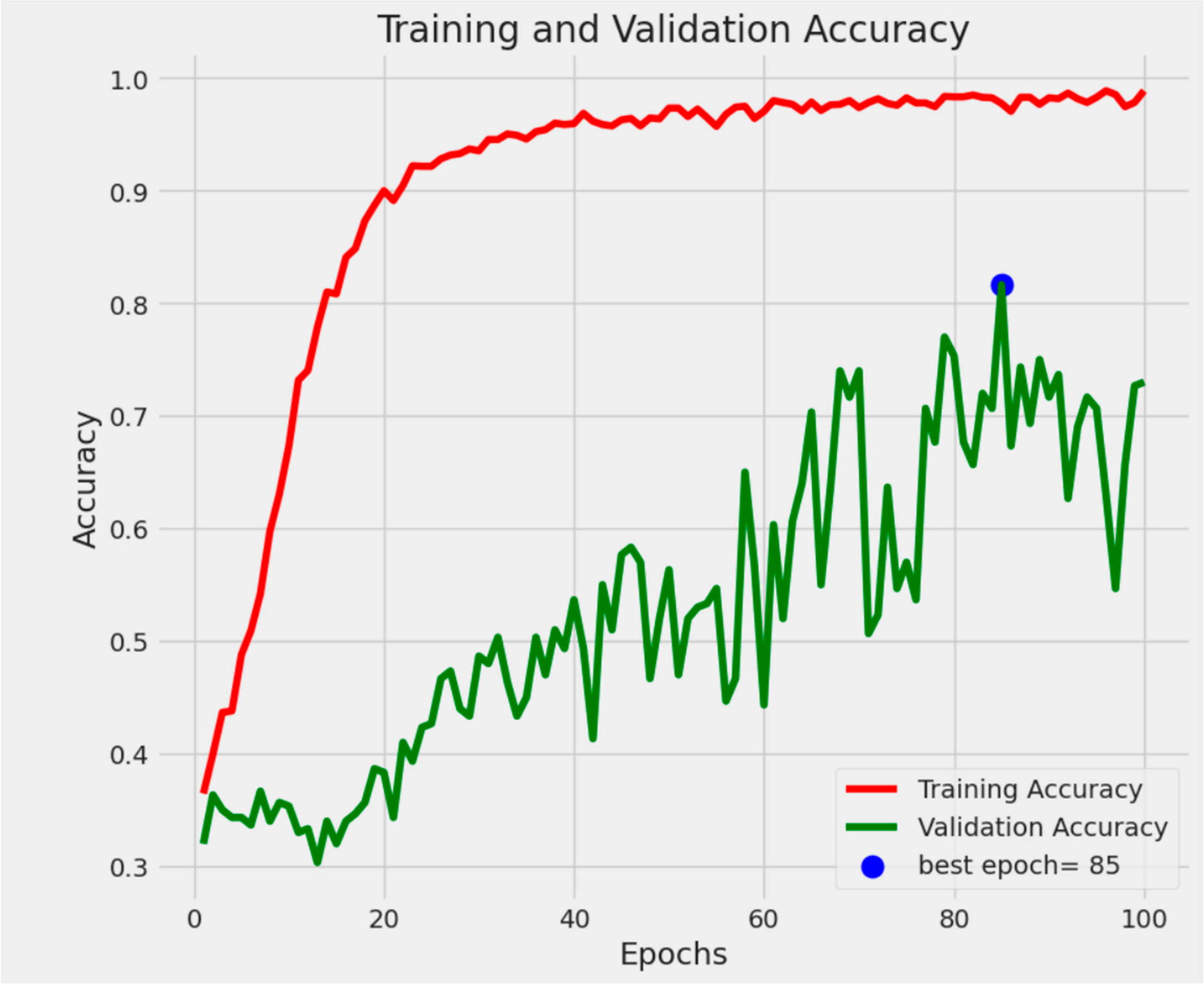

Training and Validation Accuracy
Accuracy
Epochs
Training Accuracy
Validation Accuracy
best epoch= 85
1.0
0.9
0.8
0.7
0.6
0.5
0.4
0.3
0
20
40
60
80
100

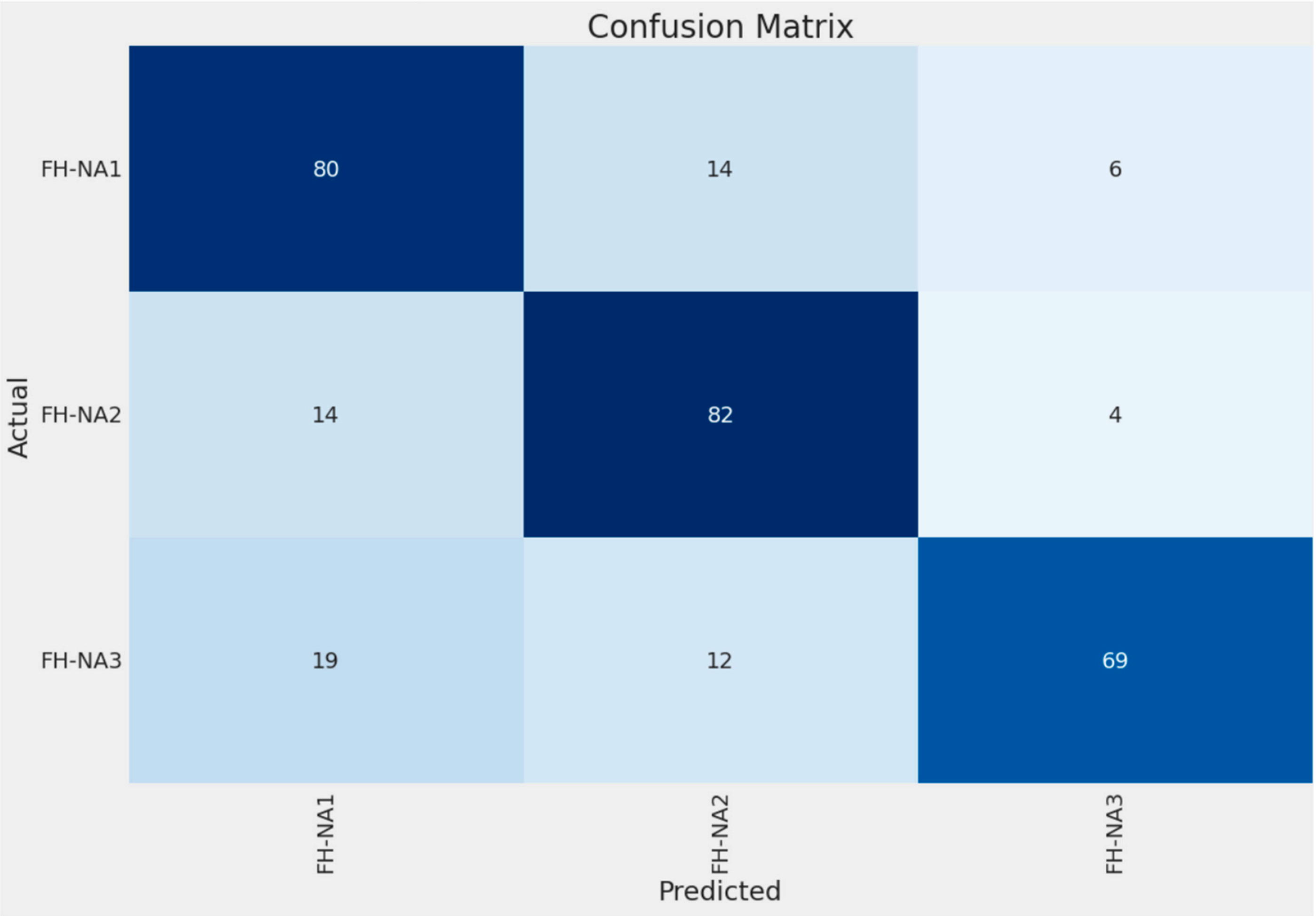


```
there were 69 errors in 300 tests for an accuracy of  77.00

Classification Report:
----------------------
              precision    recall  f1-score   support

      FH-NA1     0.7080    0.8000    0.7512       100
      FH-NA2     0.7593    0.8200    0.7885       100
      FH-NA3     0.8734    0.6900    0.7709       100

    accuracy                         0.7700       300
   macro avg     0.7802    0.7700    0.7702       300
weighted avg     0.7802    0.7700    0.7702       300
```

RESNET101

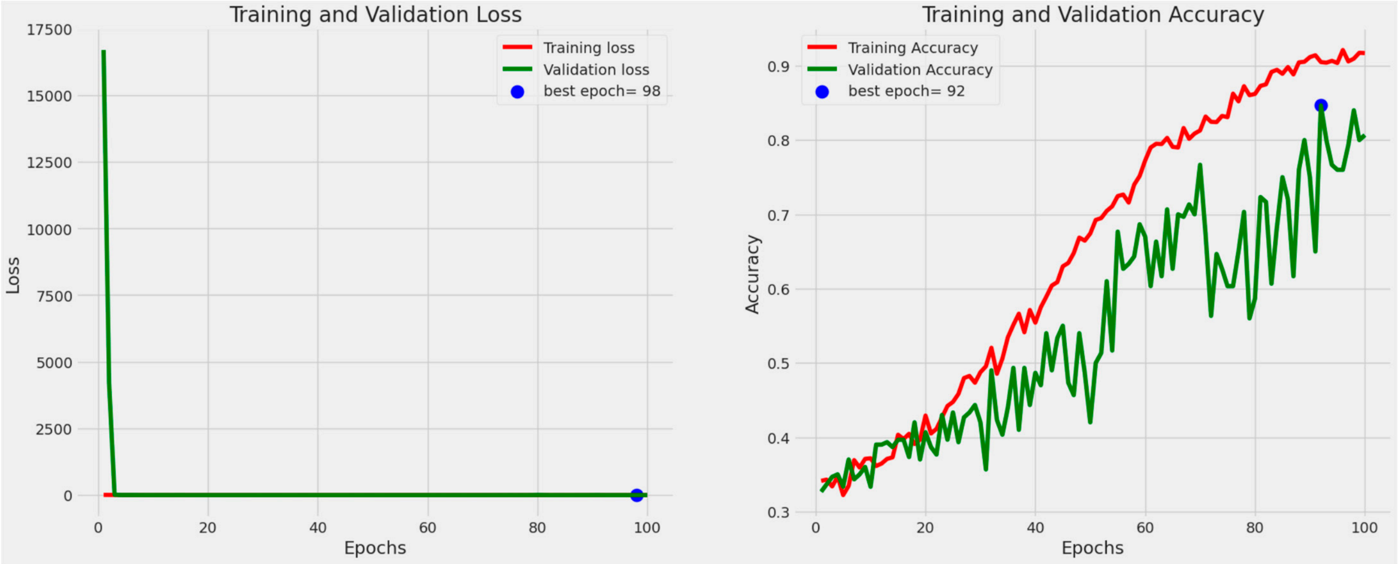
Training and Validation Loss
Training loss
Validation loss
best epoch= 98
Loss
Epochs
17500
15000
12500
10000
7500
5000
2500
0
0
20
40
60
80
100
Training and Validation Accuracy
Training Accuracy
Validation Accuracy
best epoch= 92
Accuracy
Epochs
0.9
0.8
0.7
0.6
0.5
0.4
0.3
0
20
40
60
80
100

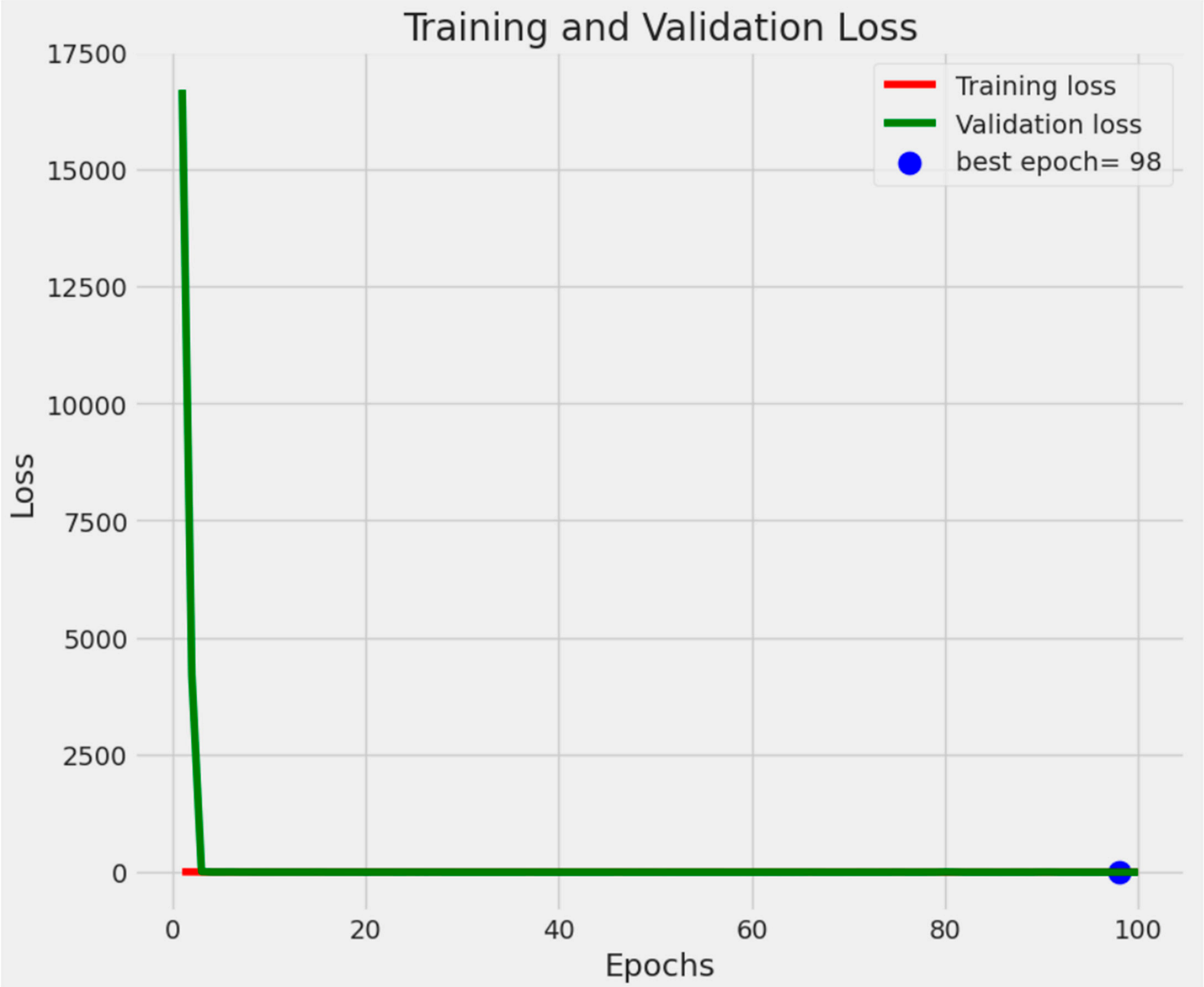
Training and Validation Loss
Training loss
Validation loss
best epoch= 98
Loss
Epochs
17500
15000
12500
10000
7500
5000
2500
0
0
20
40
60
80
100

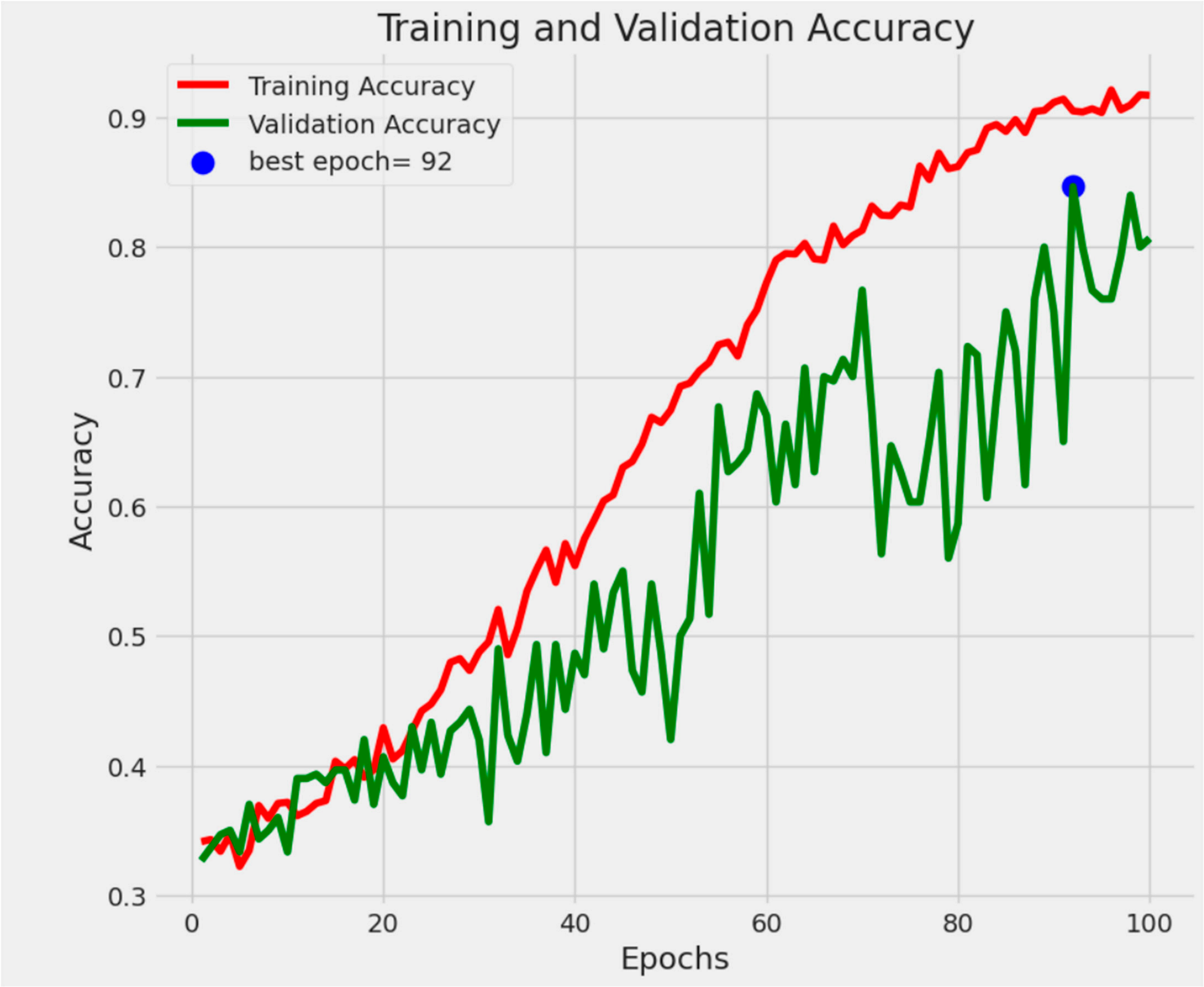

Training and Validation Accuracy
Training Accuracy
Validation Accuracy
best epoch= 92
Accuracy
Epochs
0.3
0.4
0.5
0.6
0.7
0.8
0.9
0
20
40
60
80
100

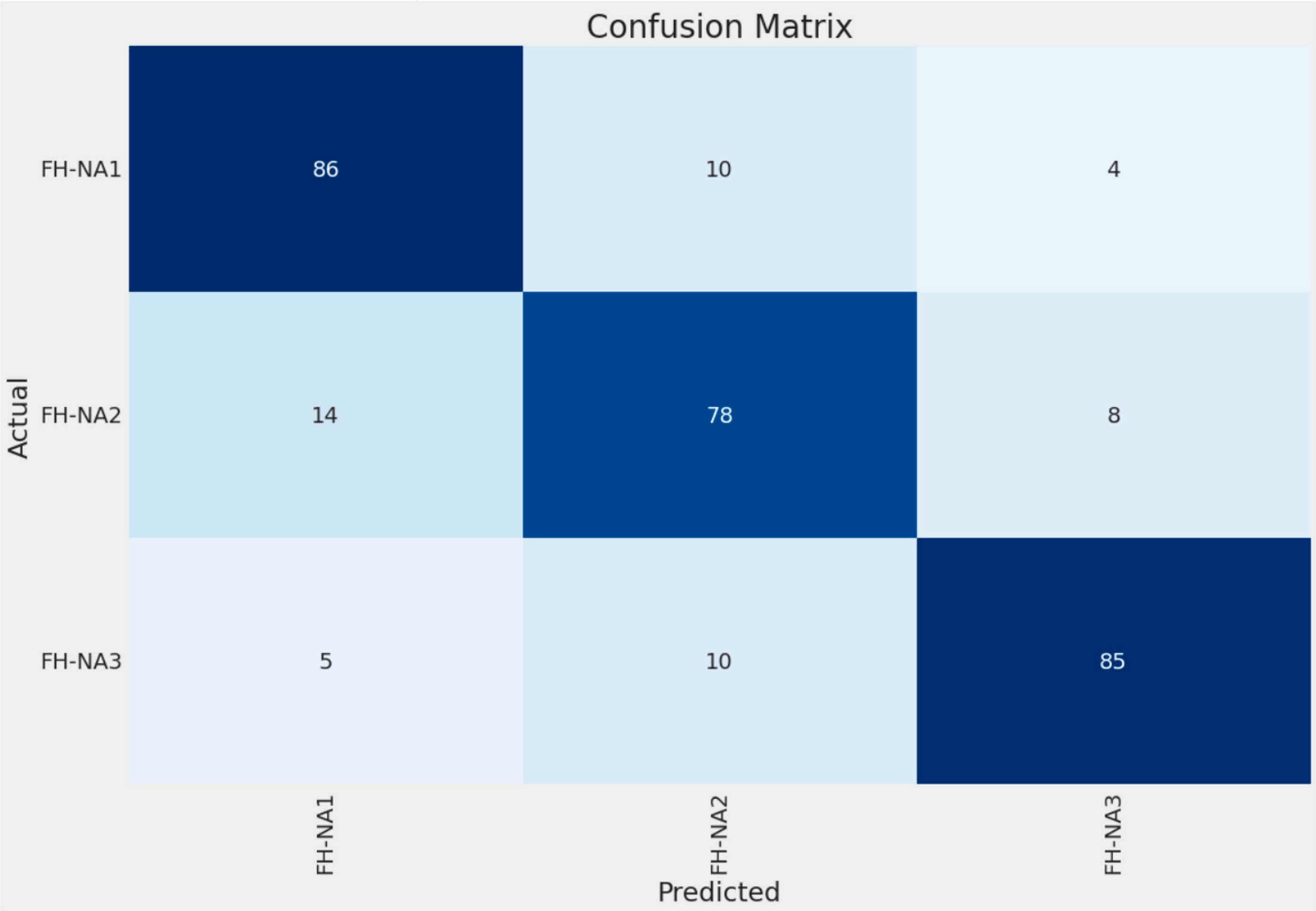

there were 51 errors in 300 tests for an accuracy of 83.00

Classification Report:
----

	precision	recall	f1-score	support
FH-NA1	0.8190	0.8600	0.8390	100
FH-NA2	0.7959	0.7800	0.7879	100
FH-NA3	0.8763	0.8500	0.8629	100
accuracy			0.8300	300
macro avg	0.8304	0.8300	0.8299	300
weighted avg	0.8304	0.8300	0.8299	300

RESNET152

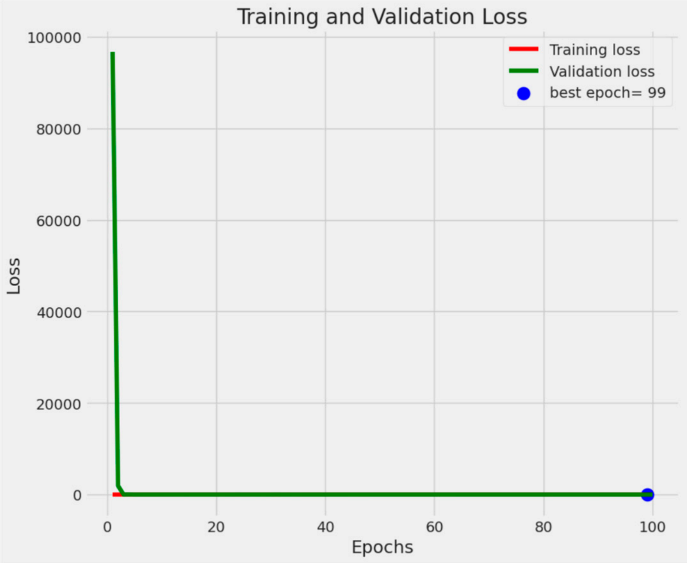

Training and Validation Loss
Training loss
Validation loss
best epoch= 99
Loss
Epochs
100000
80000
60000
40000
20000
0
0
20
40
60
80
100

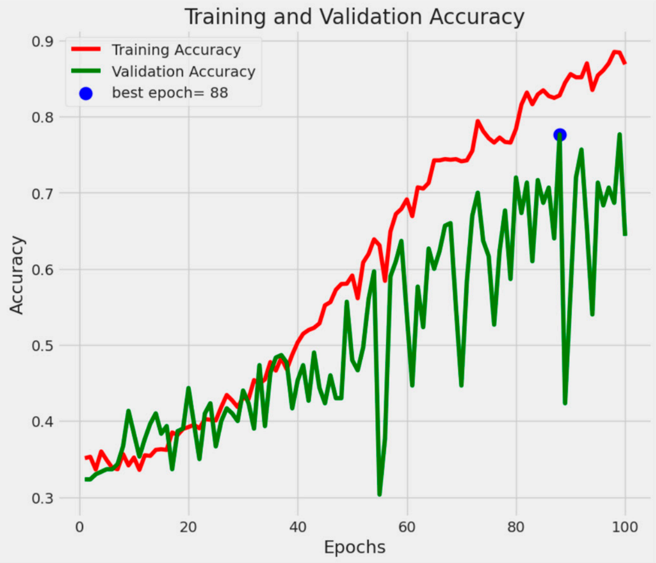

Training and Validation Accuracy
Training Accuracy
Validation Accuracy
best epoch= 88
Accuracy
Epochs
0.9
0.8
0.7
0.6
0.5
0.4
0.3
0
20
40
60
80
100

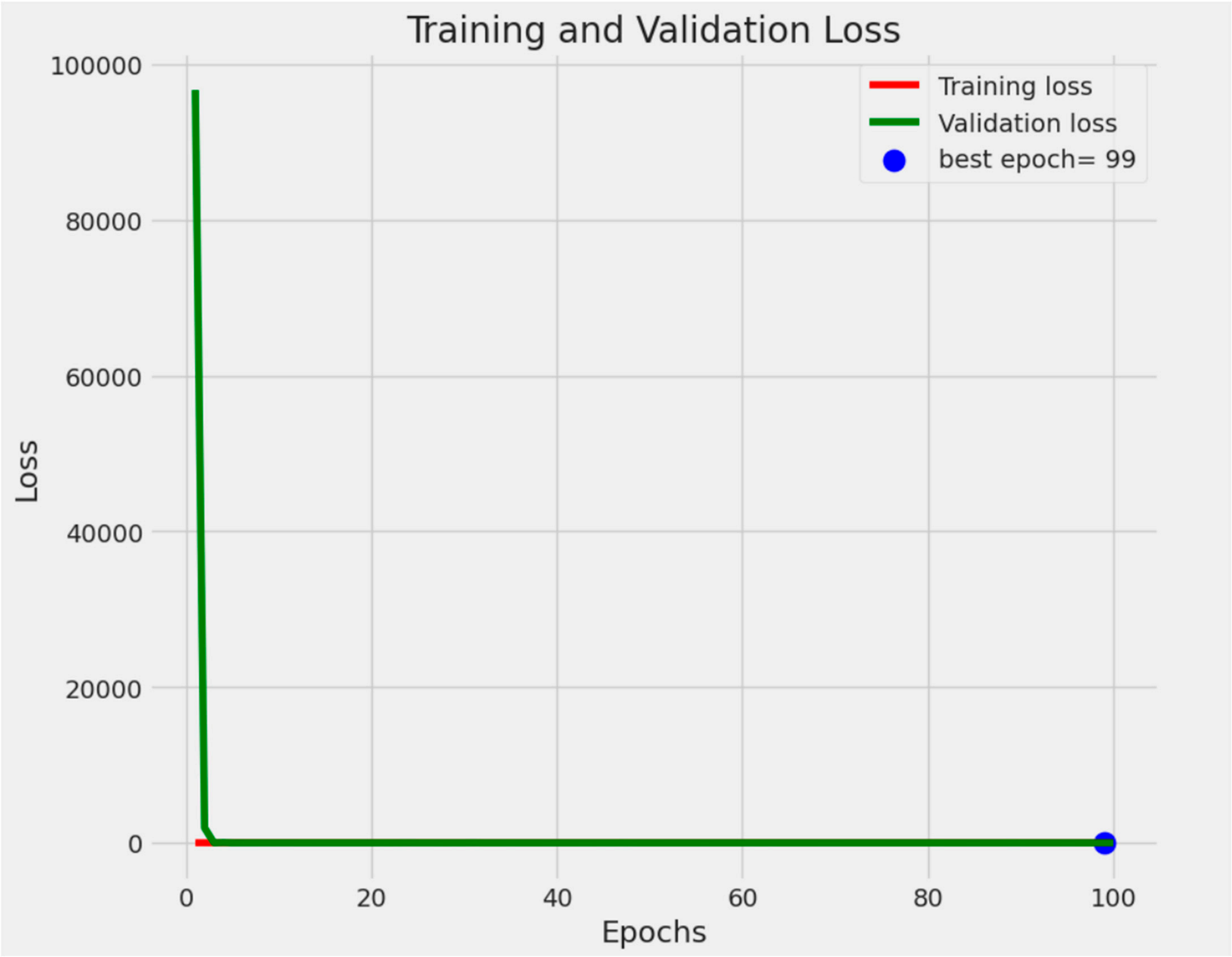

Training and Validation Loss
Training loss
Validation loss
best epoch= 99
Loss
Epochs
100000
80000
60000
40000
20000
0
0
20
40
60
80
100

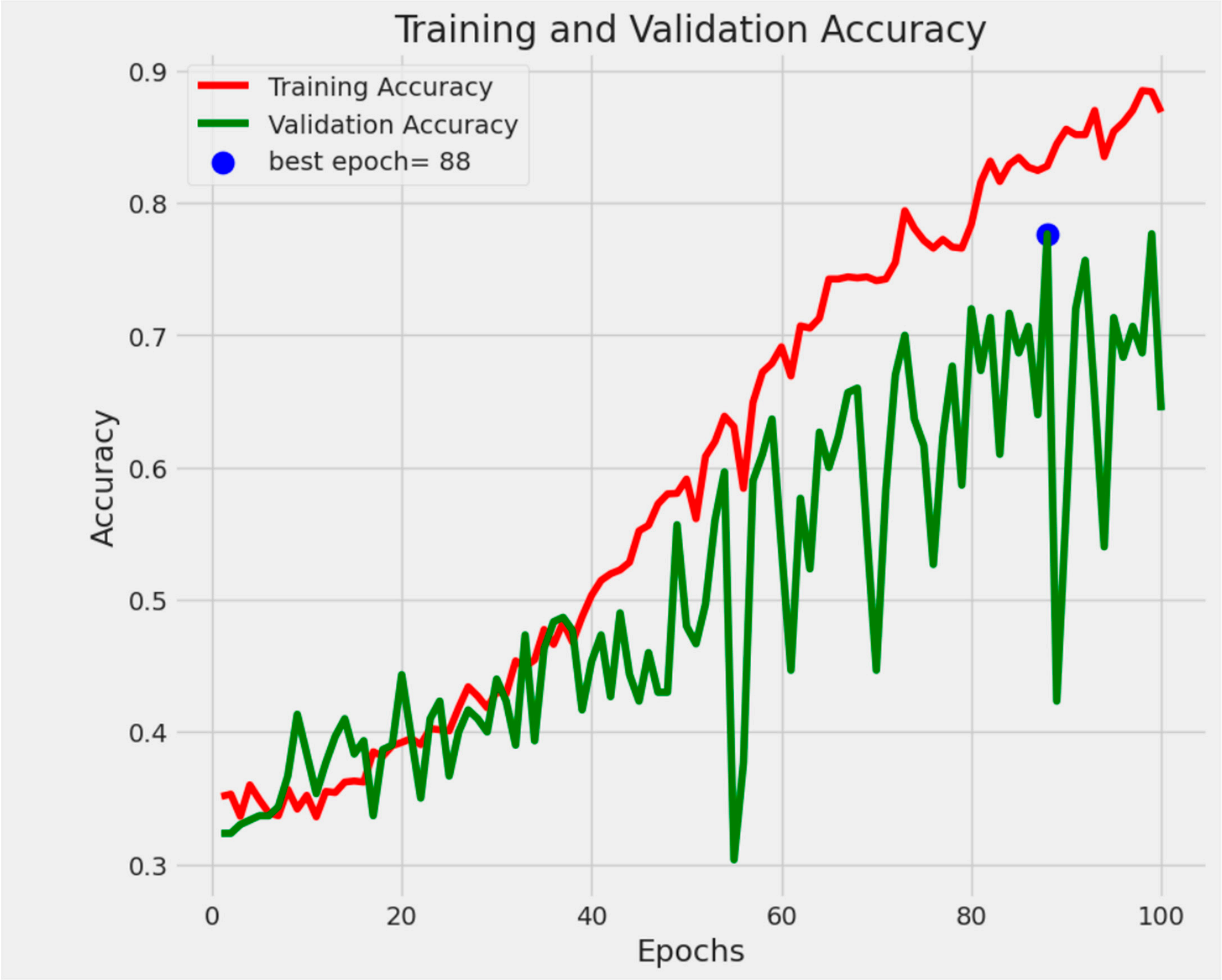
Training and Validation Accuracy
Training Accuracy
Validation Accuracy
best epoch= 88
Accuracy
Epochs
0.3
0.4
0.5
0.6
0.7
0.8
0.9
0
20
40
60
80
100

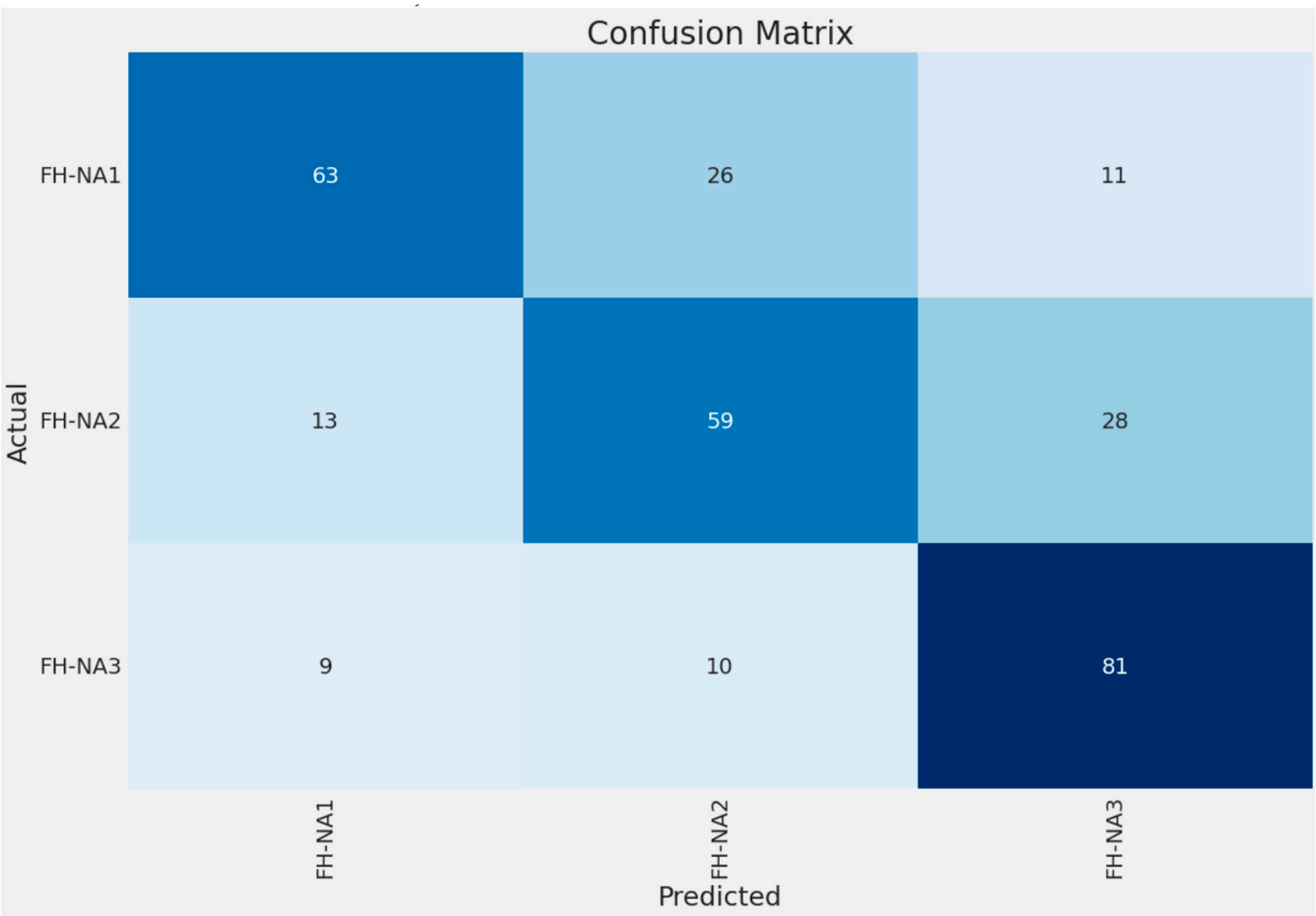

there were 97 errors in 300 tests for an accuracy of 67.67

Classification Report:
----

	precision	recall	f1-score	support
FH-NA1	0.7412	0.6300	0.6811	100
FH-NA2	0.6211	0.5900	0.6051	100
FH-NA3	0.6750	0.8100	0.7364	100
accuracy			0.6767	300
macro avg	0.6791	0.6767	0.6742	300
weighted avg	0.6791	0.6767	0.6742	300

RESNET50

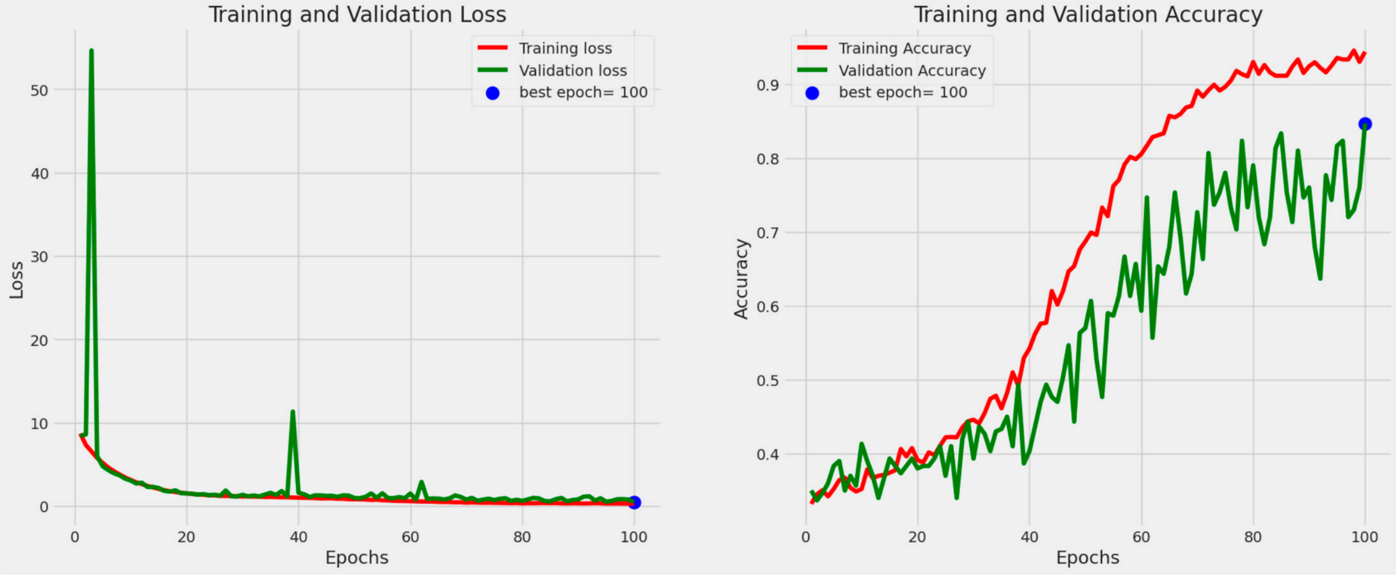
Training and Validation Loss
Training loss
Validation loss
best epoch= 100
Loss
Epochs
0
10
20
30
40
50
0
20
40
60
80
100
Training and Validation Accuracy
Training Accuracy
Validation Accuracy
best epoch= 100
Accuracy
Epochs
0.4
0.5
0.6
0.7
0.8
0.9
0
20
40
60
80
100

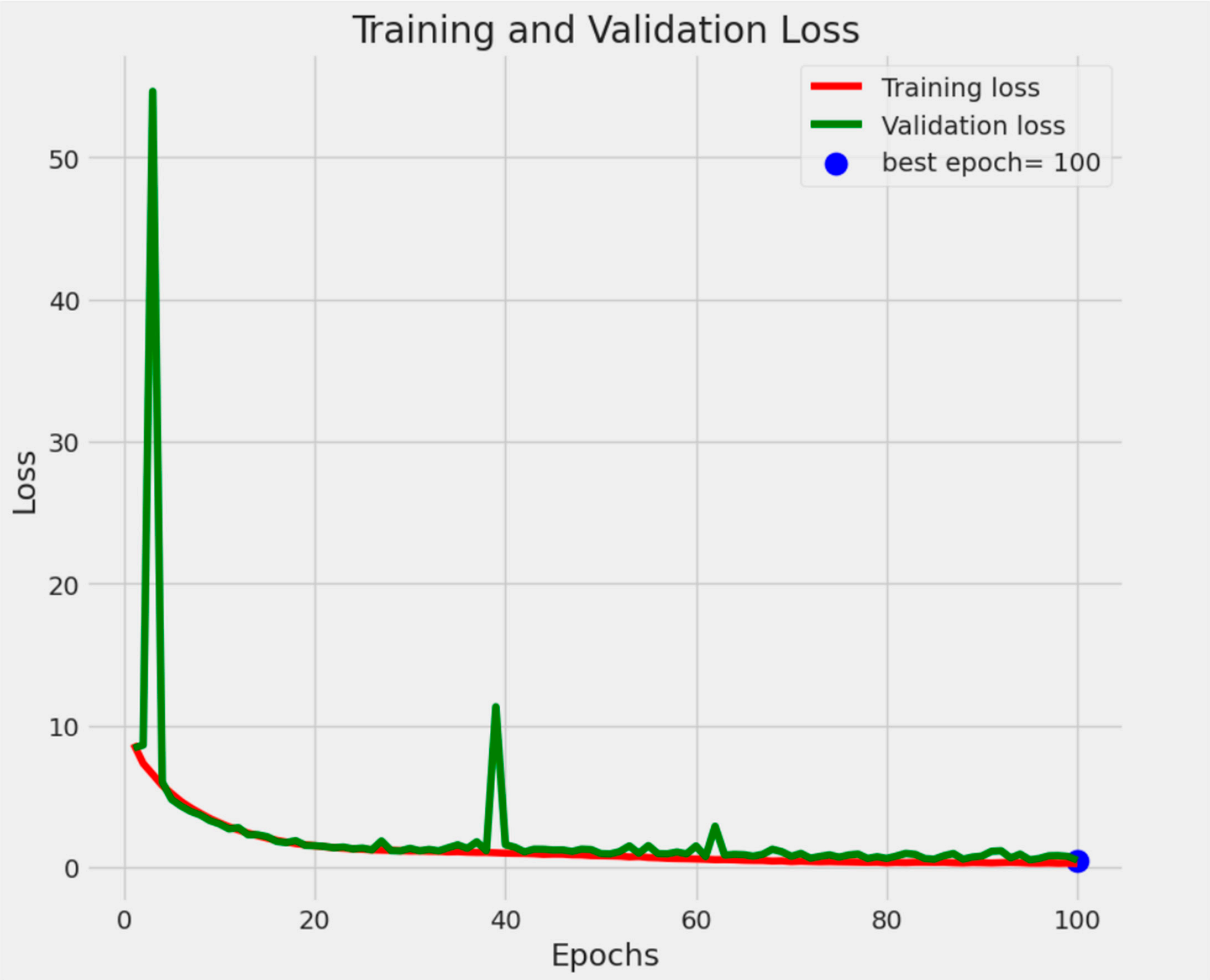
Training and Validation Loss
Training loss
Validation loss
best epoch= 100
Loss
Epochs
0
10
20
30
40
50
0
20
40
60
80
100

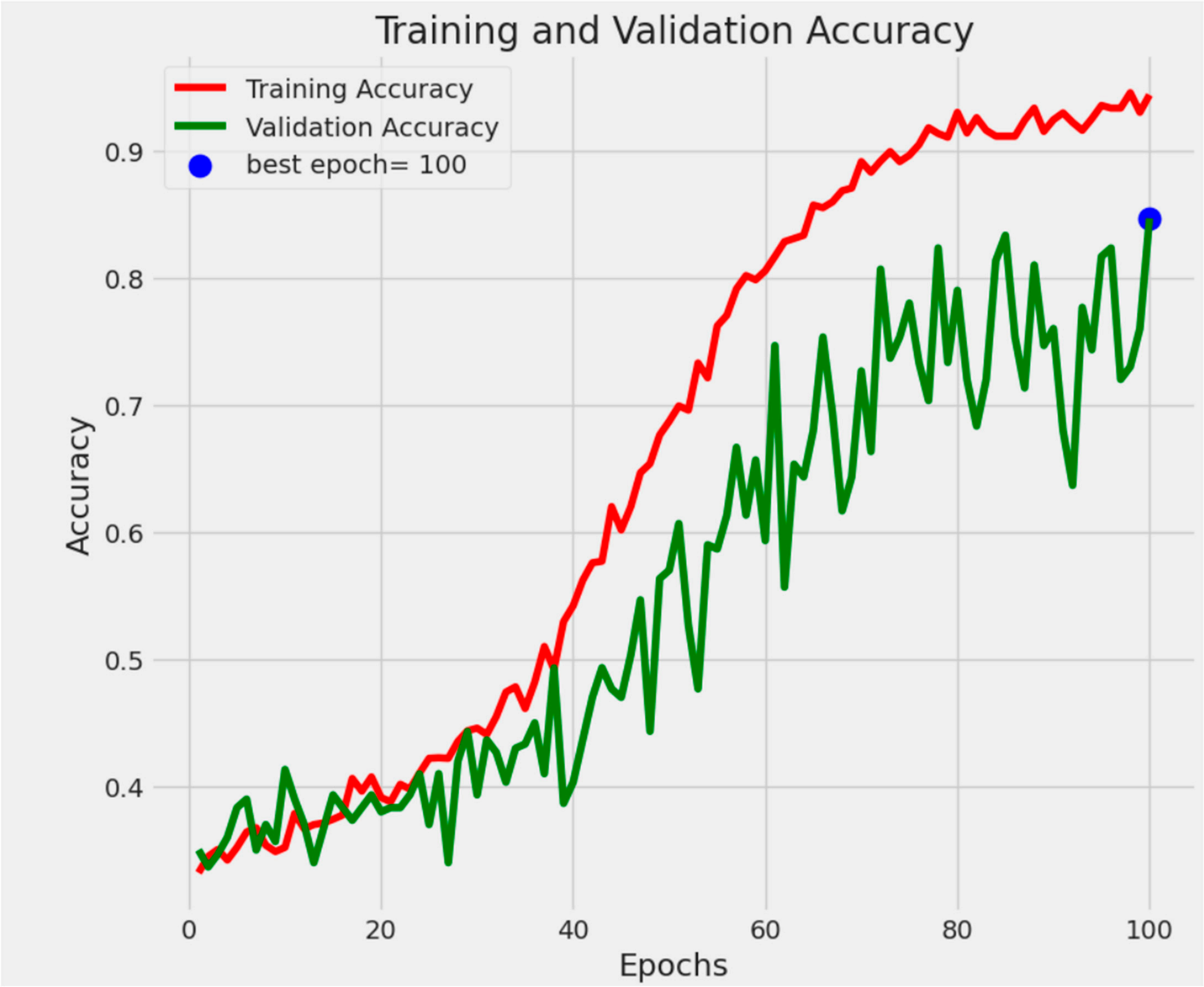

Training and Validation Accuracy
Training Accuracy
Validation Accuracy
best epoch= 100
Accuracy
0.4
0.5
0.6
0.7
0.8
0.9
0
20
40
60
80
100
Epochs

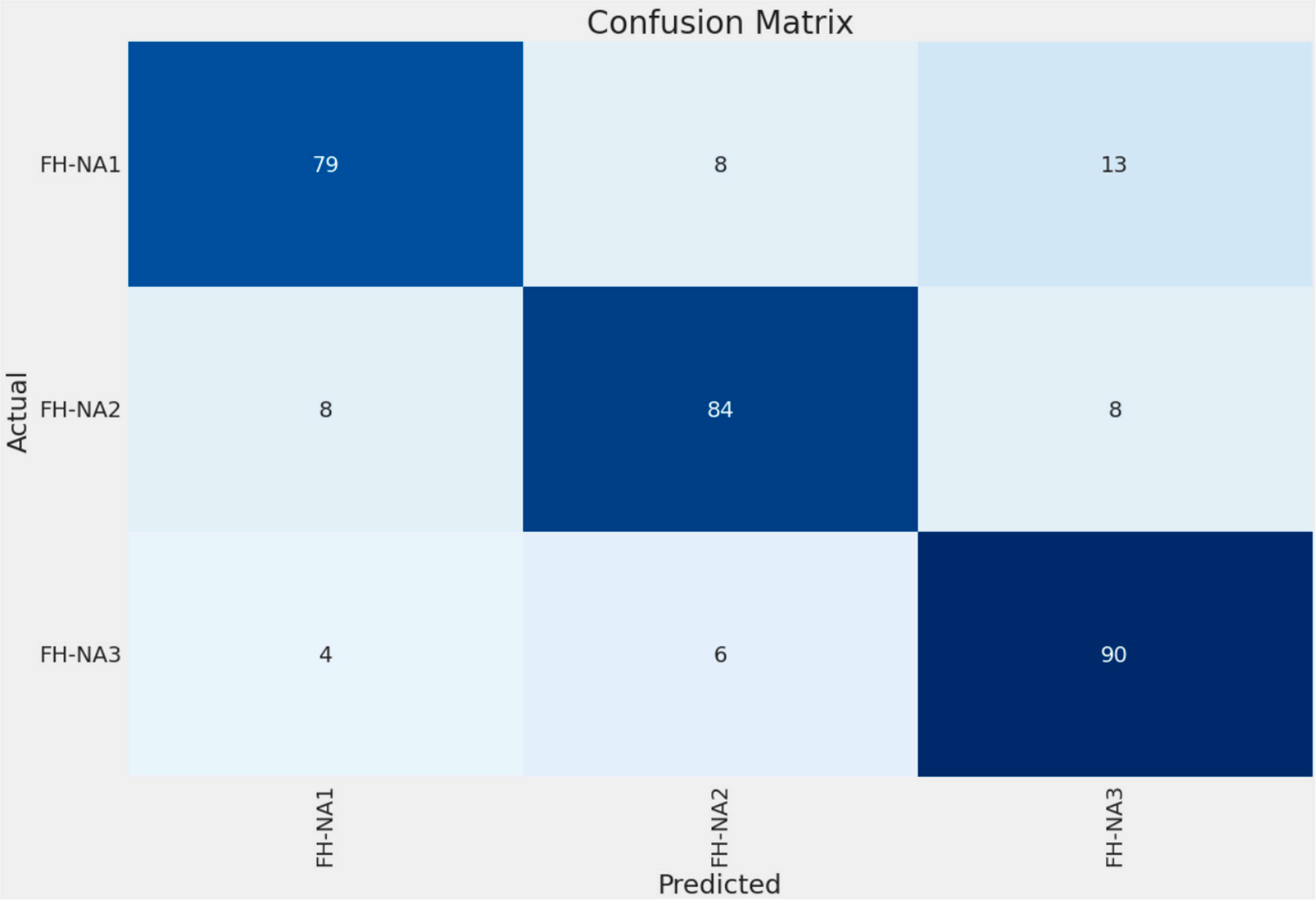

there were 47 errors in 300 tests for an accuracy of 84.33

Classification Report:
----

	precision	recall	f1-score	support
FH-NA1	0.8681	0.7900	0.8272	100
FH-NA2	0.8571	0.8400	0.8485	100
FH-NA3	0.8108	0.9000	0.8531	100
accuracy			0.8433	300
macro avg	0.8454	0.8433	0.8429	300
weighted avg	0.8454	0.8433	0.8429	300

EFFICIENTNETV2 B0

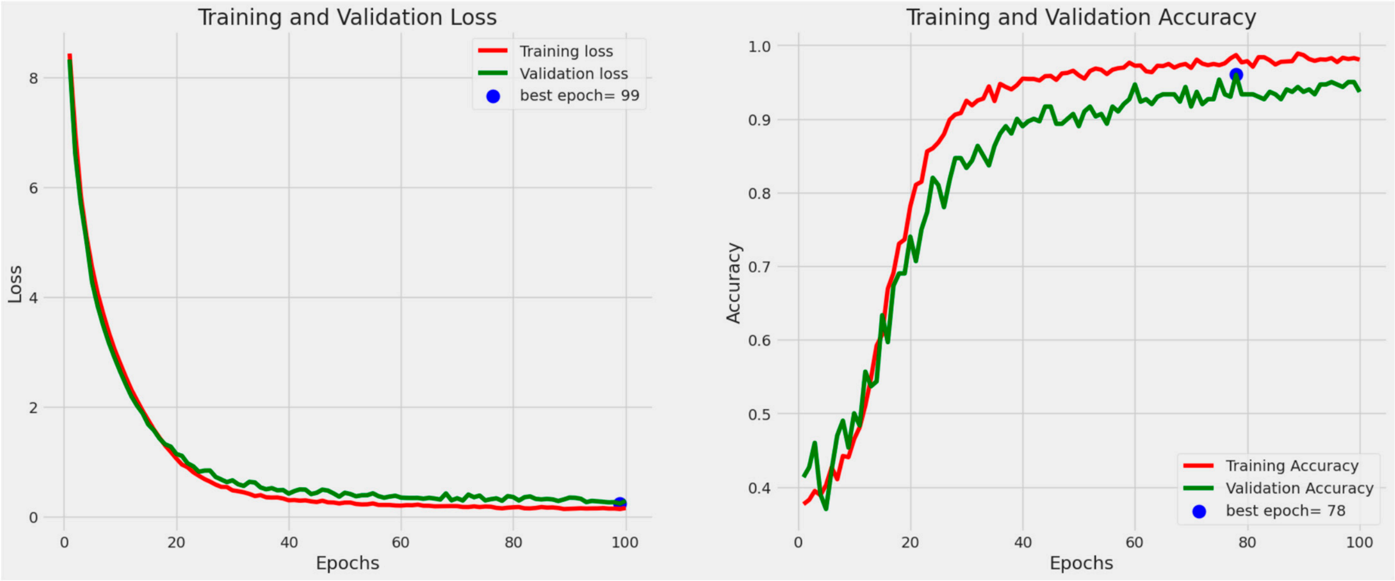

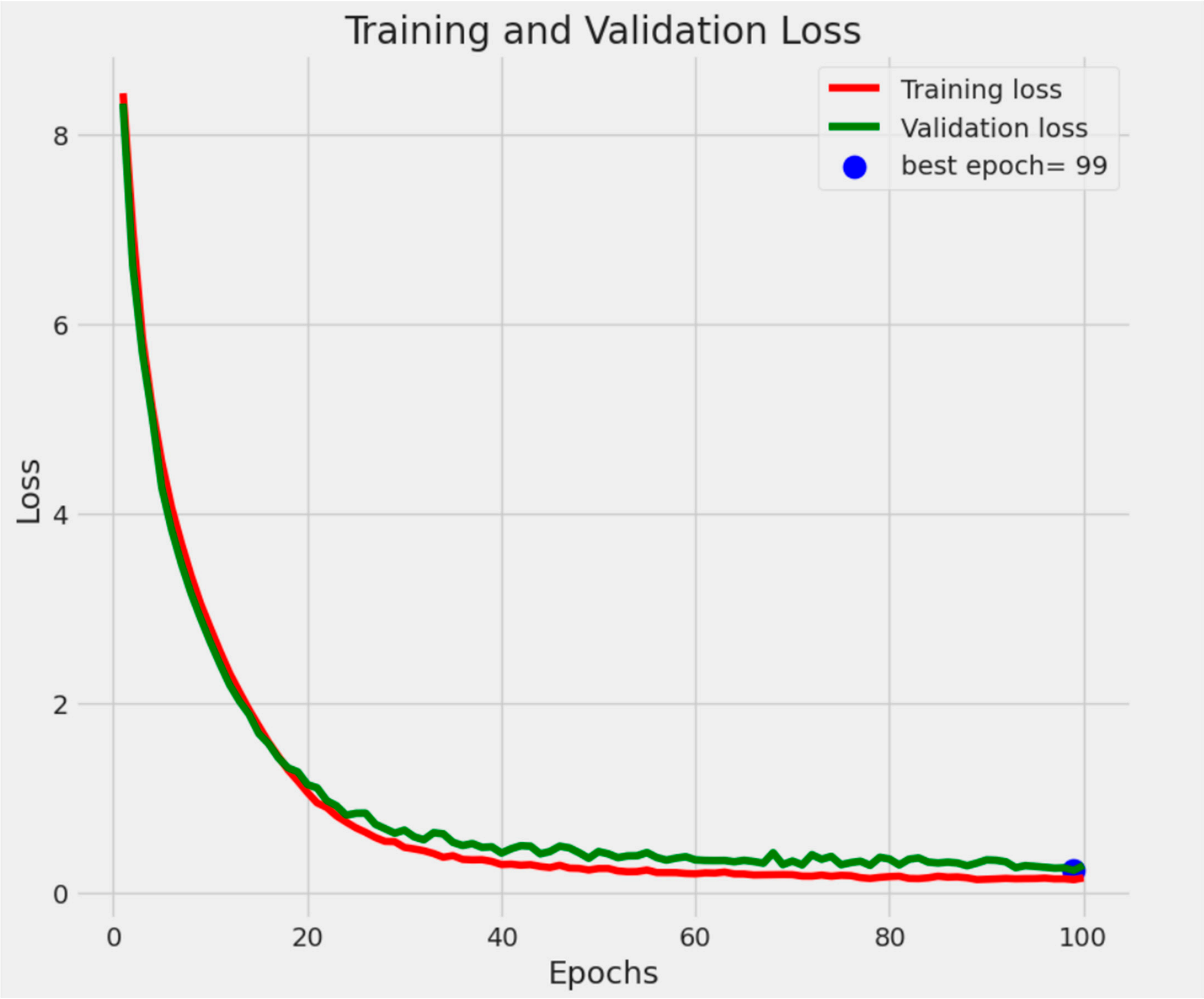

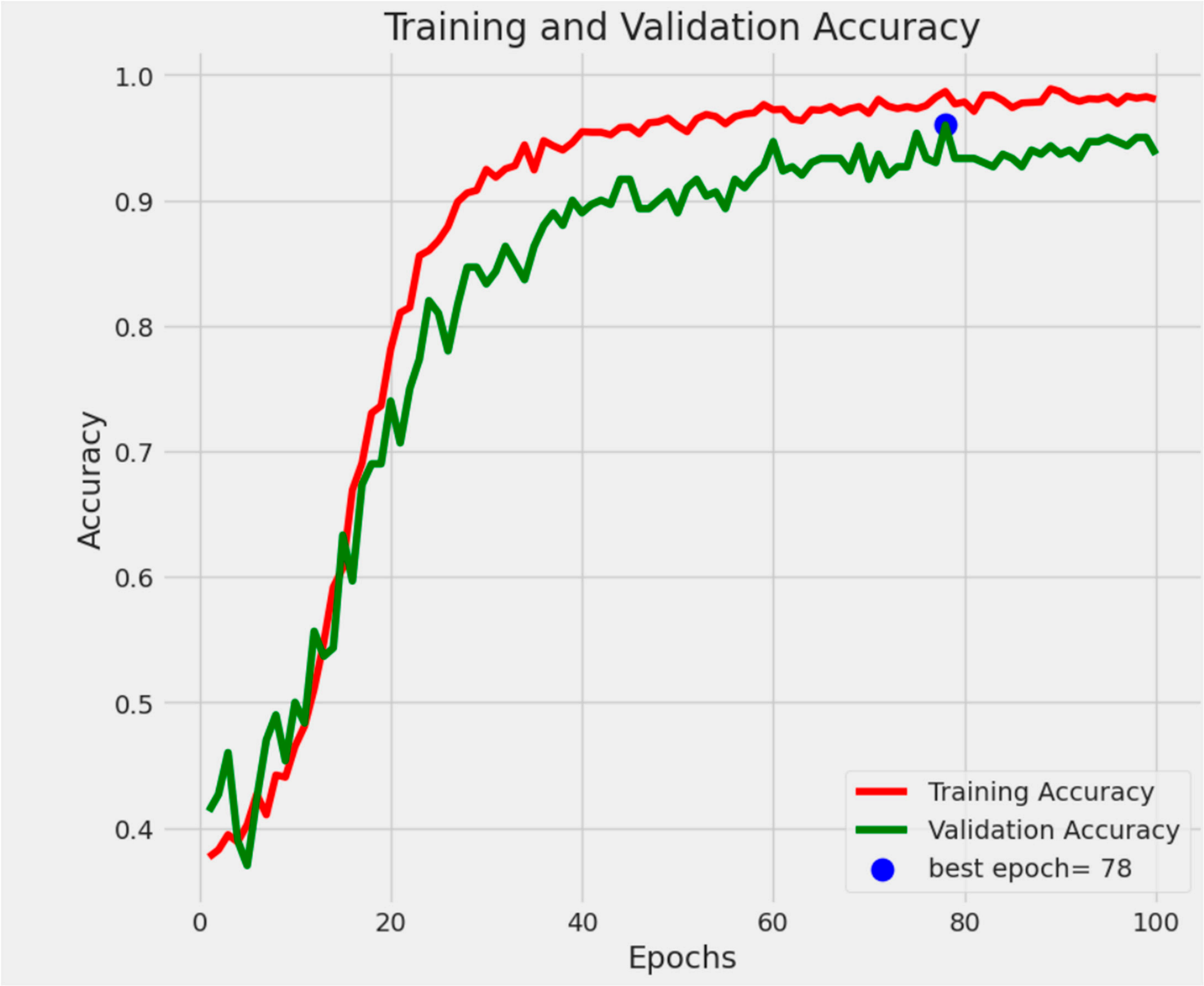

Training and Validation Accuracy
Accuracy
Epochs
1.0
0.9
0.8
0.7
0.6
0.5
0.4
0
20
40
60
80
100
Training Accuracy
Validation Accuracy
best epoch= 78

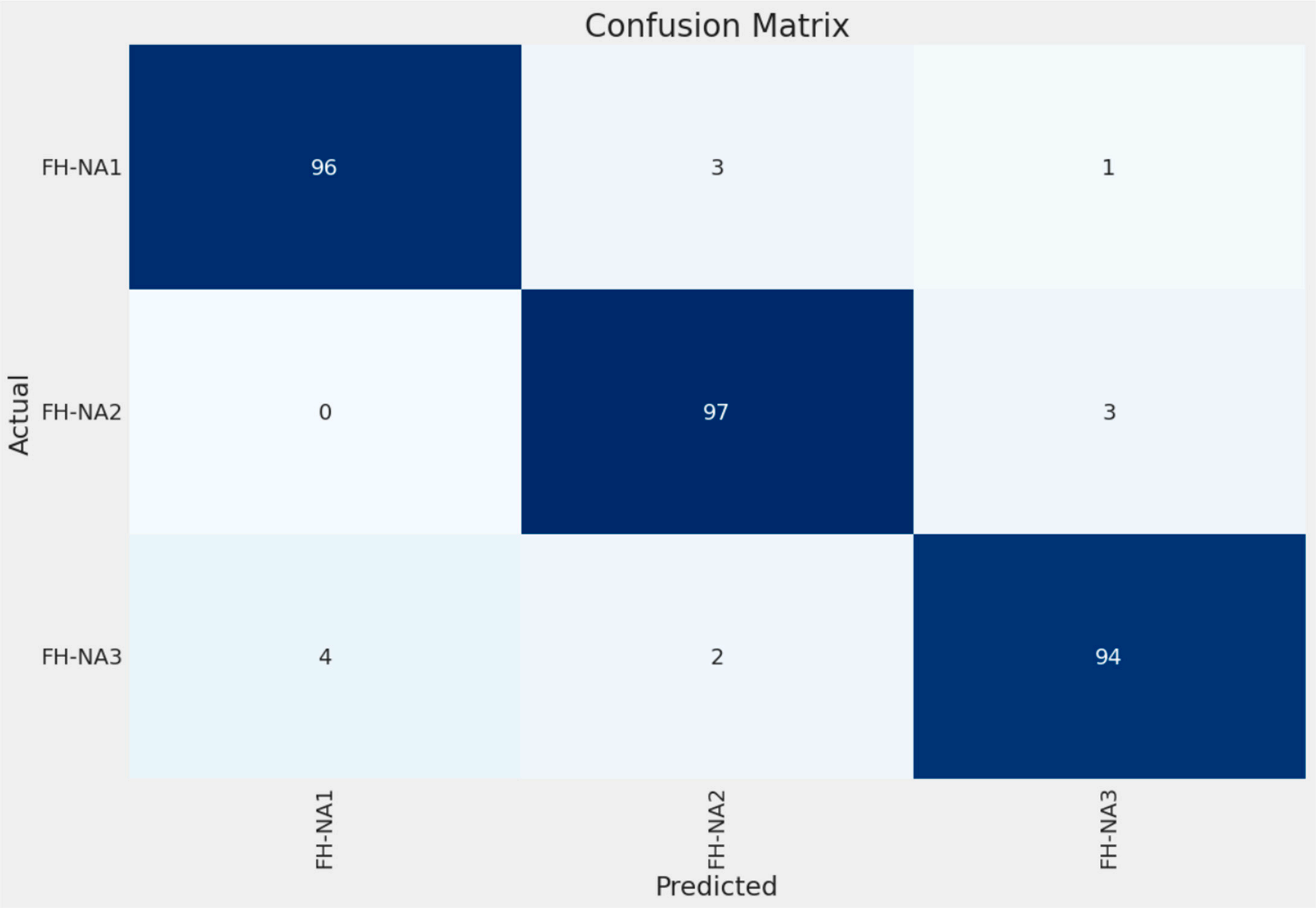

there were 13 errors in 300 tests for an accuracy of 95.67

Classification Report:
----

	precision	recall	f1-score	support
FH-NA1	0.9600	0.9600	0.9600	100
FH-NA2	0.9510	0.9700	0.9604	100
FH-NA3	0.9592	0.9400	0.9495	100
accuracy			0.9567	300
macro avg	0.9567	0.9567	0.9566	300
weighted avg	0.9567	0.9567	0.9566	300
