# Supplementary material for: Profile Photograph Classification Performance of Deep Learning Algorithms Trained Using Cephalometric Measurements: A Preliminary Study
Source: Diagnostics (Basel). 2024 Aug 30;14(17):1916. doi: 10.3390/diagnostics14171916 (PMC11394270; doi:10.3390/diagnostics14171916)
Supplement: Supplementary file 1 [file diagnostics-14-01916-s001.zip › FHNPG.pdf]

MOBILENET V2

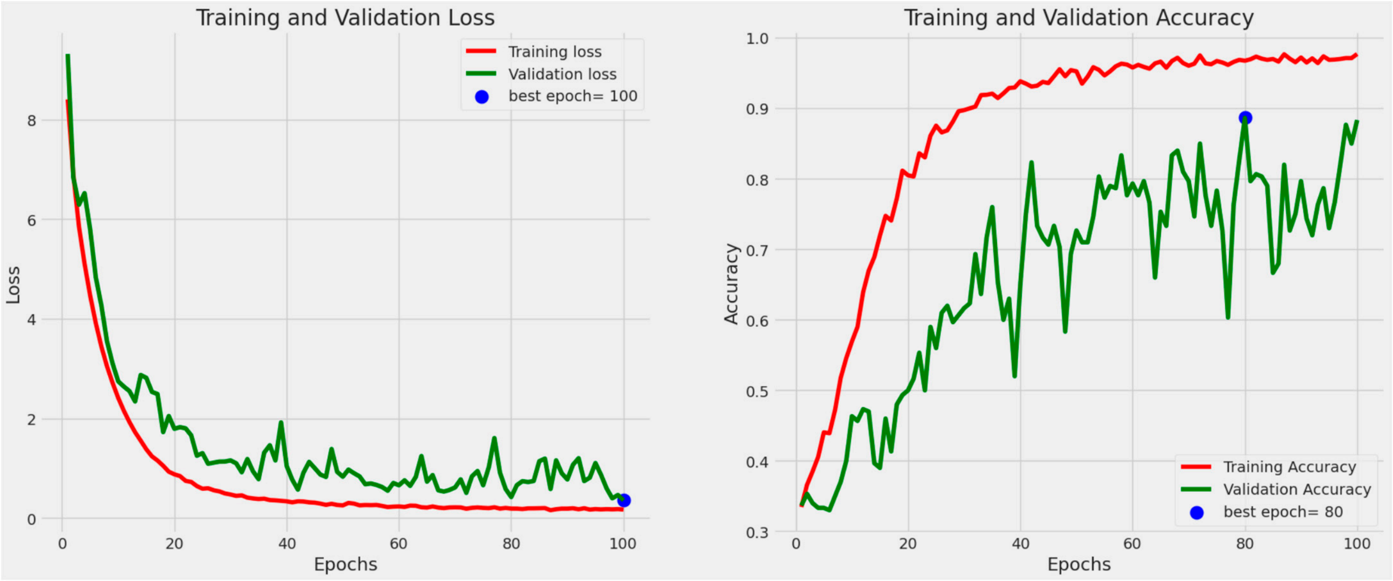

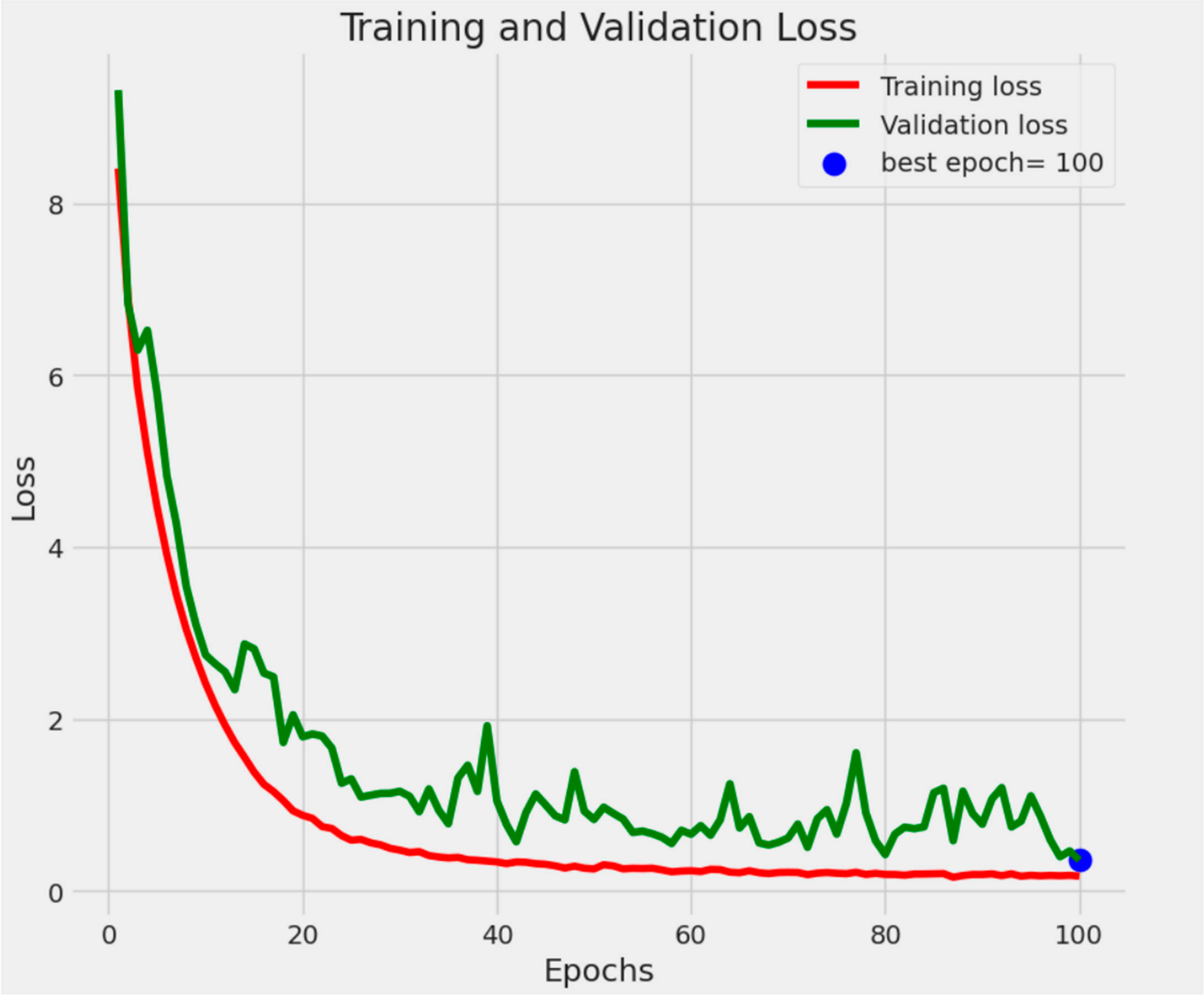

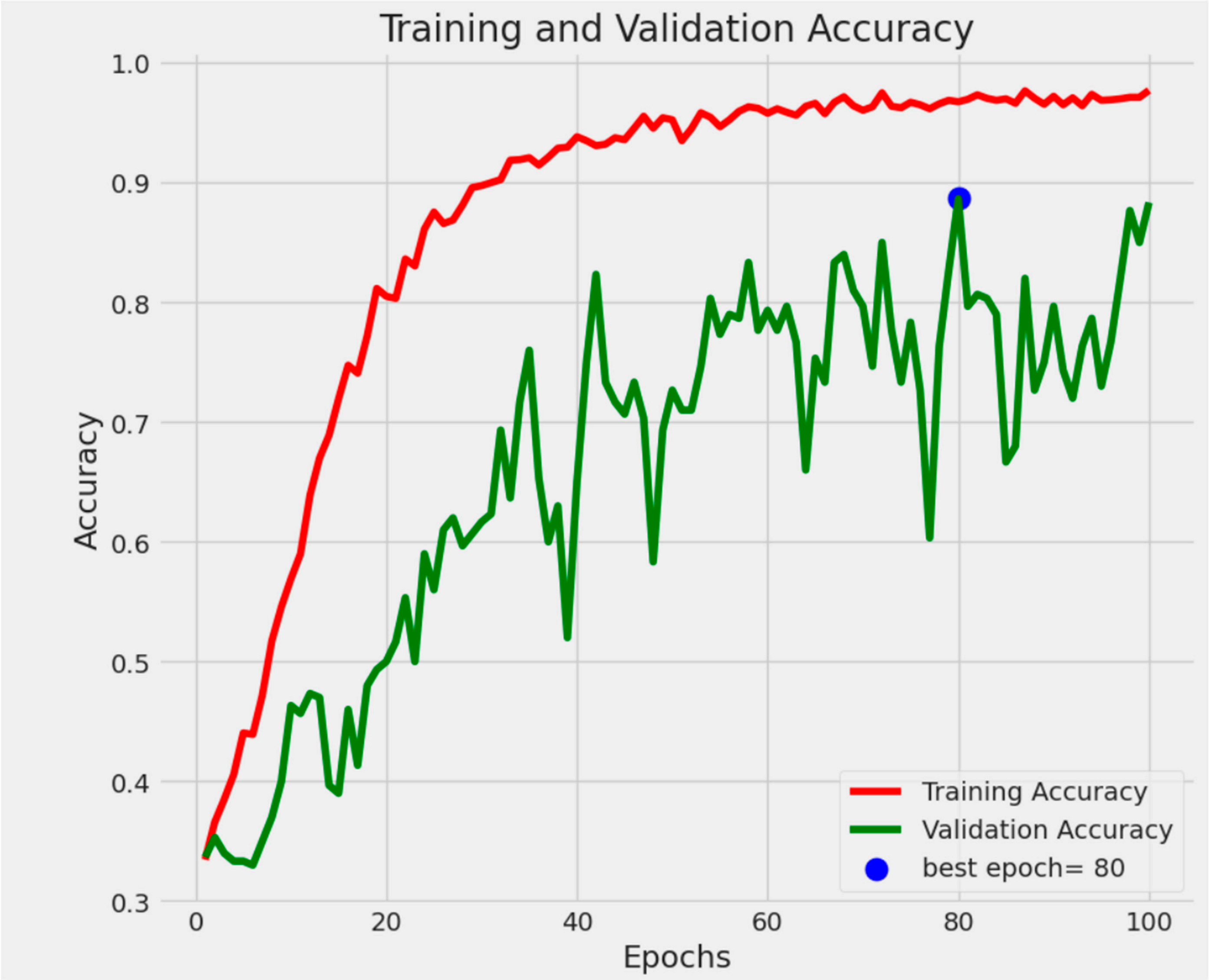

Training and Validation Accuracy
Accuracy
Epochs
1.0
0.9
0.8
0.7
0.6
0.5
0.4
0.3
0
20
40
60
80
100
Training Accuracy
Validation Accuracy
best epoch= 80

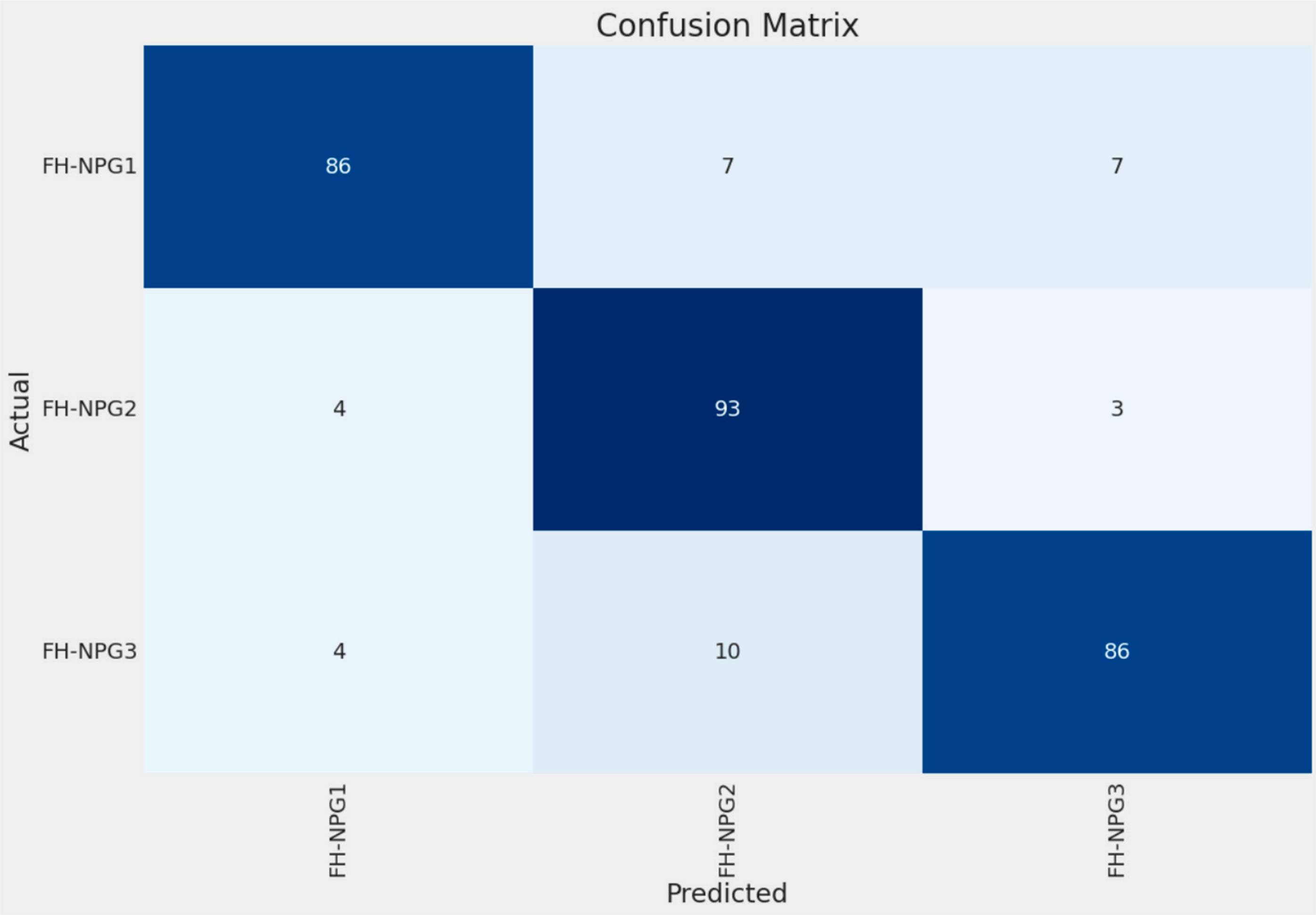

there were 35 errors in 300 tests for an accuracy of 88.33

Classification Report:
----

	precision	recall	f1-score	support
FH-NPG1	0.9149	0.8600	0.8866	100
FH-NPG2	0.8455	0.9300	0.8857	100
FH-NPG3	0.8958	0.8600	0.8776	100
accuracy			0.8833	300
macro avg	0.8854	0.8833	0.8833	300
weighted avg	0.8854	0.8833	0.8833	300

INCEPTION V3: %31.33

DENSENET121

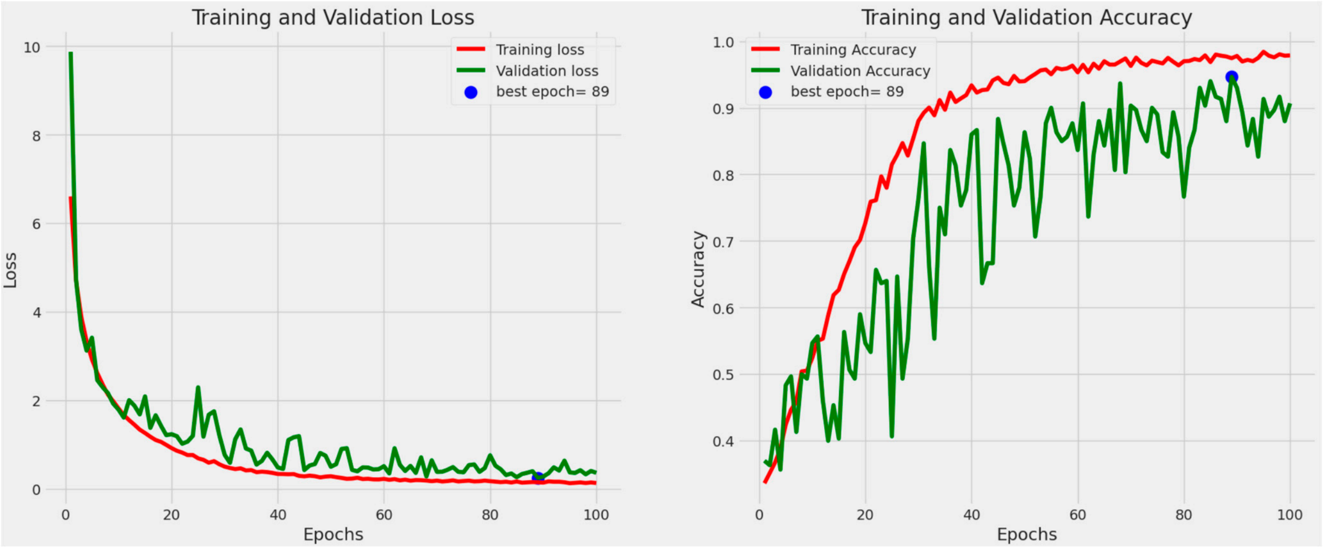

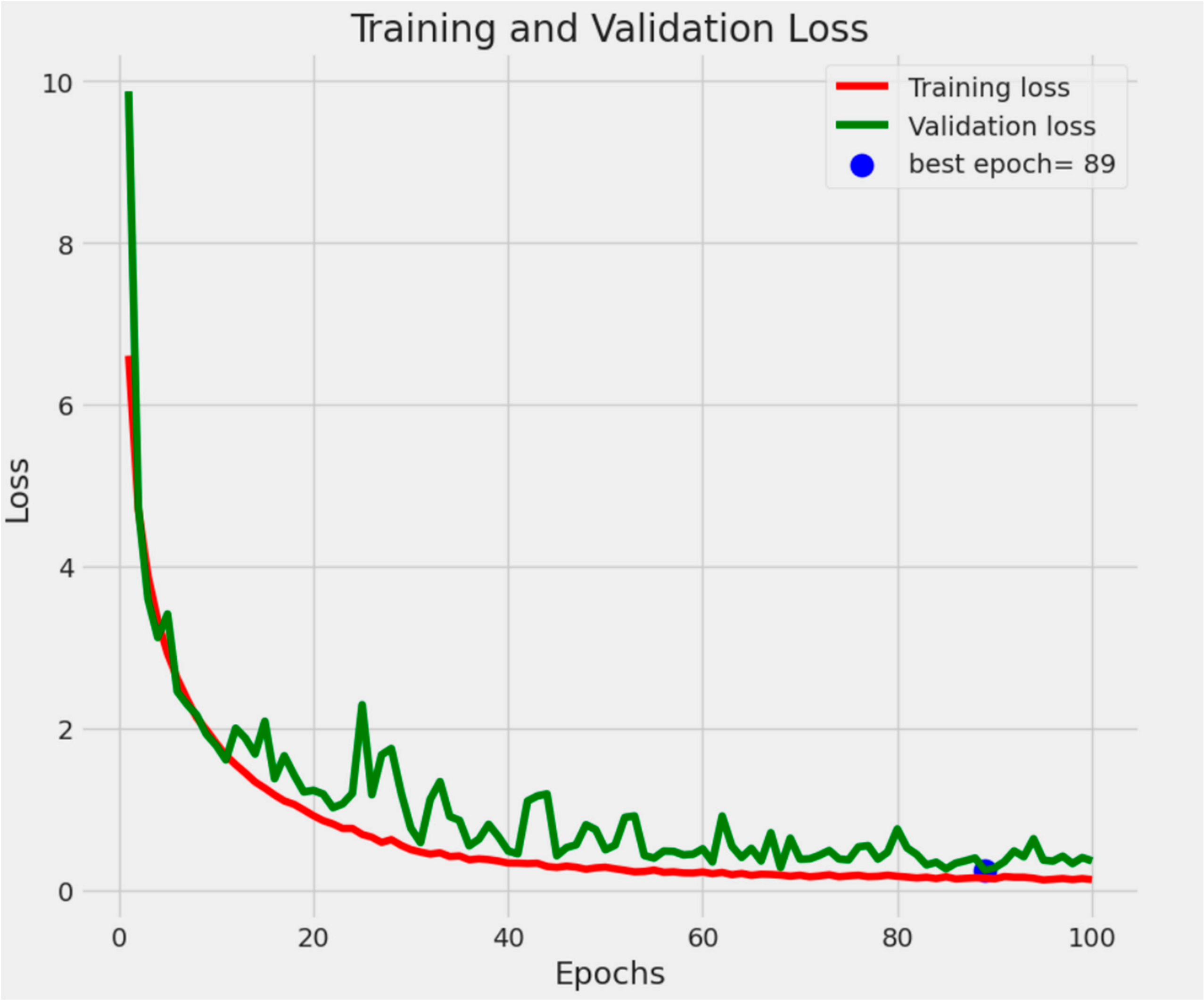
Training and Validation Loss
Training loss
Validation loss
best epoch= 89
Loss
Epochs
0
2
4
6
8
10
0
20
40
60
80
100

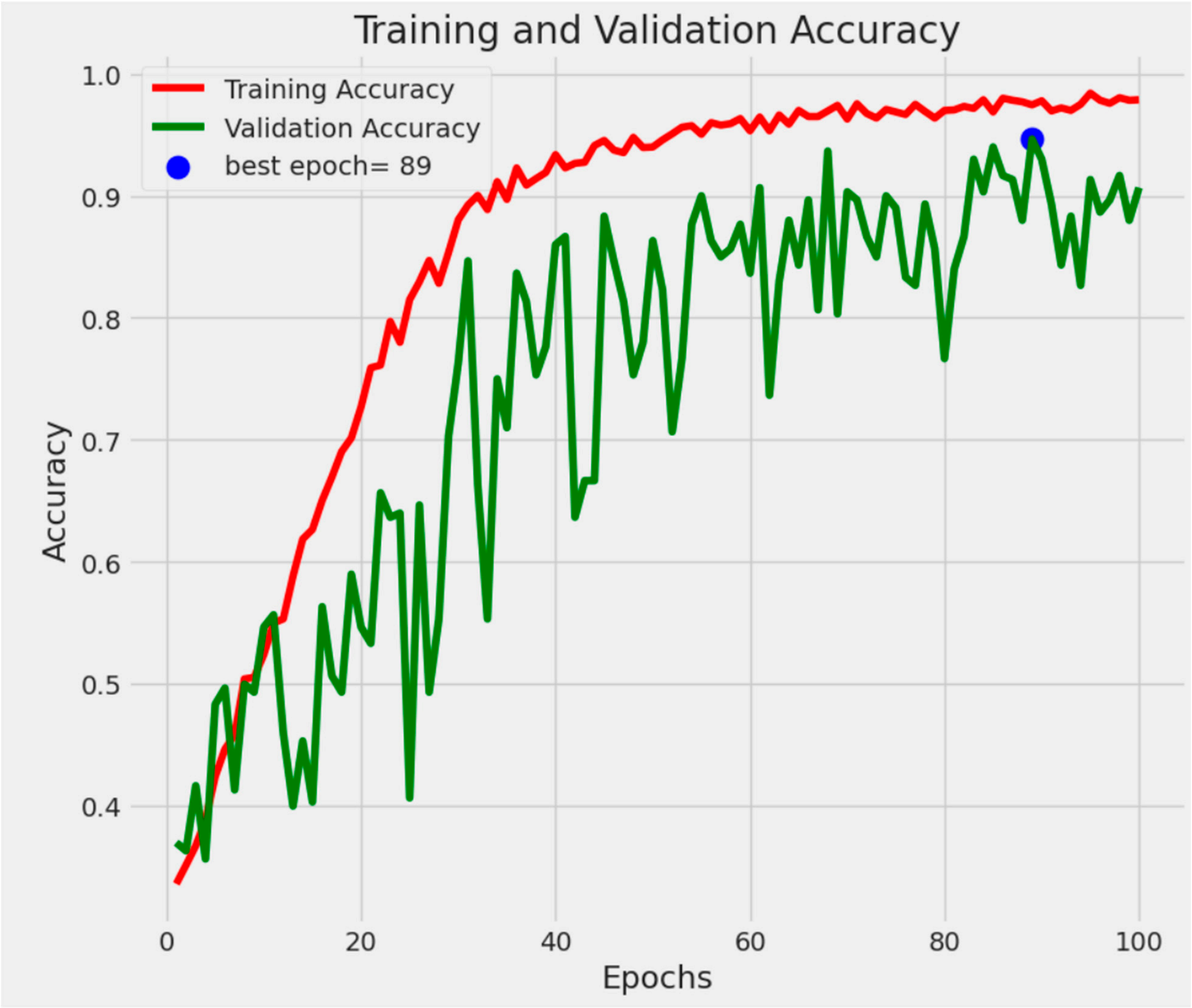
Training and Validation Accuracy
Training Accuracy
Validation Accuracy
best epoch= 89
Accuracy
Epochs
1.0
0.9
0.8
0.7
0.6
0.5
0.4
0
20
40
60
80
100

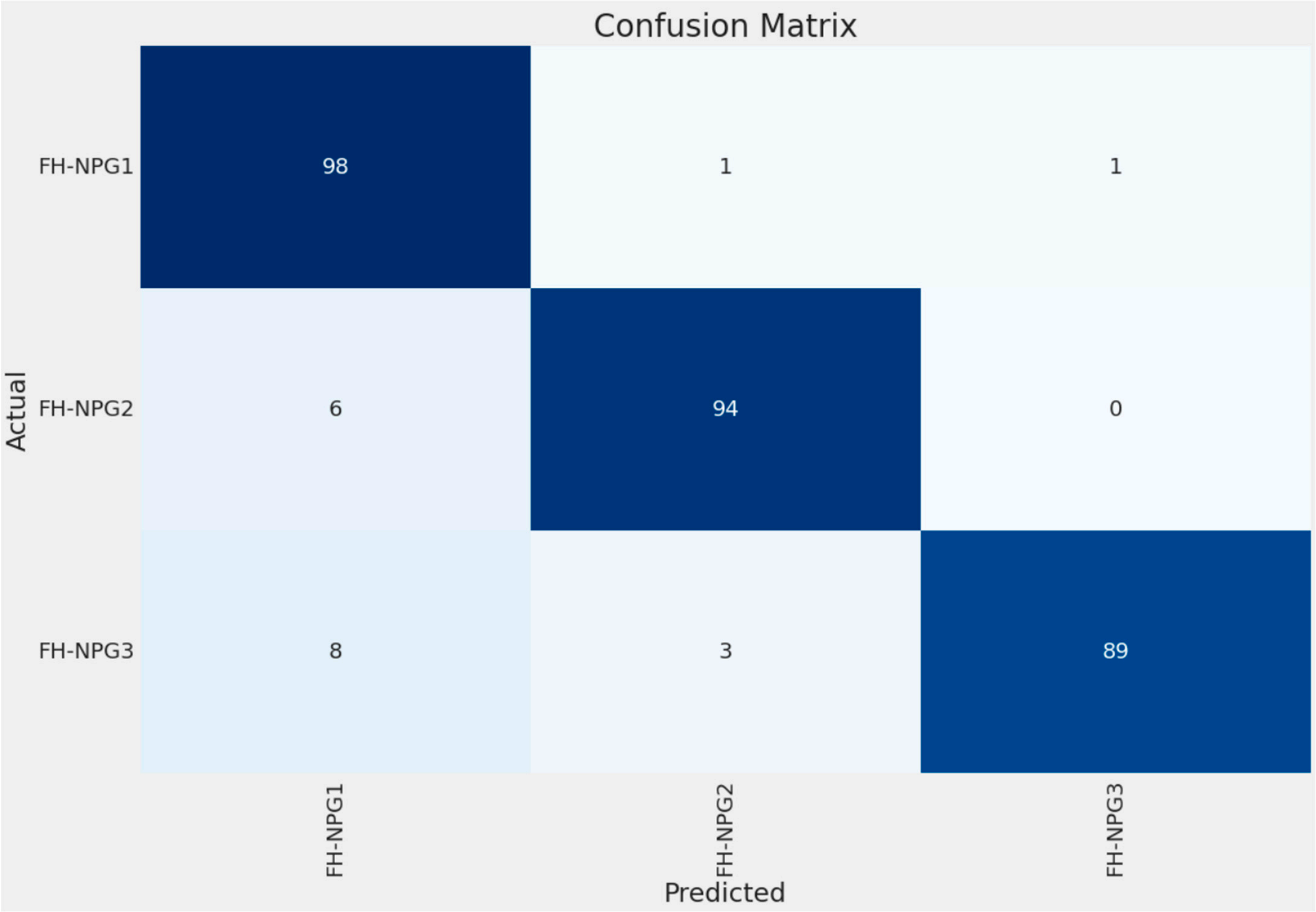

there were 19 errors in 300 tests for an accuracy of 93.67

Classification Report:
----

	precision	recall	f1-score	support
FH-NPG1	0.8750	0.9800	0.9245	100
FH-NPG2	0.9592	0.9400	0.9495	100
FH-NPG3	0.9889	0.8900	0.9368	100
accuracy			0.9367	300
macro avg	0.9410	0.9367	0.9370	300
weighted avg	0.9410	0.9367	0.9370	300

DENSENET 169

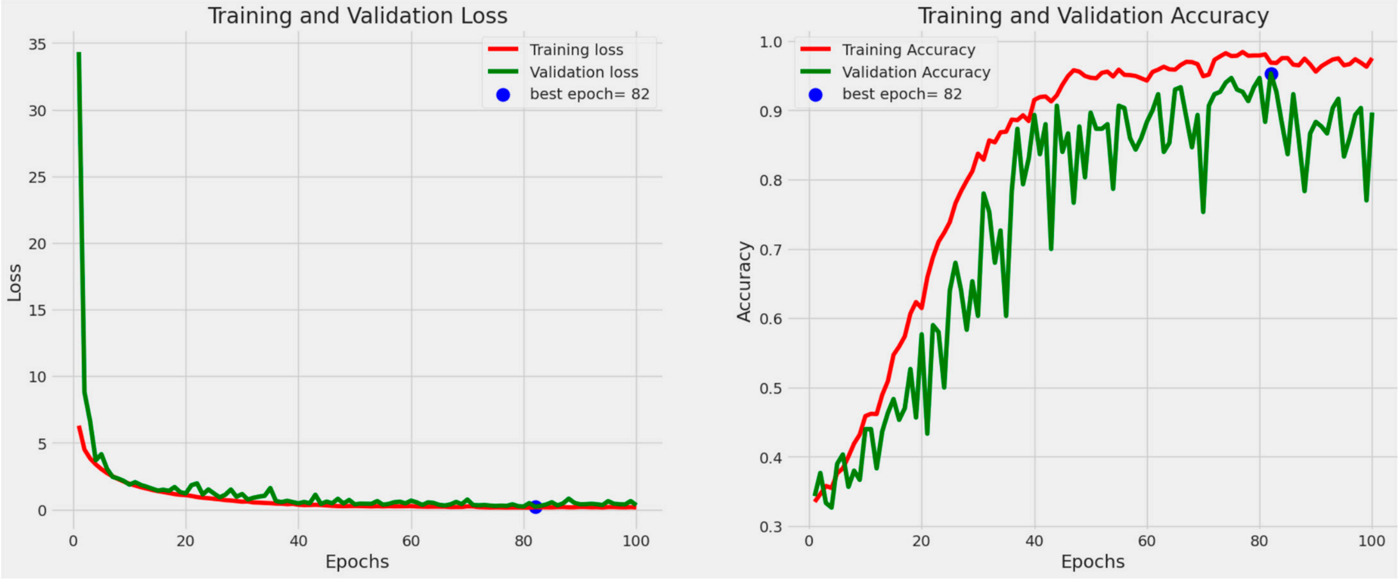

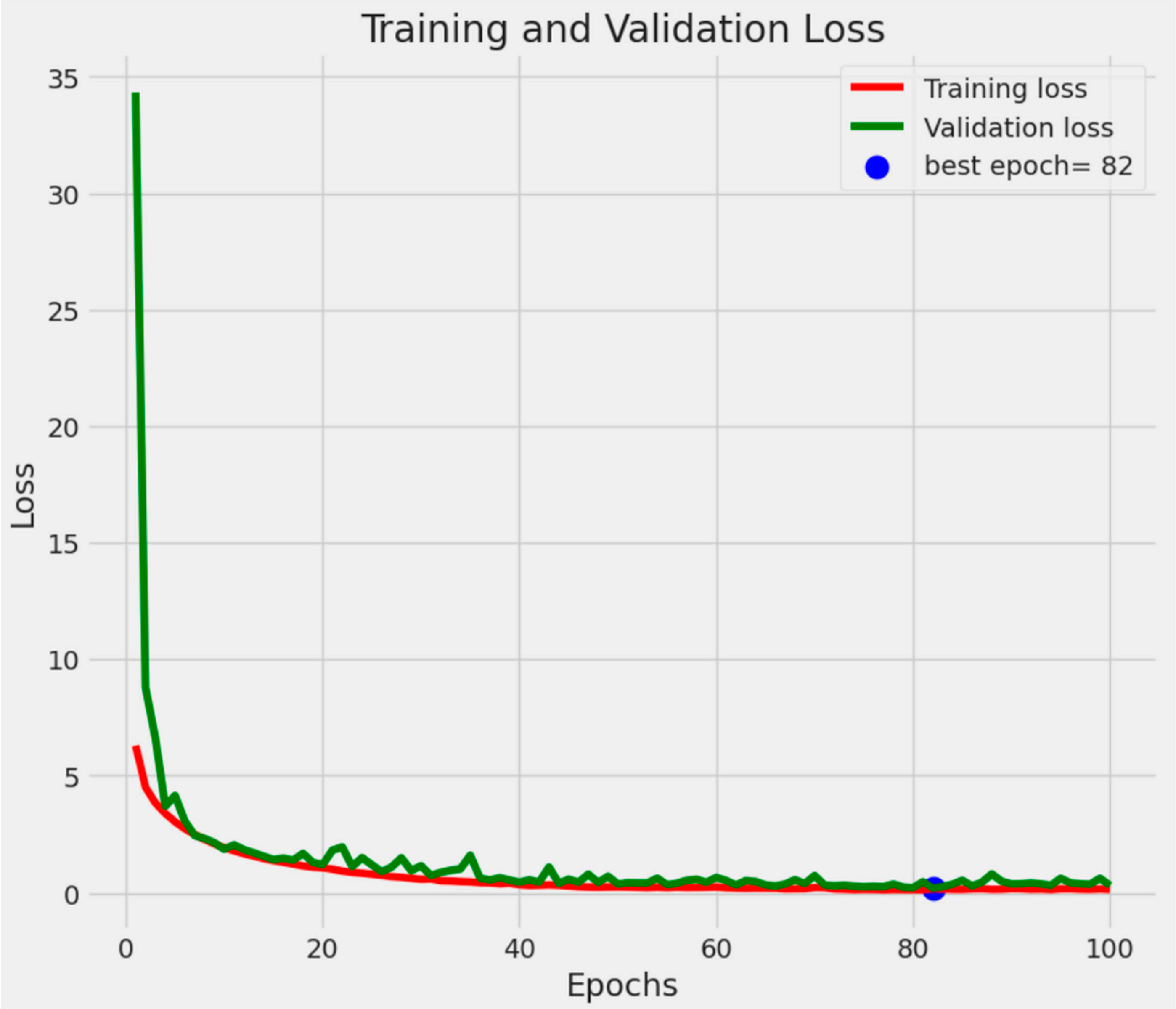

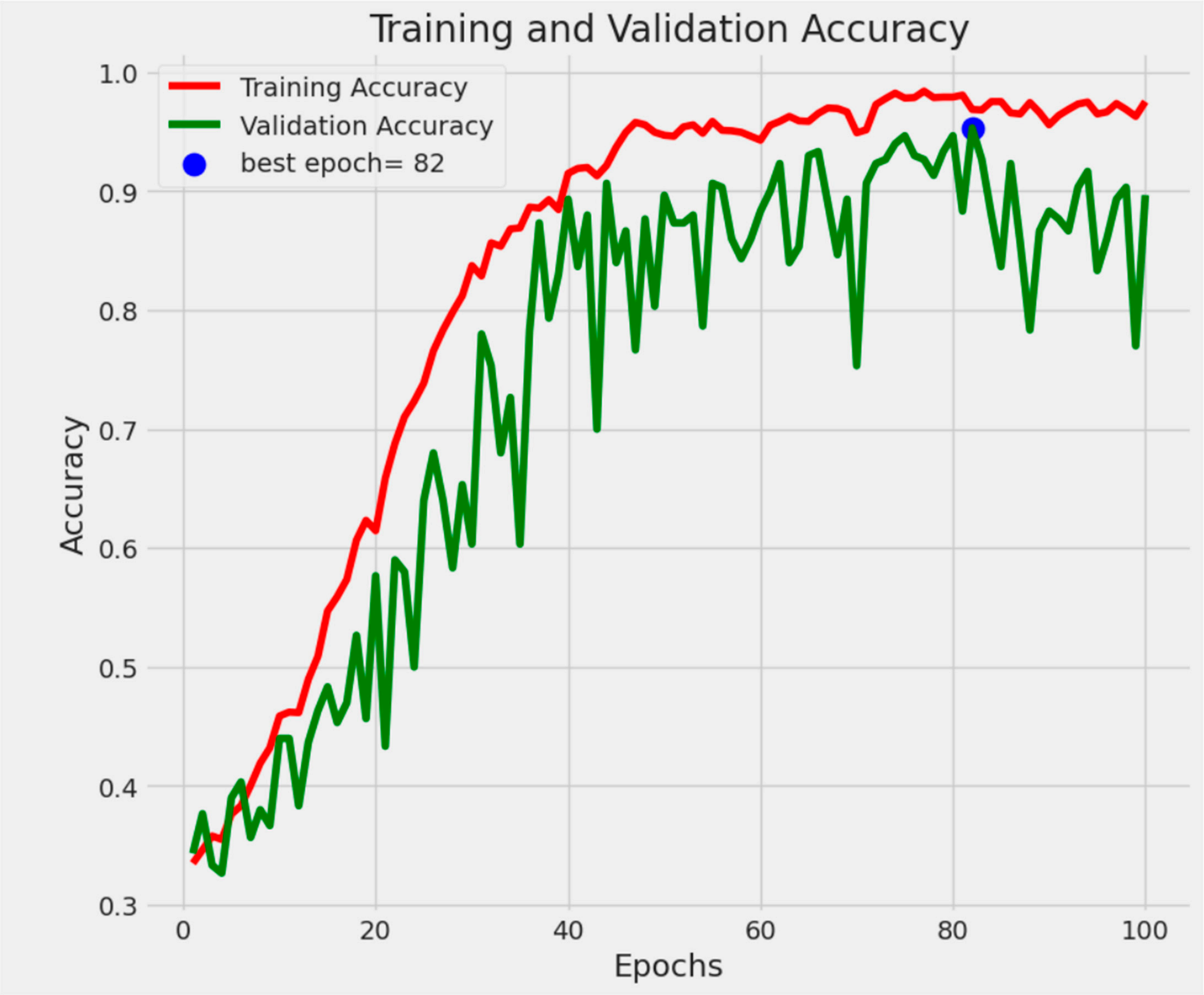
Training and Validation Accuracy
Training Accuracy
Validation Accuracy
best epoch= 82
Accuracy
Epochs
1.0
0.9
0.8
0.7
0.6
0.5
0.4
0.3
0
20
40
60
80
100

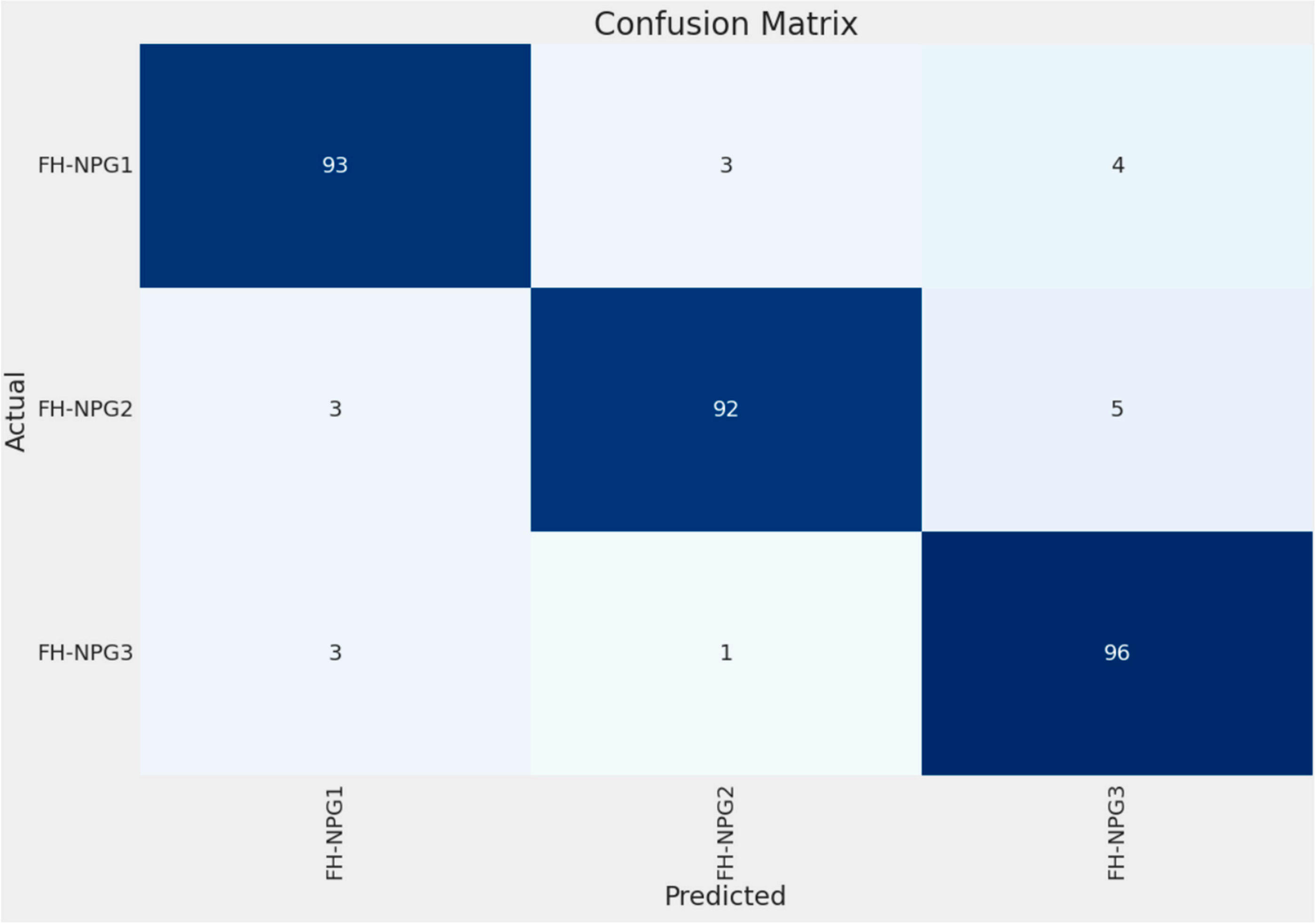

there were 19 errors in 300 tests for an accuracy of 93.67

Classification Report:
----

	precision	recall	f1-score	support
FH-NPG1	0.9394	0.9300	0.9347	100
FH-NPG2	0.9583	0.9200	0.9388	100
FH-NPG3	0.9143	0.9600	0.9366	100
accuracy			0.9367	300
macro avg	0.9373	0.9367	0.9367	300
weighted avg	0.9373	0.9367	0.9367	300

DENSENET 201

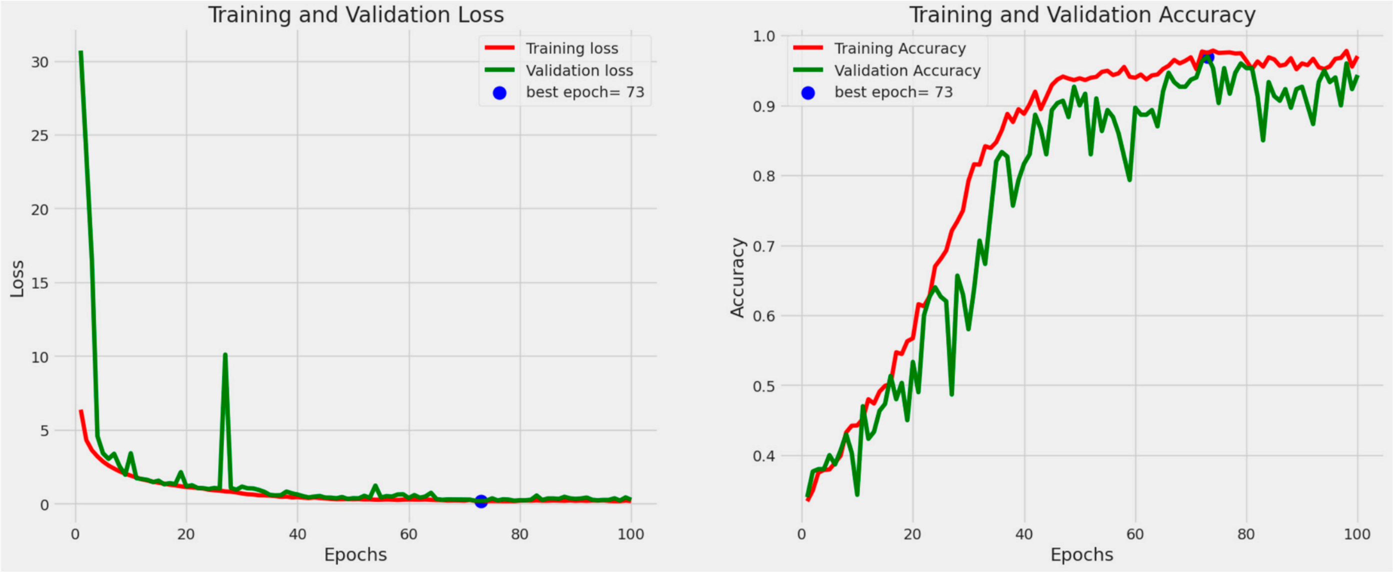

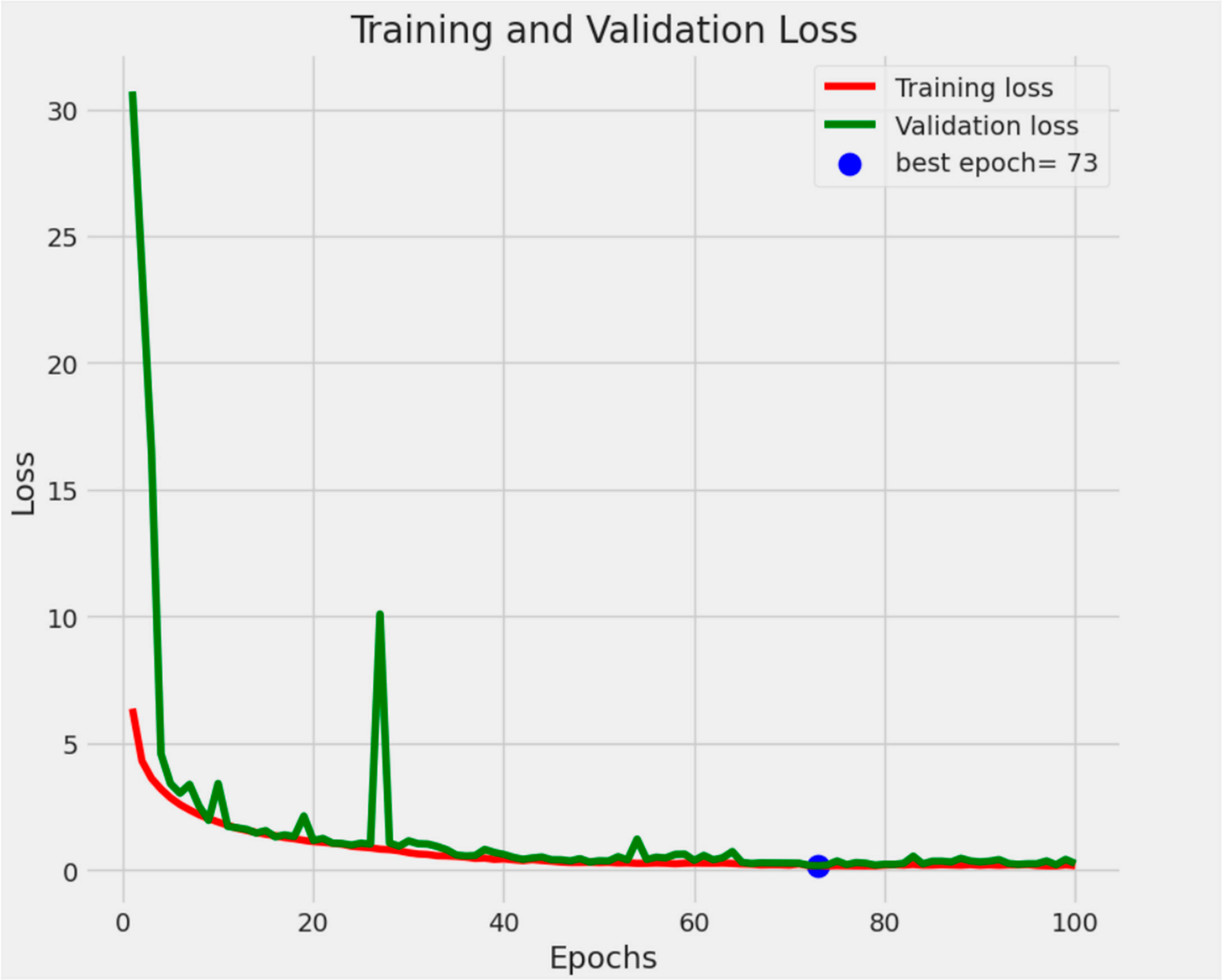
Training and Validation Loss
Training loss
Validation loss
best epoch= 73
Loss
Epochs
0
5
10
15
20
25
30
0
20
40
60
80
100

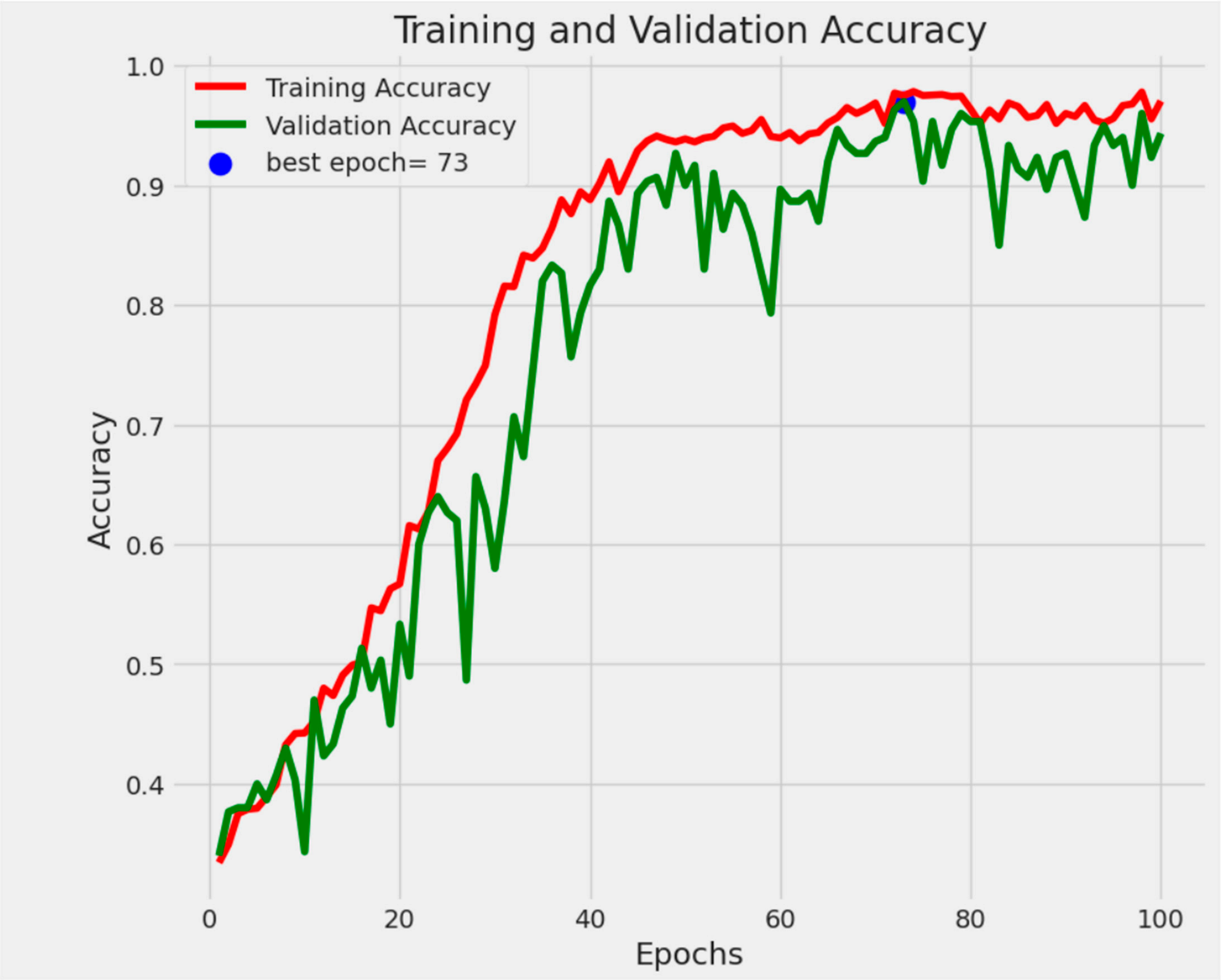

Training and Validation Accuracy
Training Accuracy
Validation Accuracy
best epoch= 73
Accuracy
Epochs
1.0
0.9
0.8
0.7
0.6
0.5
0.4
0
20
40
60
80
100

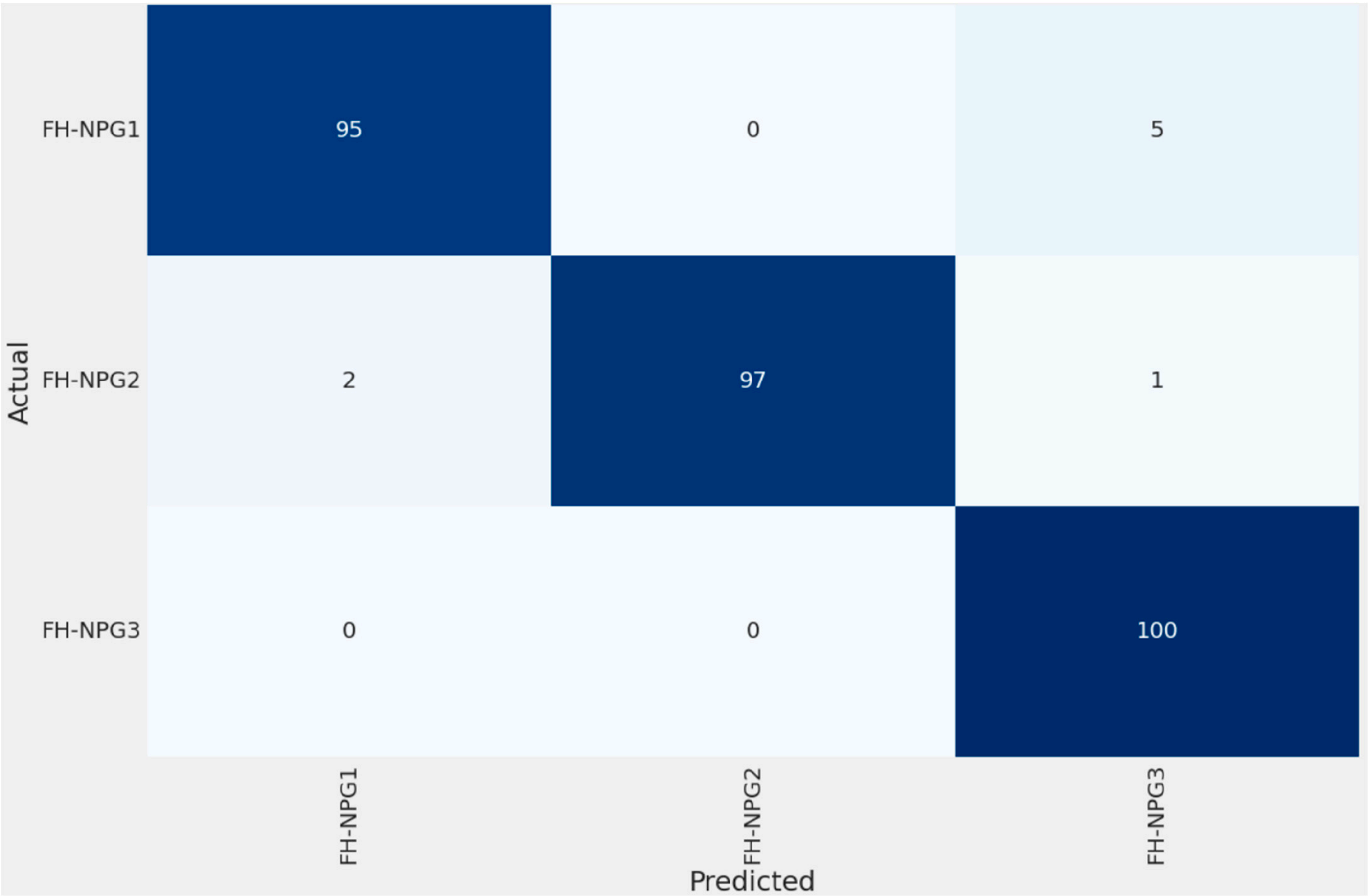

there were 8 errors in 300 tests for an accuracy of 97.33

Classification Report:
----

	precision	recall	f1-score	support
FH-NPG1	0.9794	0.9500	0.9645	100
FH-NPG2	1.0000	0.9700	0.9848	100
FH-NPG3	0.9434	1.0000	0.9709	100
accuracy			0.9733	300
macro avg	0.9743	0.9733	0.9734	300
weighted avg	0.9743	0.9733	0.9734	300

EFFICIENTNET B0

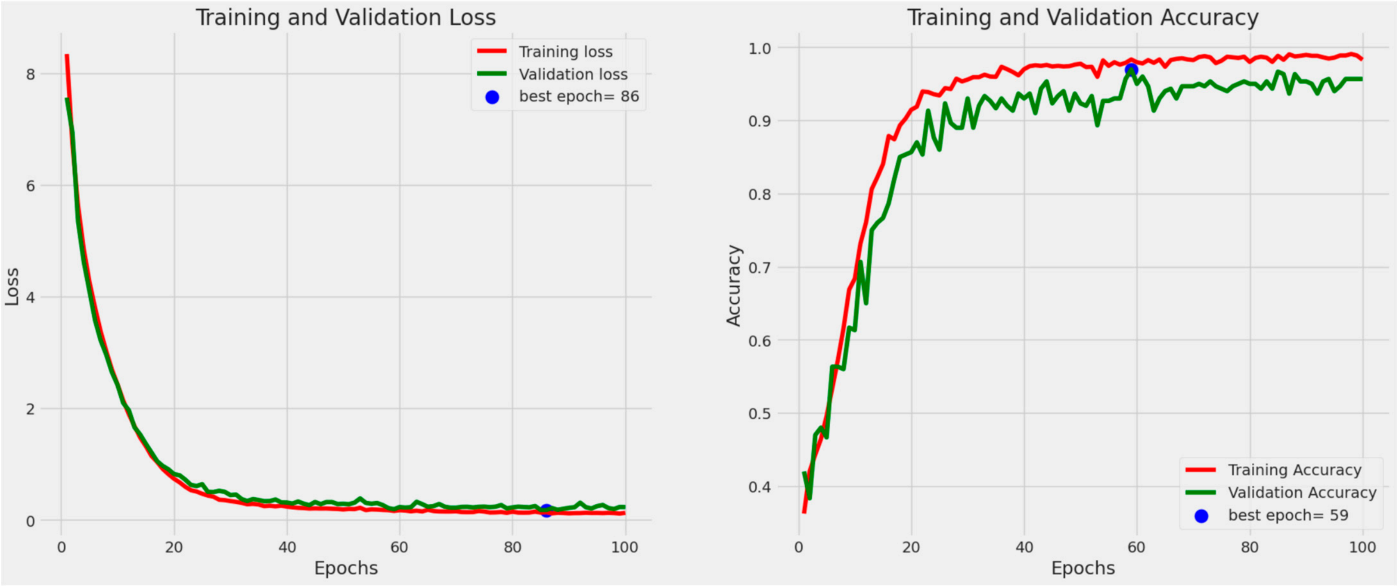

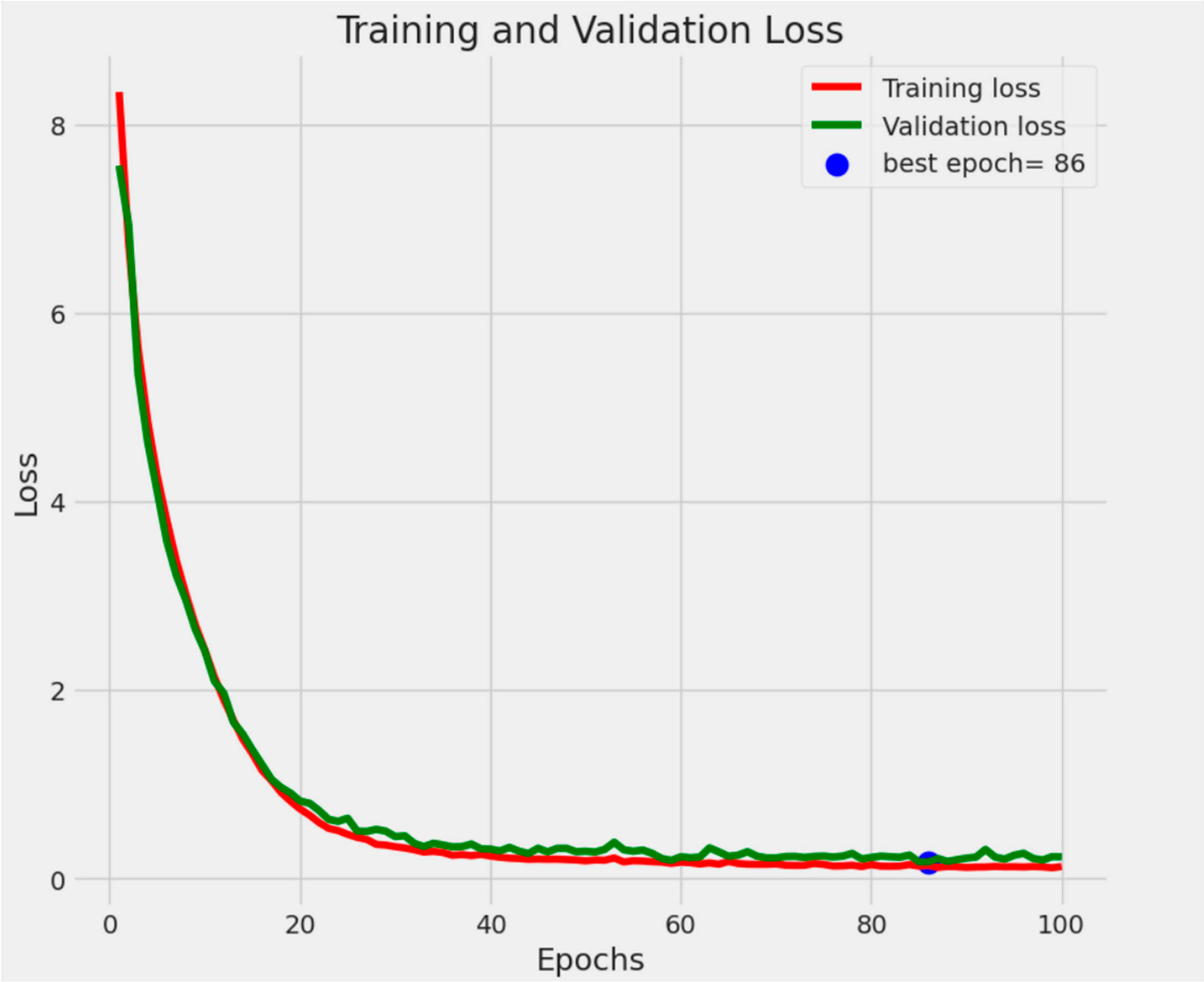

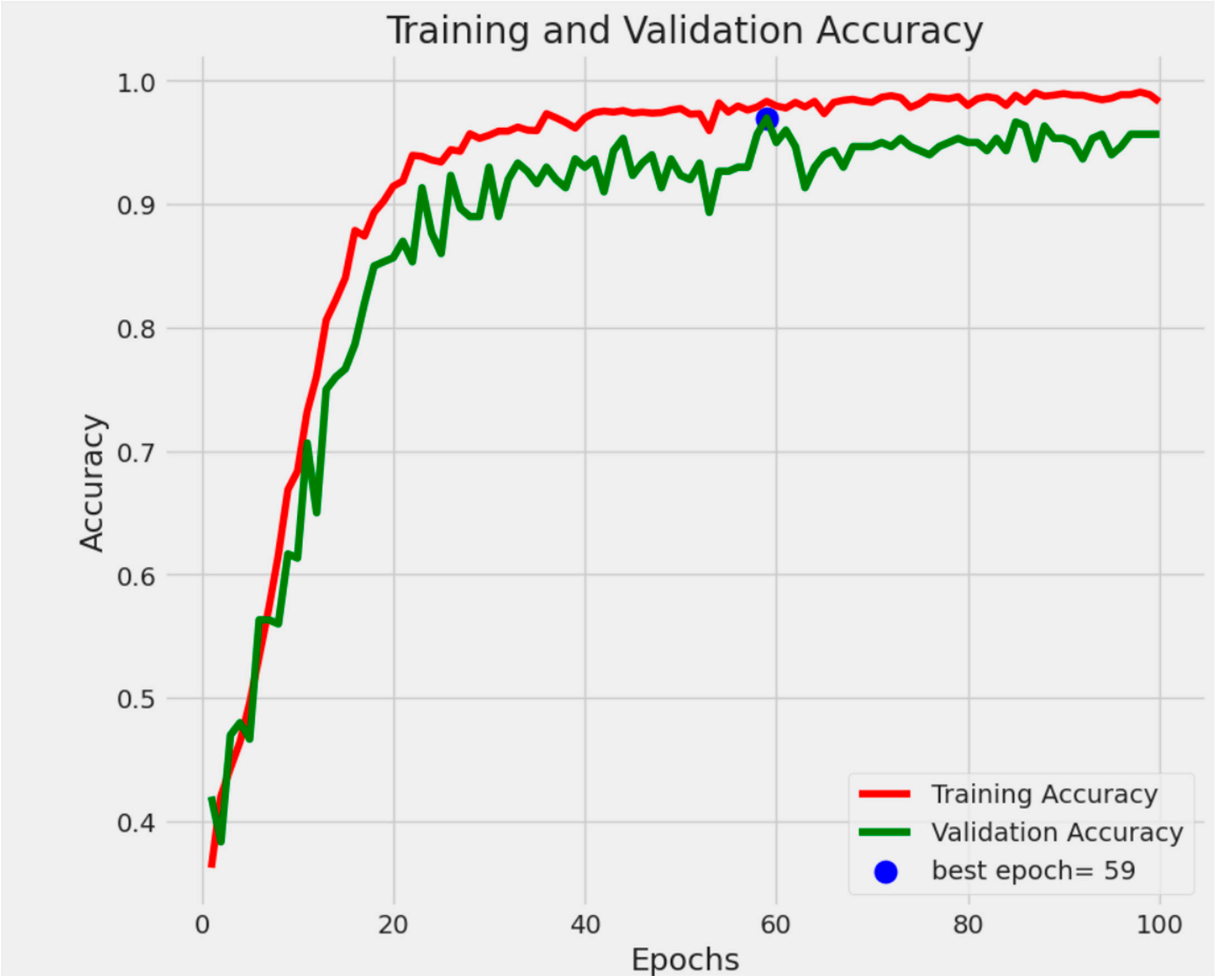
Training and Validation Accuracy
Accuracy
Epochs
1.0
0.9
0.8
0.7
0.6
0.5
0.4
0
20
40
60
80
100
Training Accuracy
Validation Accuracy
best epoch= 59

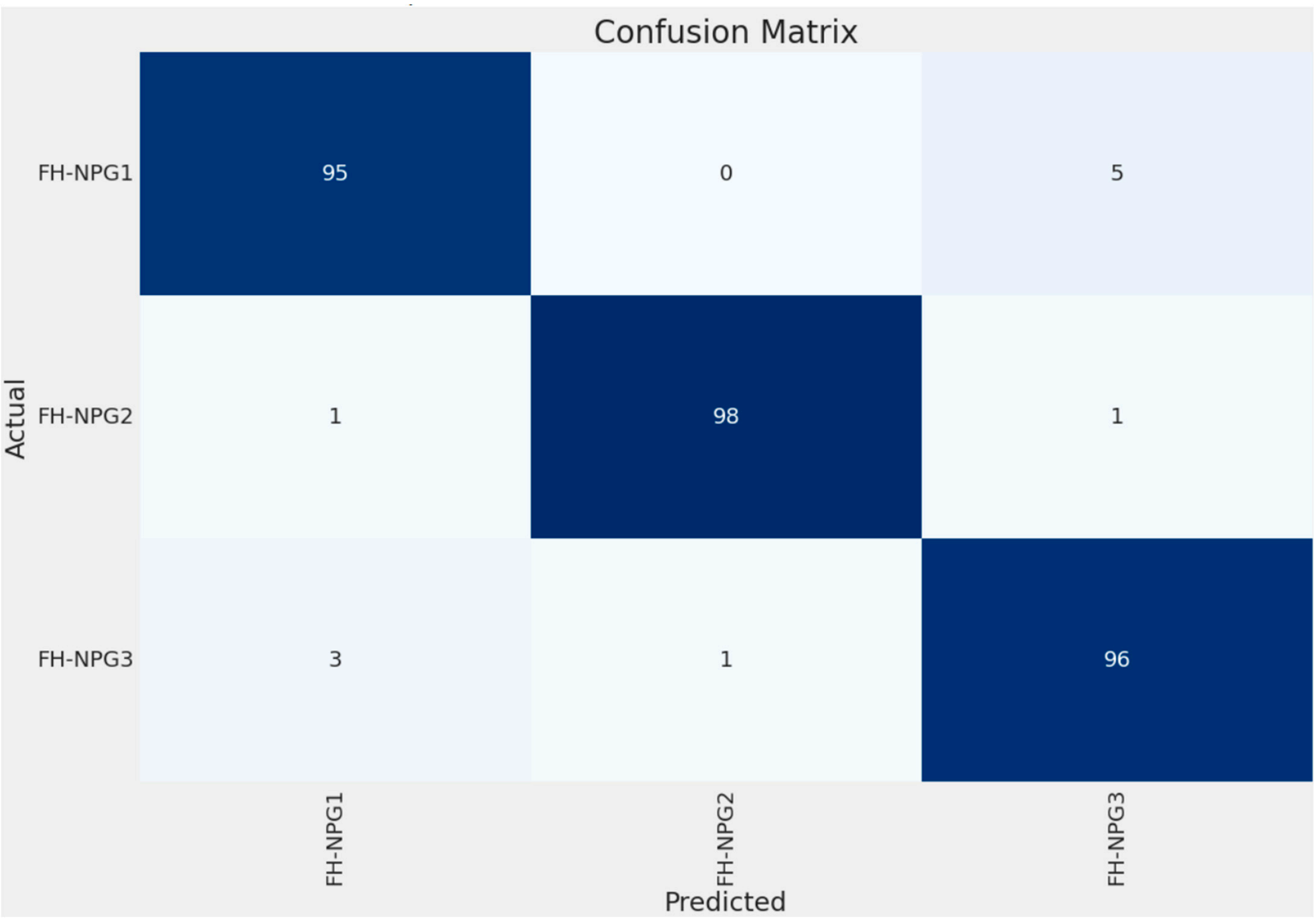


```
there were 11 errors in 300 tests for an accuracy of  96.33


Classification Report:
----------------------
              precision    recall  f1-score   support

     FH-NPG1     0.9596    0.9500    0.9548       100
     FH-NPG2     0.9899    0.9800    0.9849       100
     FH-NPG3     0.9412    0.9600    0.9505       100

    accuracy                         0.9633       300
   macro avg     0.9636    0.9633    0.9634       300
weighted avg     0.9636    0.9633    0.9634       300
```

XCEPTION %33.33

VGG16 %32.33

VGG19 %31.33

NASNETMOBILE

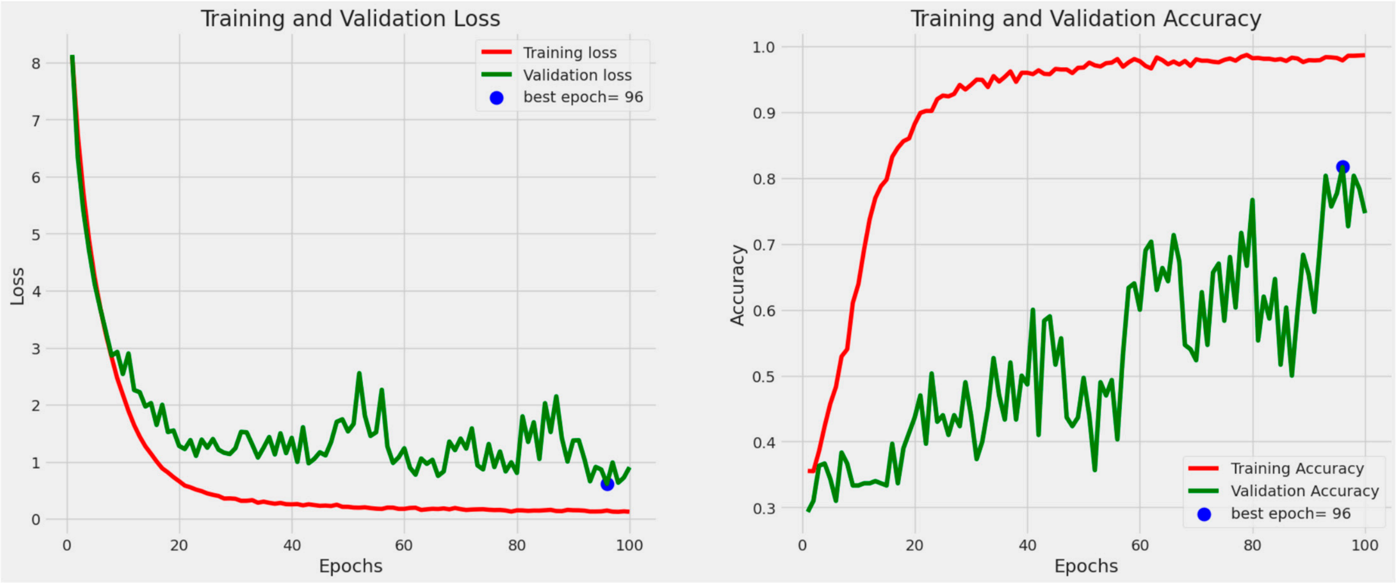

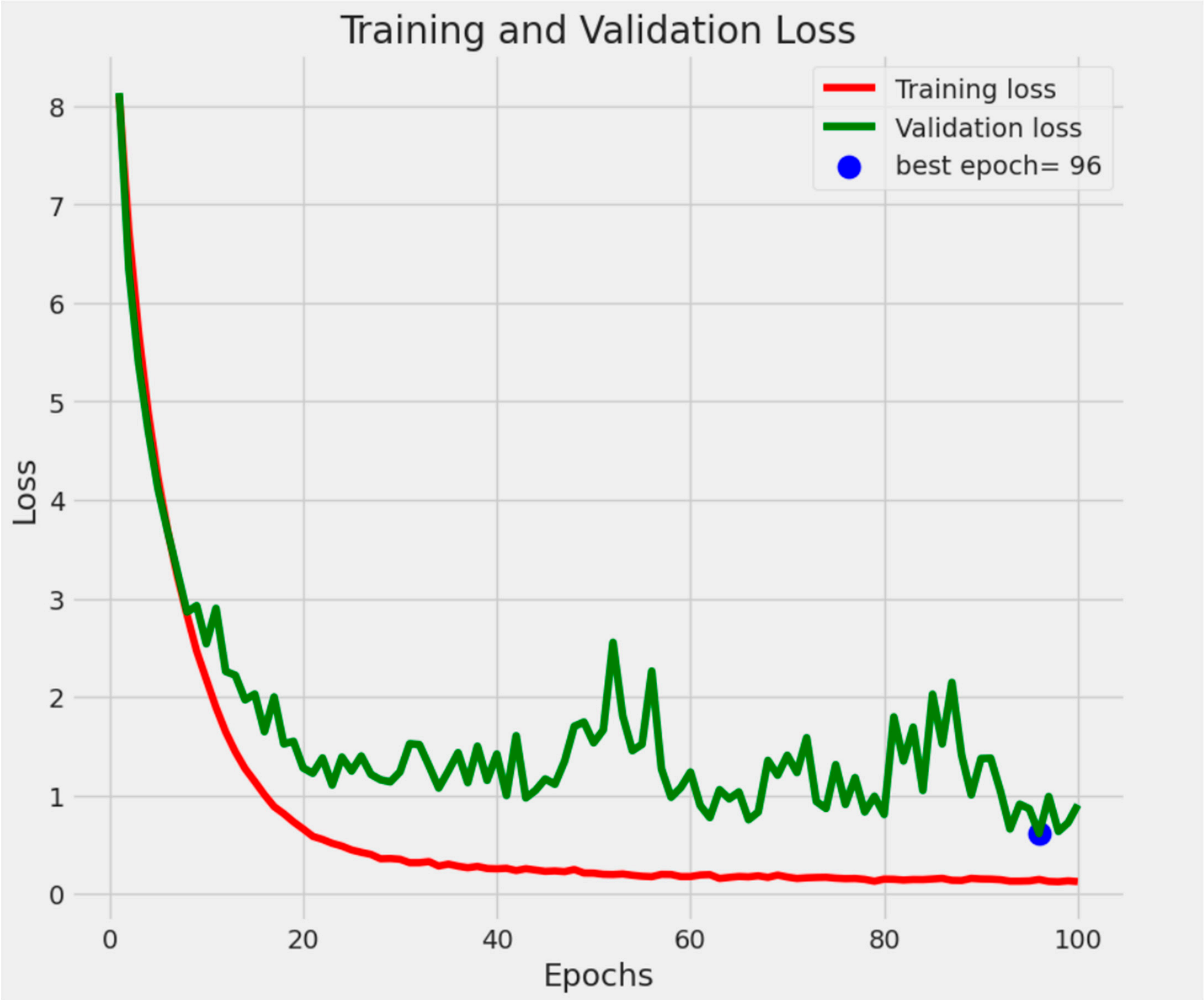

Training and Validation Loss
Training loss
Validation loss
best epoch= 96
Loss
Epochs
0
1
2
3
4
5
6
7
8
0
20
40
60
80
100

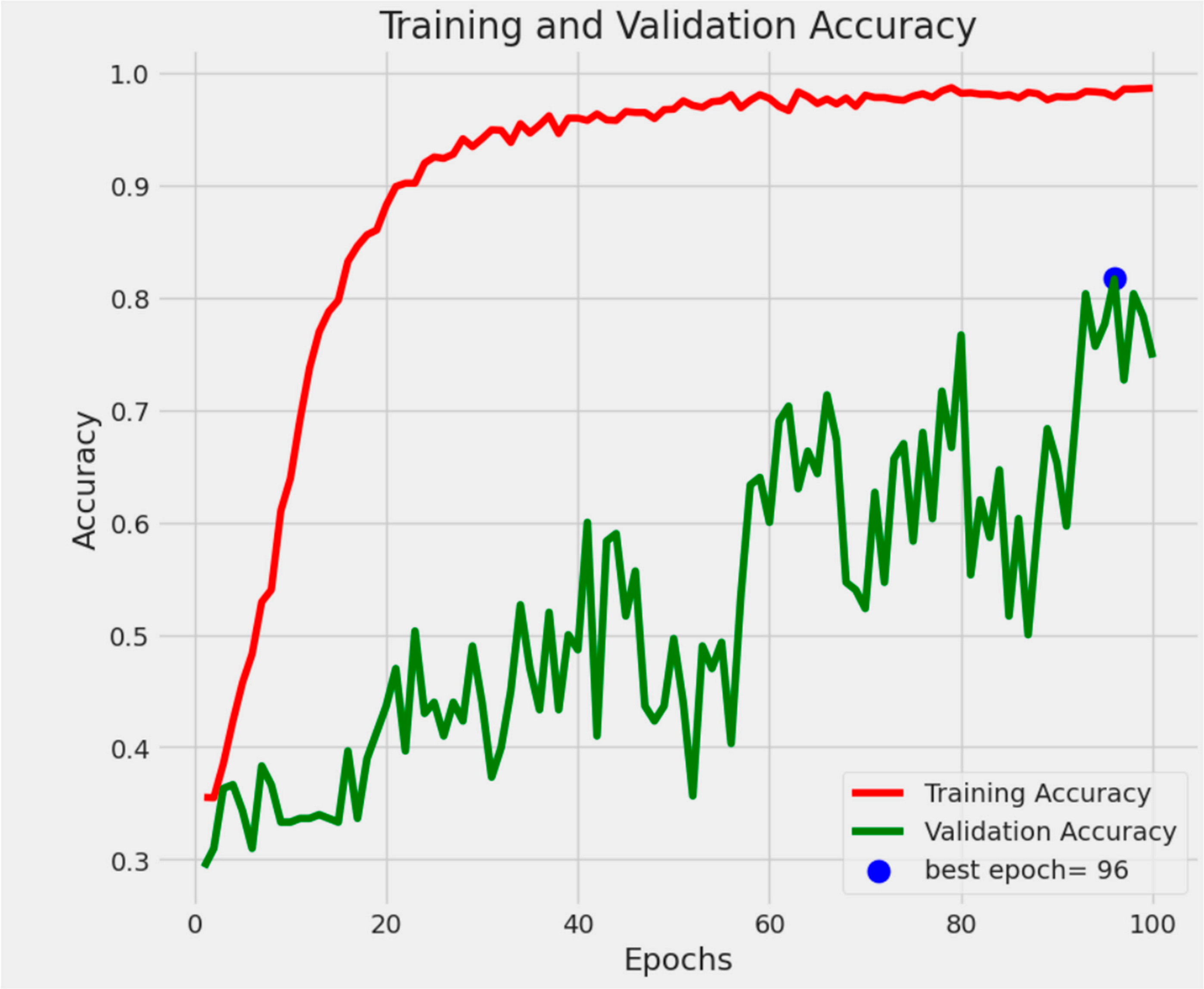
Training and Validation Accuracy
Accuracy
Epochs
Training Accuracy
Validation Accuracy
best epoch= 96
1.0
0.9
0.8
0.7
0.6
0.5
0.4
0.3
0
20
40
60
80
100

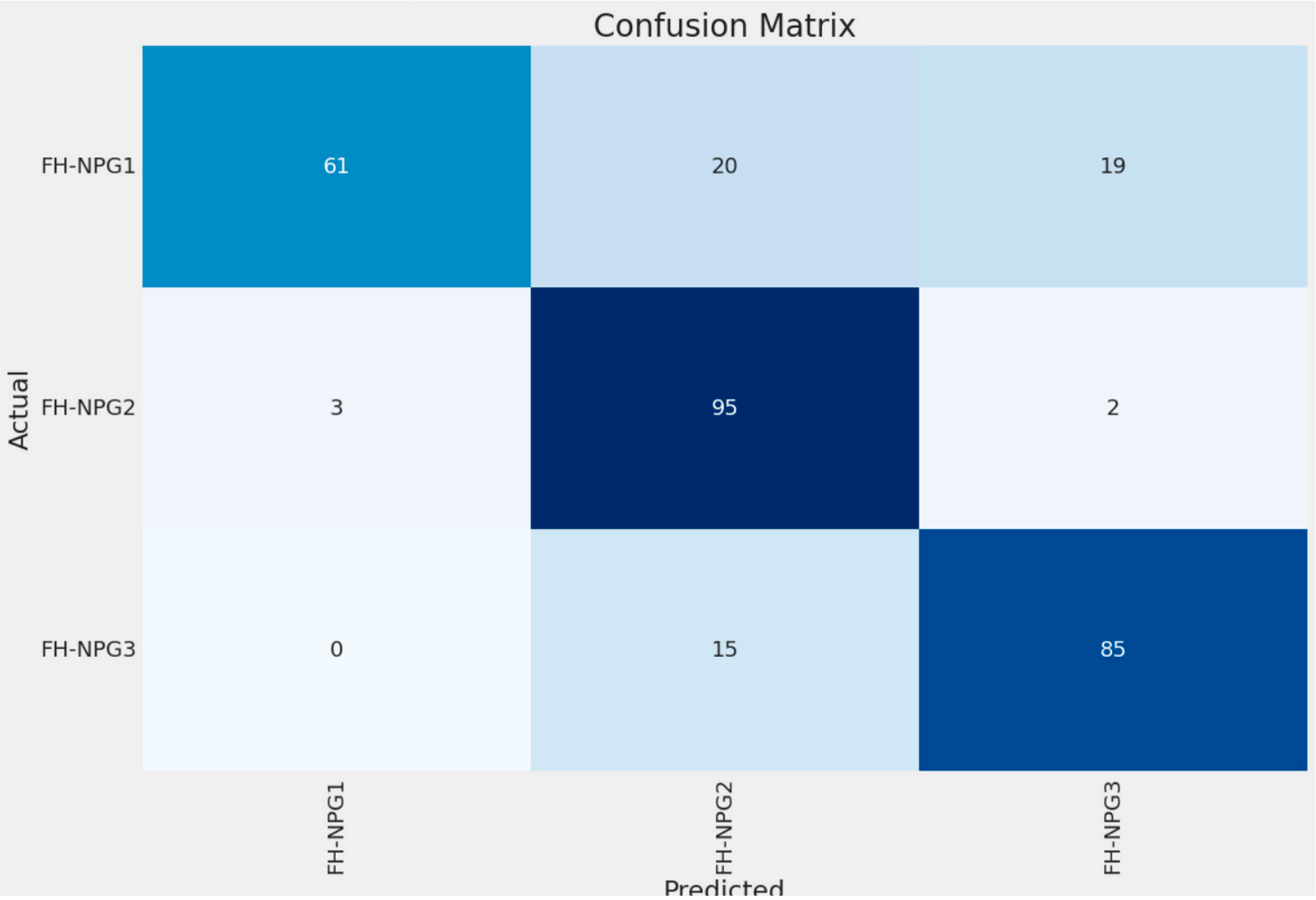


```
there were 59 errors in 300 tests for an accuracy of  80.33


Classification Report:
----------------------
              precision    recall  f1-score   support

     FH-NPG1     0.9531    0.6100    0.7439       100
     FH-NPG2     0.7308    0.9500    0.8261       100
     FH-NPG3     0.8019    0.8500    0.8252       100

    accuracy                         0.8033       300
   macro avg     0.8286    0.8033    0.7984       300
weighted avg     0.8286    0.8033    0.7984       300
```

RESNET101: %34.67

RESNET152: %35.33

RESNET50

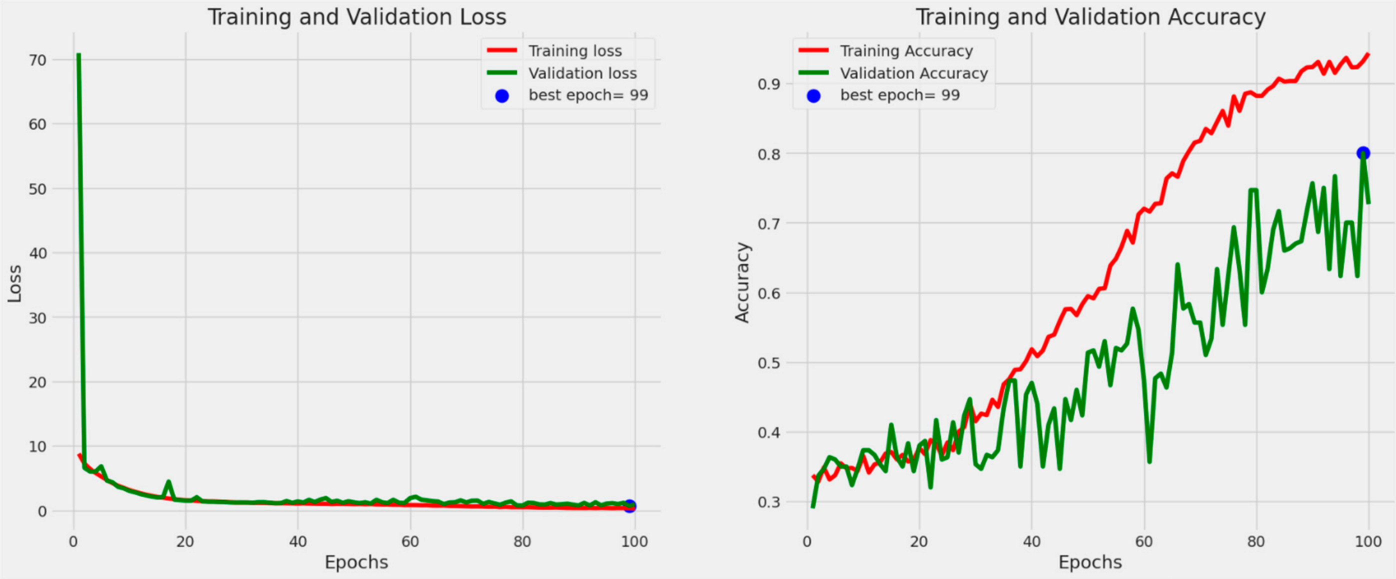

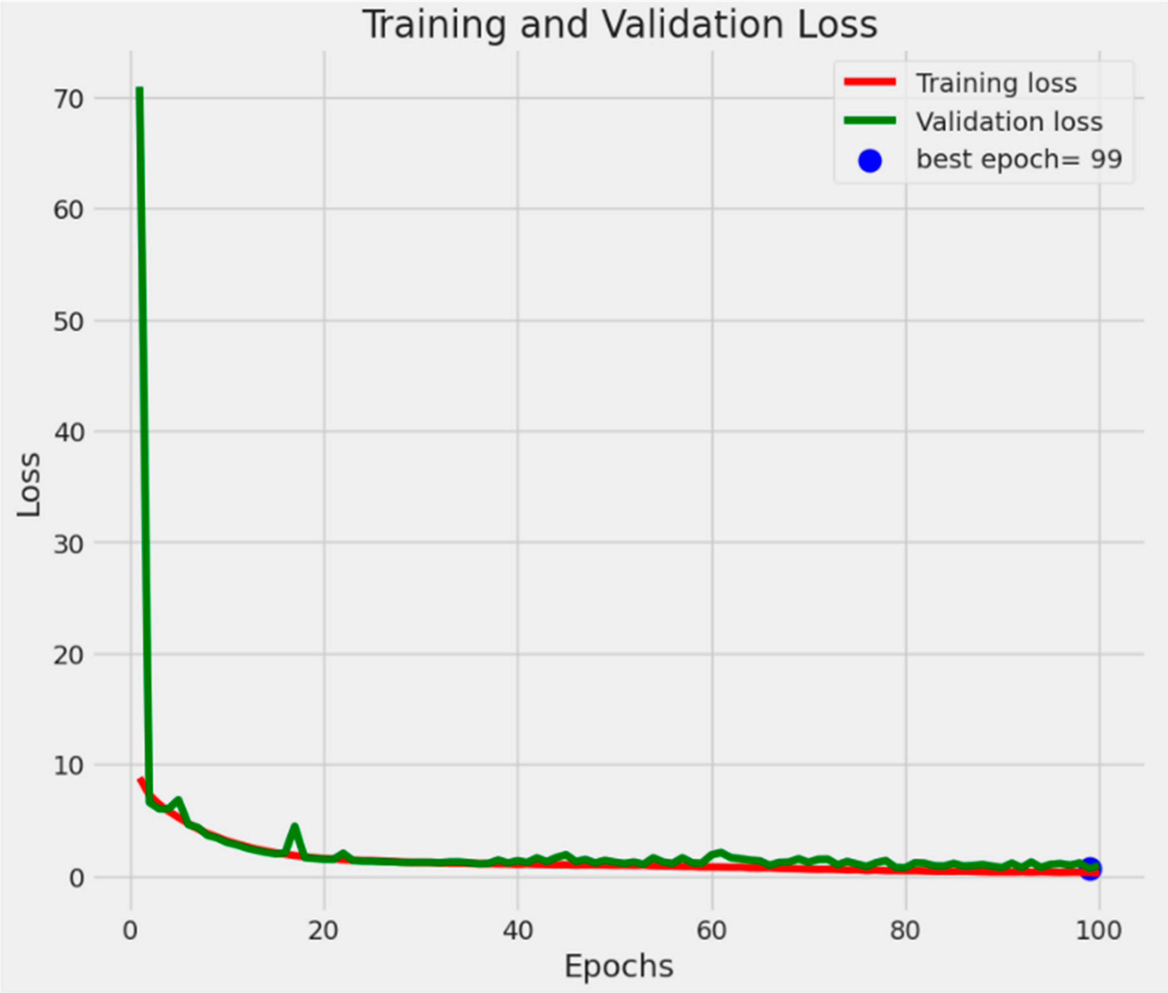

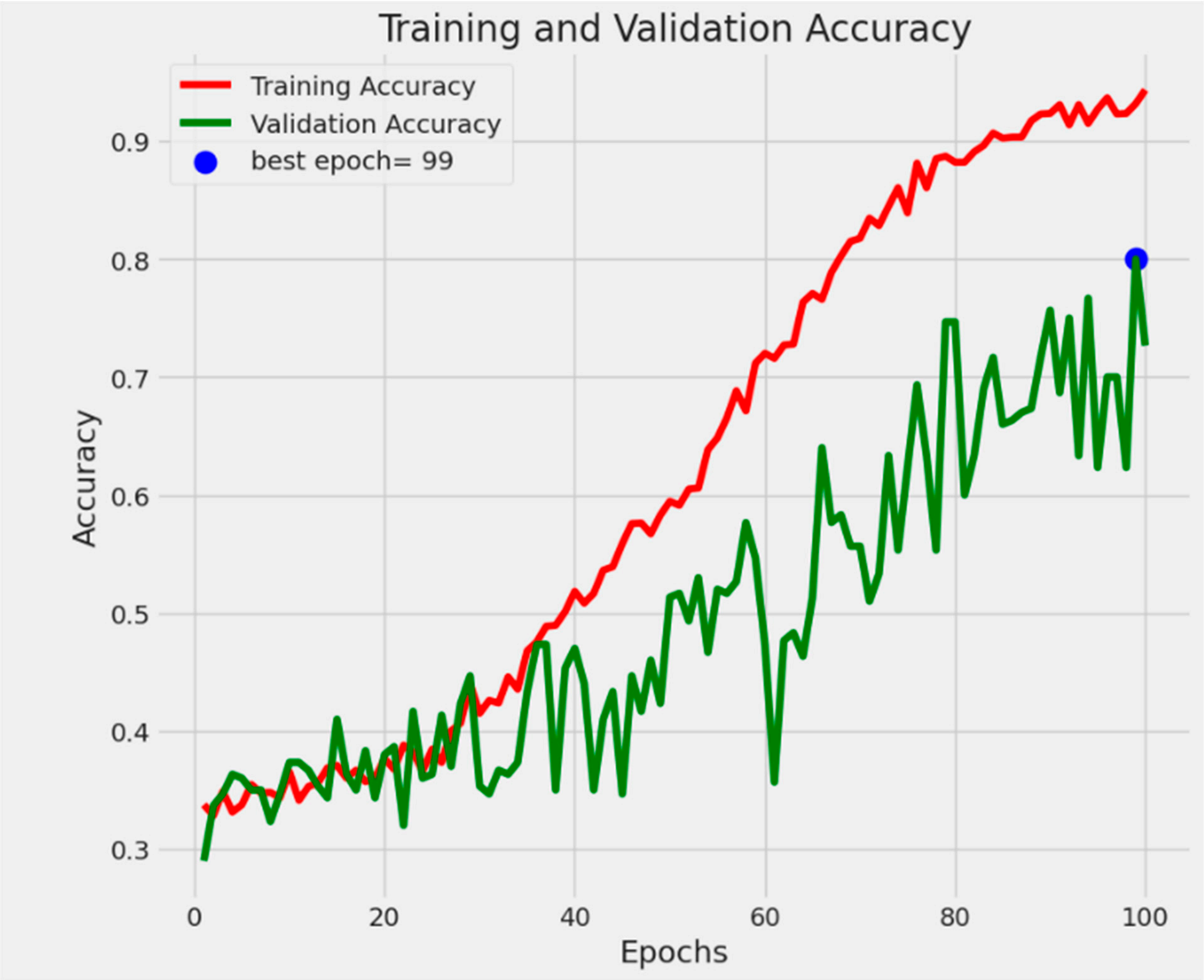

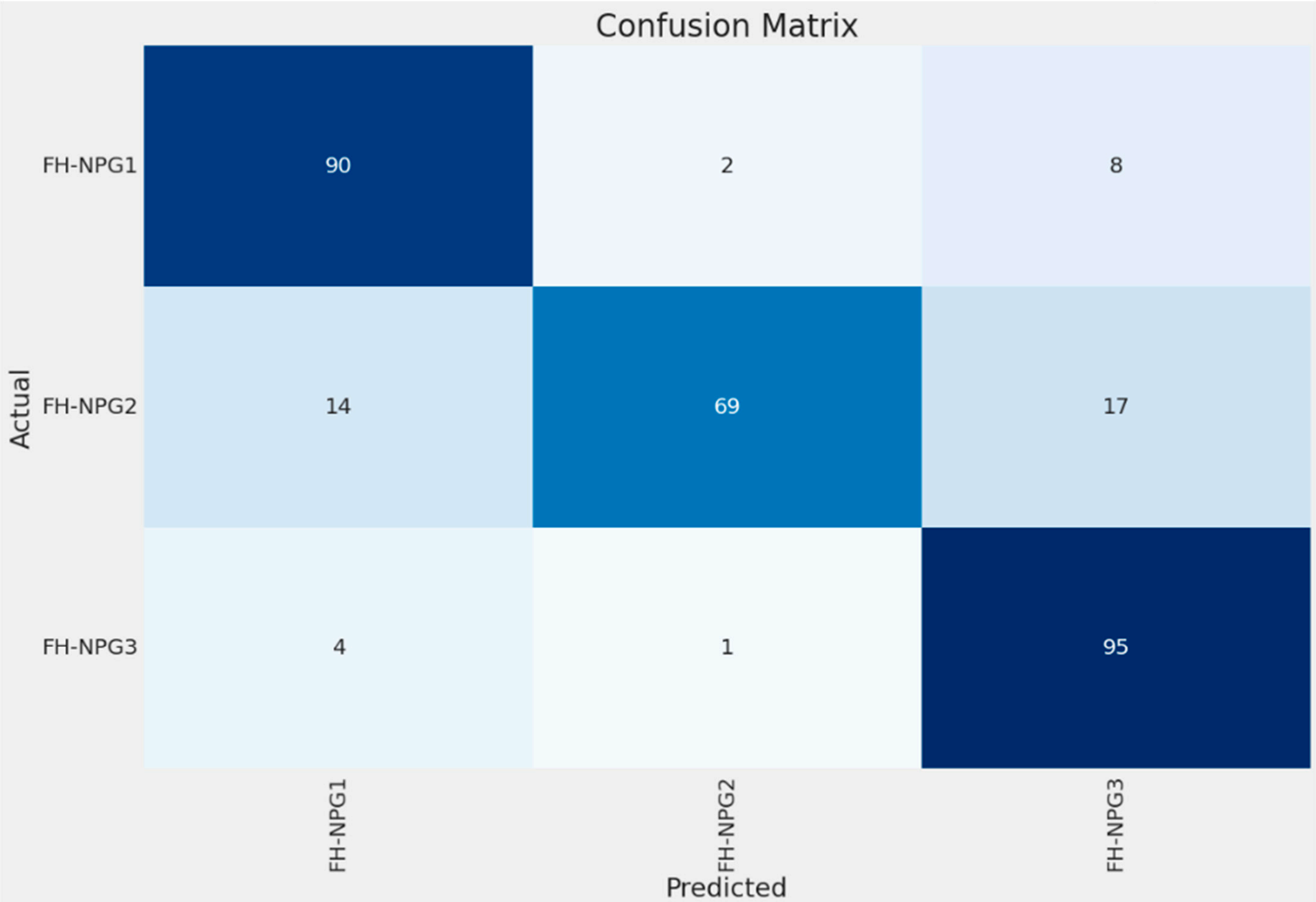


```
there were 46 errors in 300 tests for an accuracy of  84.67
```

Classification Report:
----

	precision	recall	f1-score	support
FH-NPG1	0.8333	0.9000	0.8654	100
FH-NPG2	0.9583	0.6900	0.8023	100
FH-NPG3	0.7917	0.9500	0.8636	100
accuracy			0.8467	300
macro avg	0.8611	0.8467	0.8438	300
weighted avg	0.8611	0.8467	0.8438	300

EFFICIENTNET V2

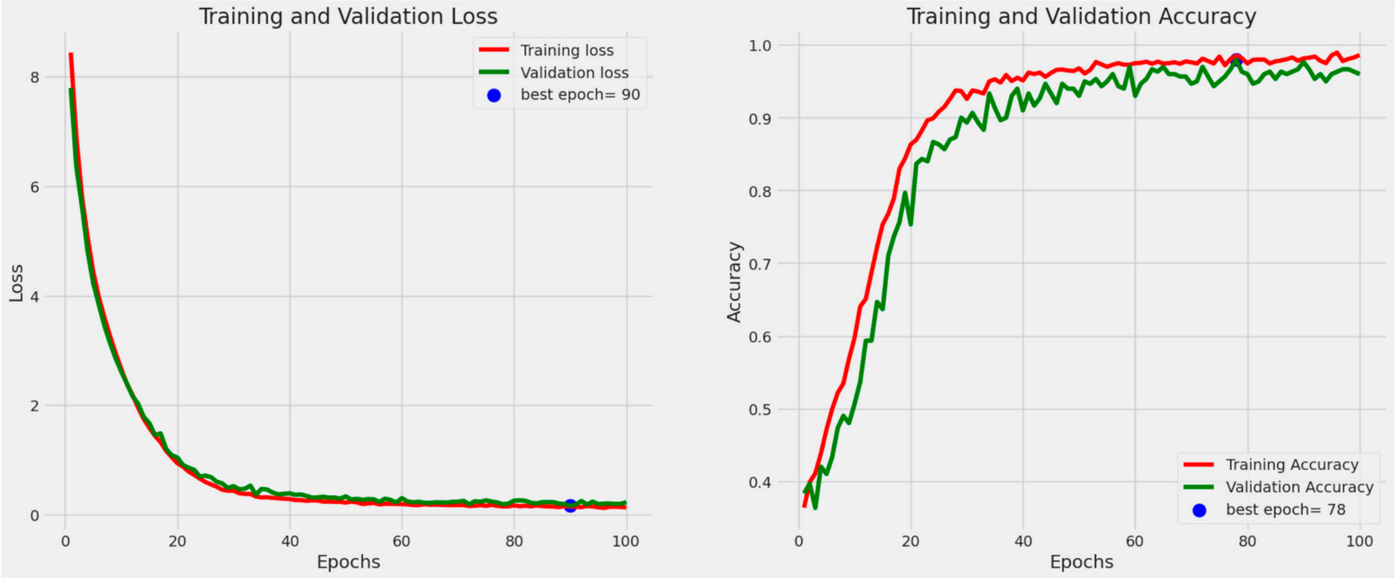

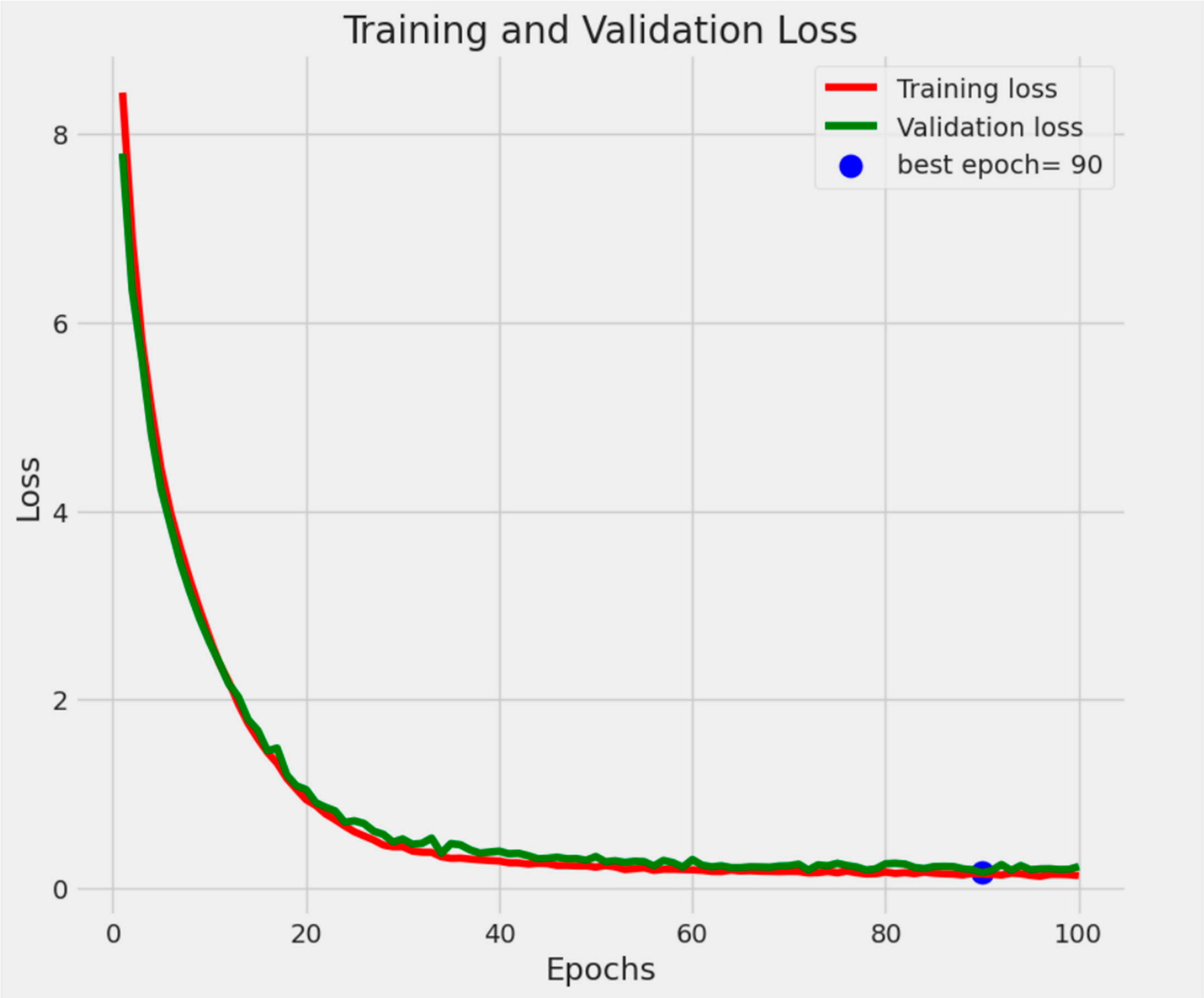

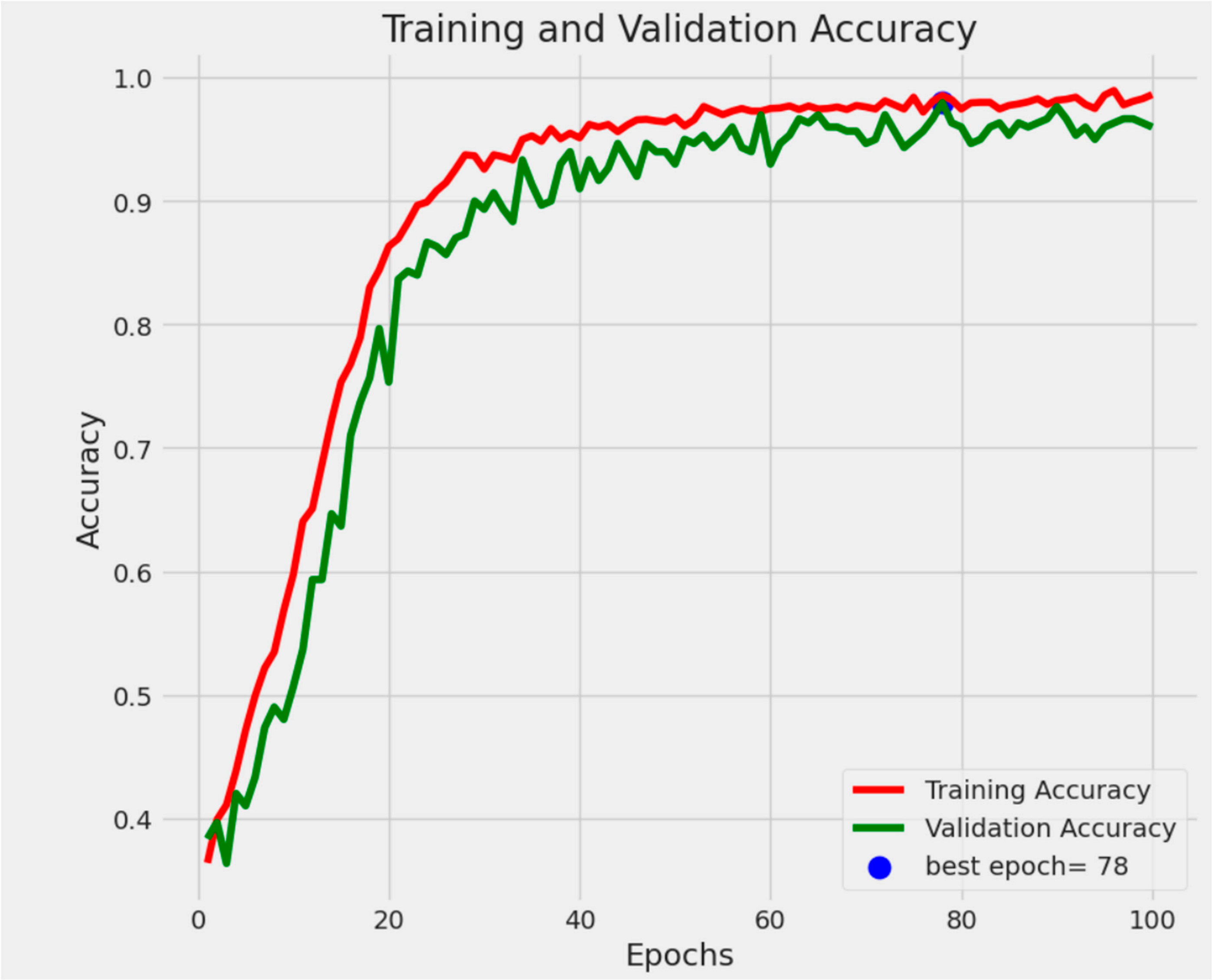

Training and Validation Accuracy
Accuracy
Epochs
1.0
0.9
0.8
0.7
0.6
0.5
0.4
0
20
40
60
80
100
Training Accuracy
Validation Accuracy
best epoch= 78

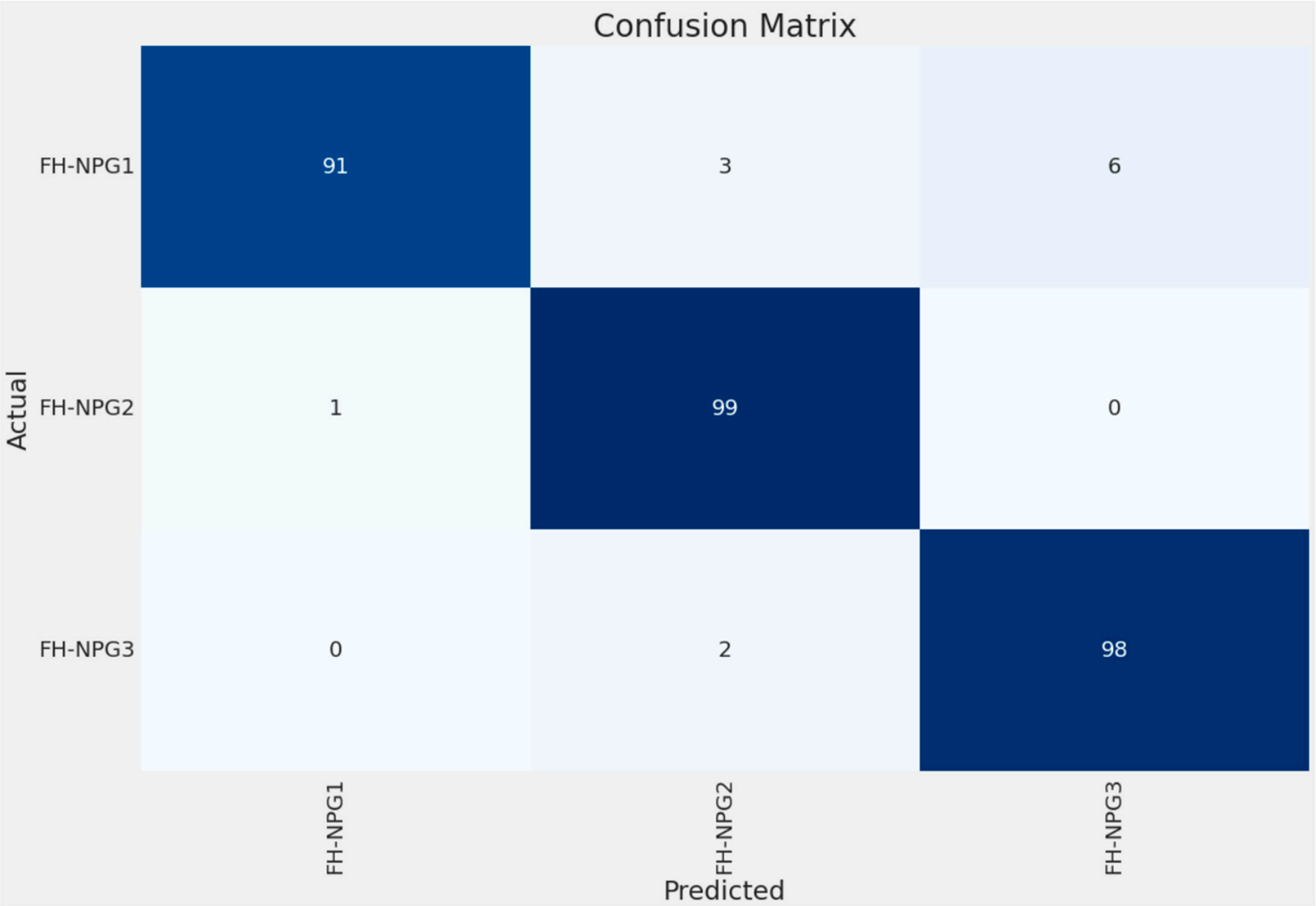

there were 12 errors in 300 tests for an accuracy of 96.00

Classification Report:
----

	precision	recall	f1-score	support
FH-NPG1	0.9891	0.9100	0.9479	100
FH-NPG2	0.9519	0.9900	0.9706	100
FH-NPG3	0.9423	0.9800	0.9608	100
accuracy			0.9600	300
macro avg	0.9611	0.9600	0.9598	300
weighted avg	0.9611	0.9600	0.9598	300
