# Supplementary material for: Profile Photograph Classification Performance of Deep Learning Algorithms Trained Using Cephalometric Measurements: A Preliminary Study
Source: Diagnostics (Basel). 2024 Aug 30;14(17):1916. doi: 10.3390/diagnostics14171916 (PMC11394270; doi:10.3390/diagnostics14171916)
Supplement: Supplementary file 1 [file diagnostics-14-01916-s001.zip › FMA.pdf]

MOBILENET V2

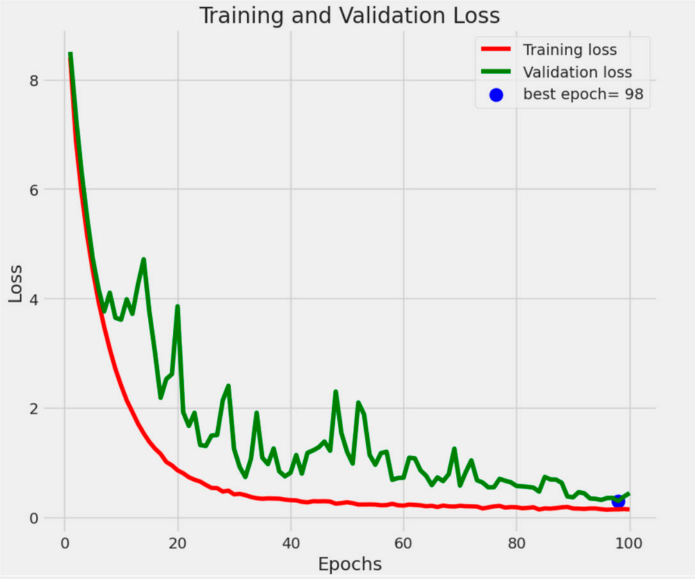

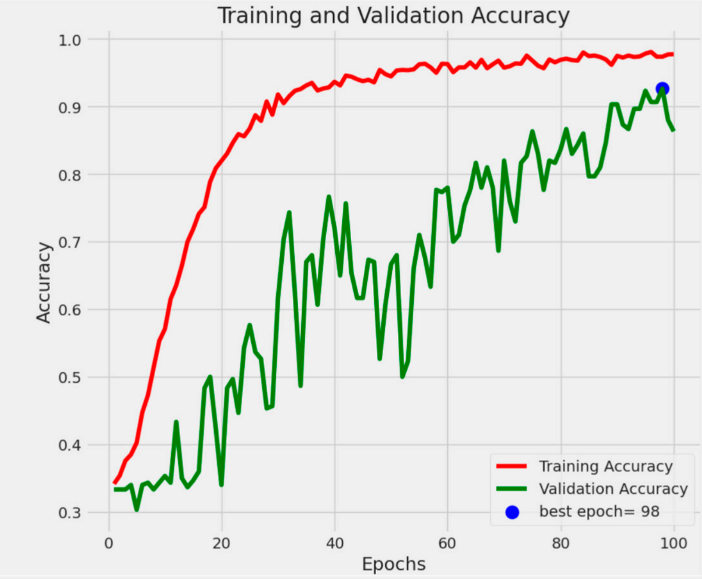

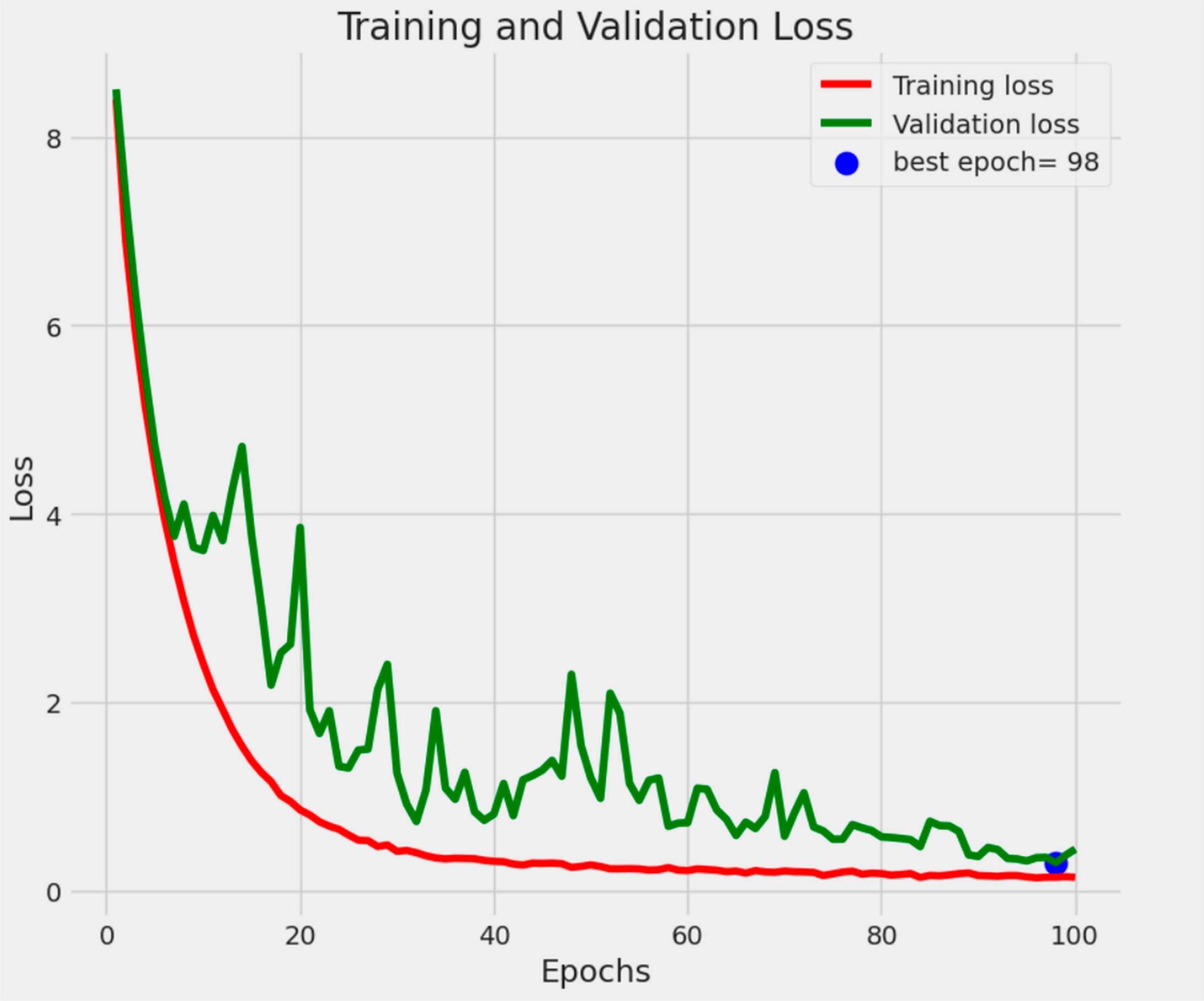

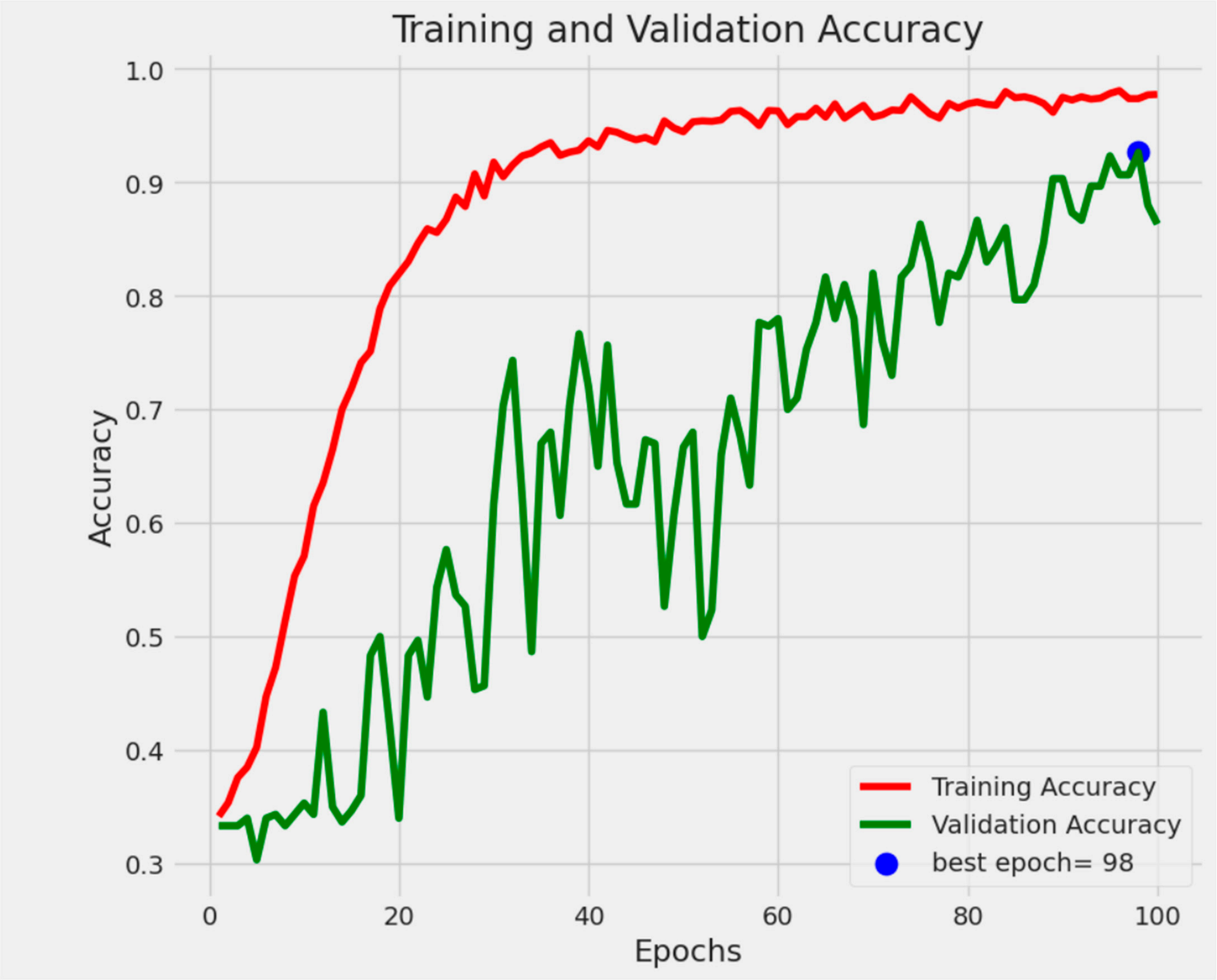
Training and Validation Accuracy
Accuracy
Epochs
1.0
0.9
0.8
0.7
0.6
0.5
0.4
0.3
0
20
40
60
80
100
Training Accuracy
Validation Accuracy
best epoch= 98

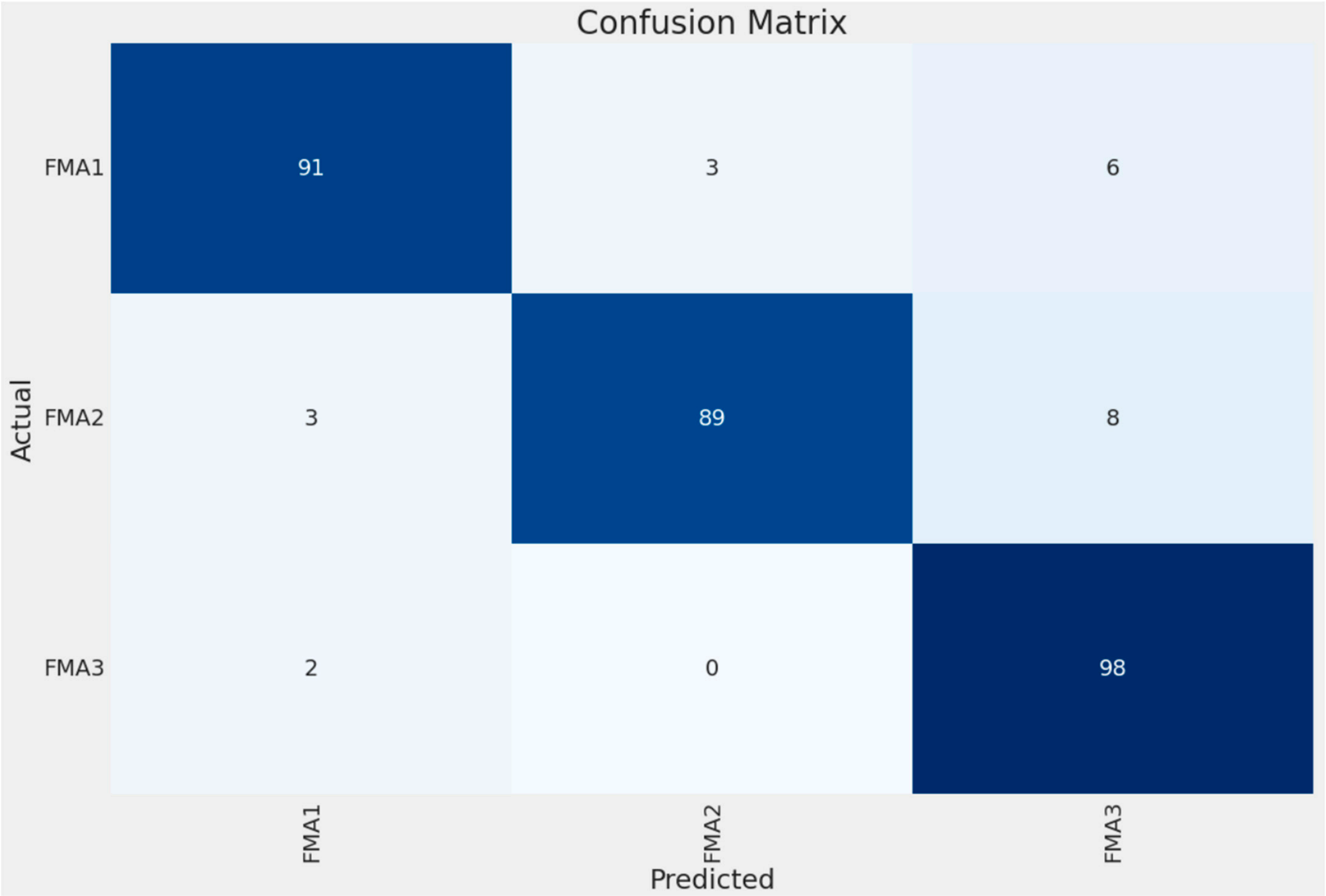

there were 22 errors in 300 tests for an accuracy of 92.67

```
Classification Report:
----------------------
              precision    recall  f1-score   support

        FMA1     0.9479    0.9100    0.9286       100
        FMA2     0.9674    0.8900    0.9271       100
        FMA3     0.8750    0.9800    0.9245       100

    accuracy                         0.9267       300
   macro avg     0.9301    0.9267    0.9267       300
weighted avg     0.9301    0.9267    0.9267       300
```

INCEPTION V3

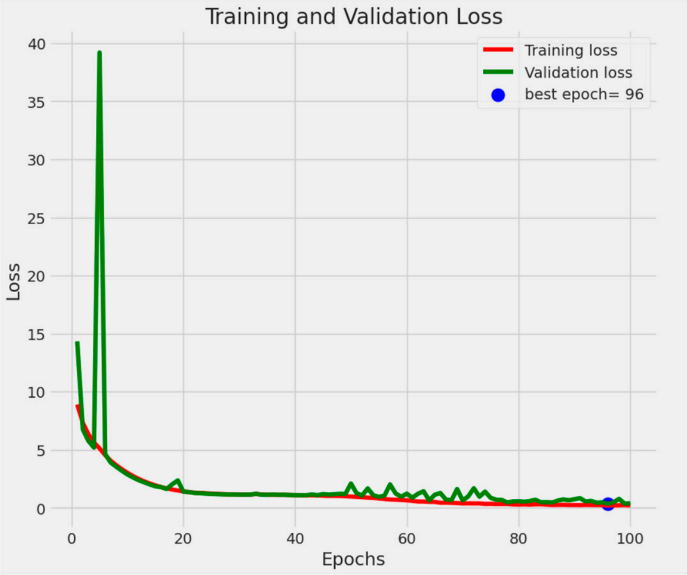

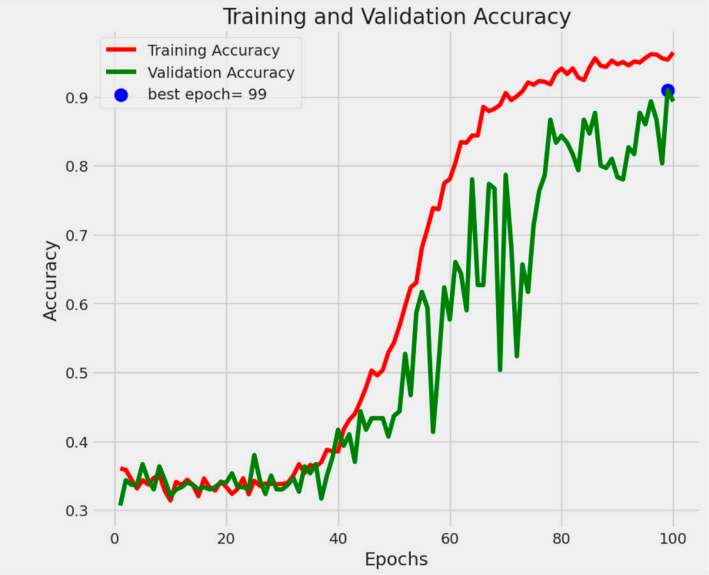

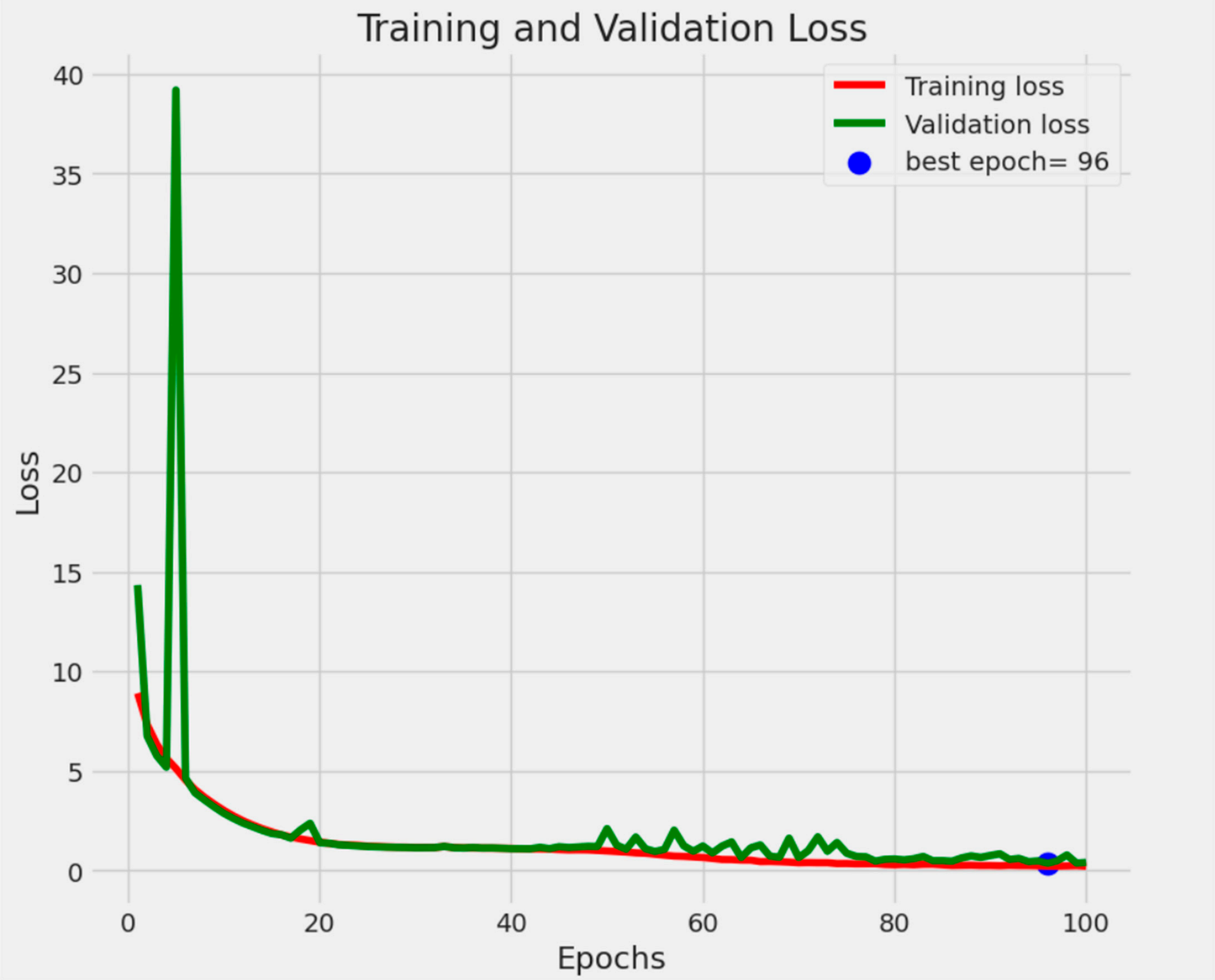

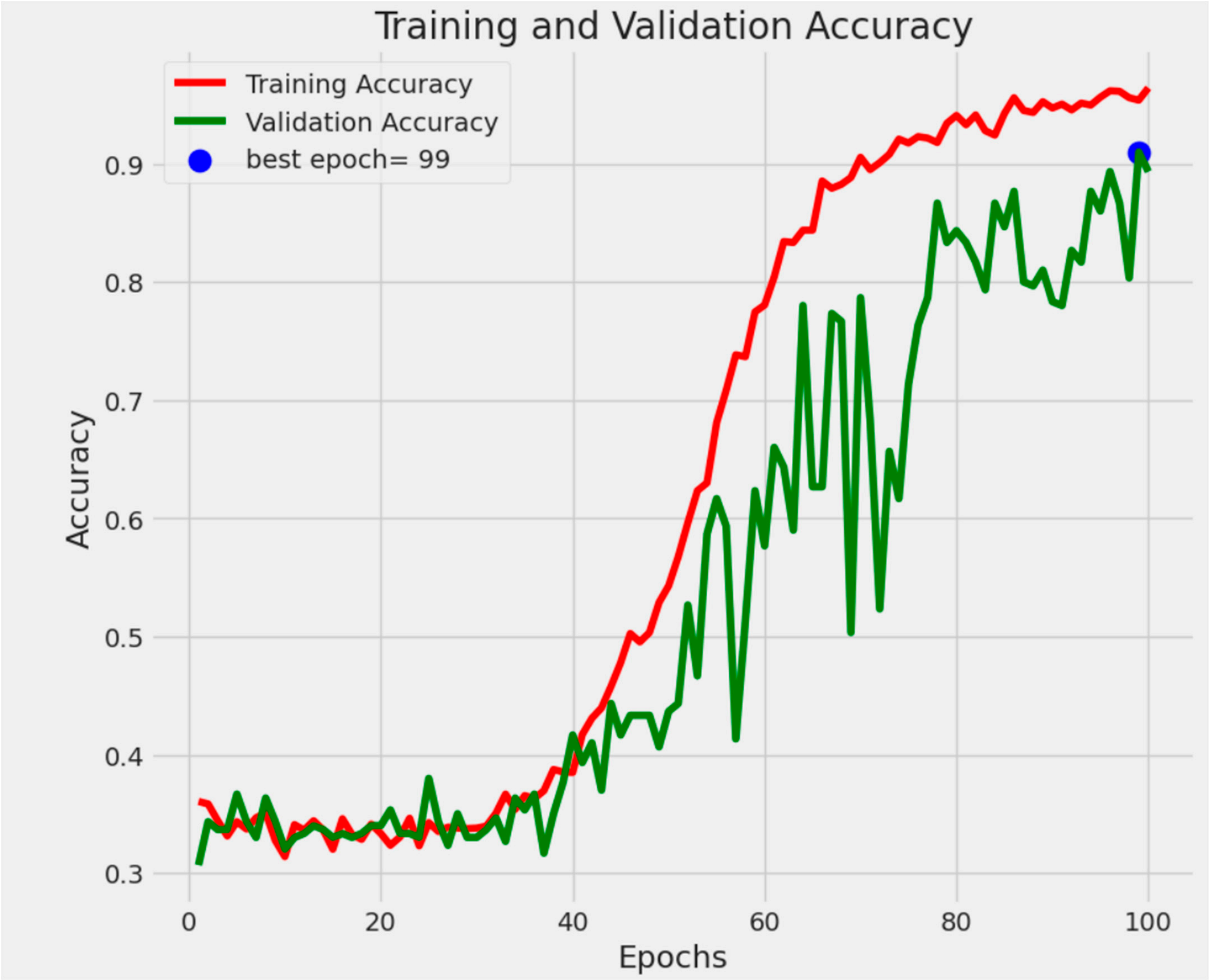

Training and Validation Accuracy
Training Accuracy
Validation Accuracy
best epoch= 99
Accuracy
Epochs
0.3
0.4
0.5
0.6
0.7
0.8
0.9
0
20
40
60
80
100

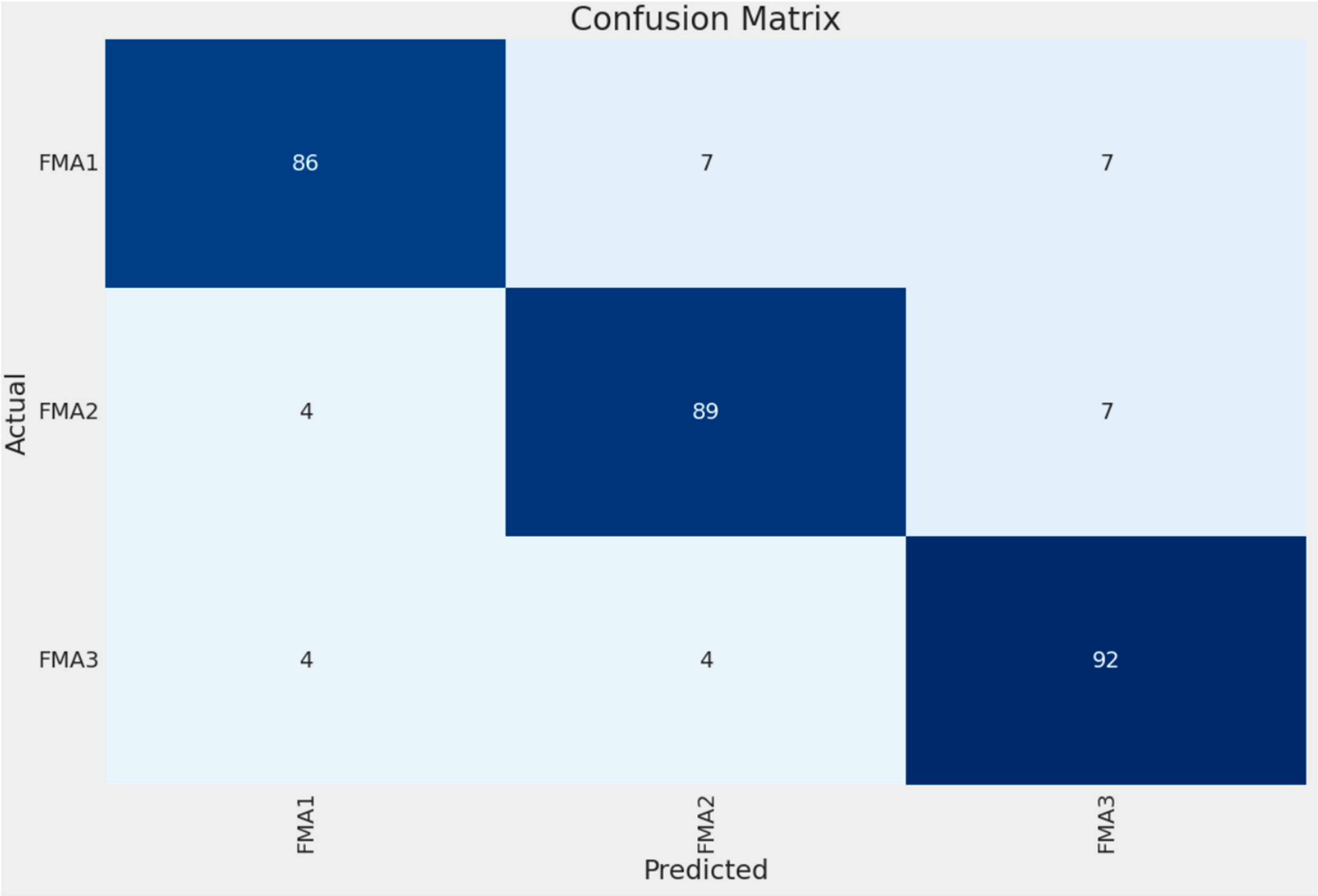


```
there were 33 errors in 300 tests for an accuracy of  89.00


Classification Report:
----------------------
              precision    recall  f1-score   support

        FMA1     0.9149    0.8600    0.8866       100
        FMA2     0.8900    0.8900    0.8900       100
        FMA3     0.8679    0.9200    0.8932       100

    accuracy                         0.8900       300
   macro avg     0.8909    0.8900    0.8899       300
weighted avg     0.8909    0.8900    0.8899       300
```

DENSENET 121

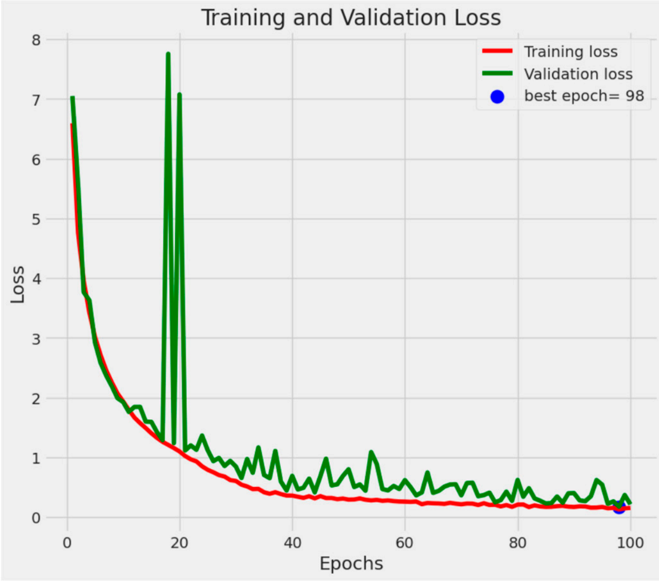

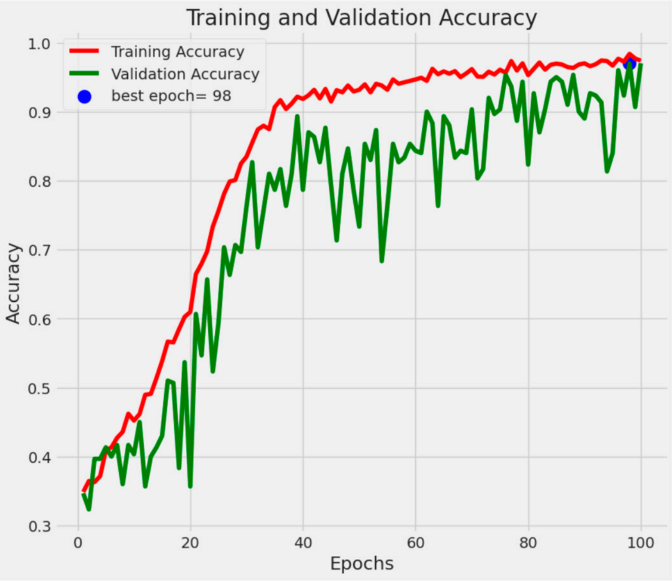

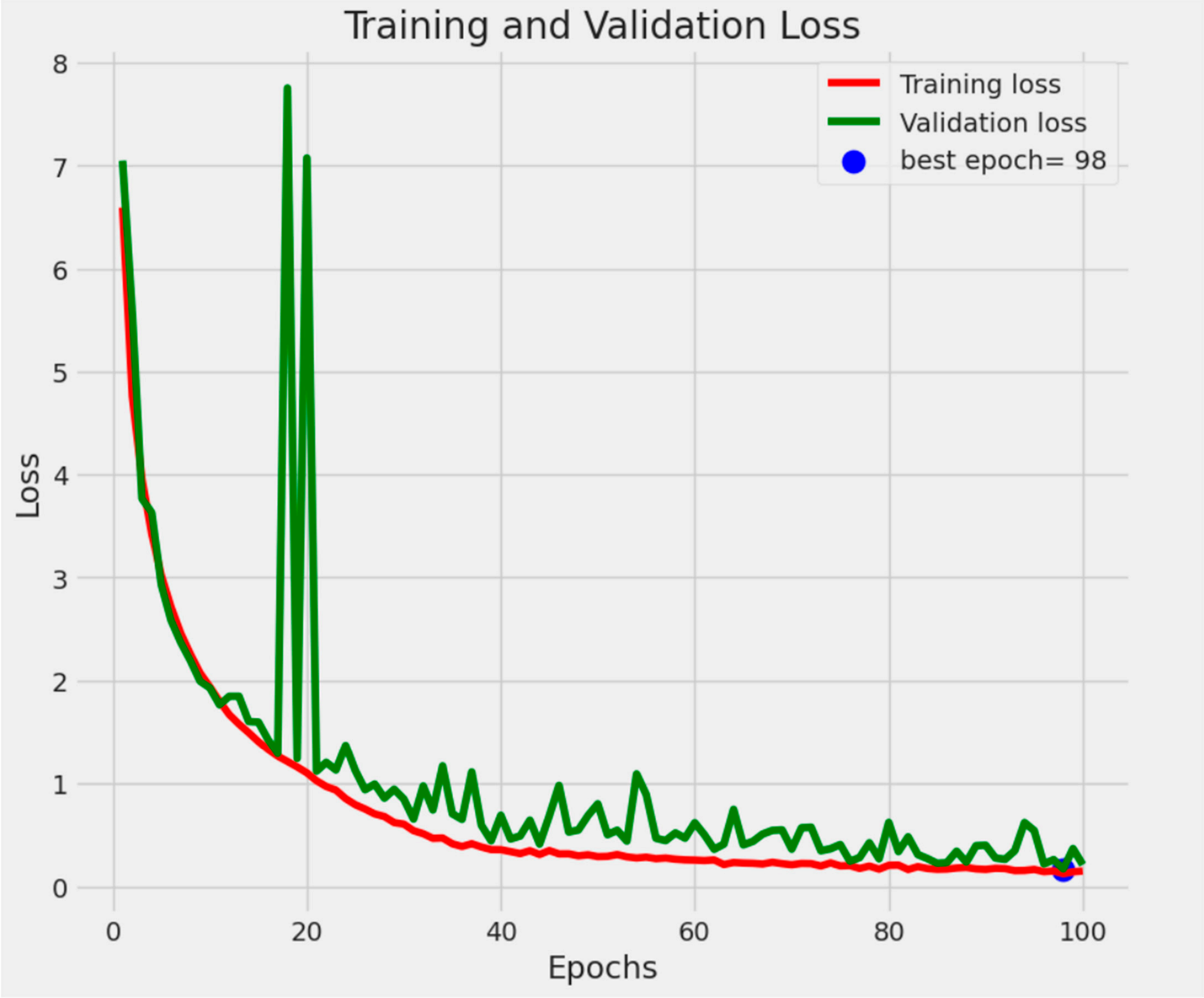

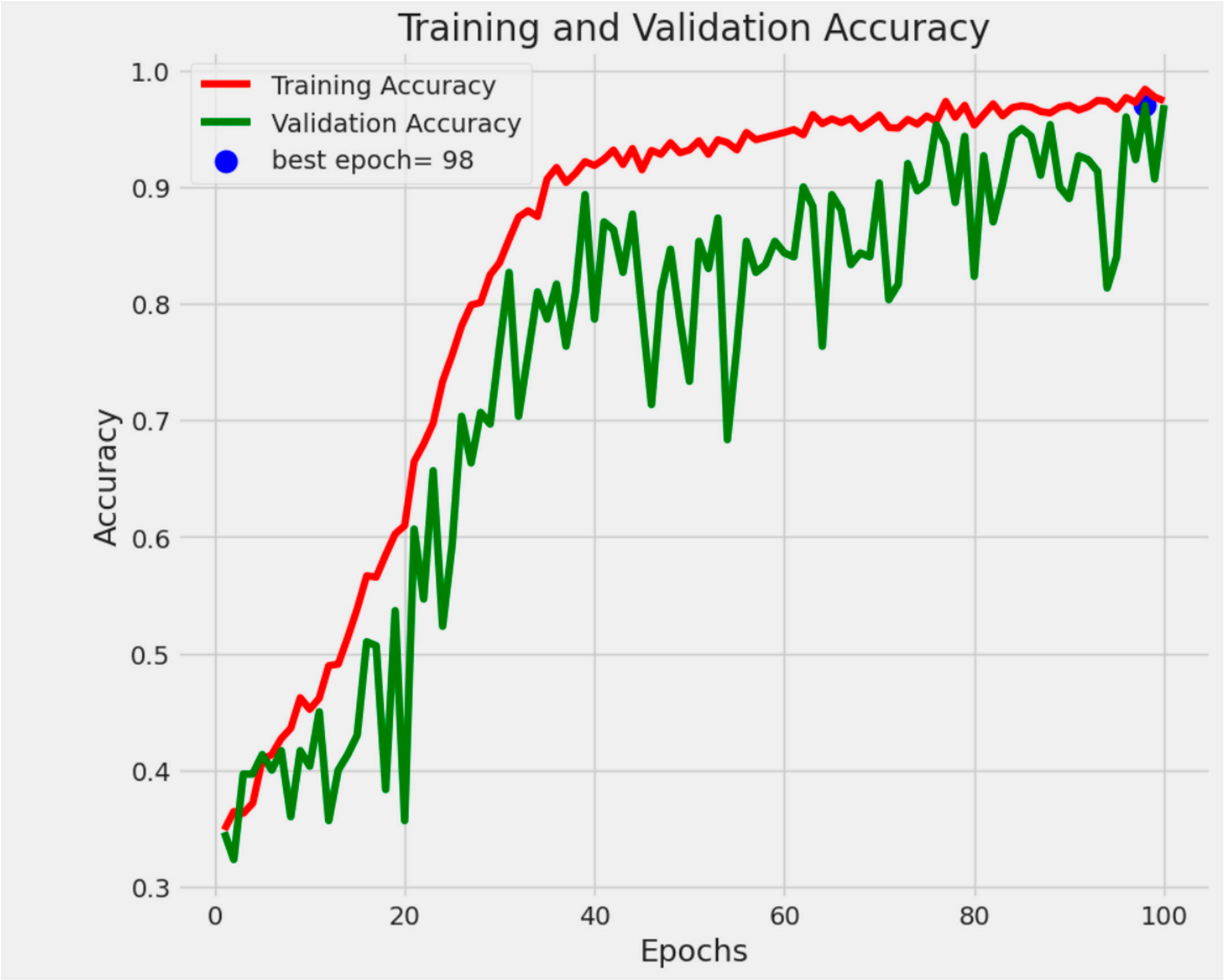
Training and Validation Accuracy
Training Accuracy
Validation Accuracy
best epoch= 98
Accuracy
Epochs
1.0
0.9
0.8
0.7
0.6
0.5
0.4
0.3
0
20
40
60
80
100

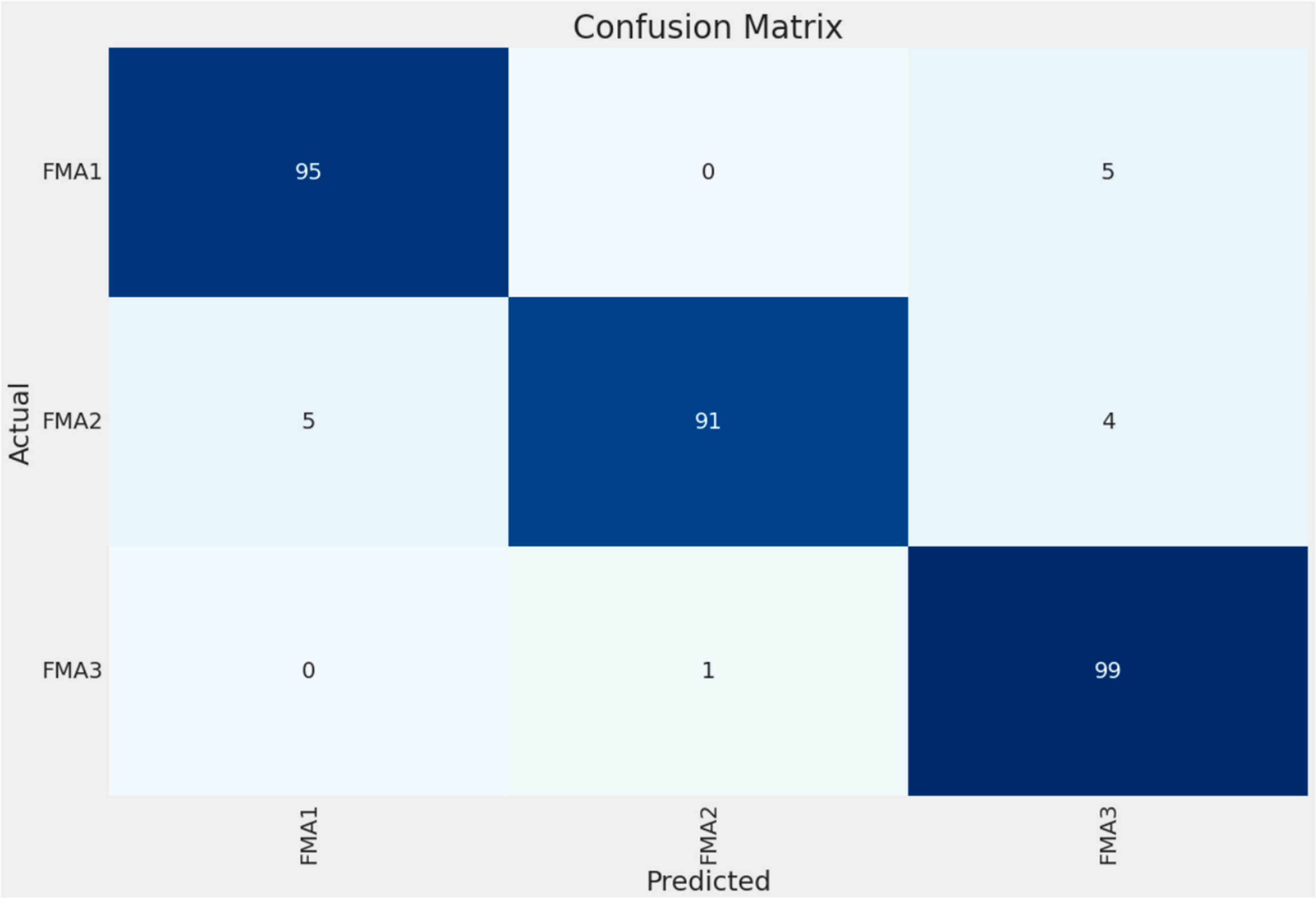


```
there were 15 errors in 300 tests for an accuracy of  95.00


Classification Report:
----------------------
              precision    recall  f1-score   support

        FMA1     0.9500    0.9500    0.9500       100
        FMA2     0.9891    0.9100    0.9479       100
        FMA3     0.9167    0.9900    0.9519       100

    accuracy                         0.9500       300
   macro avg     0.9519    0.9500    0.9499       300
weighted avg     0.9519    0.9500    0.9499       300
```

DENSENET 169

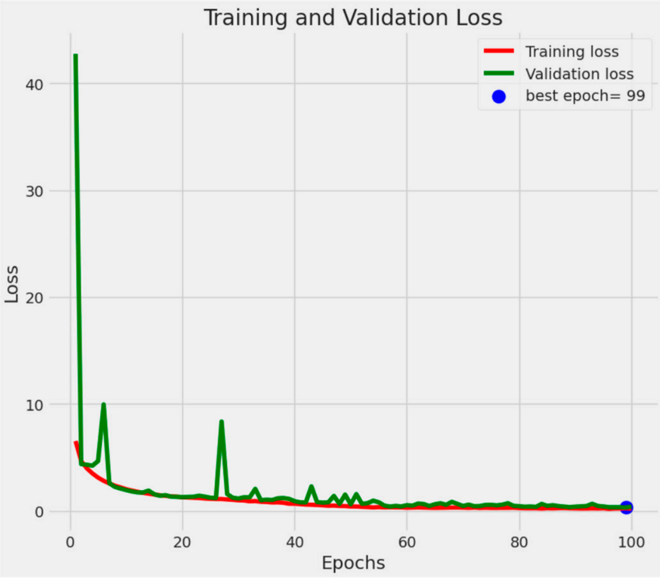

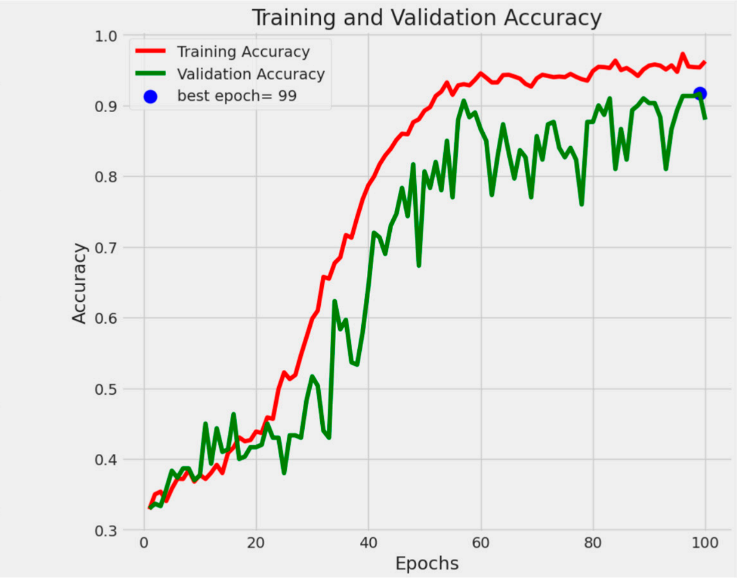

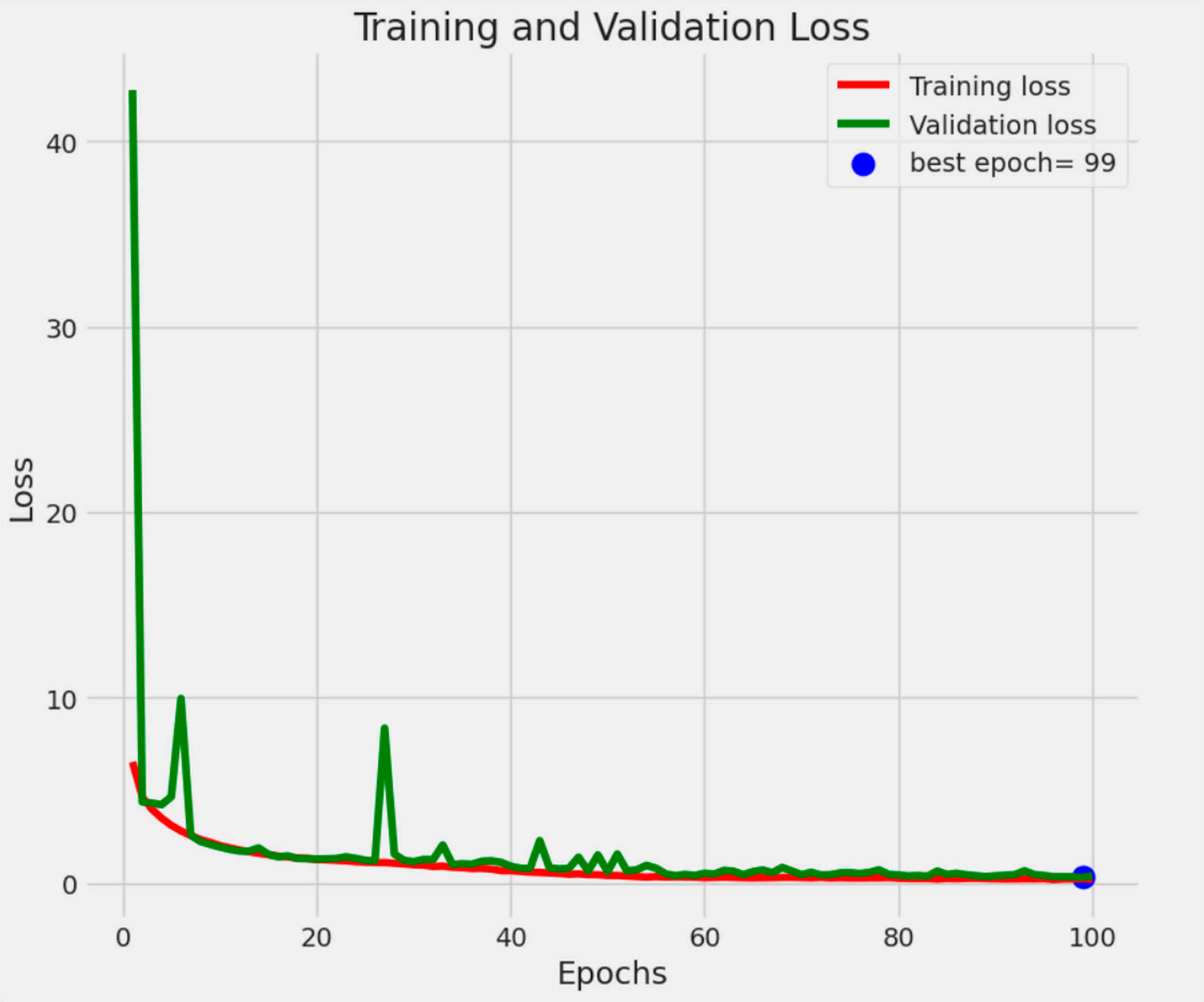

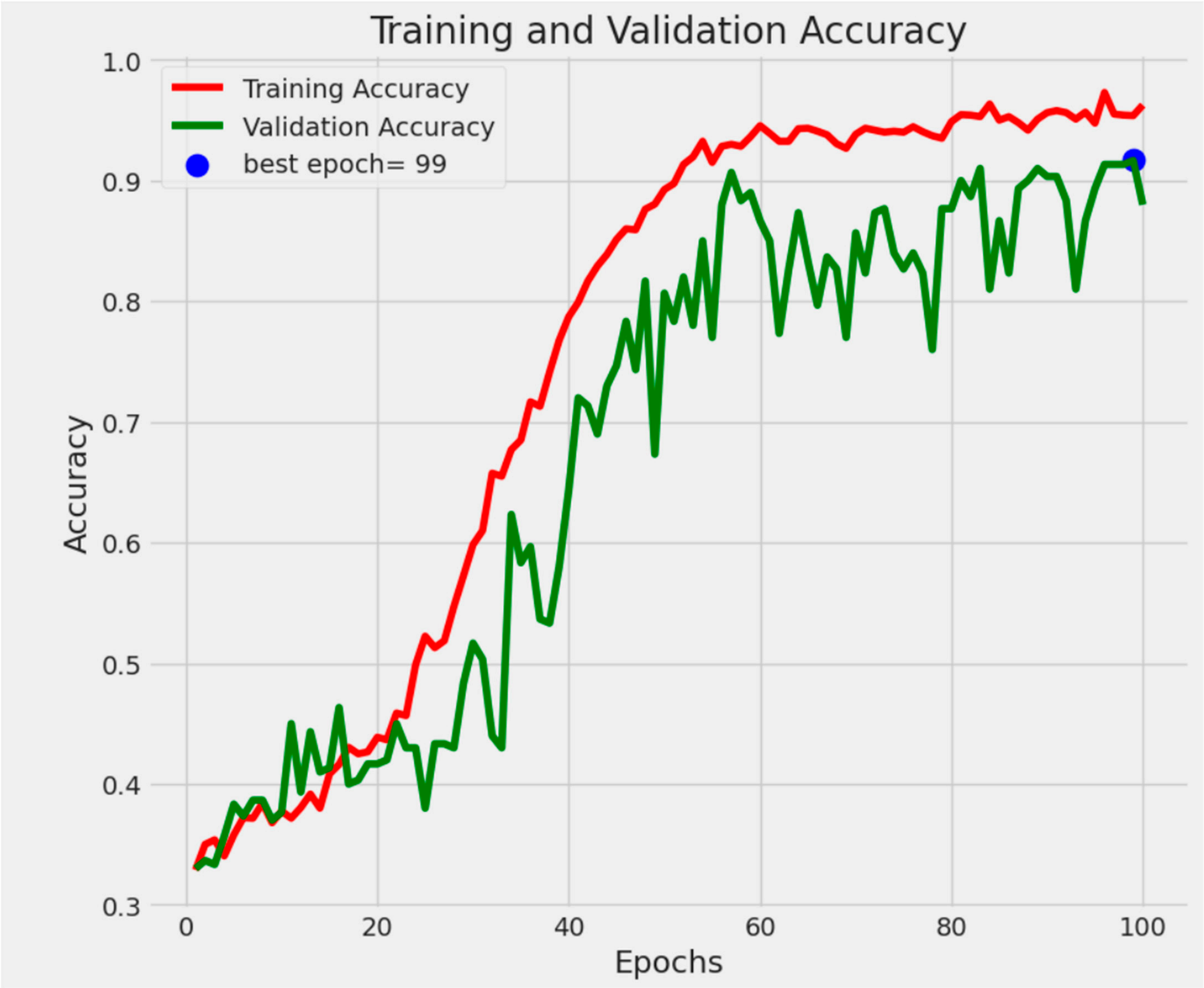

Training and Validation Accuracy
Training Accuracy
Validation Accuracy
best epoch= 99
Accuracy
Epochs
1.0
0.9
0.8
0.7
0.6
0.5
0.4
0.3
0
20
40
60
80
100

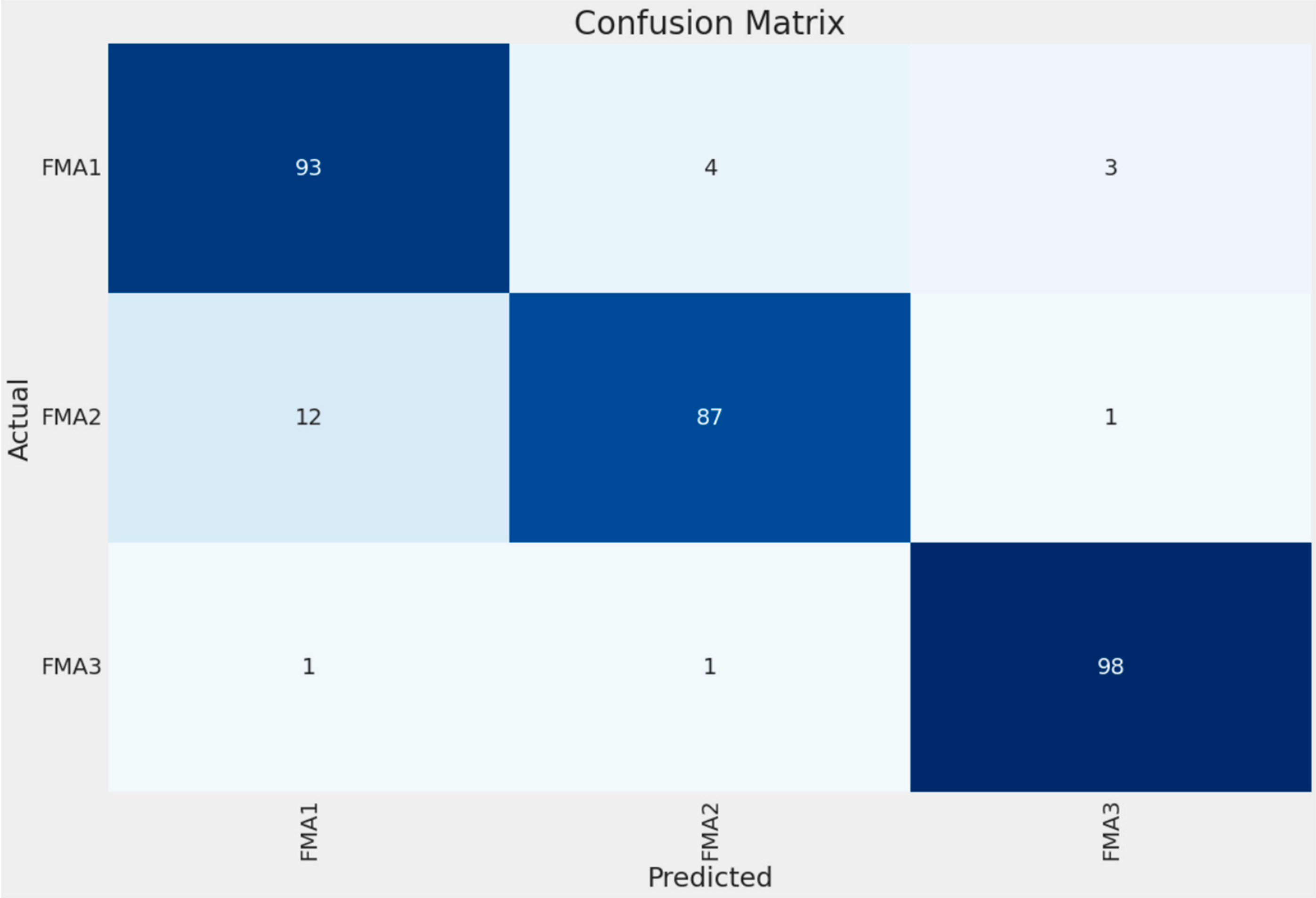

there were 22 errors in 300 tests for an accuracy of 92.67

Classification Report:
----

	precision	recall	f1-score	support
FMA1	0.8774	0.9300	0.9029	100
FMA2	0.9457	0.8700	0.9062	100
FMA3	0.9608	0.9800	0.9703	100
accuracy			0.9267	300
macro avg	0.9279	0.9267	0.9265	300
weighted avg	0.9279	0.9267	0.9265	300

DENSENET 201

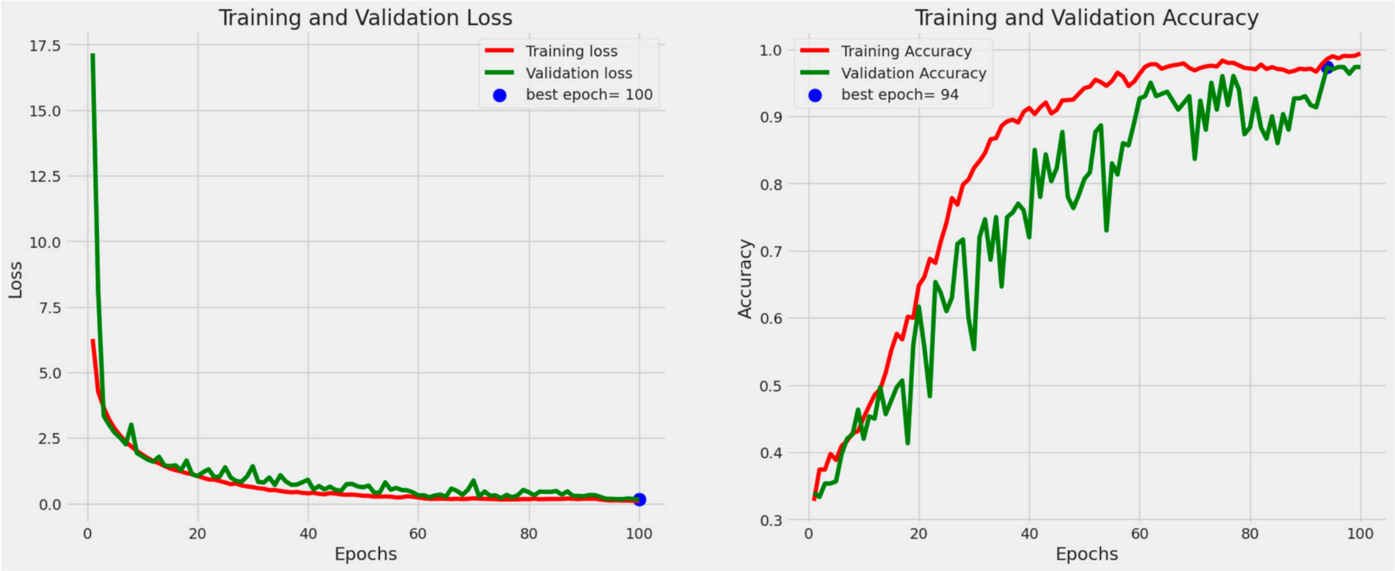

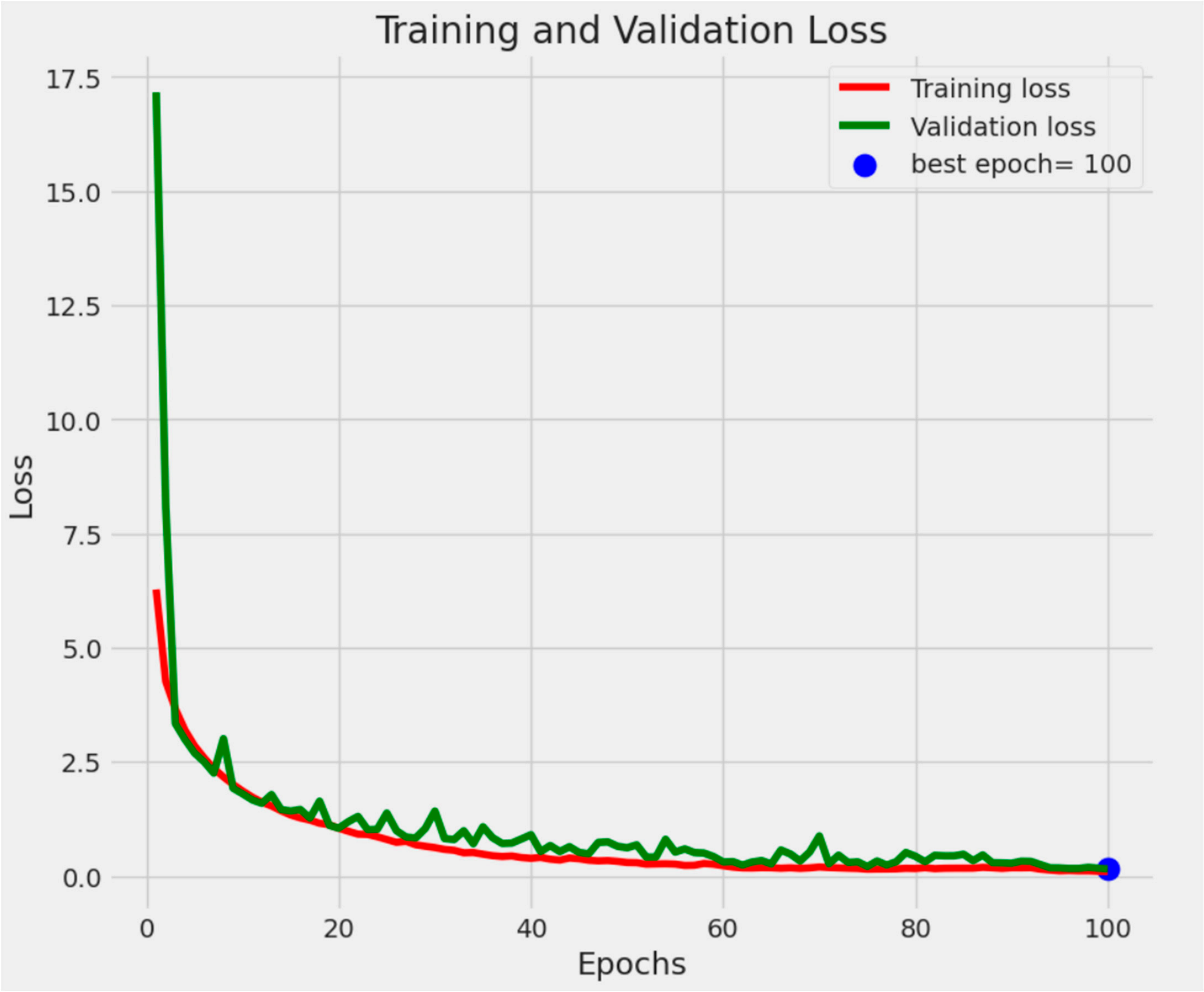

Training and Validation Loss
Training loss
Validation loss
best epoch= 100
Loss
Epochs
17.5
15.0
12.5
10.0
7.5
5.0
2.5
0.0
0
20
40
60
80
100

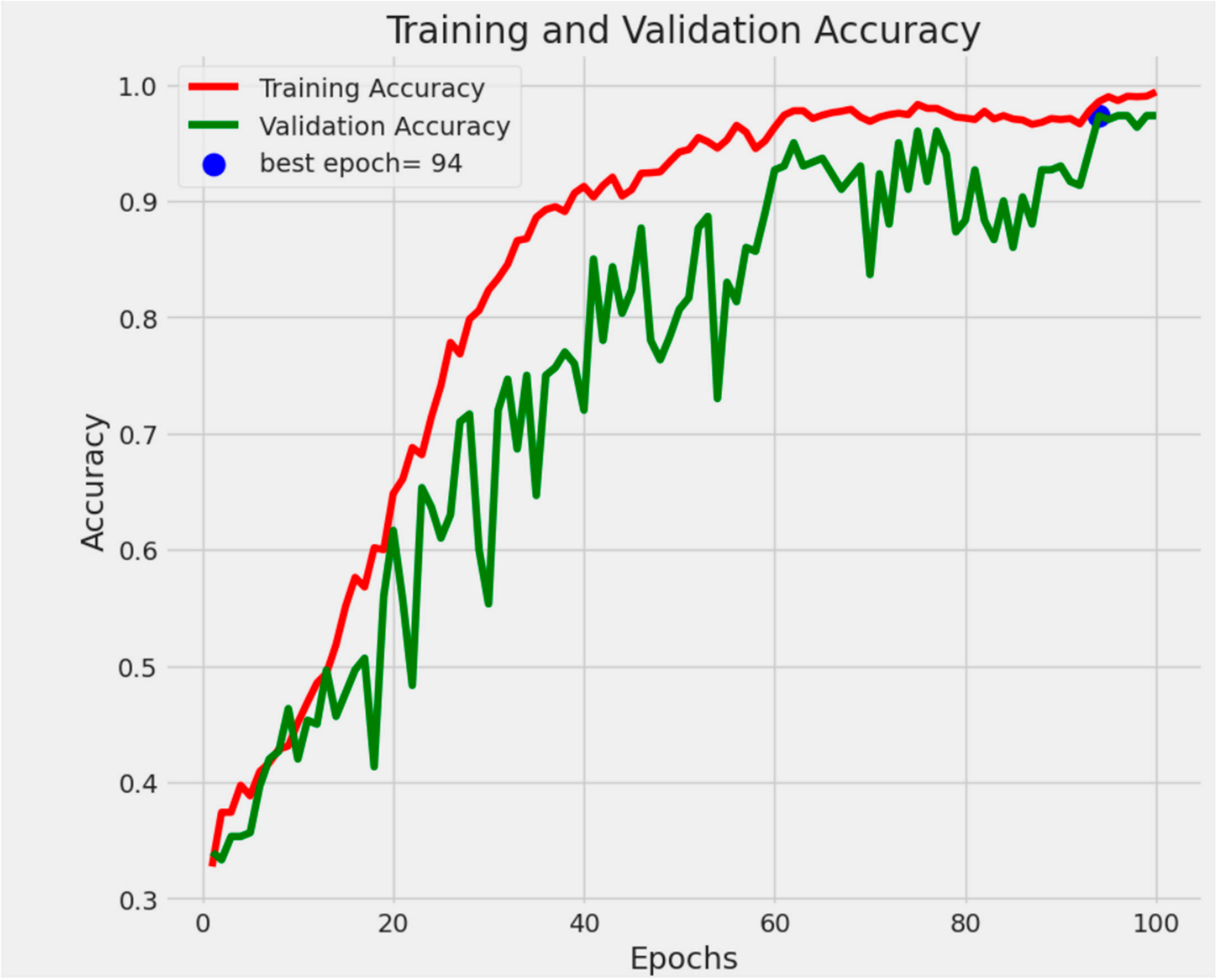

Training and Validation Accuracy
Training Accuracy
Validation Accuracy
best epoch= 94
Accuracy
Epochs
1.0
0.9
0.8
0.7
0.6
0.5
0.4
0.3
0
20
40
60
80
100

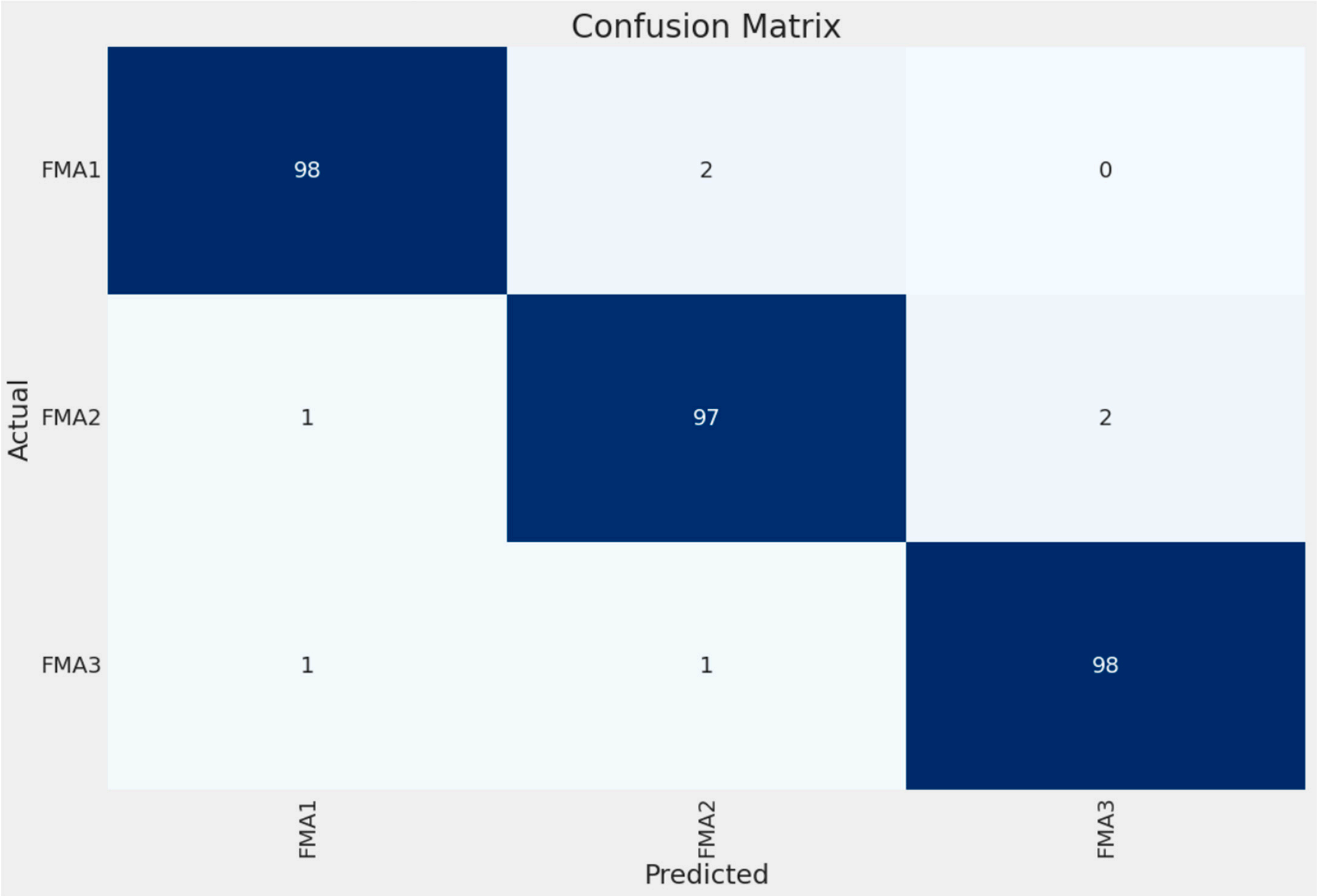


```
there were 7 errors in 300 tests for an accuracy of  97.67


Classification Report:
----------------------
              precision    recall  f1-score   support

        FMA1     0.9800    0.9800    0.9800       100
        FMA2     0.9700    0.9700    0.9700       100
        FMA3     0.9800    0.9800    0.9800       100

    accuracy                         0.9767       300
   macro avg     0.9767    0.9767    0.9767       300
weighted avg     0.9767    0.9767    0.9767       300
```

EFFICIENTNET B0

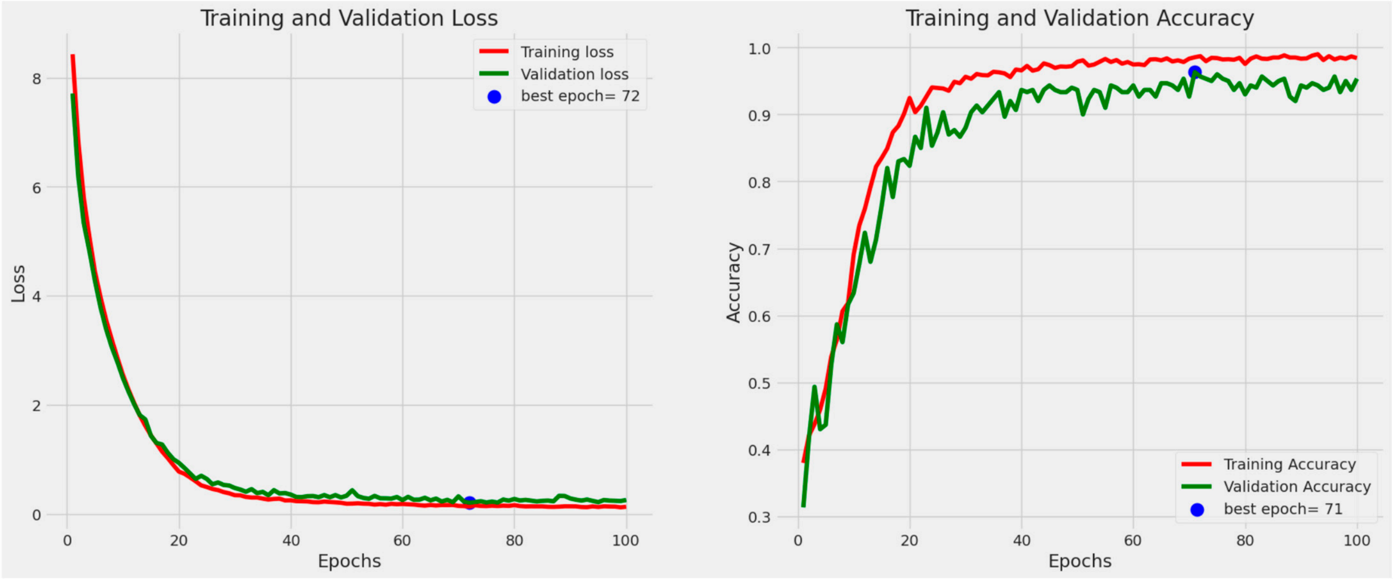

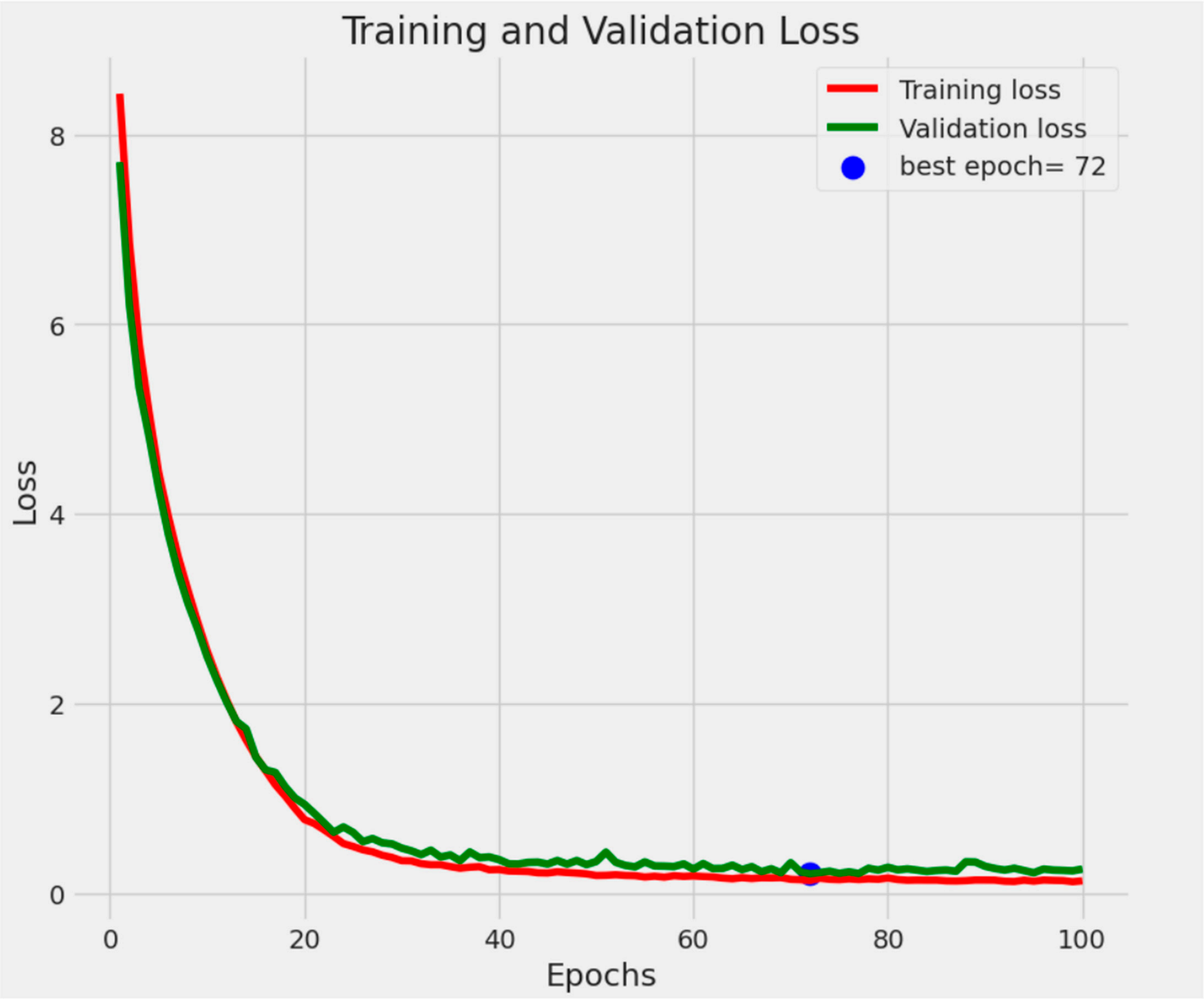

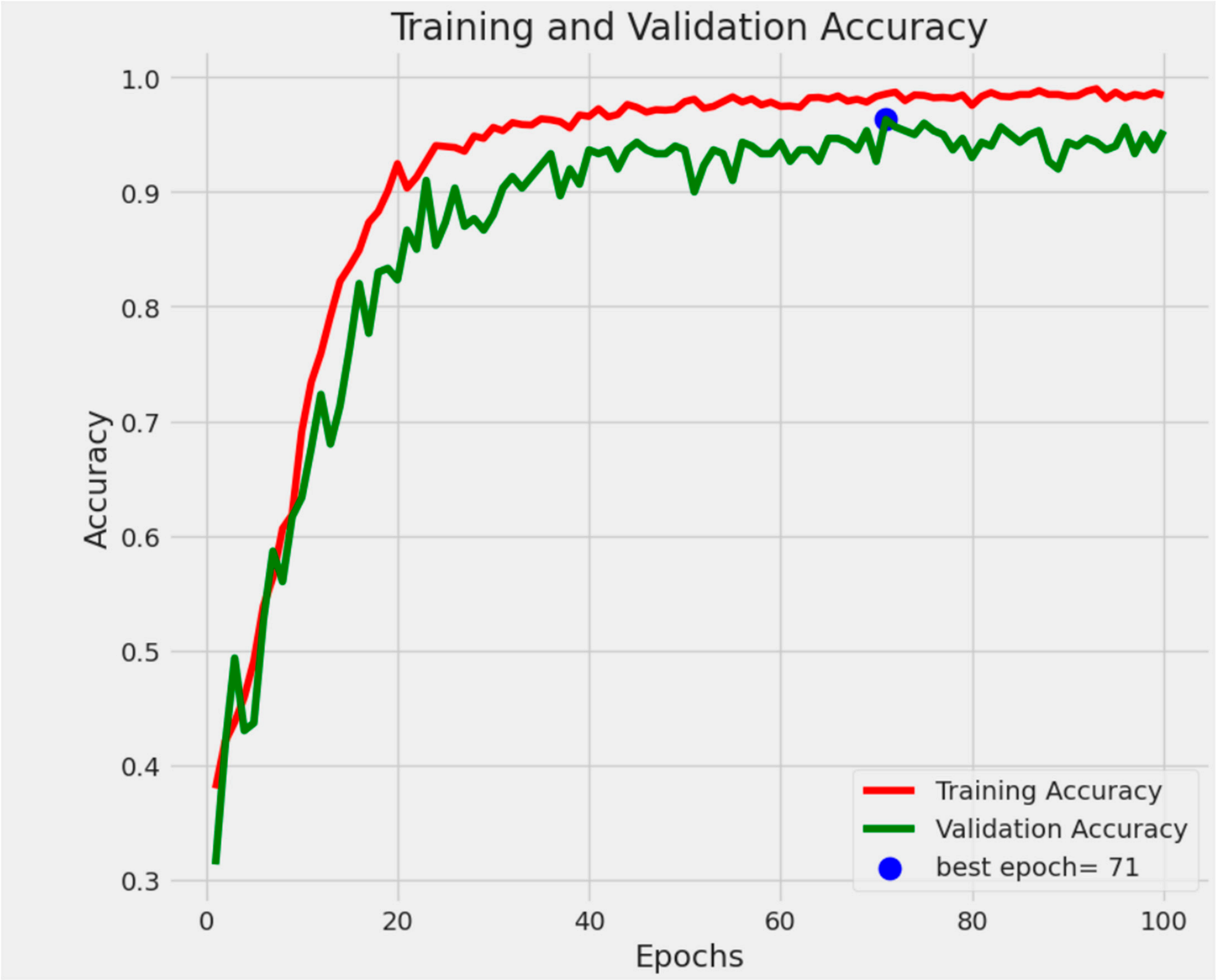
Training and Validation Accuracy
Accuracy
Epochs
1.0
0.9
0.8
0.7
0.6
0.5
0.4
0.3
0
20
40
60
80
100
Training Accuracy
Validation Accuracy
best epoch= 71

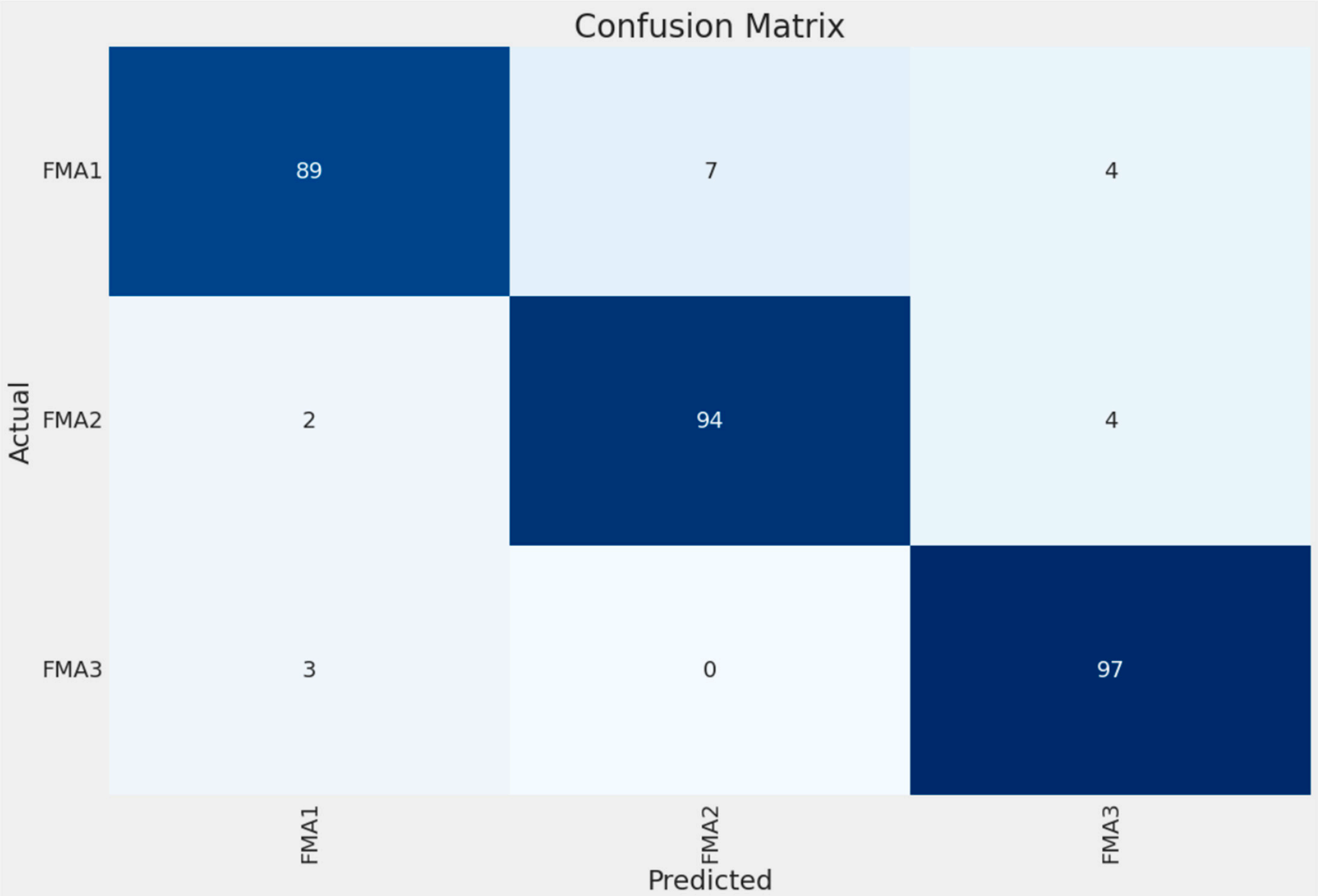

there were 20 errors in 300 tests for an accuracy of 93.33

Classification Report:
----

	precision	recall	f1-score	support
FMA1	0.9468	0.8900	0.9175	100
FMA2	0.9307	0.9400	0.9353	100
FMA3	0.9238	0.9700	0.9463	100
accuracy			0.9333	300
macro avg	0.9338	0.9333	0.9331	300
weighted avg	0.9338	0.9333	0.9331	300

XCEPTION: %33.67

VGG16 :%37

VGG19 :%32.67

NASNETMOBILE

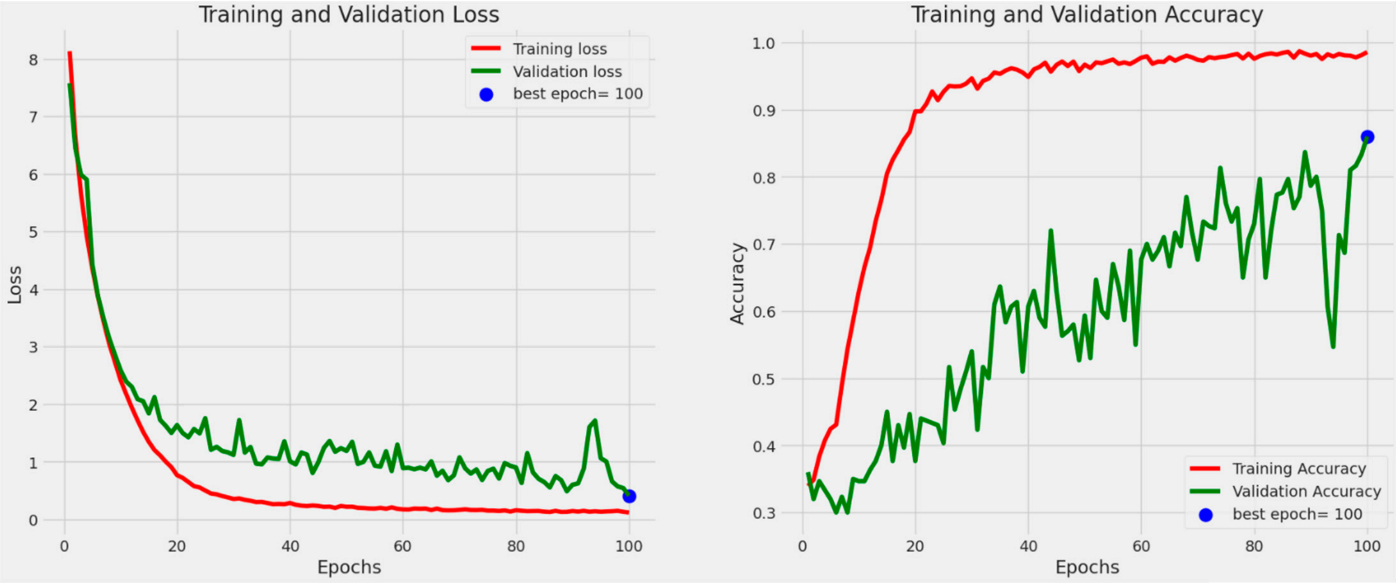

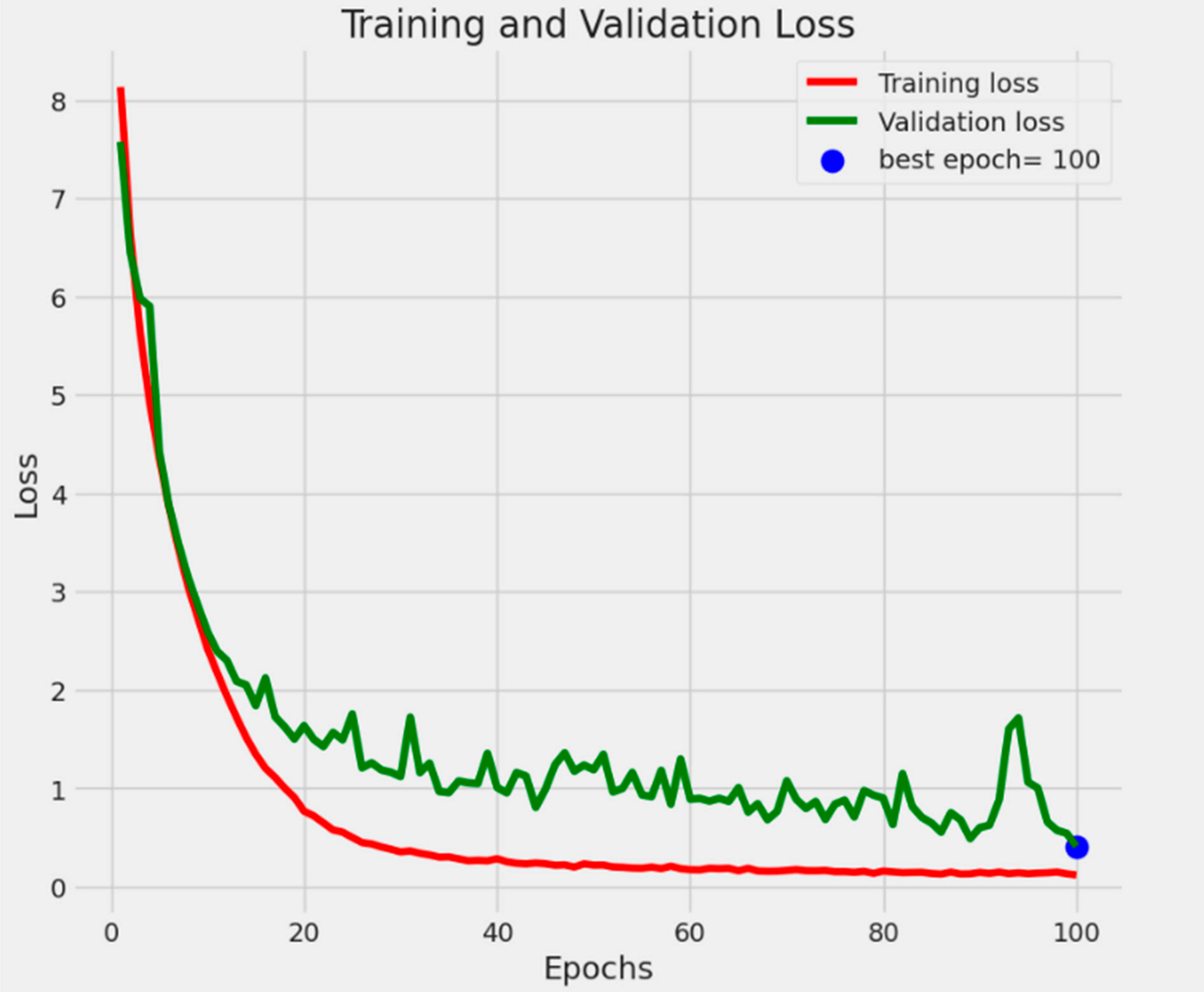

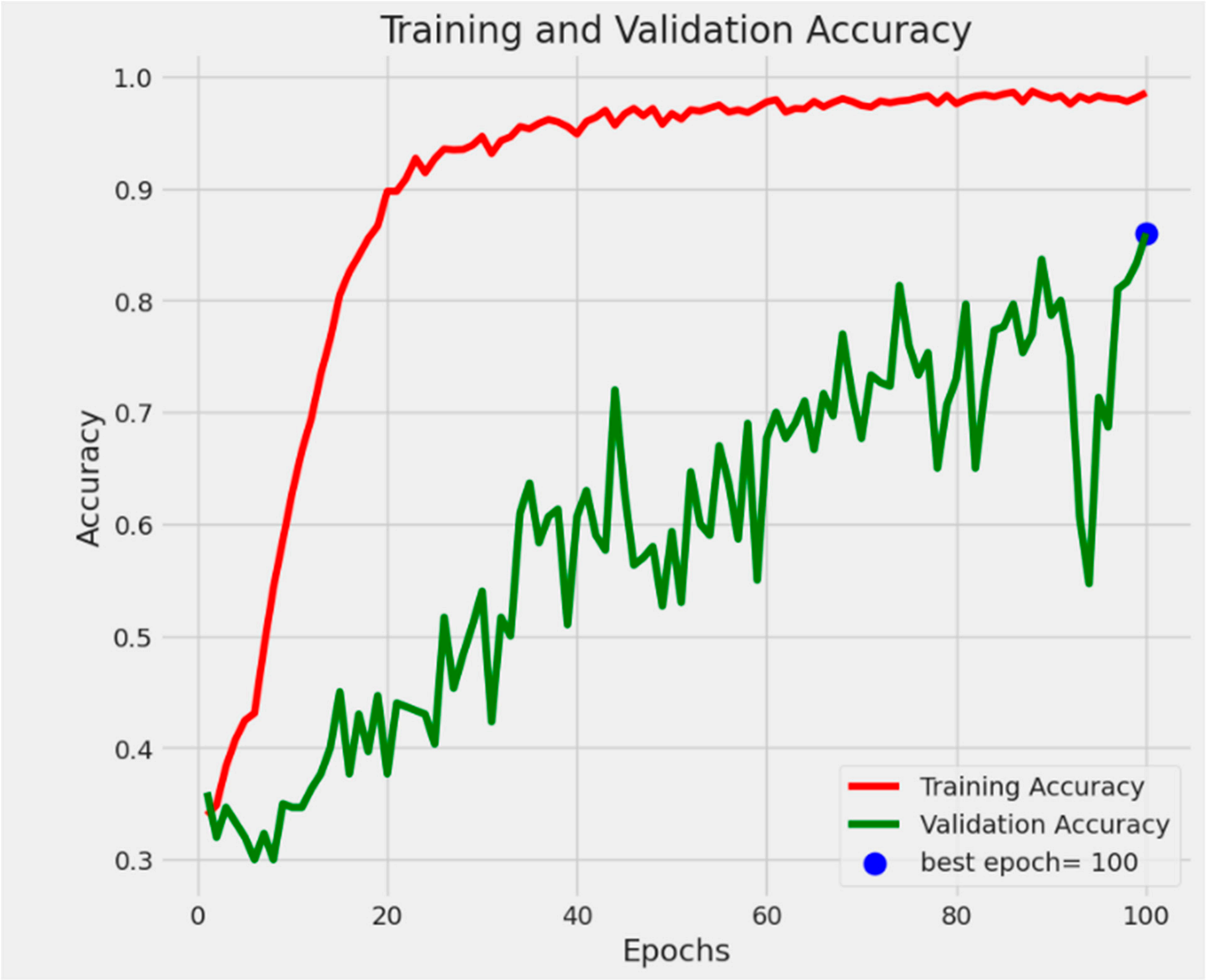

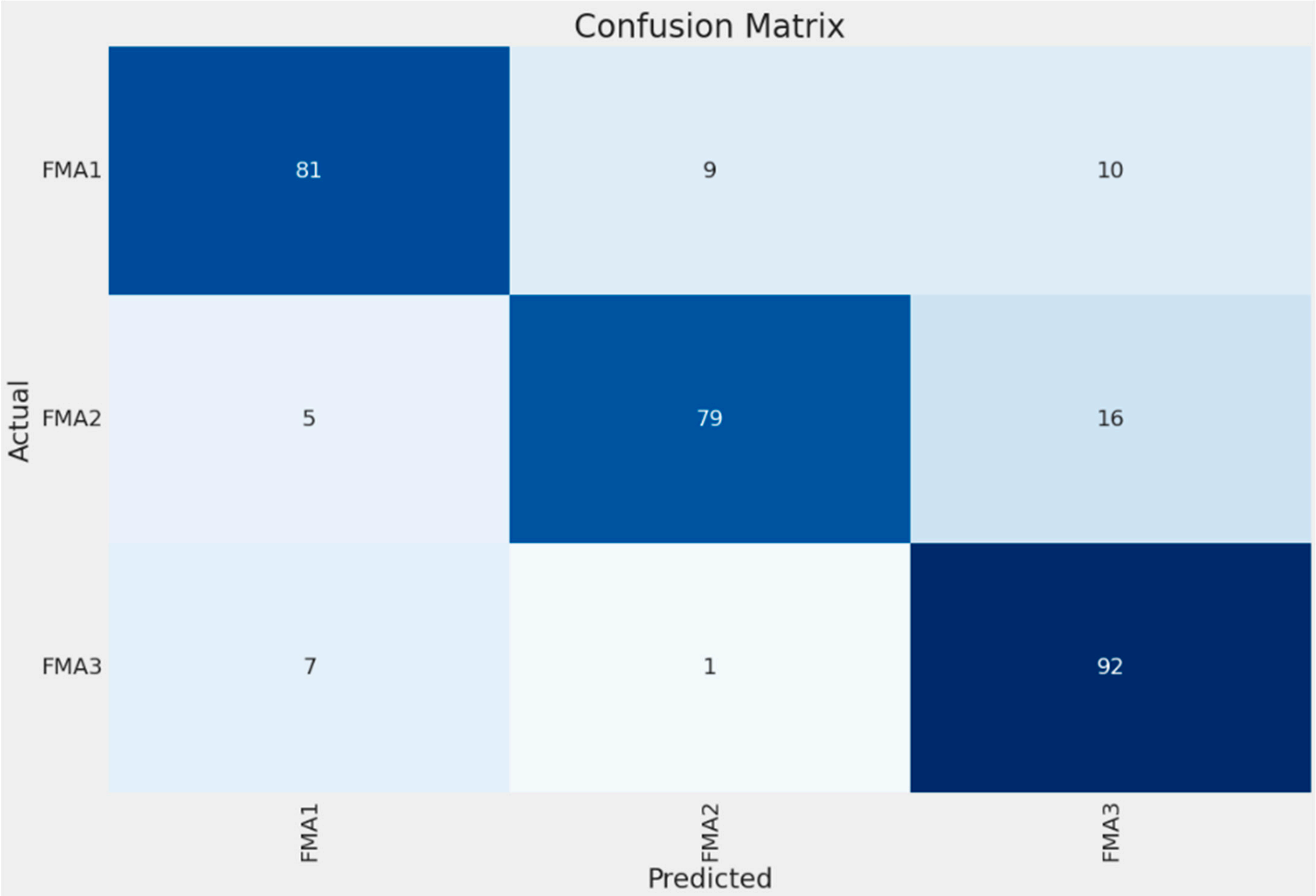

there were 48 errors in 300 tests for an accuracy of 84.00

Classification Report:
----

	precision	recall	f1-score	support
FMA1	0.8710	0.8100	0.8394	100
FMA2	0.8876	0.7900	0.8360	100
FMA3	0.7797	0.9200	0.8440	100
accuracy			0.8400	300
macro avg	0.8461	0.8400	0.8398	300
weighted avg	0.8461	0.8400	0.8398	300

RESNET101 :%34.33

RESNET152 :%33.37

RESNET50

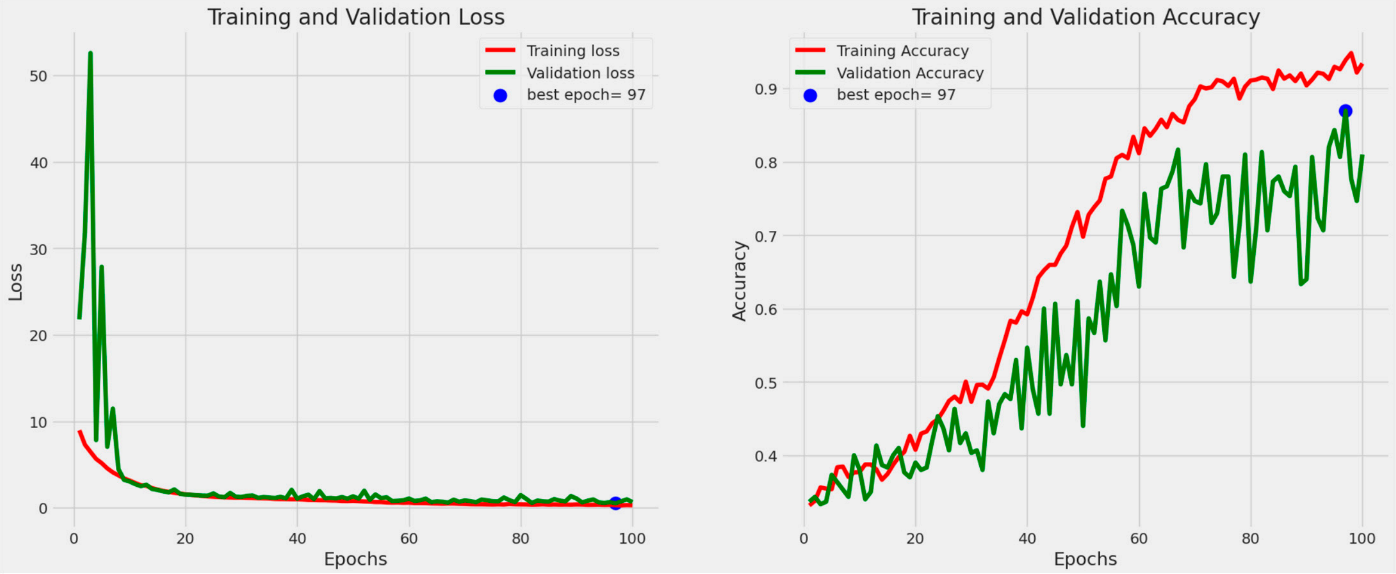

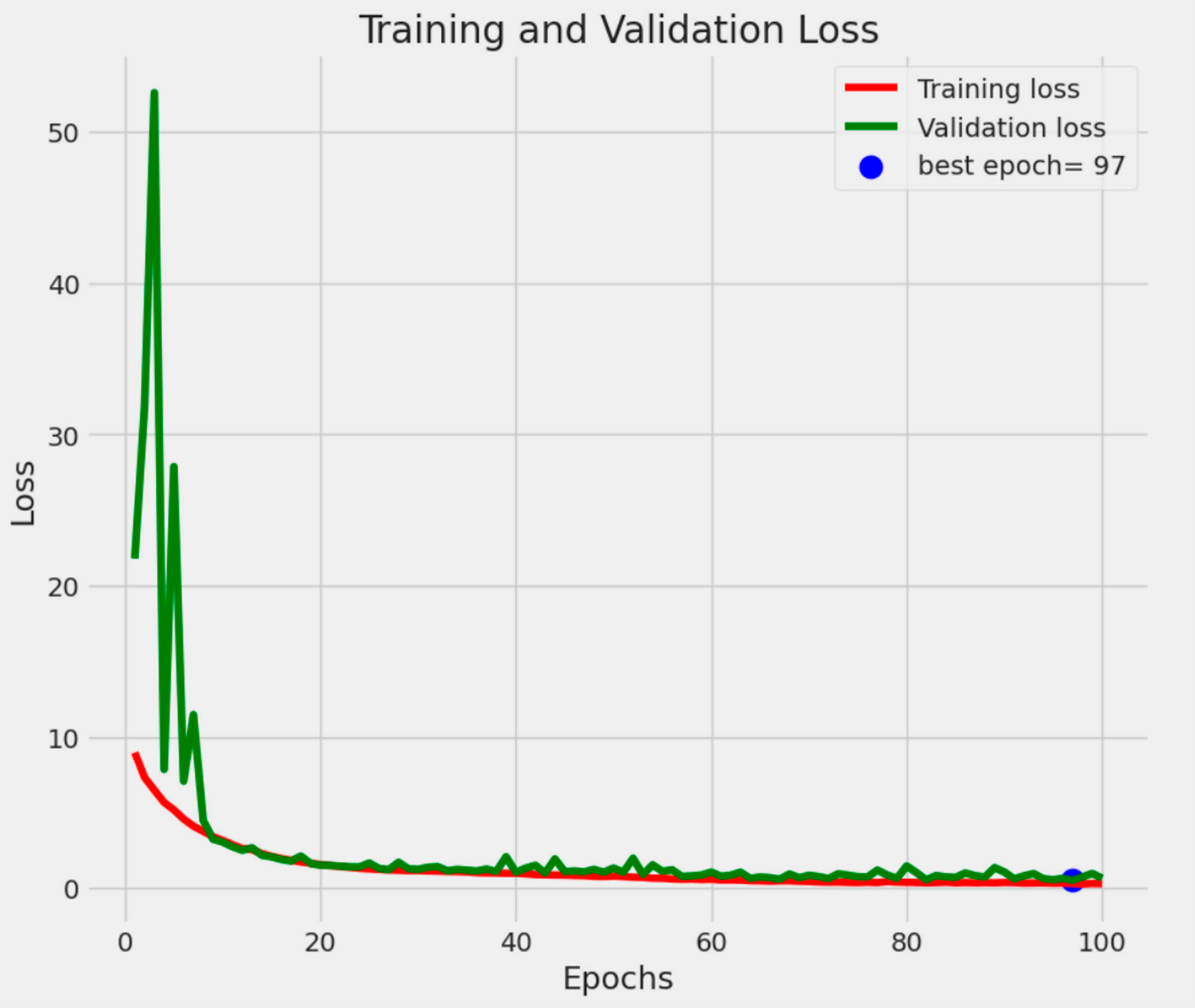

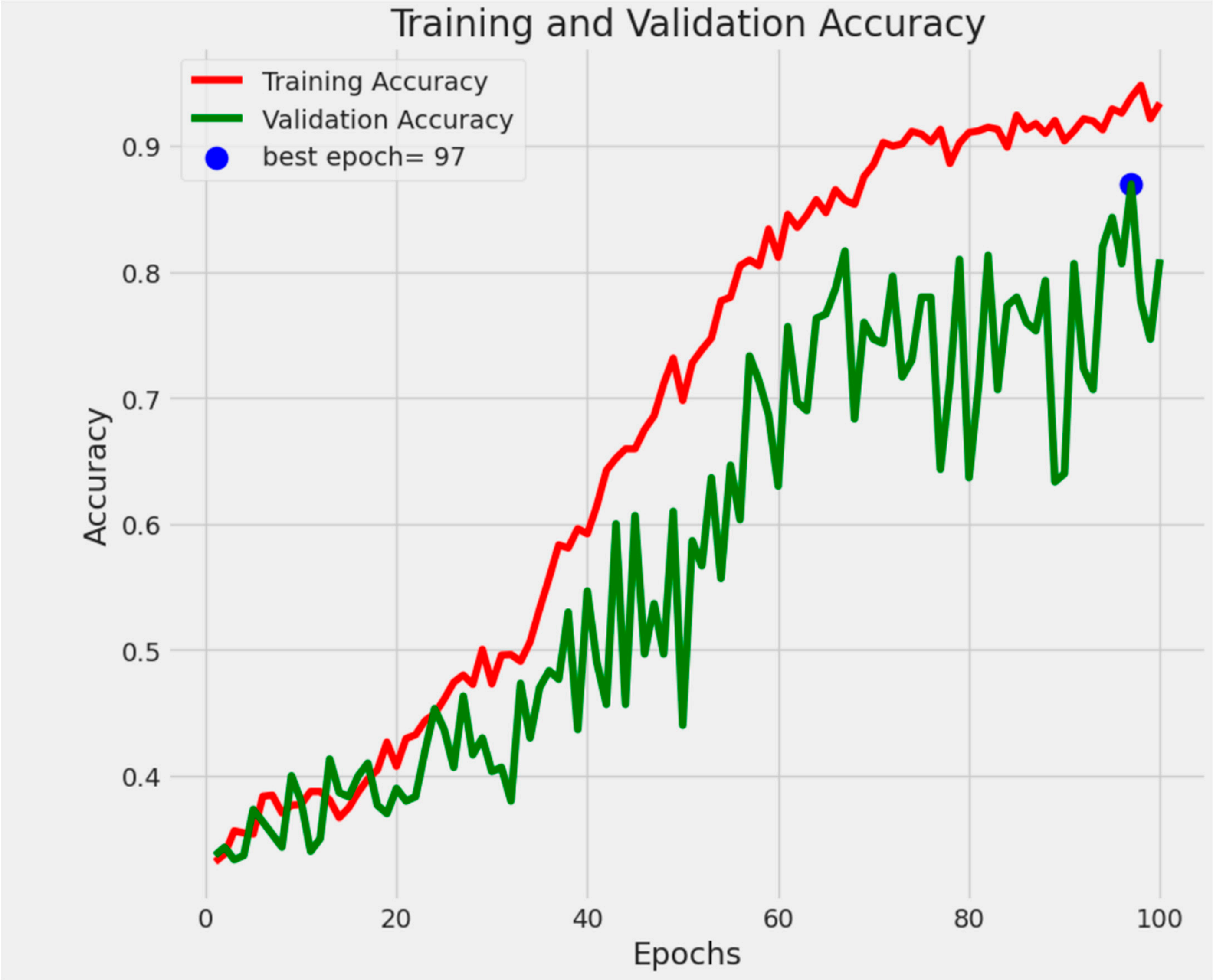

Training and Validation Accuracy
Training Accuracy
Validation Accuracy
best epoch= 97
Accuracy
0.4
0.5
0.6
0.7
0.8
0.9
0
20
40
60
80
100
Epochs

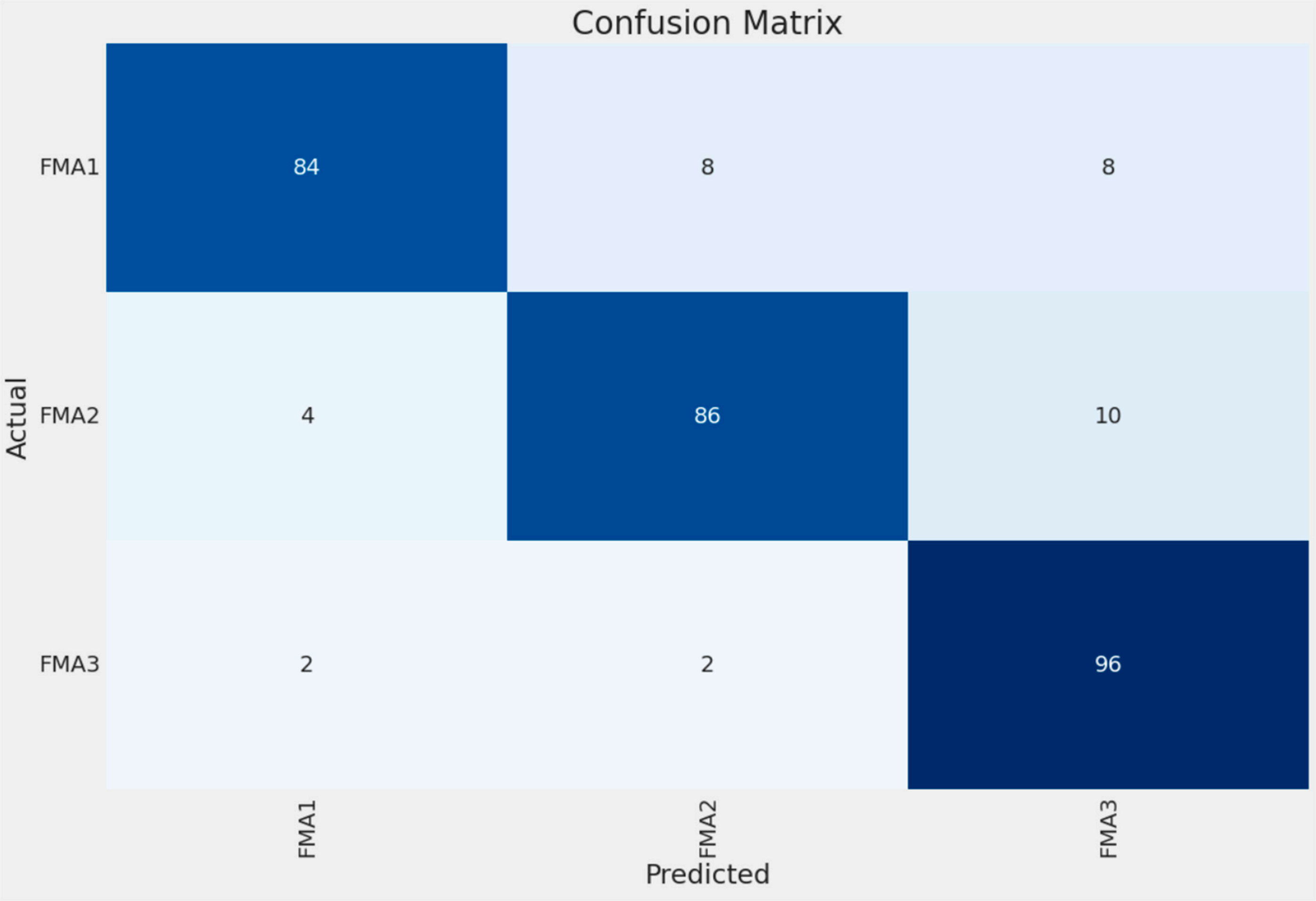

there were 34 errors in 300 tests for an accuracy of 88.67

Classification Report:
----

	precision	recall	f1-score	support
FMA1	0.9333	0.8400	0.8842	100
FMA2	0.8958	0.8600	0.8776	100
FMA3	0.8421	0.9600	0.8972	100
accuracy			0.8867	300
macro avg	0.8904	0.8867	0.8863	300
weighted avg	0.8904	0.8867	0.8863	300

EFFICIENTNET V2

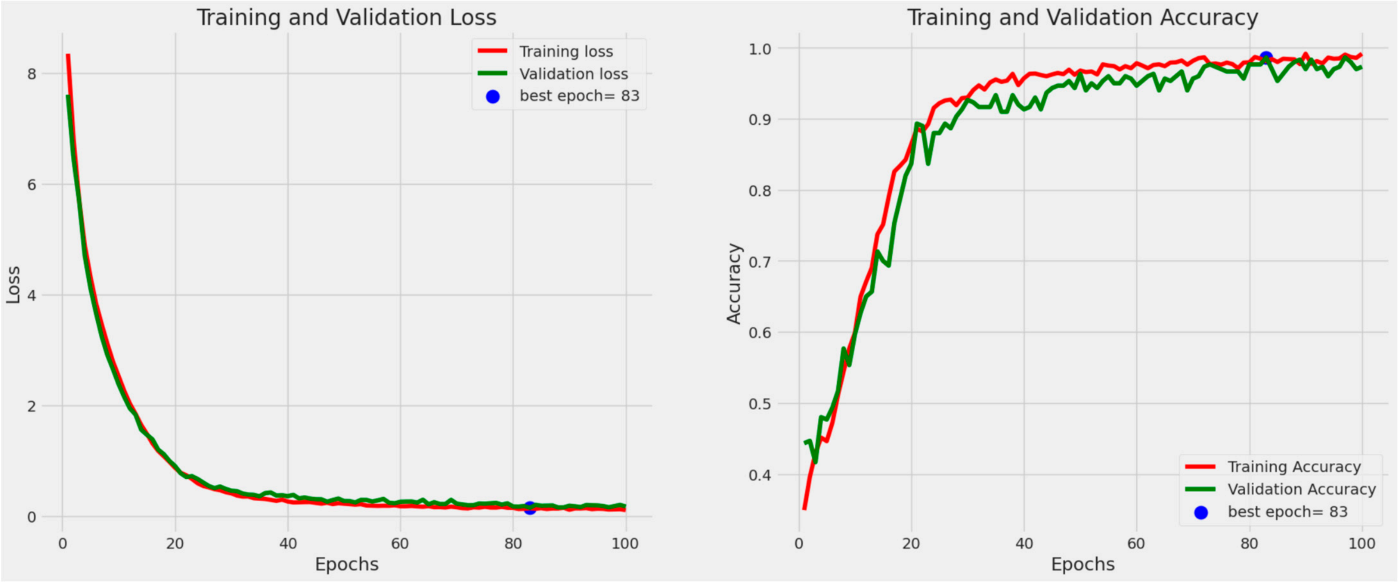

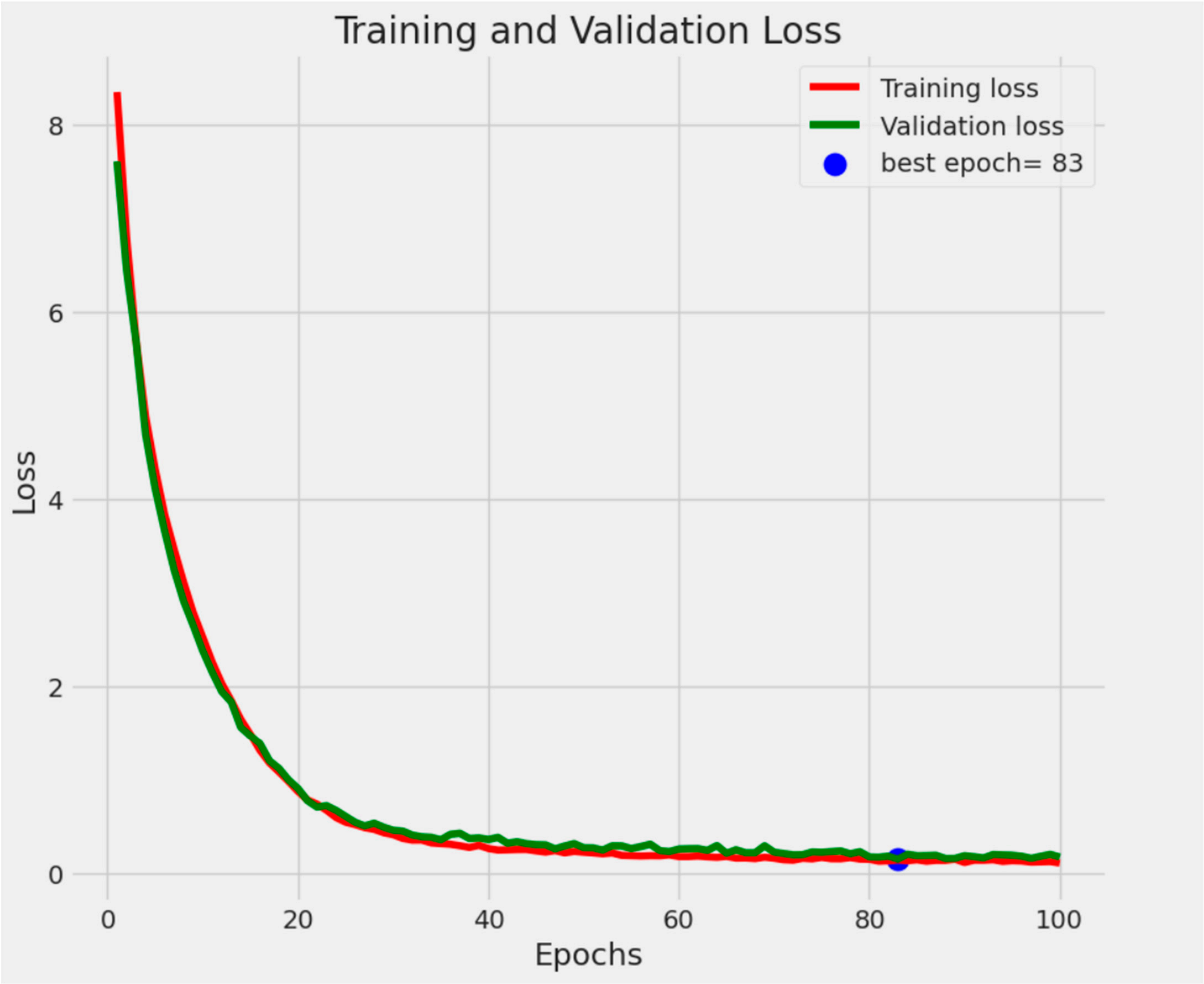

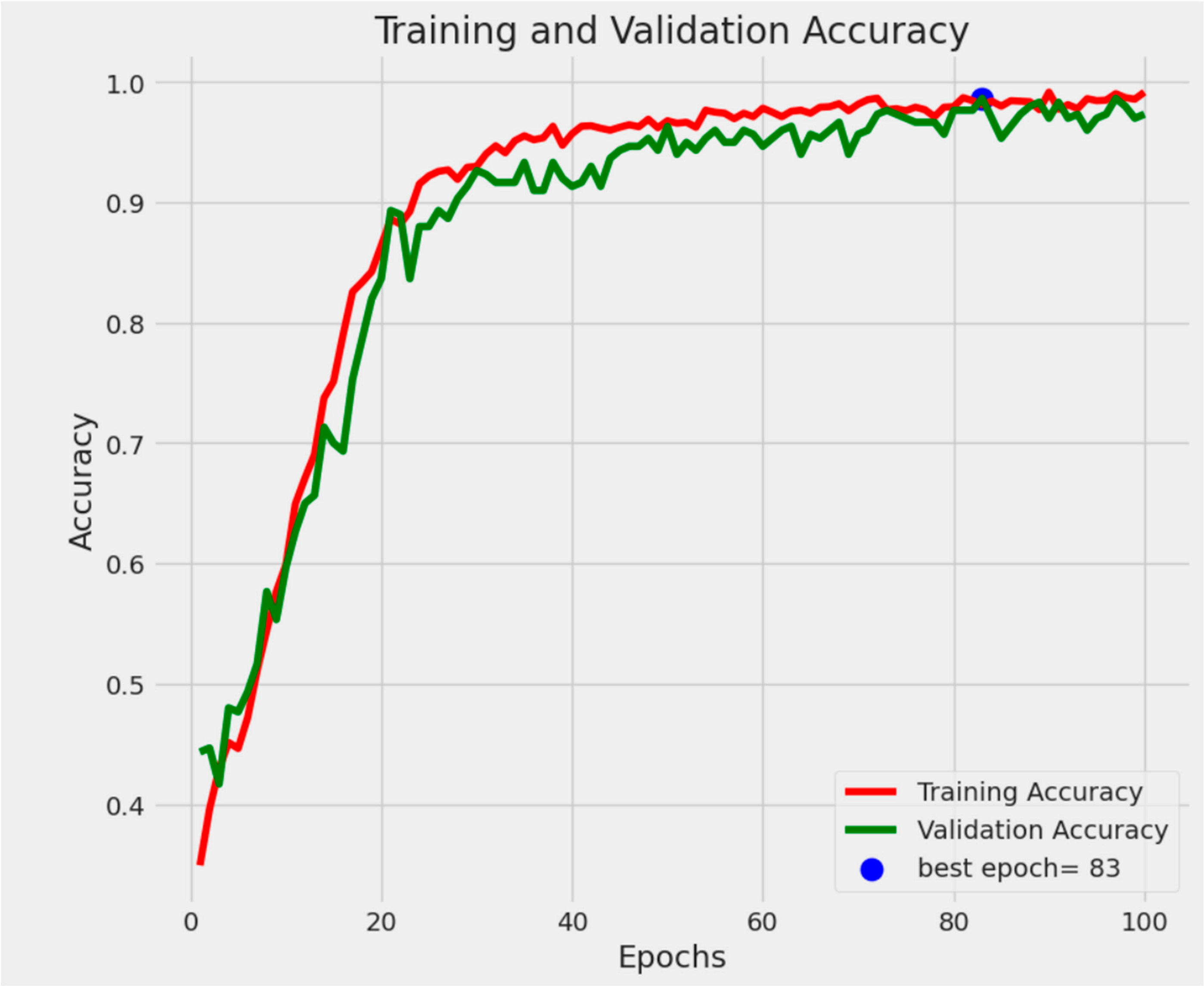
Training and Validation Accuracy
1.0
0.9
0.8
0.7
0.6
0.5
0.4
Accuracy
0
20
40
60
80
100
Epochs
Training Accuracy
Validation Accuracy
best epoch= 83

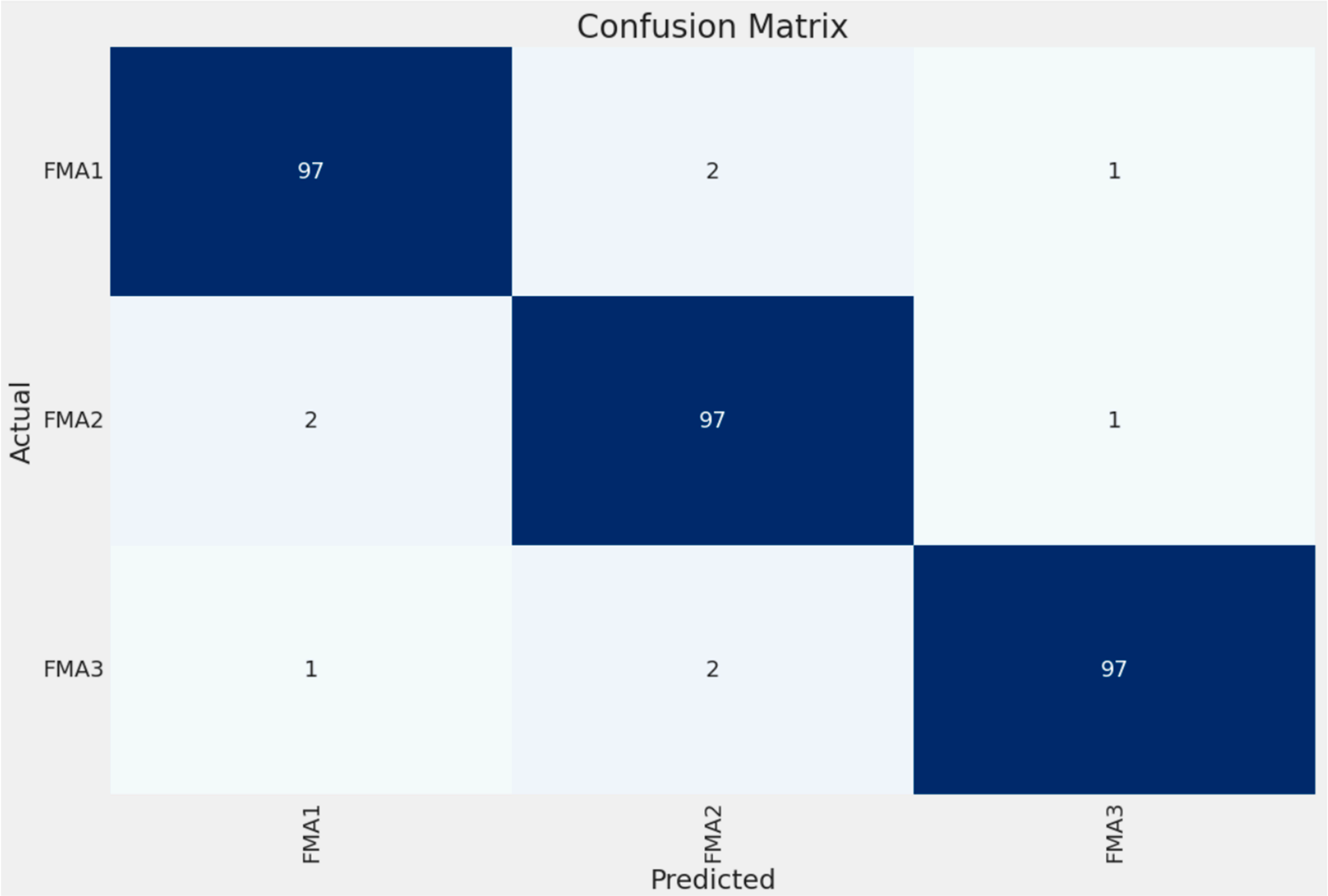

there were 9 errors in 300 tests for an accuracy of 97.00

Classification Report:
----

	precision	recall	f1-score	support
FMA1	0.9700	0.9700	0.9700	100
FMA2	0.9604	0.9700	0.9652	100
FMA3	0.9798	0.9700	0.9749	100
accuracy			0.9700	300
macro avg	0.9701	0.9700	0.9700	300
weighted avg	0.9701	0.9700	0.9700	300
