# Supplementary material for: Profile Photograph Classification Performance of Deep Learning Algorithms Trained Using Cephalometric Measurements: A Preliminary Study
Source: Diagnostics (Basel). 2024 Aug 30;14(17):1916. doi: 10.3390/diagnostics14171916 (PMC11394270; doi:10.3390/diagnostics14171916)
Supplement: Supplementary file 1 [file diagnostics-14-01916-s001.zip › KONVEKS.pdf]

MOBILENET V2

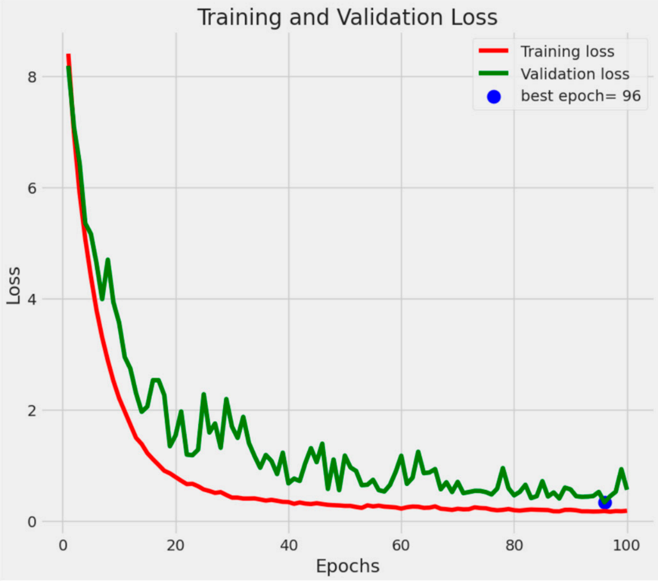

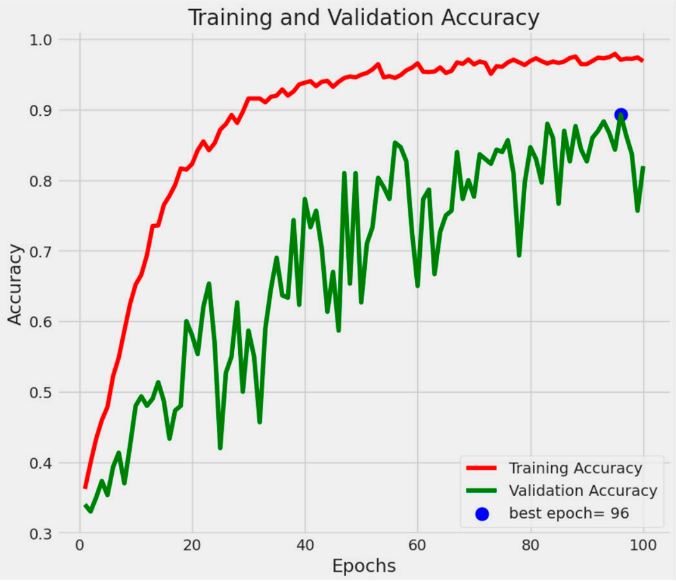

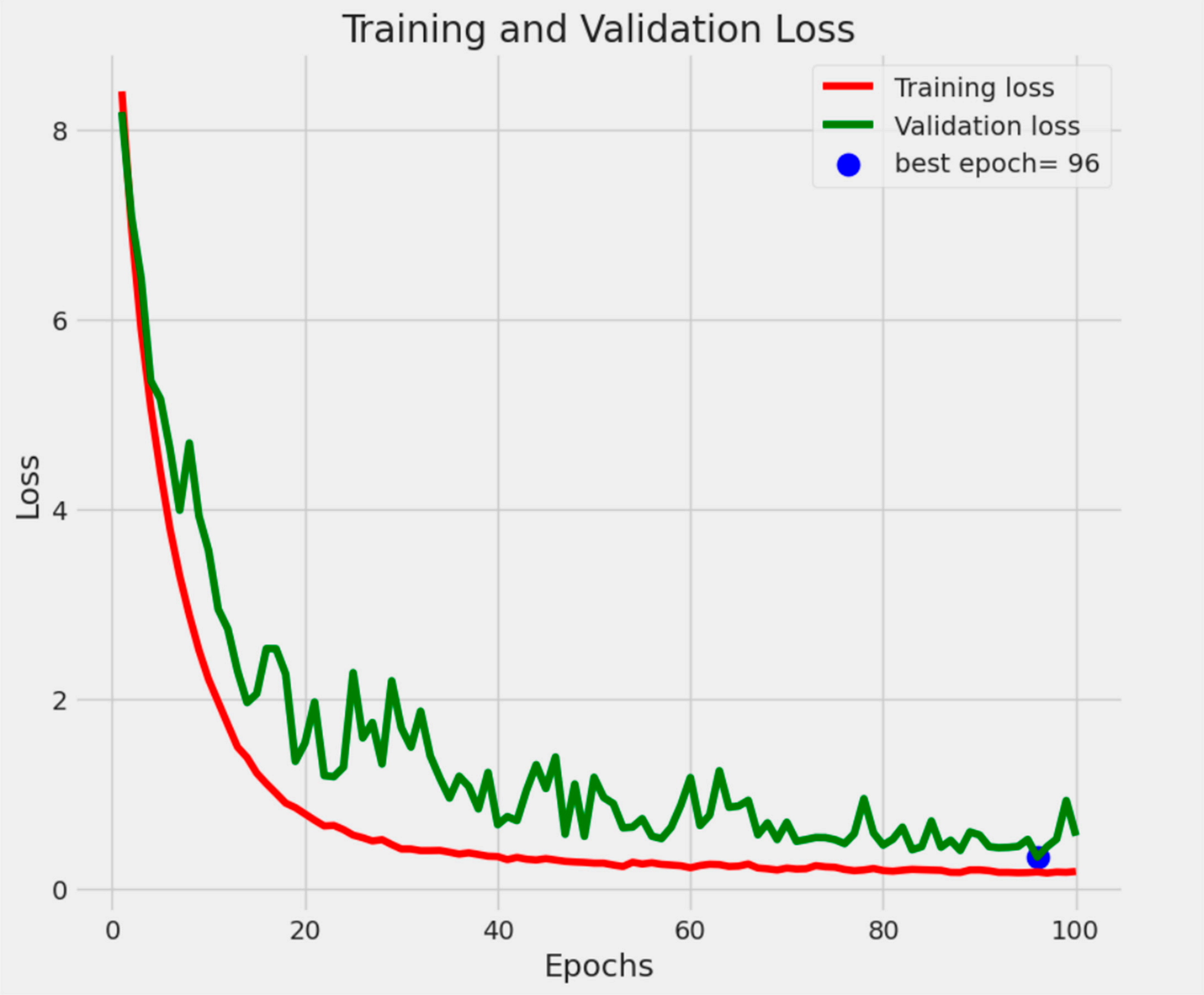

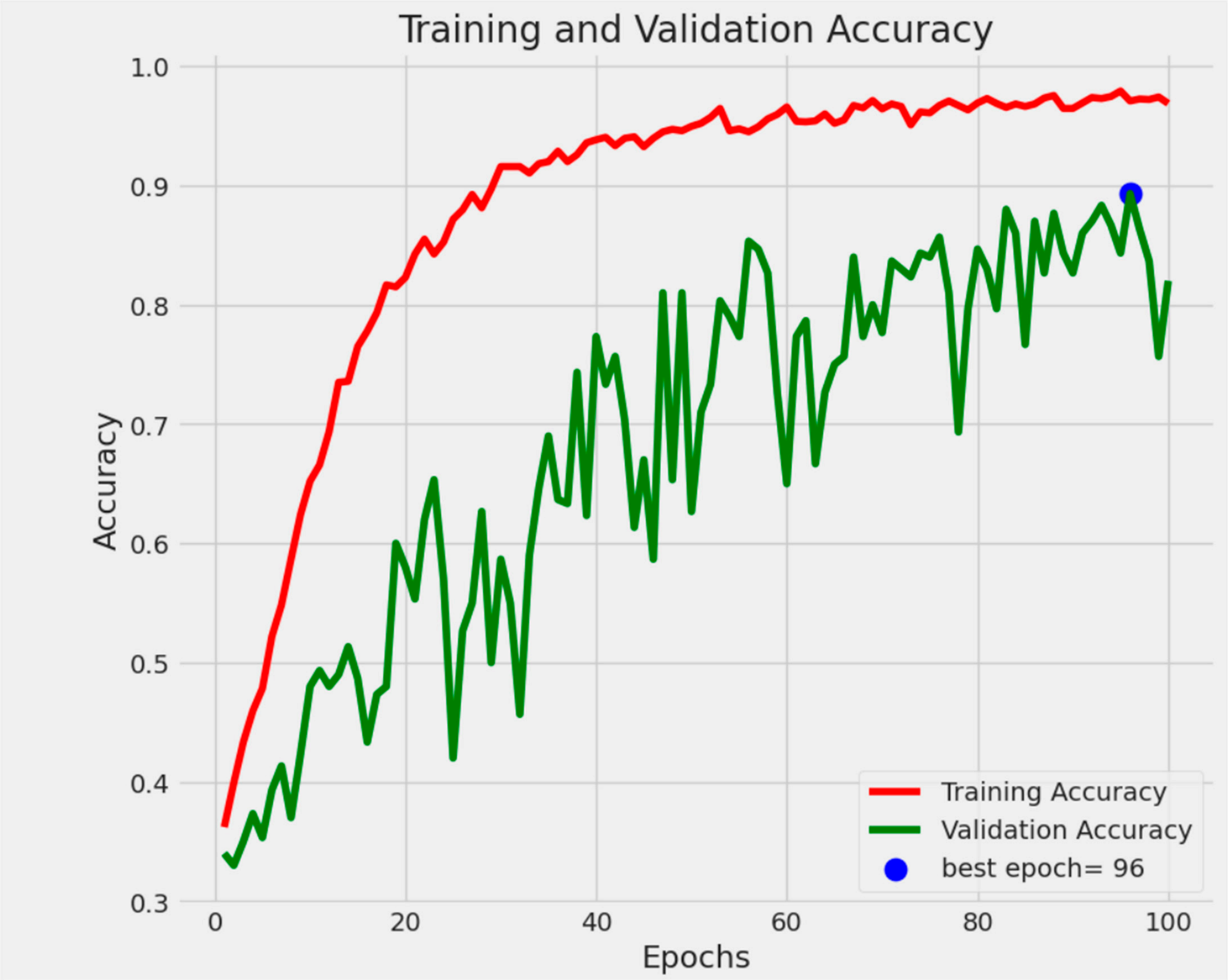

Training and Validation Accuracy
1.0
0.9
0.8
0.7
0.6
0.5
0.4
0.3
Accuracy
0
20
40
60
80
100
Epochs
Training Accuracy
Validation Accuracy
best epoch= 96

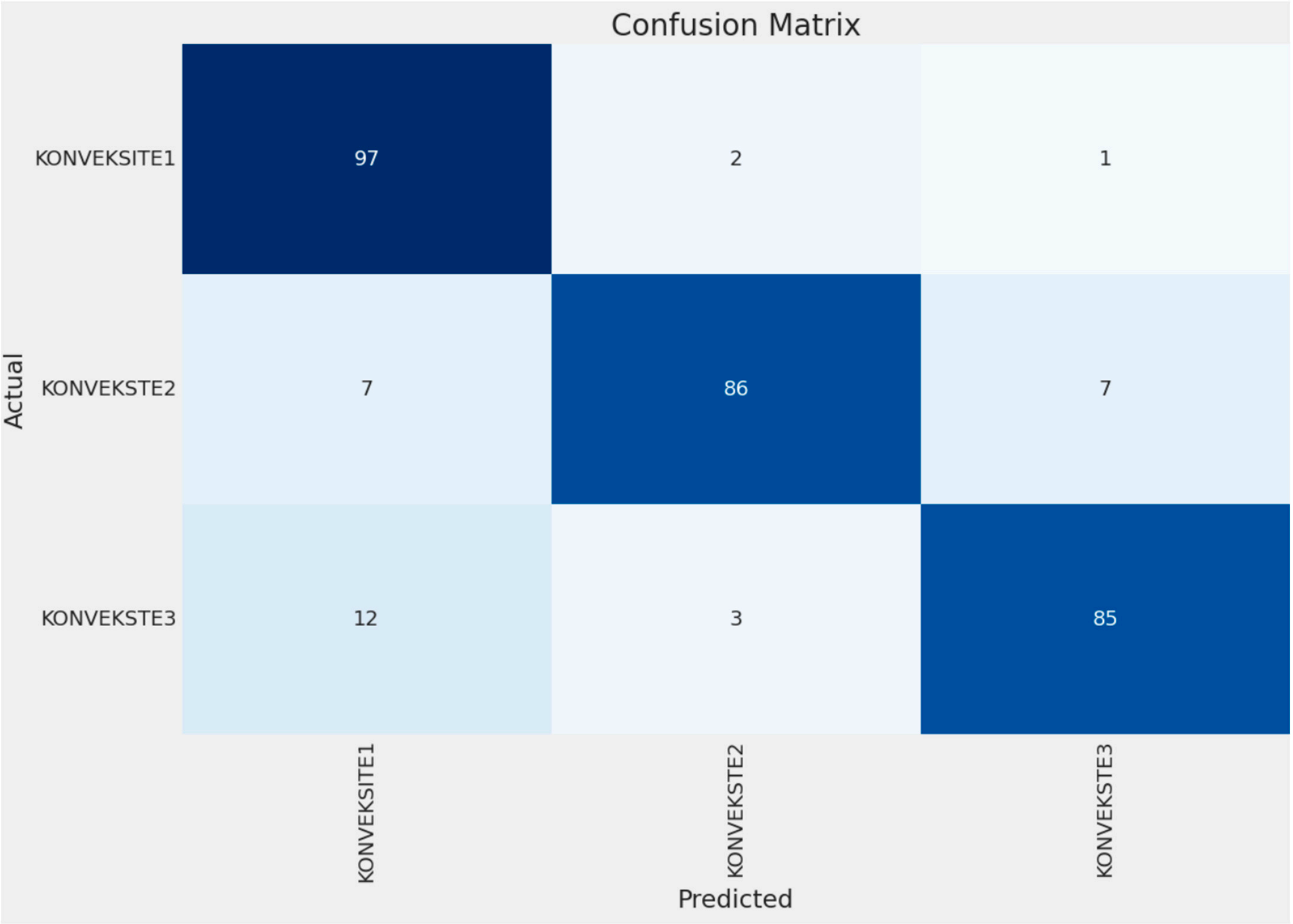

there were 32 errors in 300 tests for an accuracy of 89.33

Classification Report:
----

	precision	recall	f1-score	support
KONVEKSITE1	0.8362	0.9700	0.8981	100
KONVEKSTE2	0.9451	0.8600	0.9005	100
KONVEKSTE3	0.9140	0.8500	0.8808	100
accuracy			0.8933	300
macro avg	0.8984	0.8933	0.8932	300
weighted avg	0.8984	0.8933	0.8932	300

INCEPTION V3

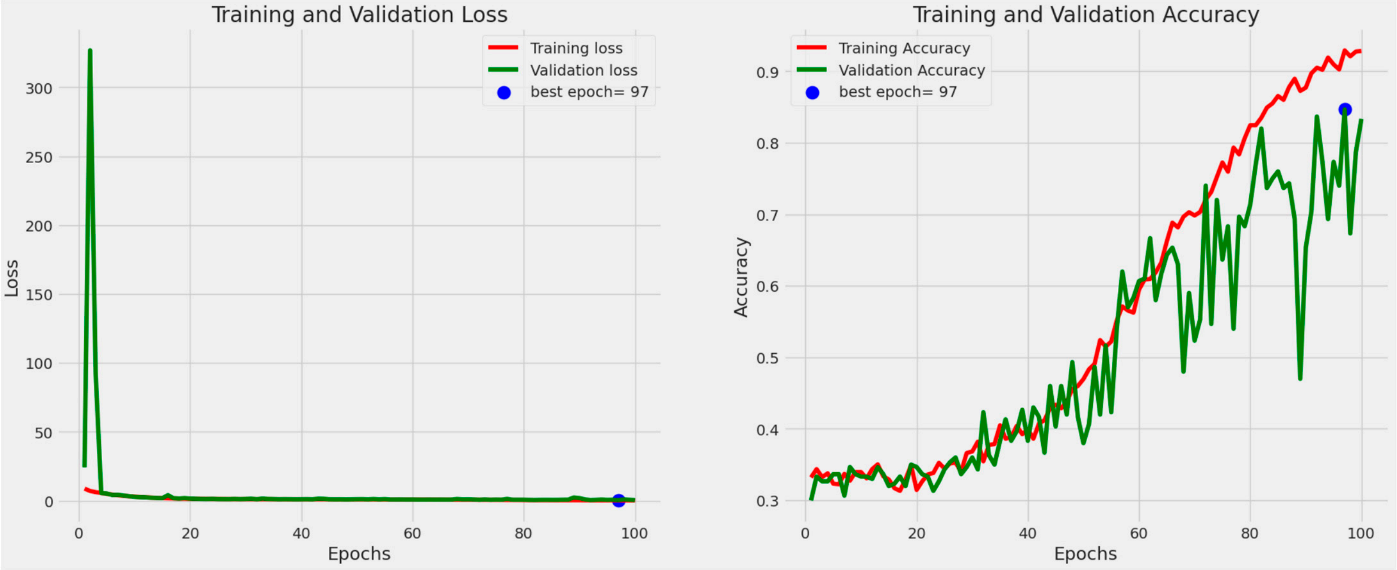

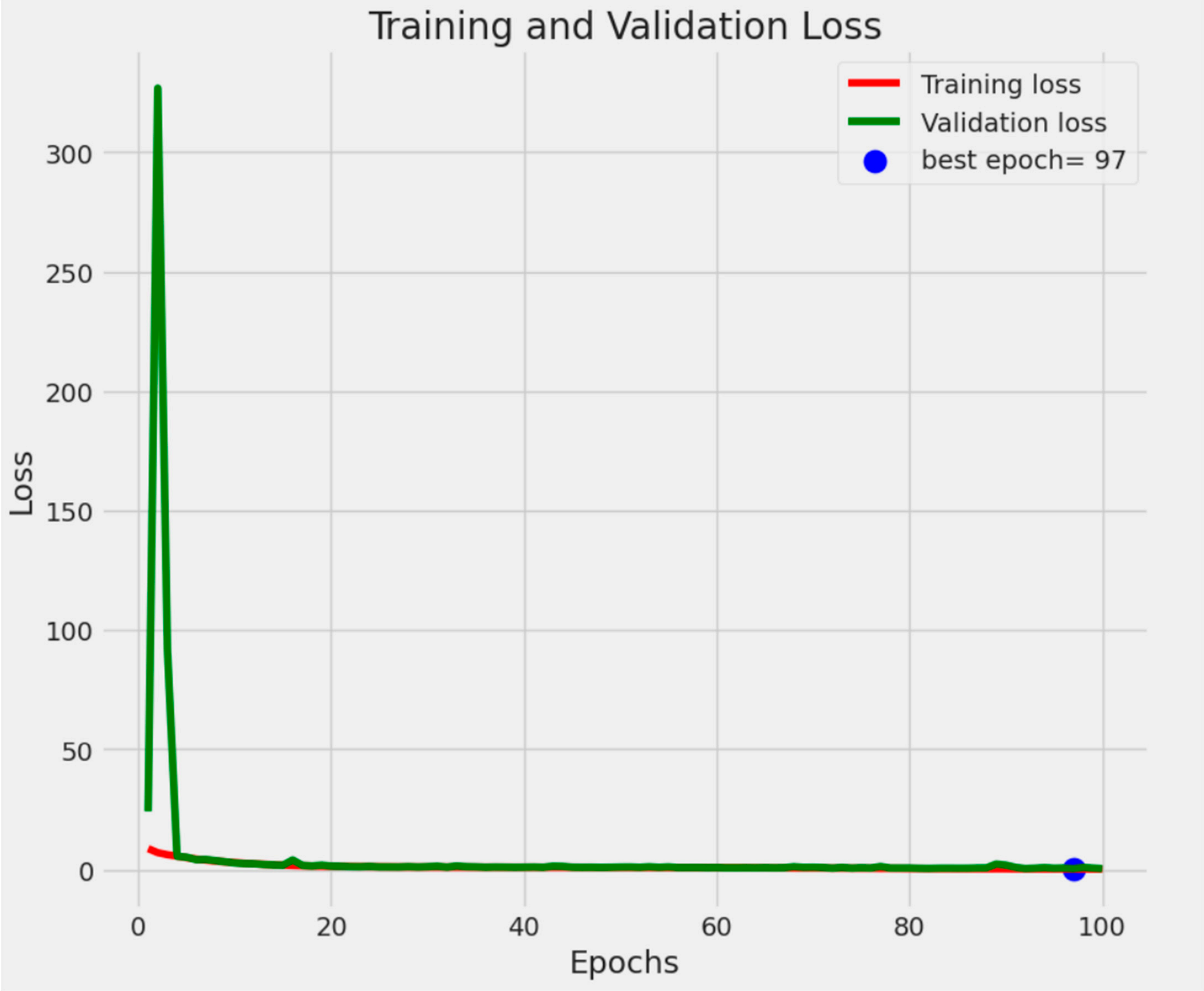

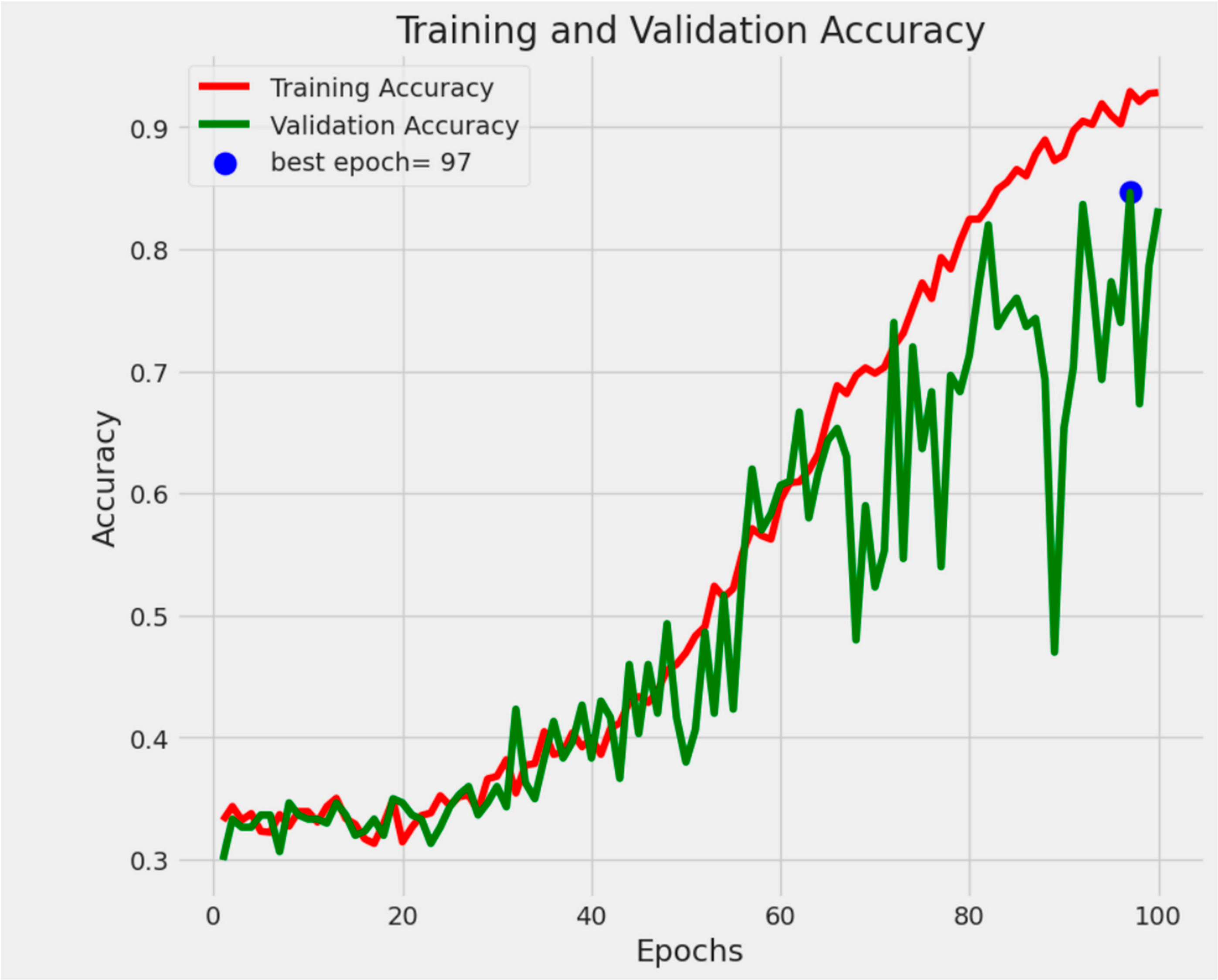

Training and Validation Accuracy
Training Accuracy
Validation Accuracy
best epoch= 97
0.9
0.8
0.7
0.6
0.5
0.4
0.3
Accuracy
0
20
40
60
80
100
Epochs

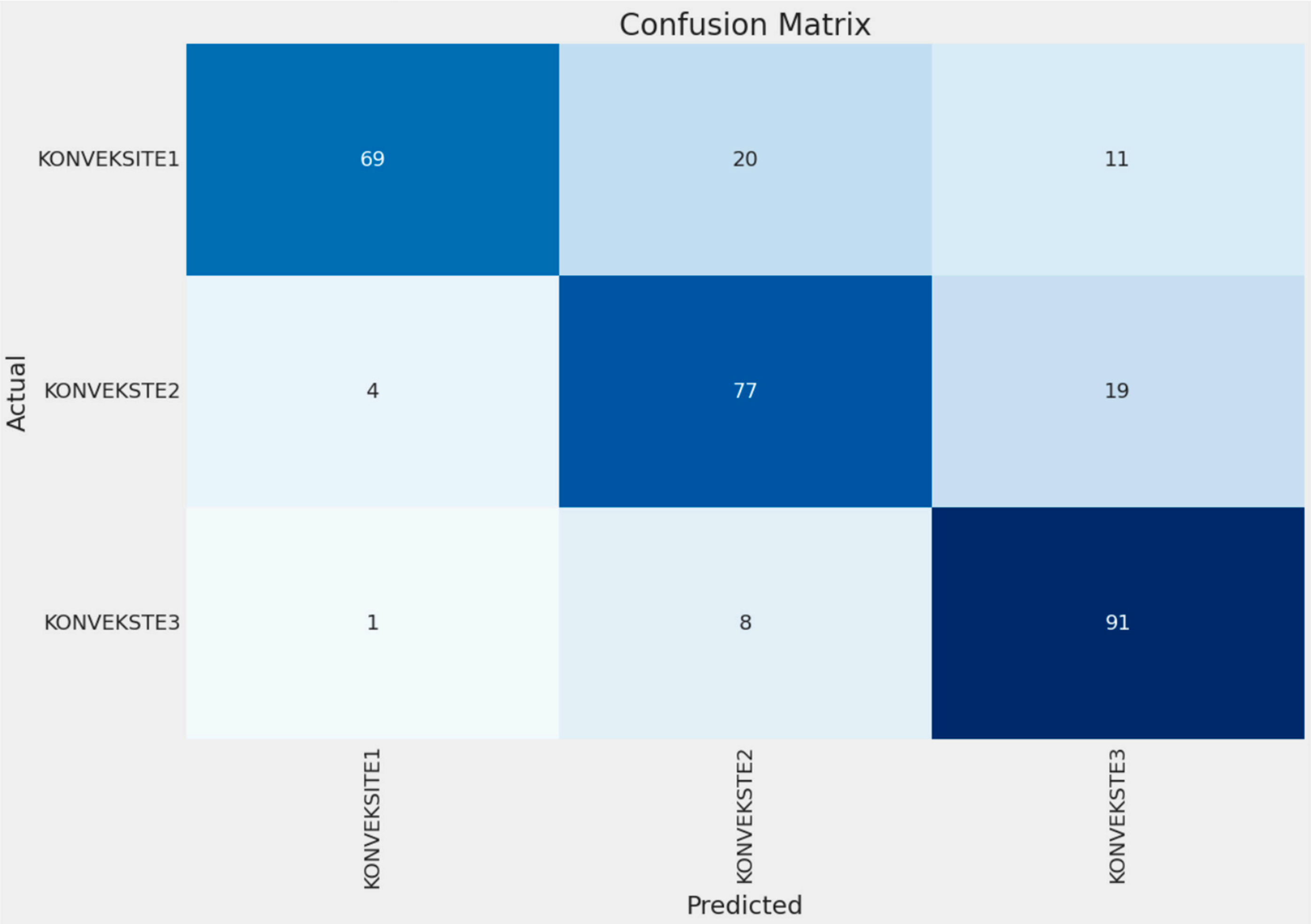

there were 63 errors in 300 tests for an accuracy of 79.00

Classification Report:

----

	precision	recall	f1-score	support
KONVEKSITE1	0.9324	0.6900	0.7931	100
KONVEKSTE2	0.7333	0.7700	0.7512	100
KONVEKSTE3	0.7521	0.9100	0.8235	100
accuracy			0.7900	300
macro avg	0.8059	0.7900	0.7893	300
weighted avg	0.8059	0.7900	0.7893	300

DENSENET 121

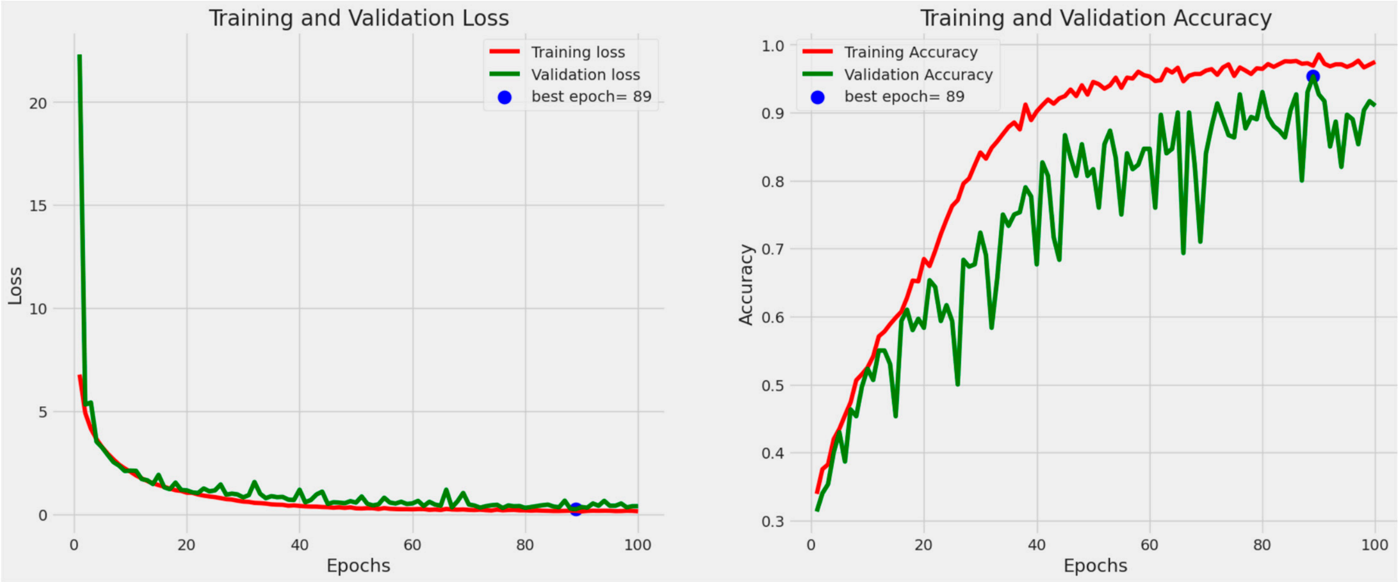

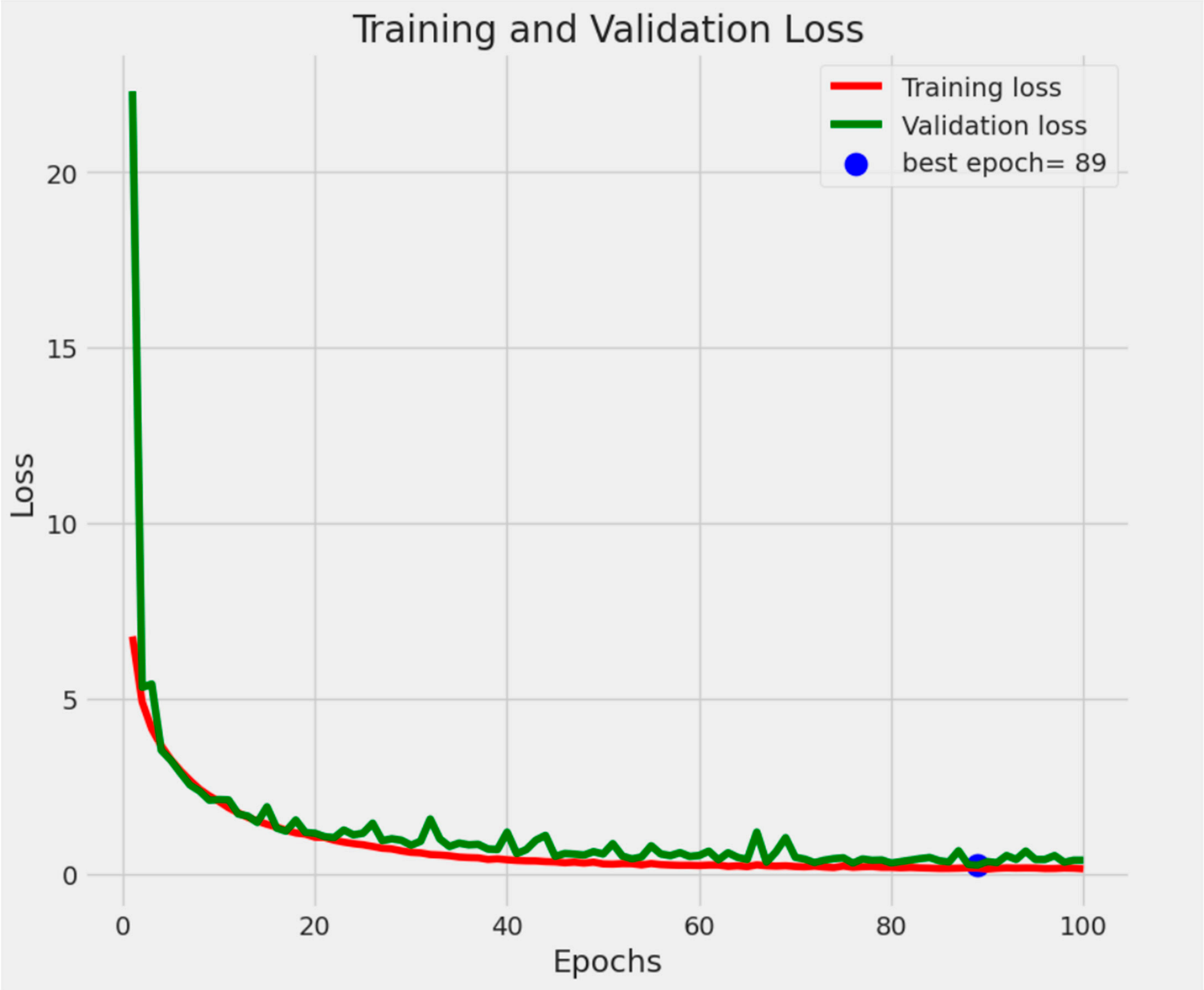

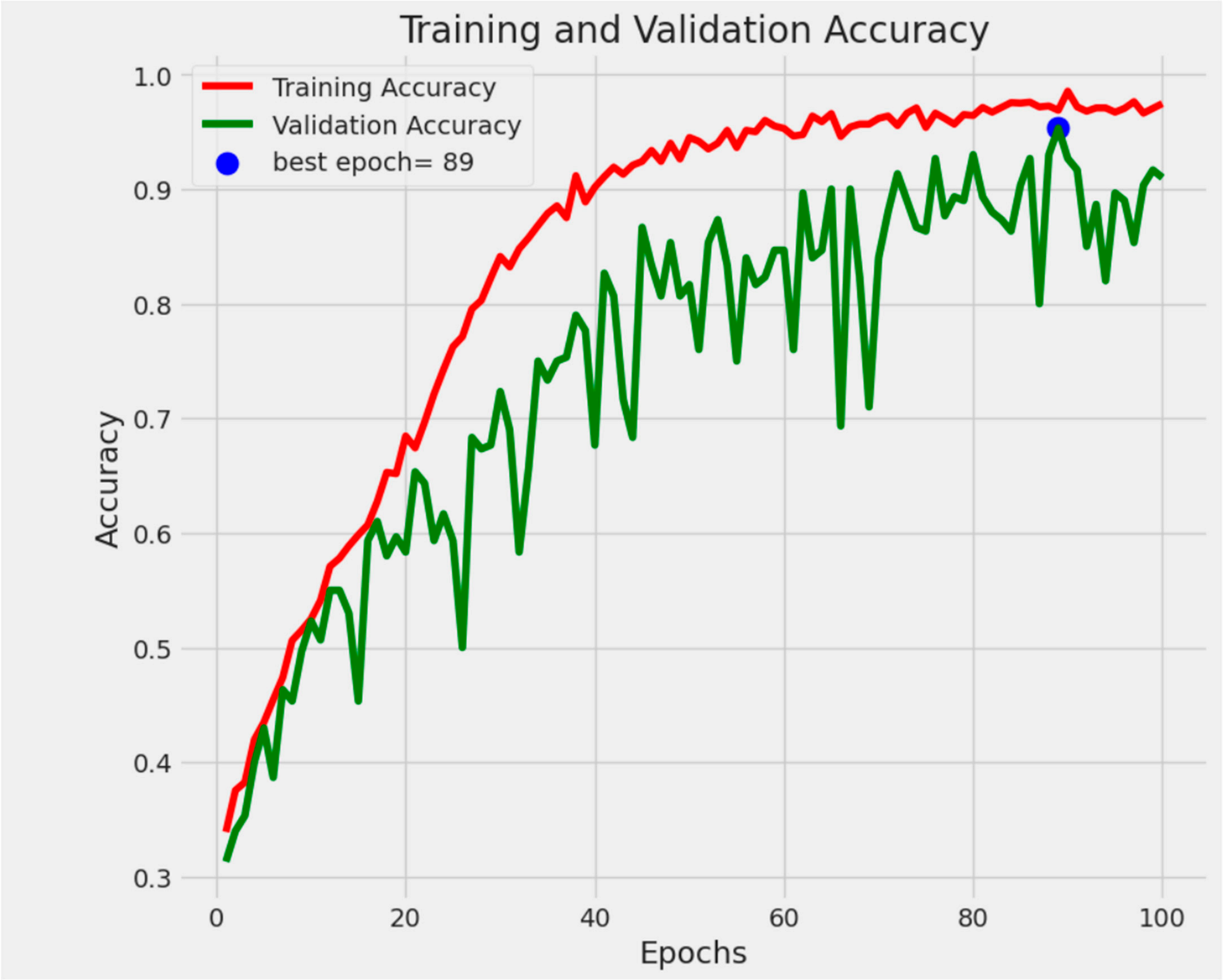
Training and Validation Accuracy
Training Accuracy
Validation Accuracy
best epoch= 89
Accuracy
Epochs
1.0
0.9
0.8
0.7
0.6
0.5
0.4
0.3
0
20
40
60
80
100

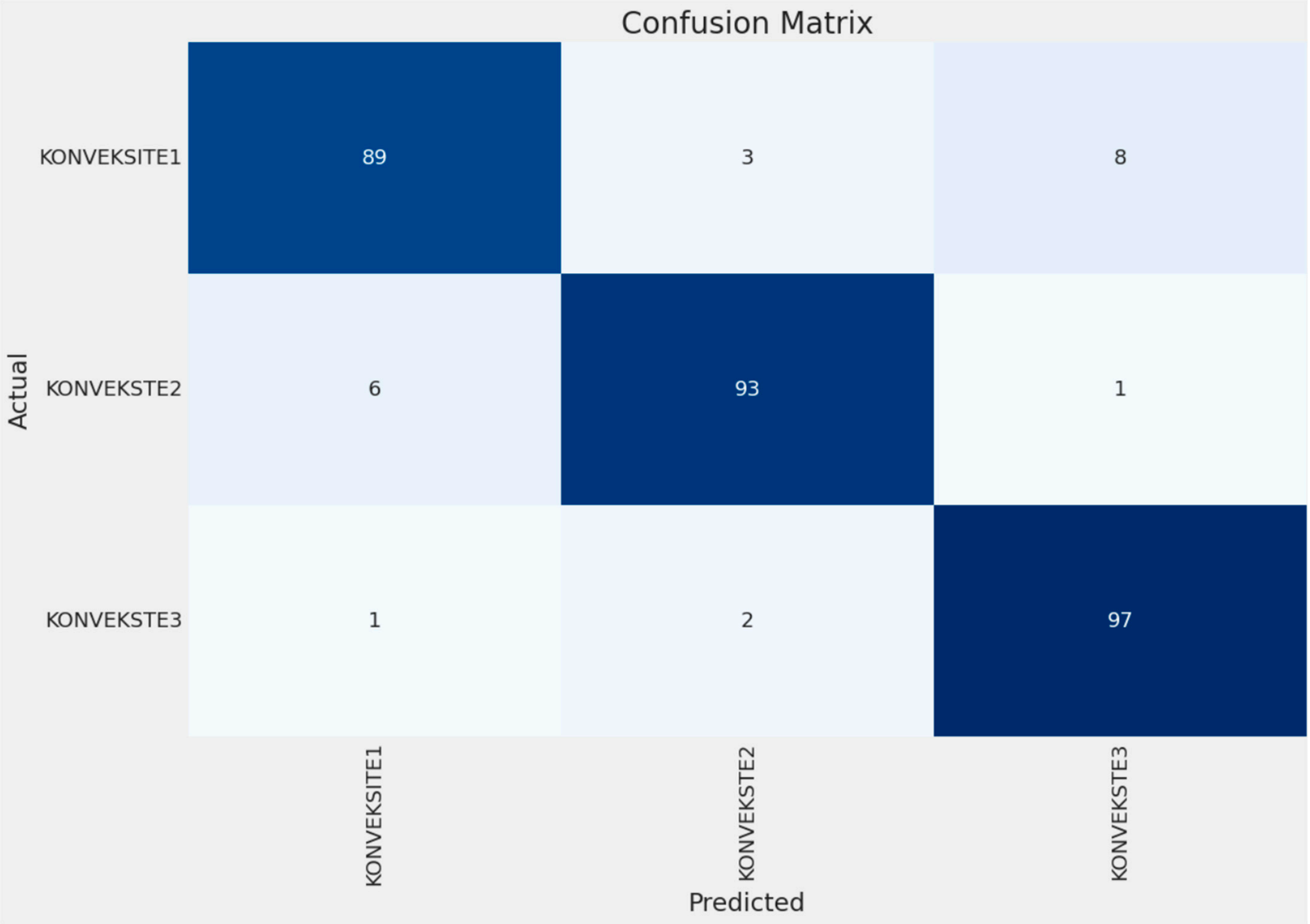

there were 21 errors in 300 tests for an accuracy of 93.00

Classification Report:
----

	precision	recall	f1-score	support
KONVEKSITE1	0.9271	0.8900	0.9082	100
KONVEKSTE2	0.9490	0.9300	0.9394	100
KONVEKSTE3	0.9151	0.9700	0.9417	100
accuracy			0.9300	300
macro avg	0.9304	0.9300	0.9298	300
weighted avg	0.9304	0.9300	0.9298	300

DENSENET 169

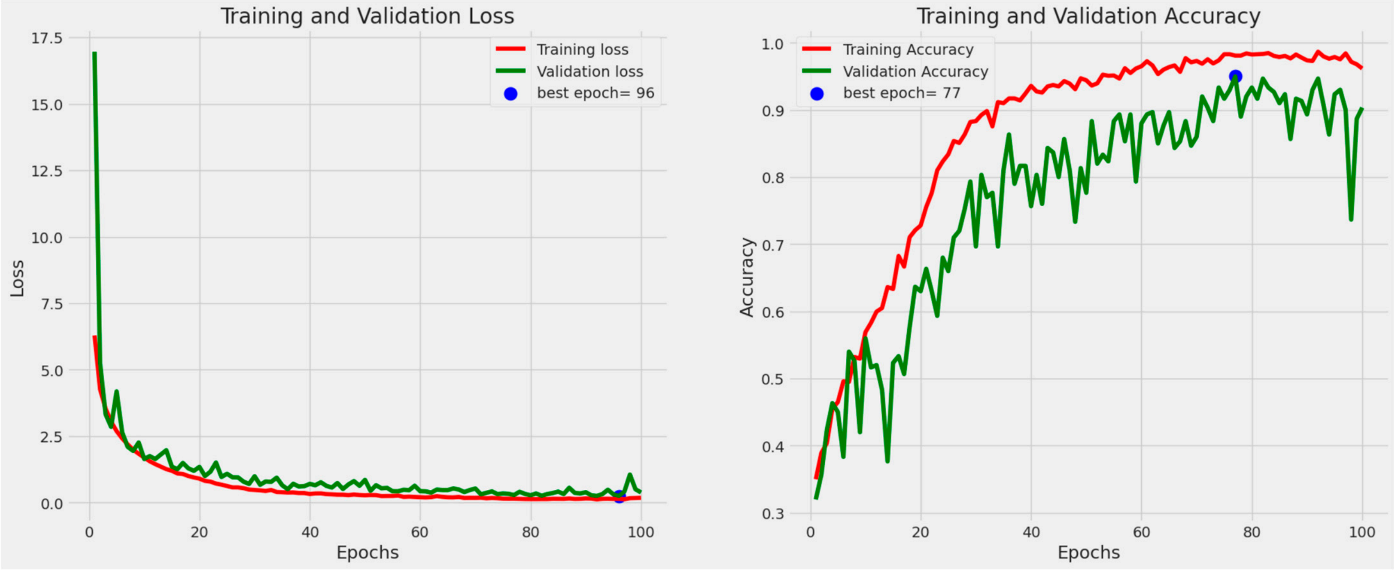

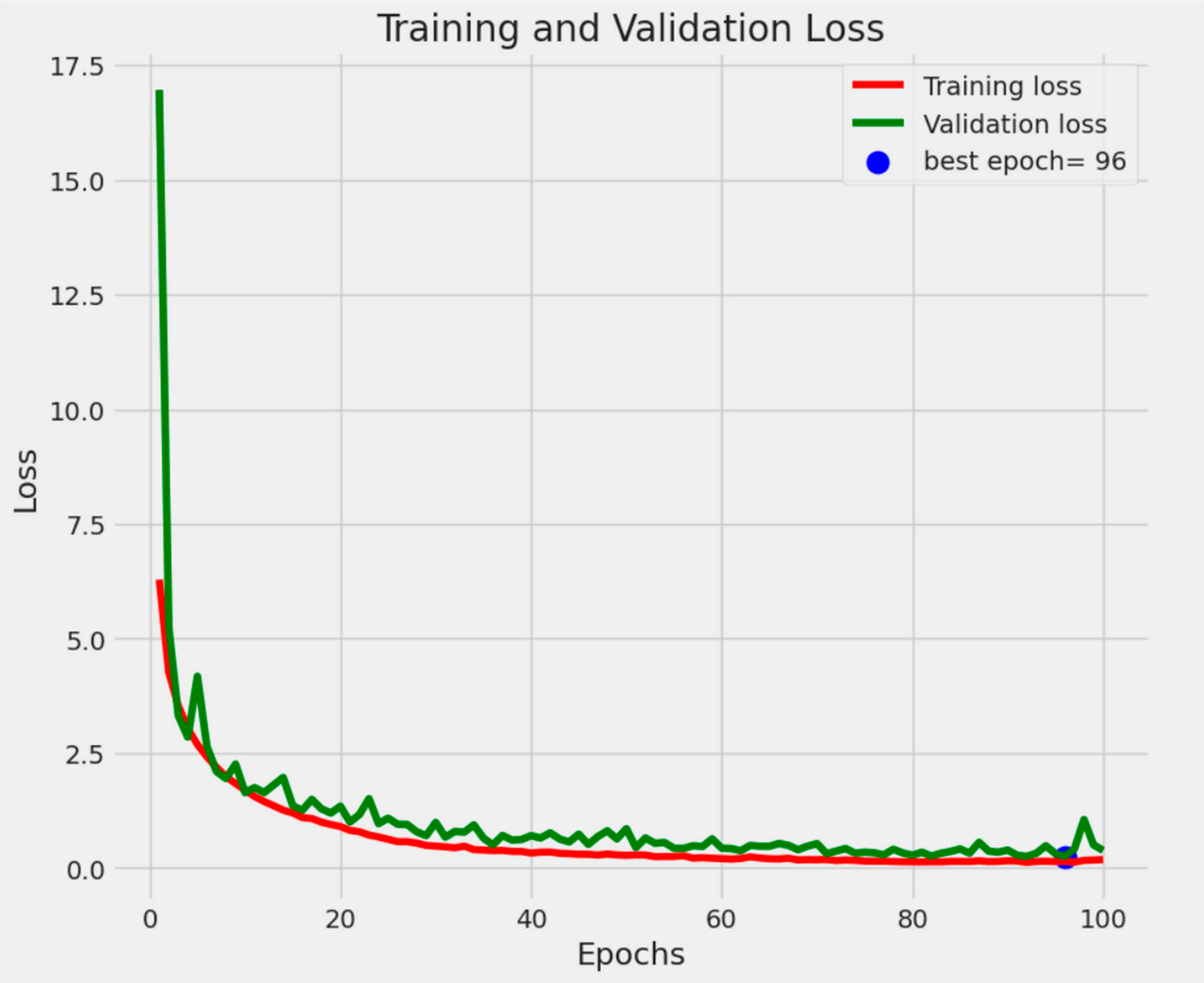

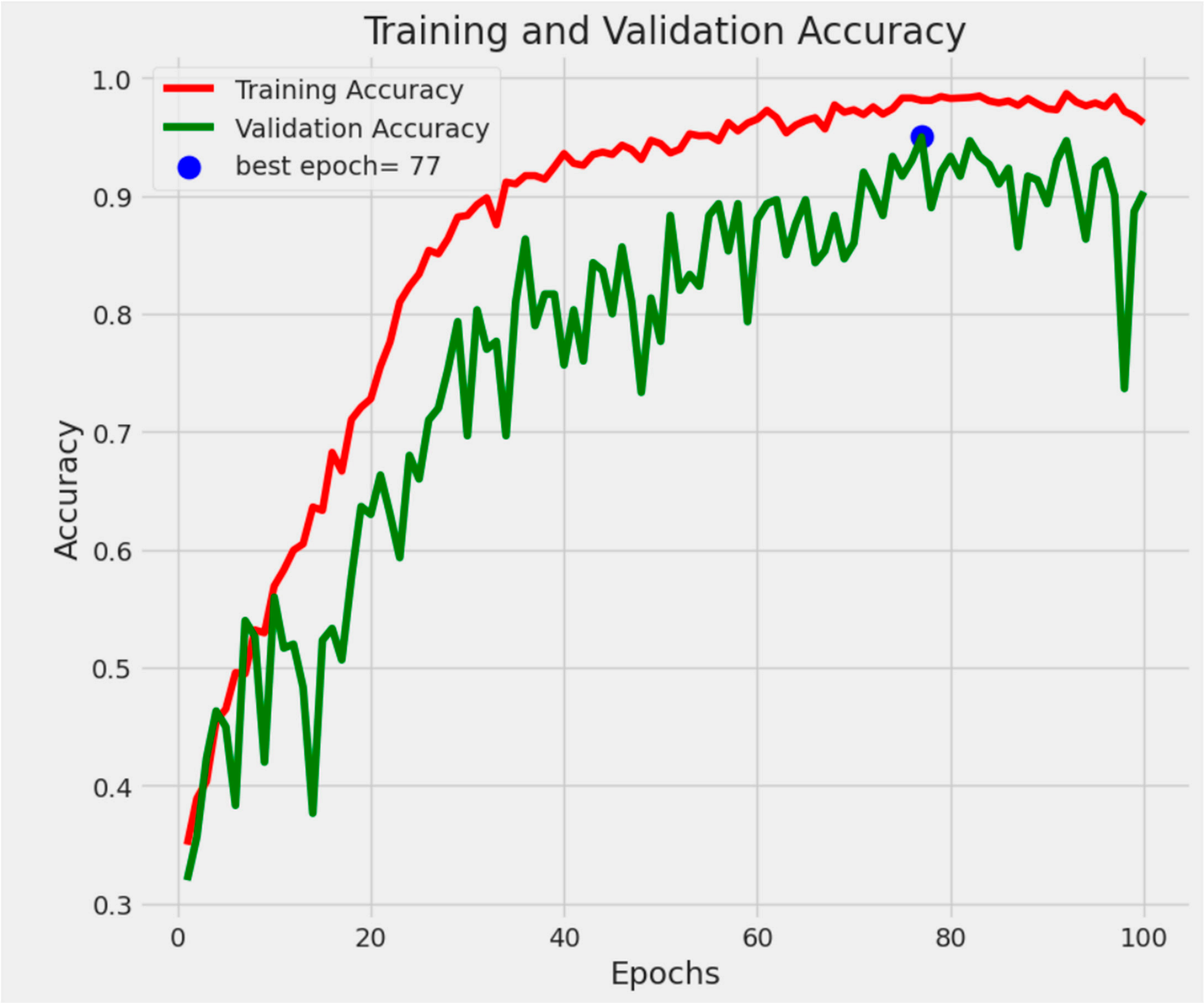
Training and Validation Accuracy
Training Accuracy
Validation Accuracy
best epoch= 77
Accuracy
Epochs
1.0
0.9
0.8
0.7
0.6
0.5
0.4
0.3
0
20
40
60
80
100

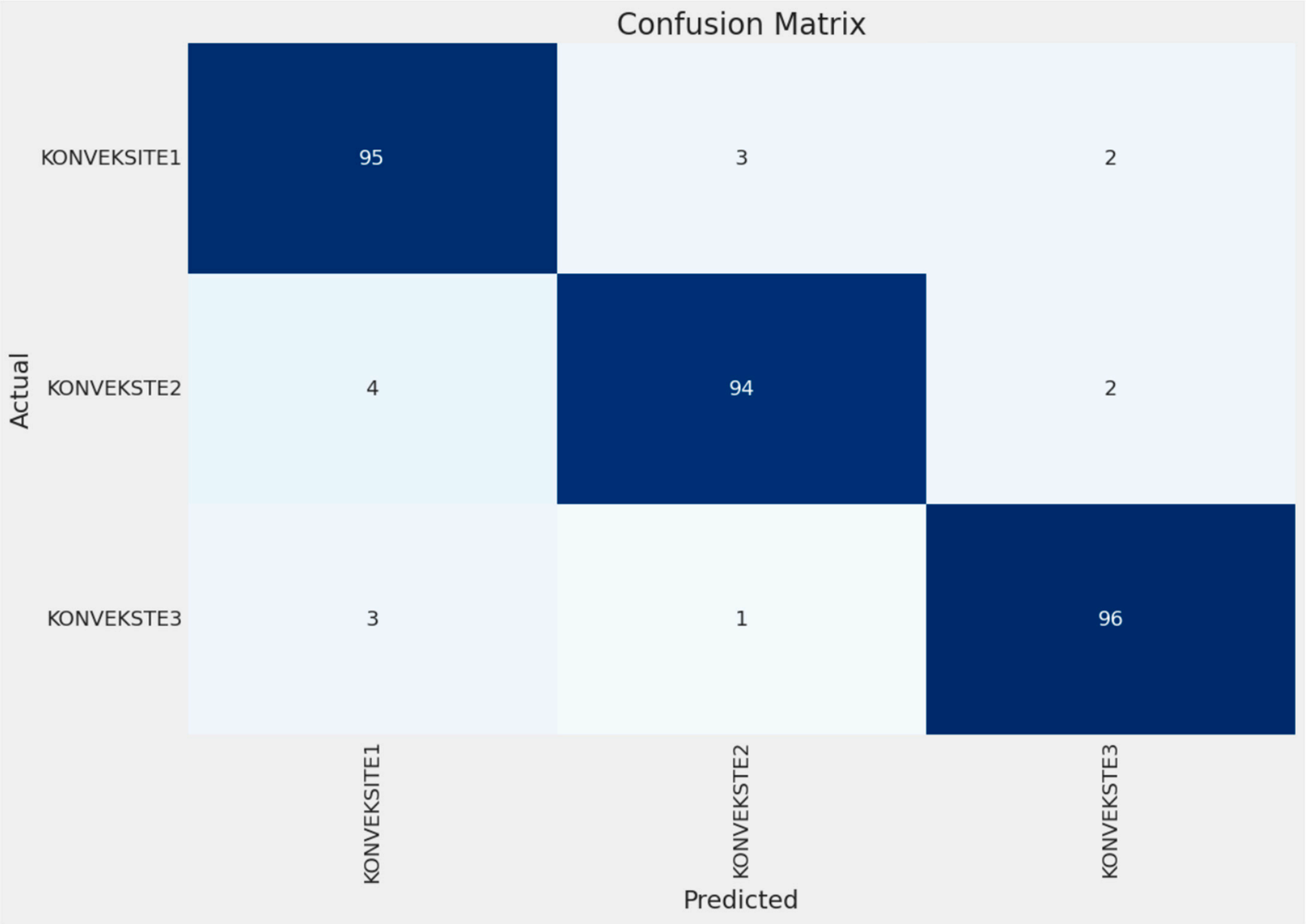

there were 15 errors in 300 tests for an accuracy of 95.00

Classification Report:
----

	precision	recall	f1-score	support
KONVEKSITE1	0.9314	0.9500	0.9406	100
KONVEKSTE2	0.9592	0.9400	0.9495	100
KONVEKSTE3	0.9600	0.9600	0.9600	100
accuracy			0.9500	300
macro avg	0.9502	0.9500	0.9500	300
weighted avg	0.9502	0.9500	0.9500	300

DENSENET 201

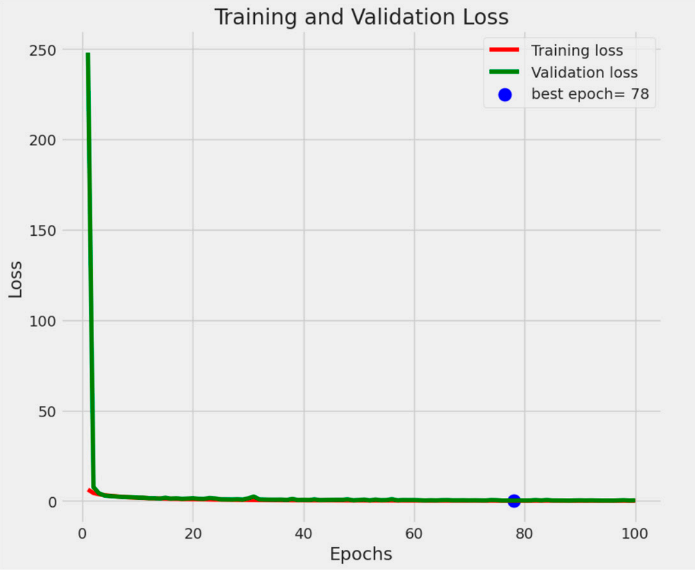

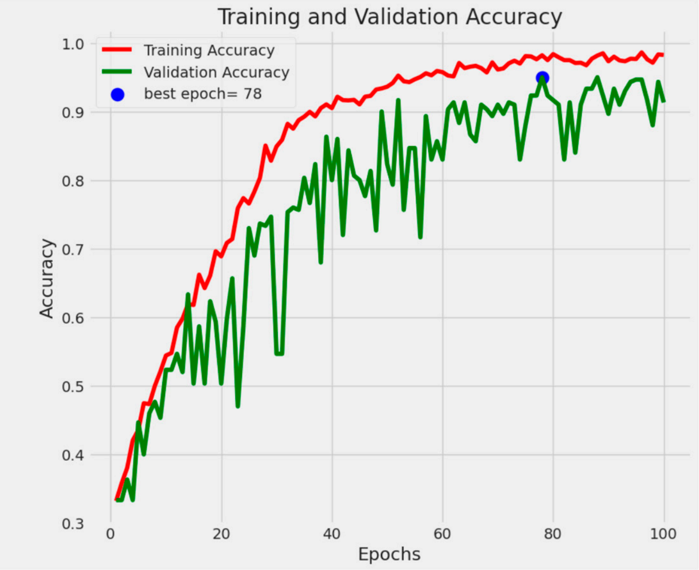

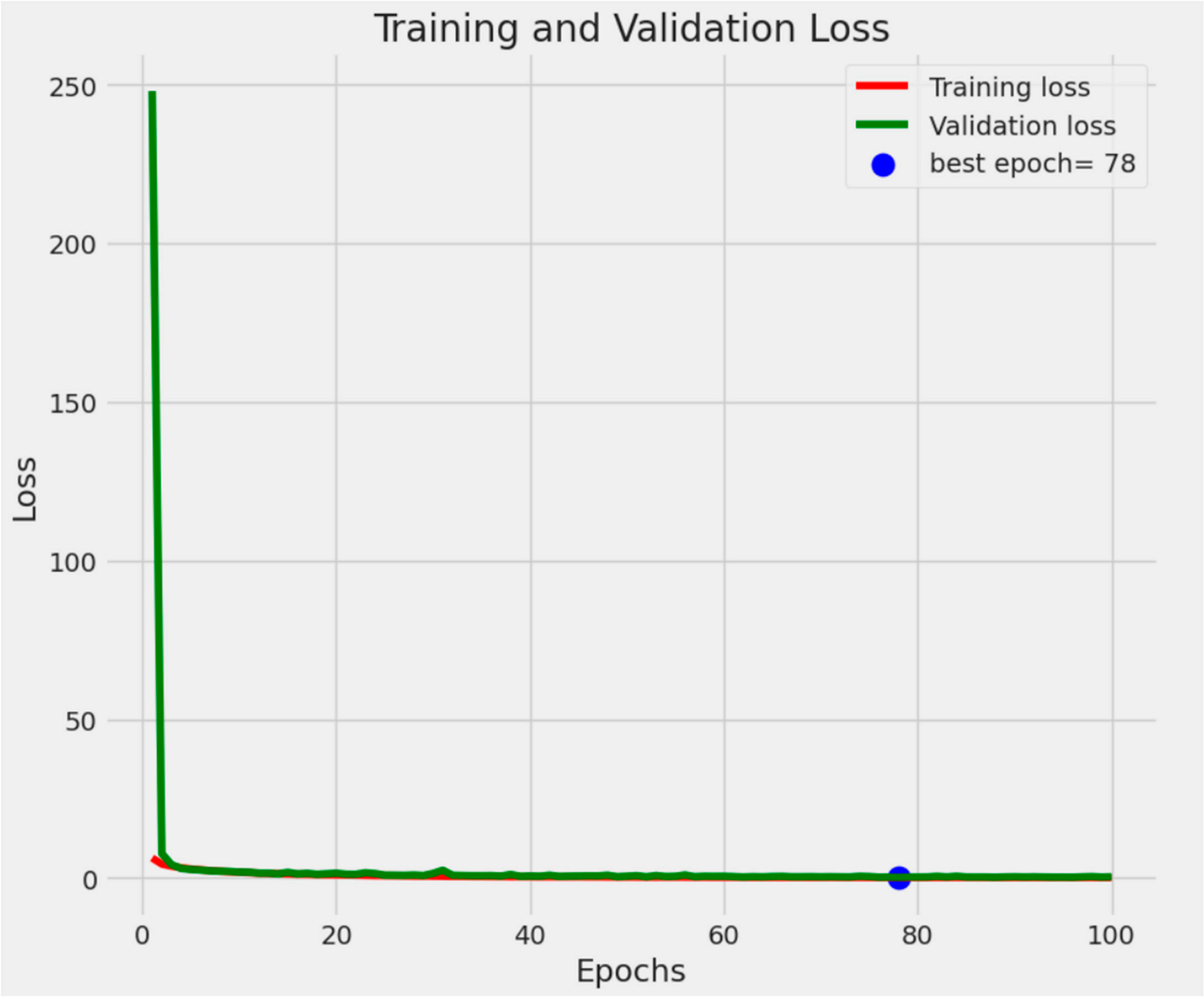

Training and Validation Loss
Training loss
Validation loss
best epoch= 78
250
200
150
100
50
0
Loss
0
20
40
60
80
100
Epochs

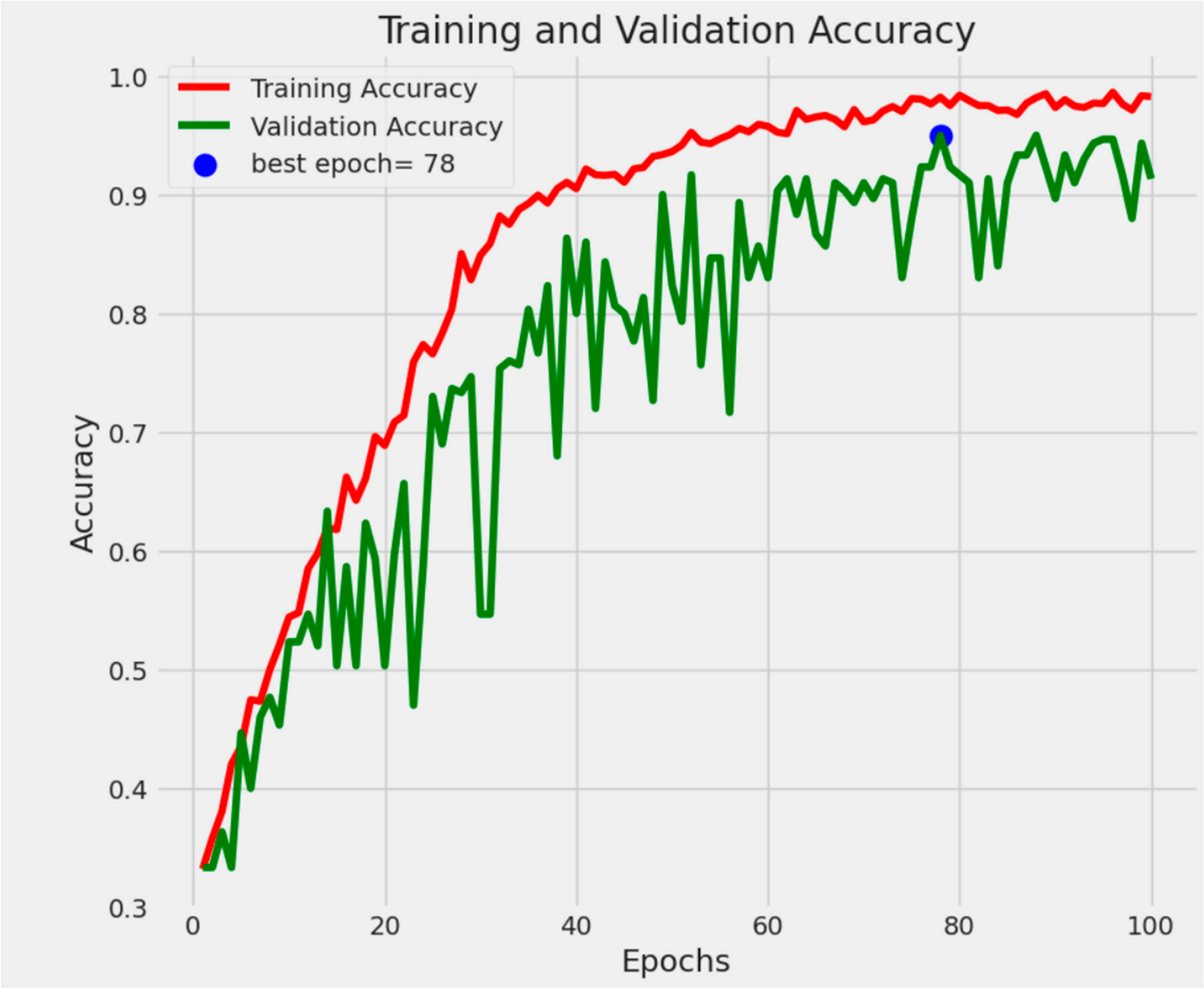
Training and Validation Accuracy
Training Accuracy
Validation Accuracy
best epoch= 78
Accuracy
Epochs
1.0
0.9
0.8
0.7
0.6
0.5
0.4
0.3
0
20
40
60
80
100

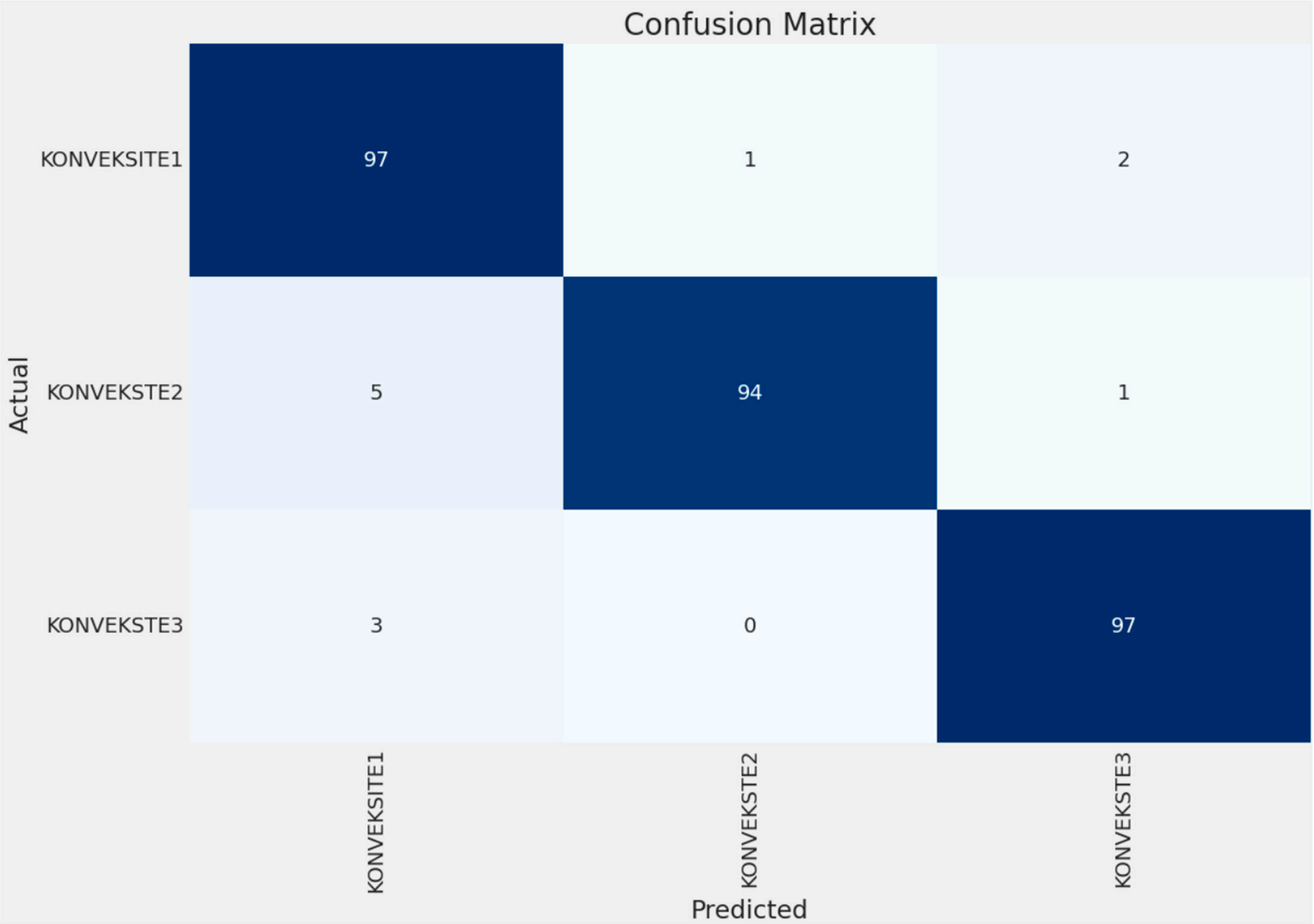

there were 12 errors in 300 tests for an accuracy of 96.00

Classification Report:

----

	precision	recall	f1-score	support
KONVEKSITE1	0.9238	0.9700	0.9463	100
KONVEKSTE2	0.9895	0.9400	0.9641	100
KONVEKSTE3	0.9700	0.9700	0.9700	100
accuracy			0.9600	300
macro avg	0.9611	0.9600	0.9601	300
weighted avg	0.9611	0.9600	0.9601	300

EFFICIENTNET B0

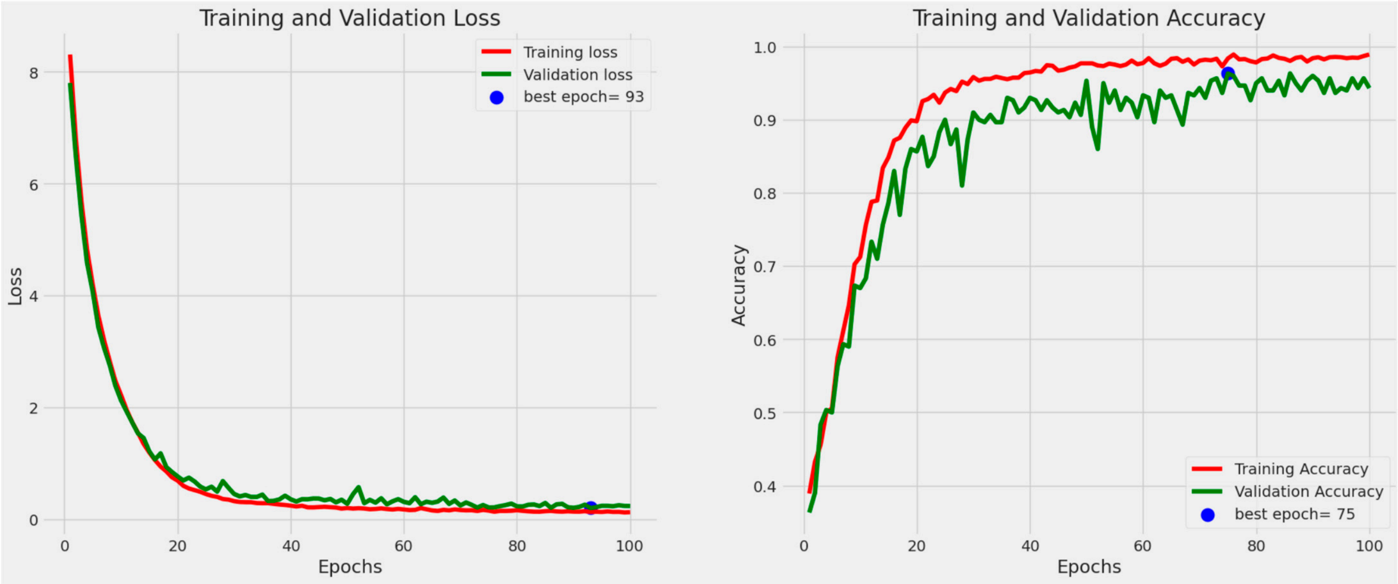

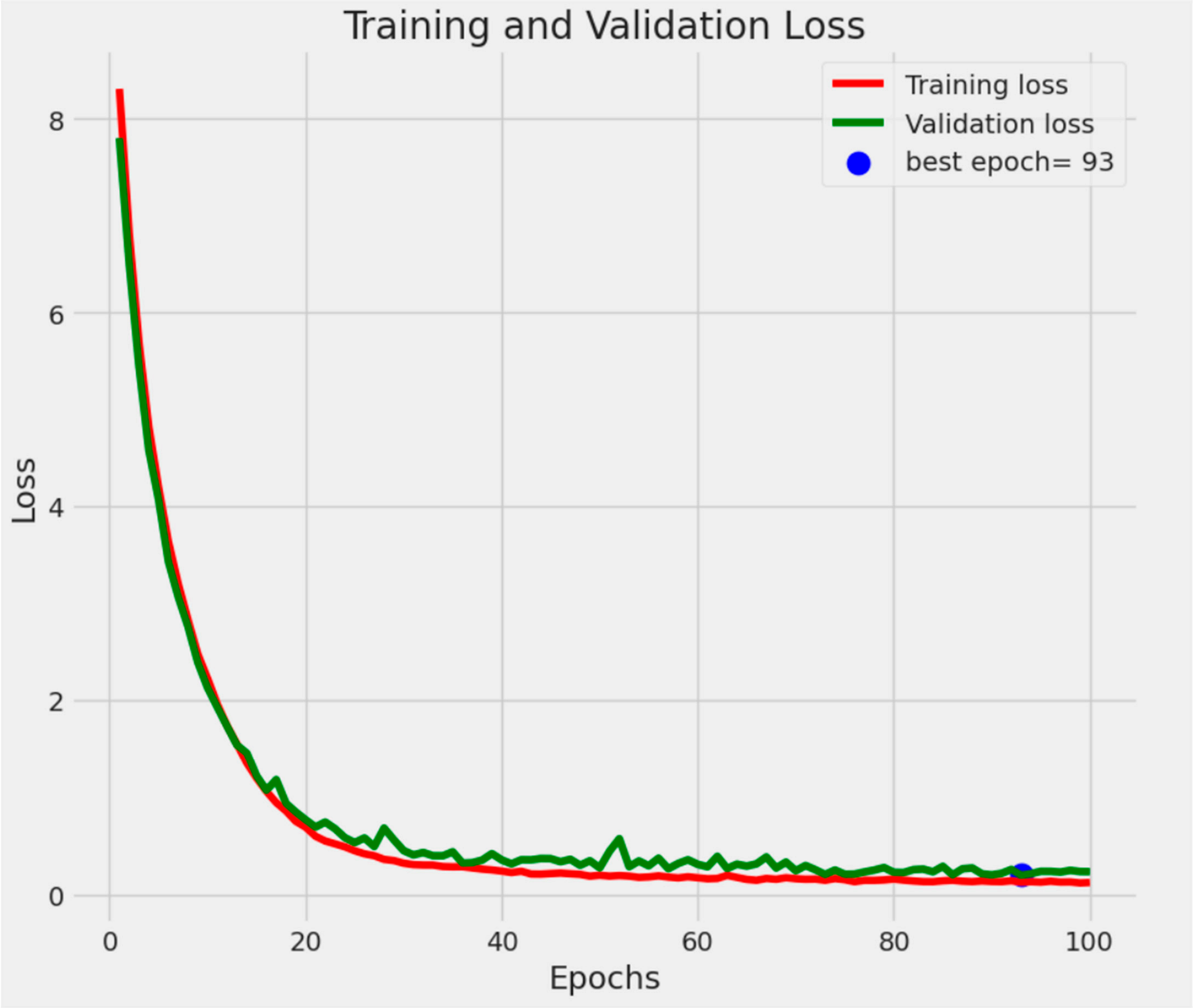

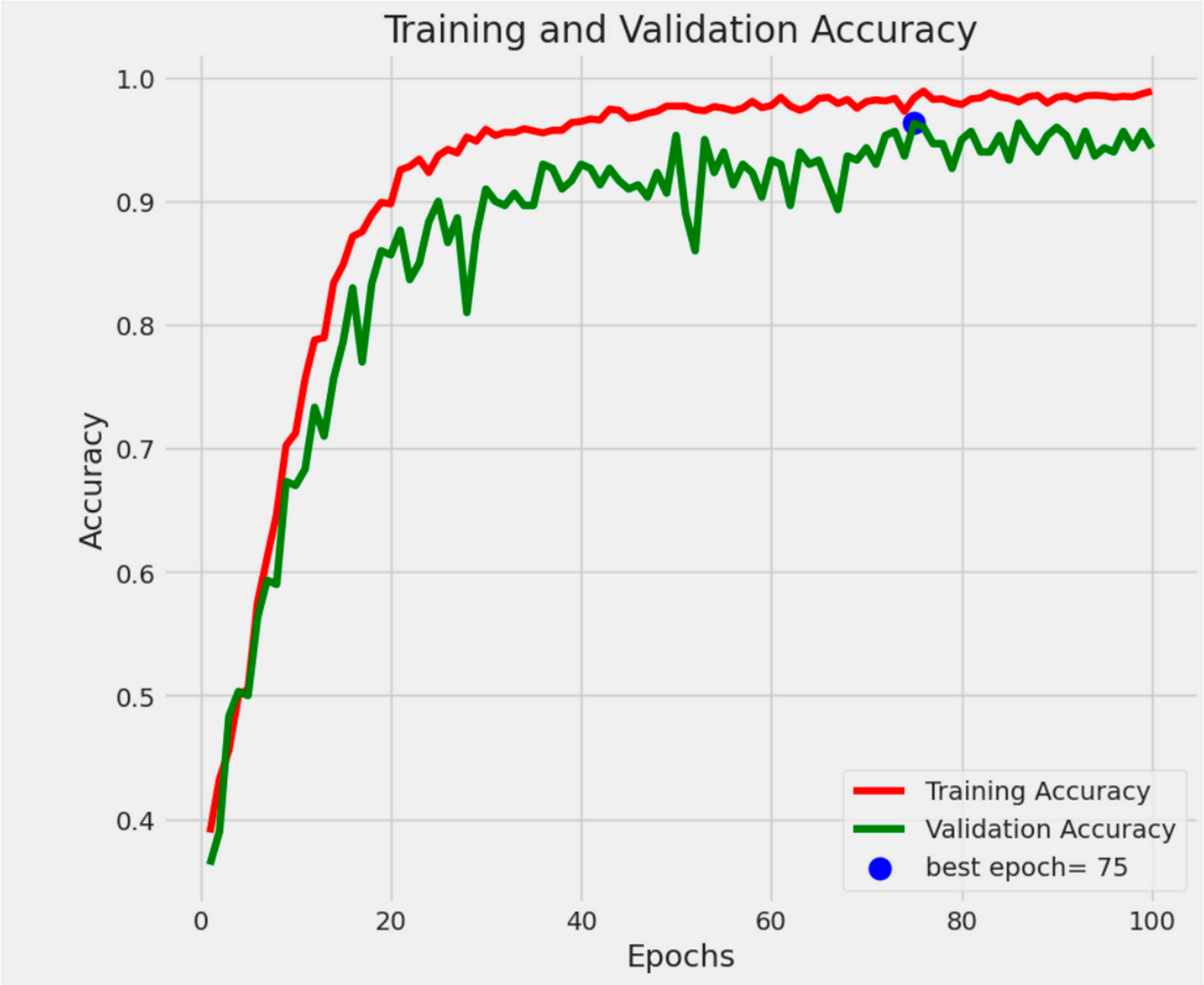

Training and Validation Accuracy
Accuracy
Epochs
1.0
0.9
0.8
0.7
0.6
0.5
0.4
0
20
40
60
80
100
Training Accuracy
Validation Accuracy
best epoch= 75

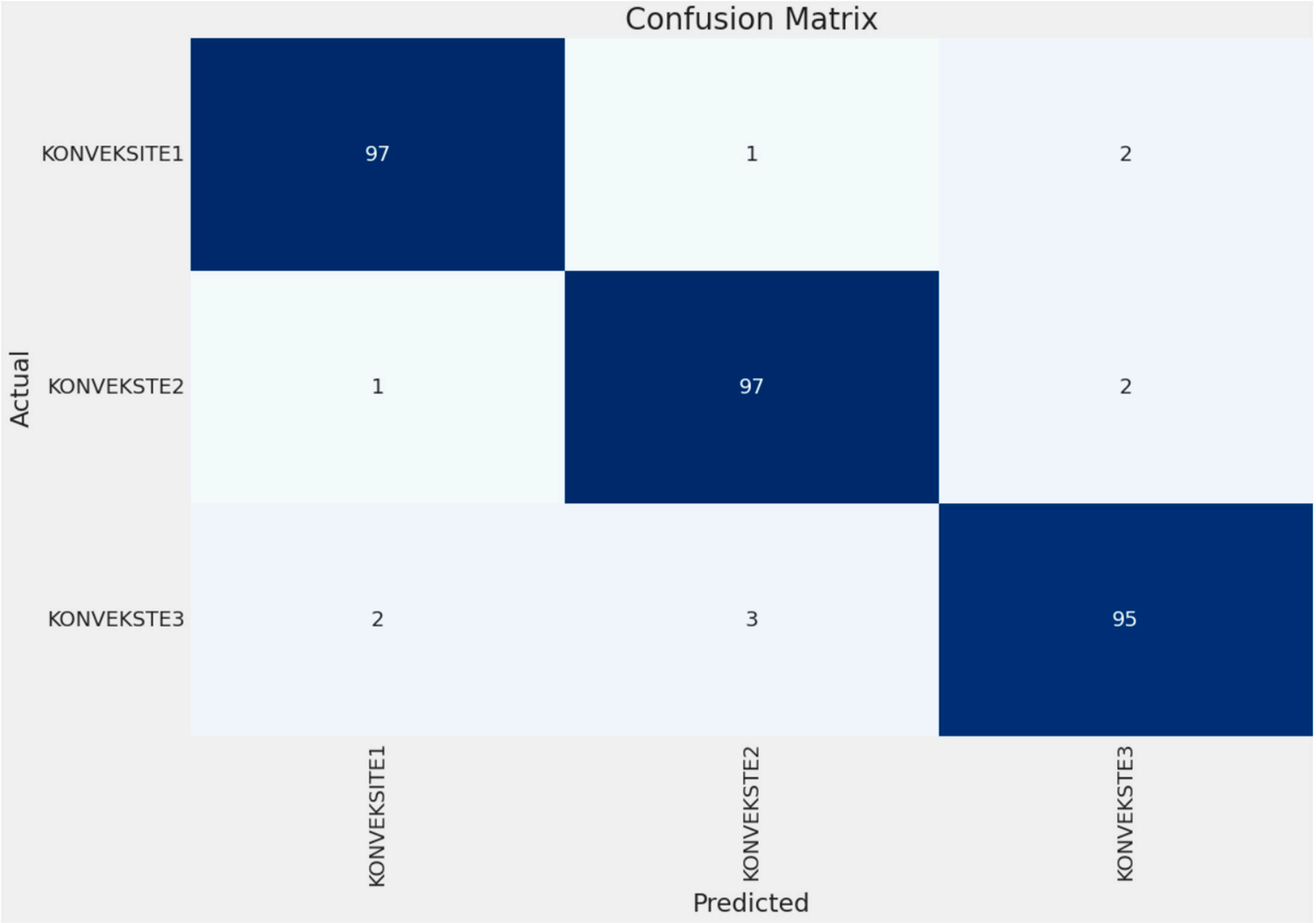


```
there were 11 errors in 300 tests for an accuracy of  96.33

Classification Report:
----------------------
               precision    recall  f1-score   support

 KONVEKSITE1      0.9700    0.9700    0.9700       100
  KONVEKSTE2      0.9604    0.9700    0.9652       100
  KONVEKSTE3      0.9596    0.9500    0.9548       100

    accuracy                          0.9633       300
   macro avg      0.9633    0.9633    0.9633       300
weighted avg      0.9633    0.9633    0.9633       300
```

XCEPTION

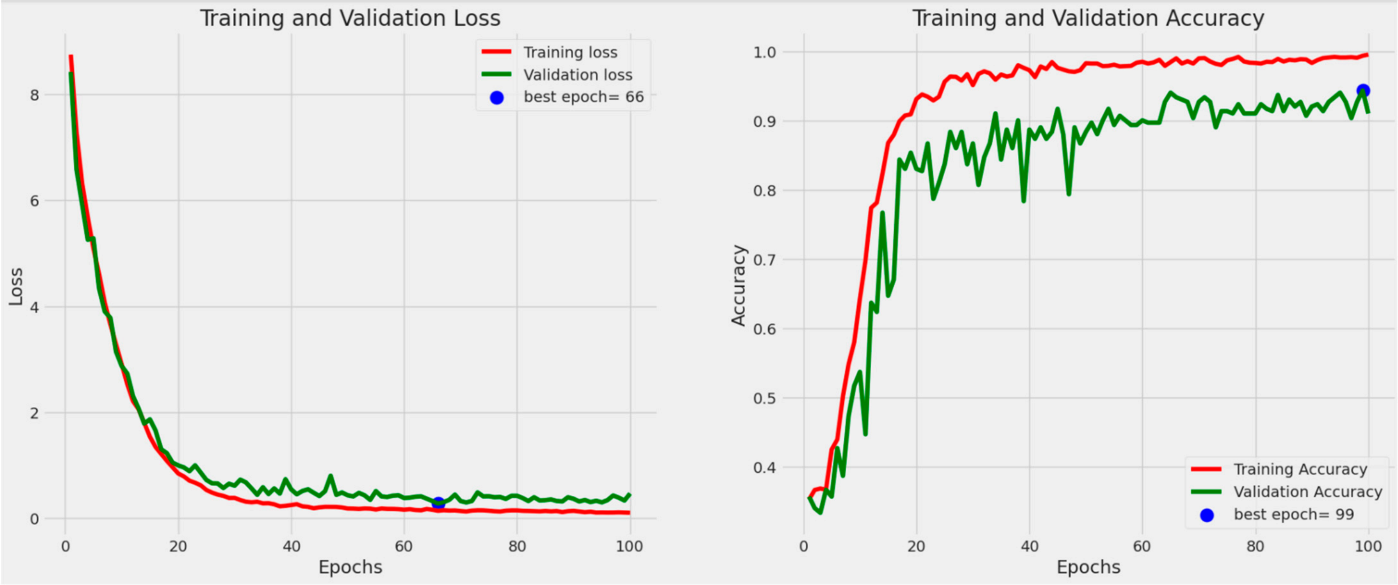

Training and Validation Loss
Training loss
Validation loss
best epoch= 66
Loss
Epochs
Training and Validation Accuracy
Training Accuracy
Validation Accuracy
best epoch= 99
Accuracy
Epochs

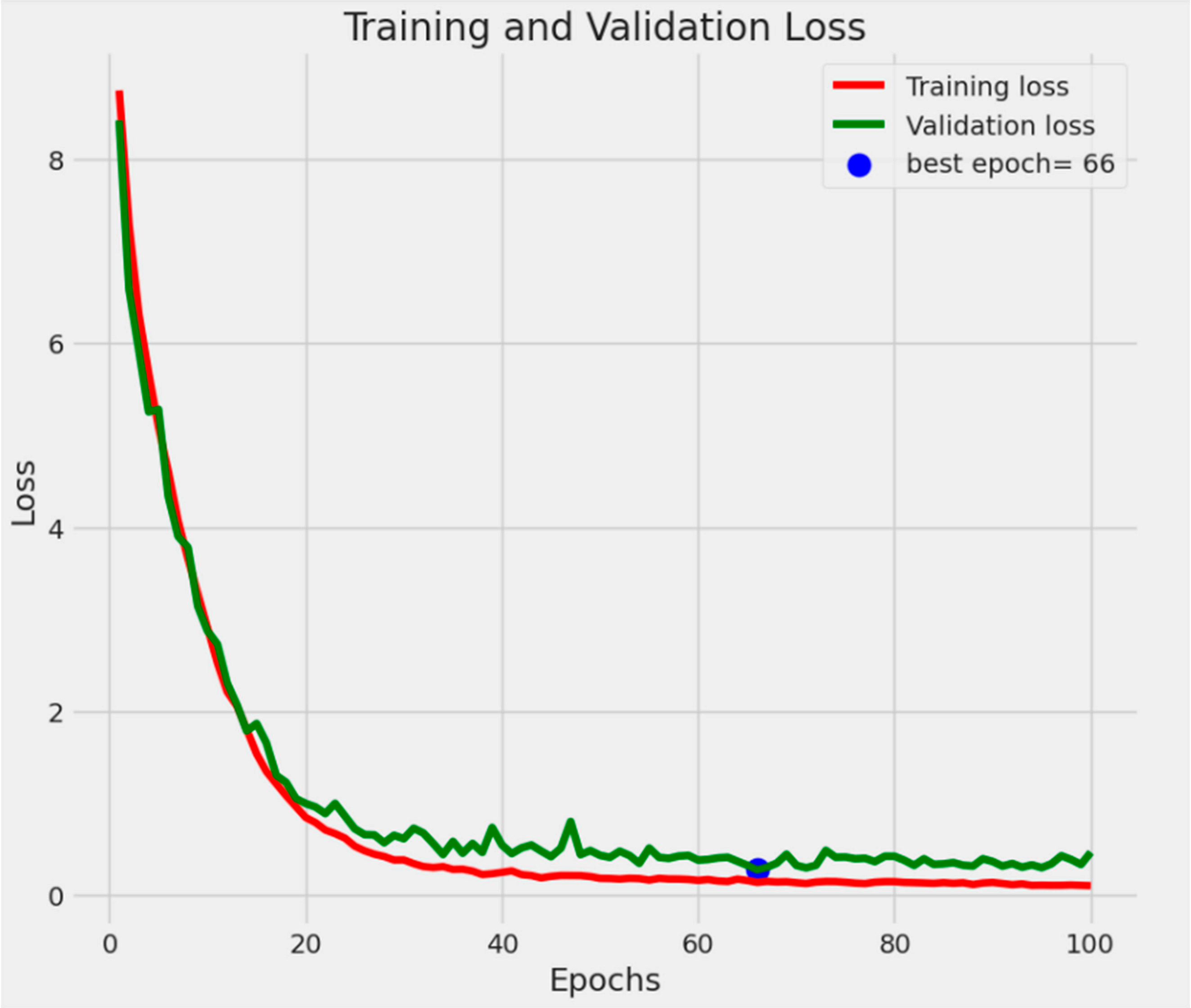

Training and Validation Loss
Training loss
Validation loss
best epoch= 66
Loss
Epochs

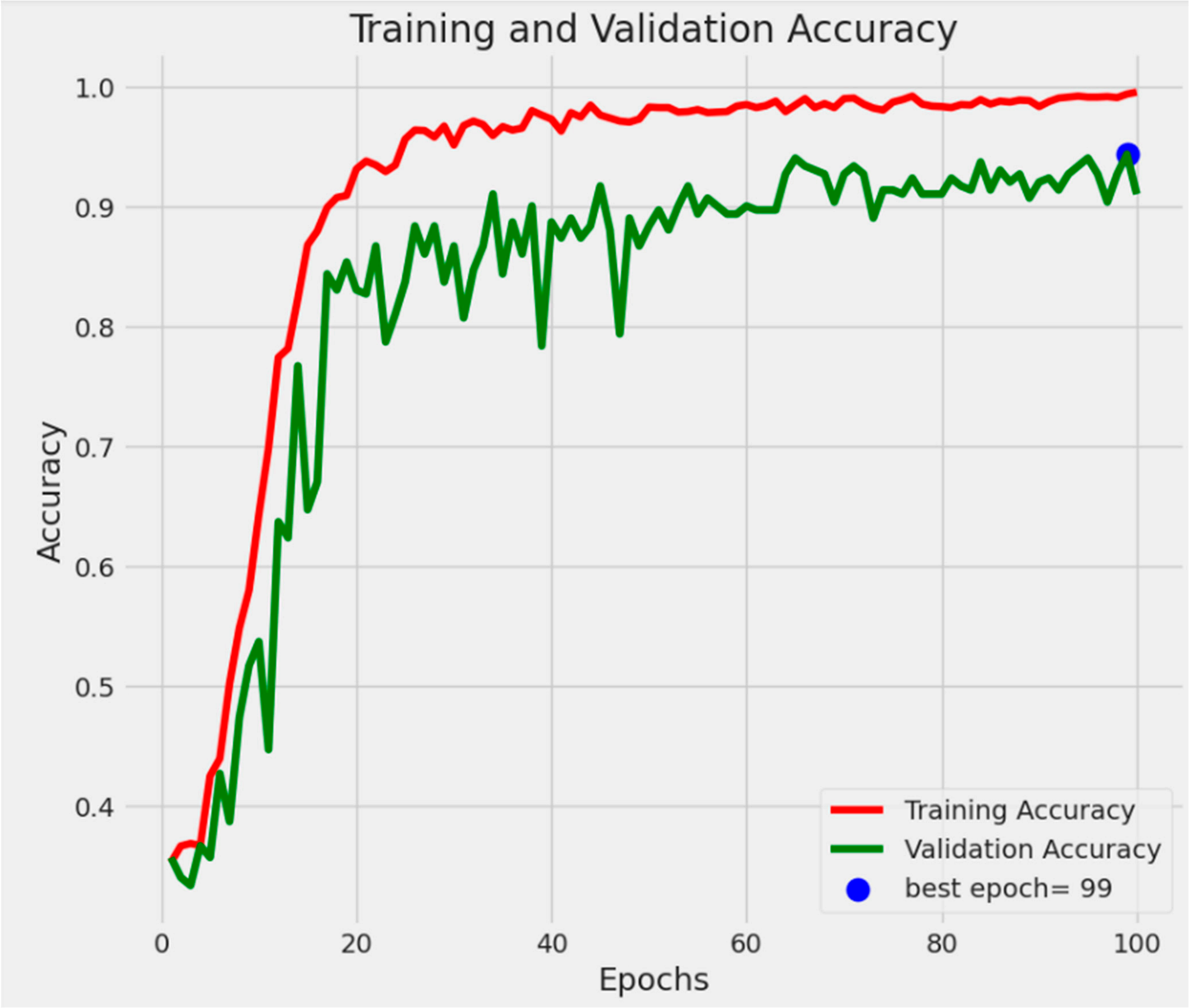
Training and Validation Accuracy
Accuracy
Epochs
1.0
0.9
0.8
0.7
0.6
0.5
0.4
0
20
40
60
80
100
Training Accuracy
Validation Accuracy
best epoch= 99

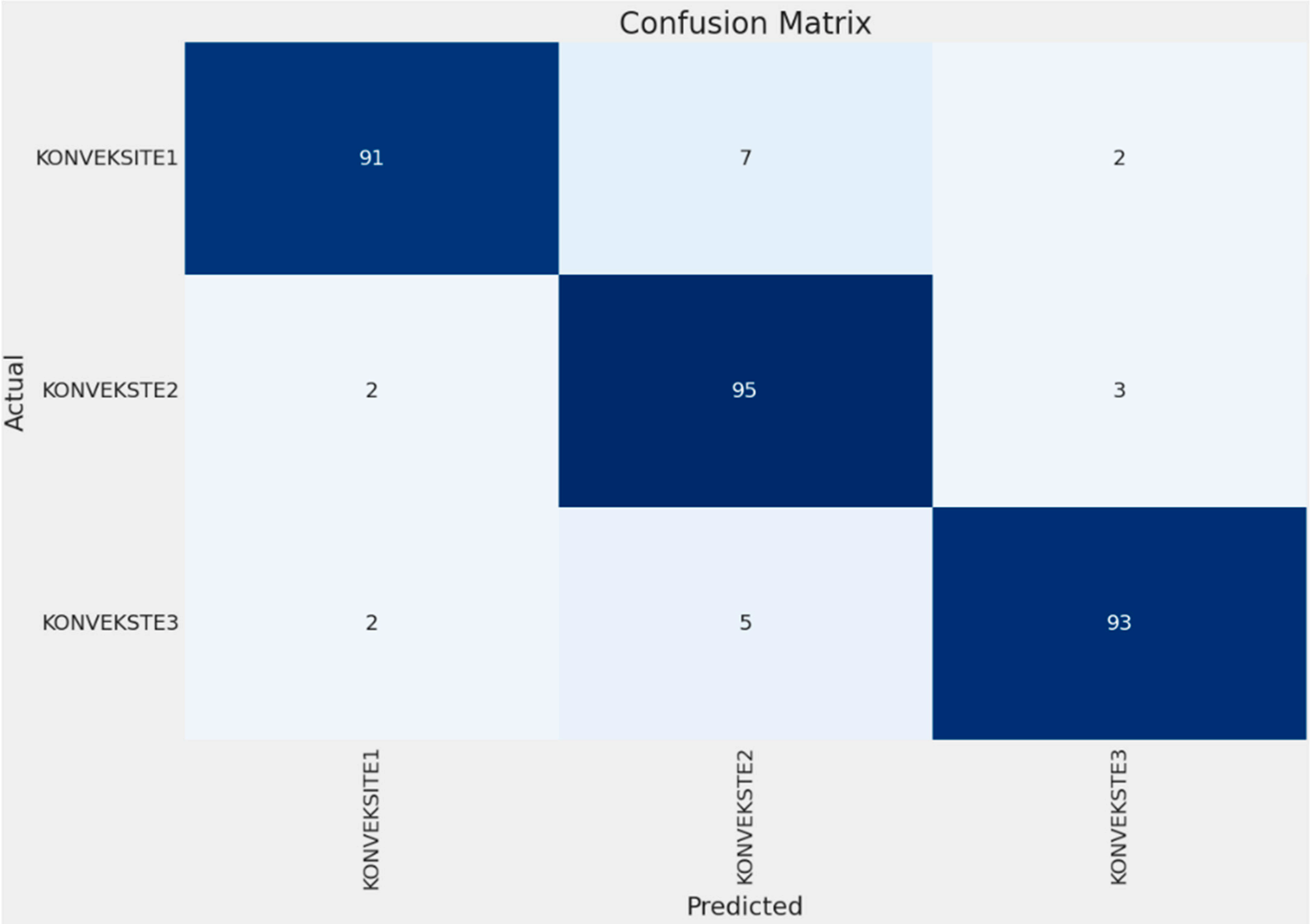

there were 21 errors in 300 tests for an accuracy of 93.00

Classification Report:
----

	precision	recall	f1-score	support
KONVEKSITE1	0.9579	0.9100	0.9333	100
KONVEKSTE2	0.8879	0.9500	0.9179	100
KONVEKSTE3	0.9490	0.9300	0.9394	100
accuracy			0.9300	300
macro avg	0.9316	0.9300	0.9302	300
weighted avg	0.9316	0.9300	0.9302	300

VGG16: %35.33

VGG19: %34.67

NASNETMOBILE

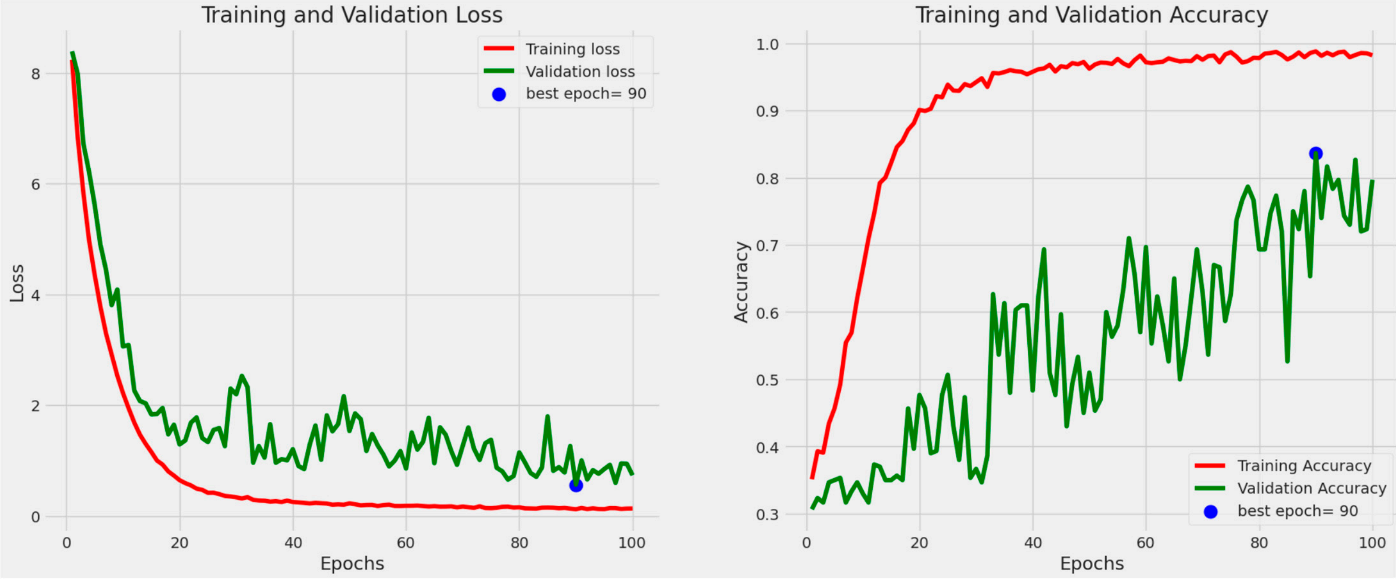

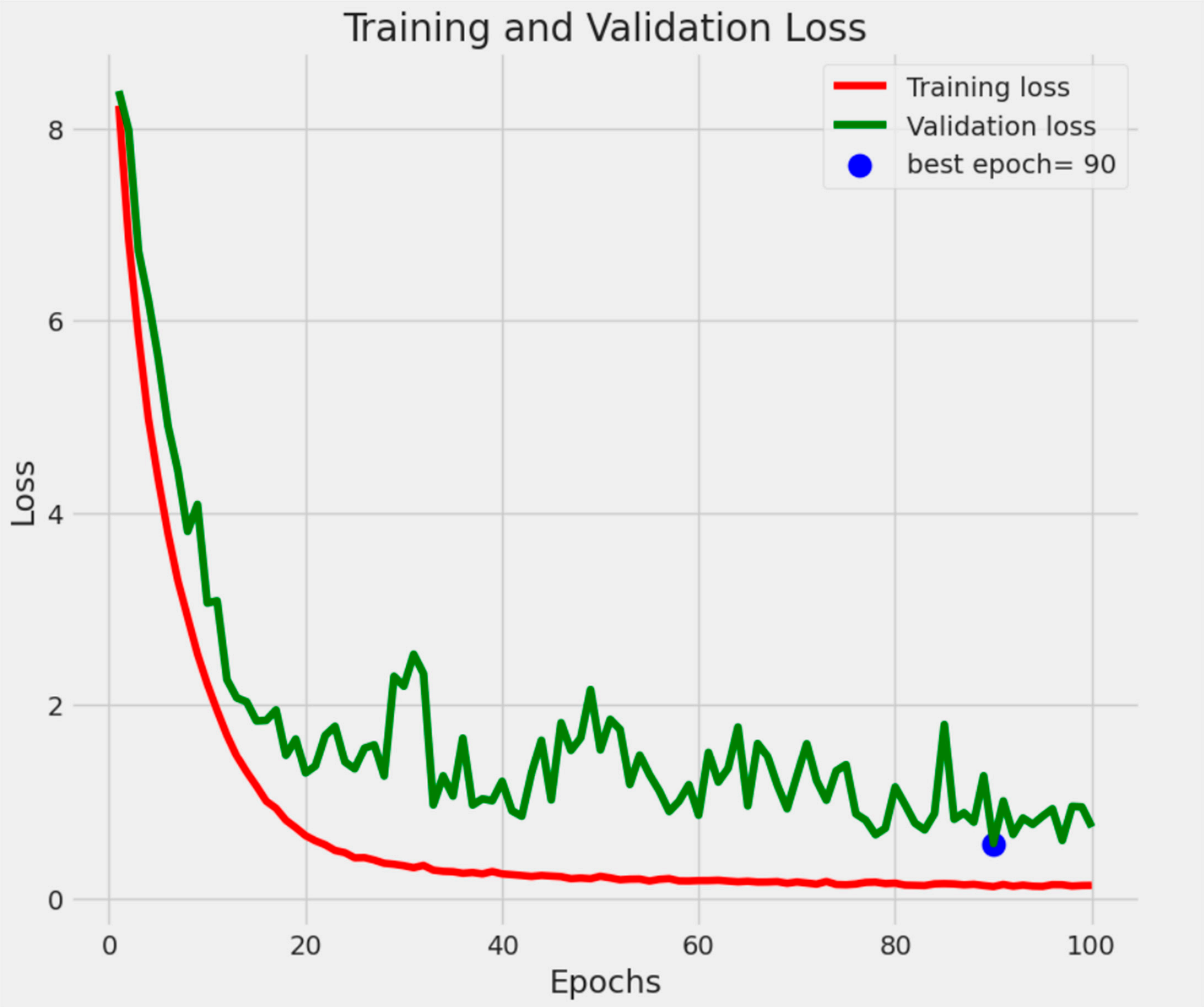

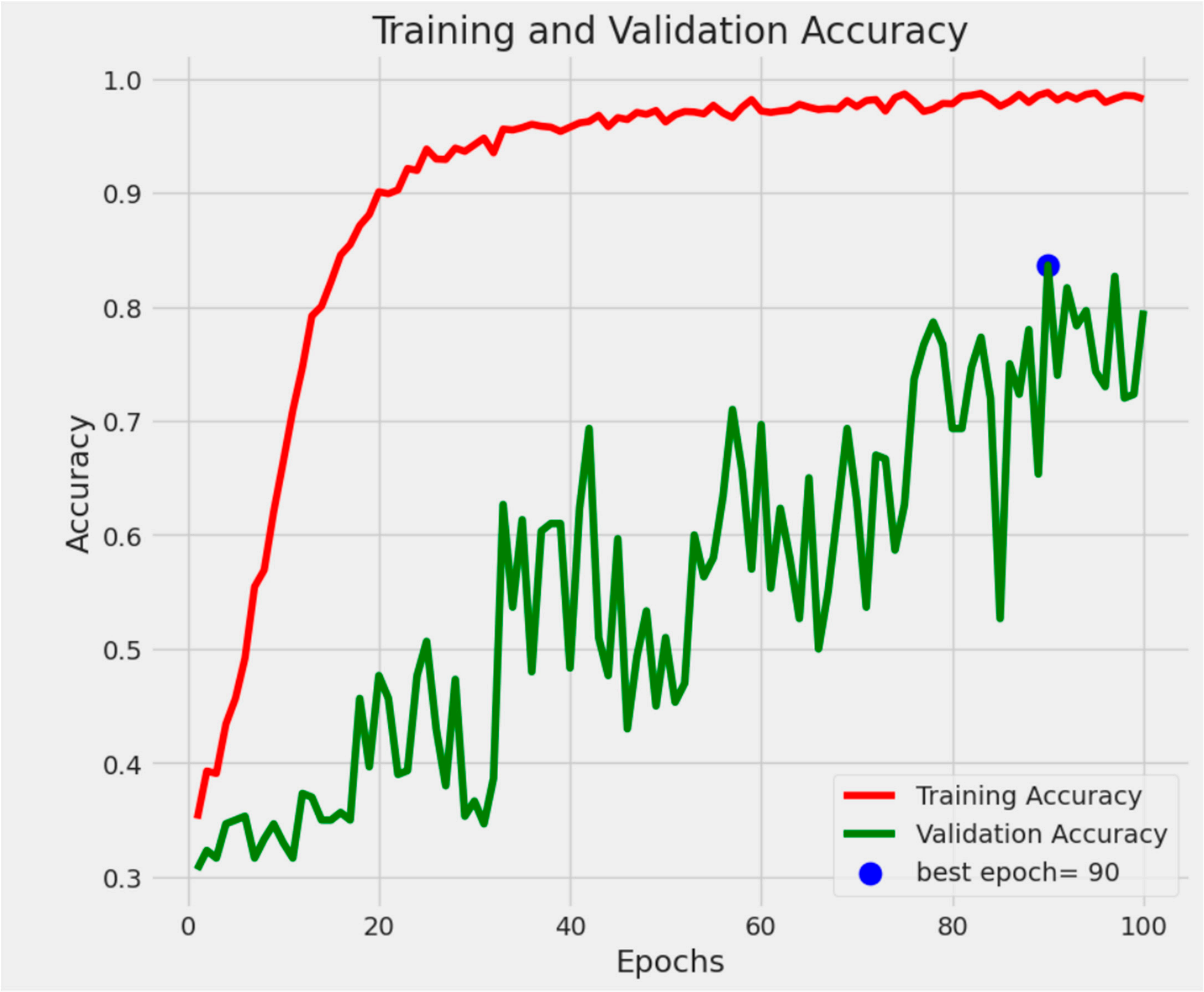
Training and Validation Accuracy
1.0
0.9
0.8
0.7
0.6
0.5
0.4
0.3
Accuracy
0
20
40
60
80
100
Epochs
Training Accuracy
Validation Accuracy
best epoch= 90

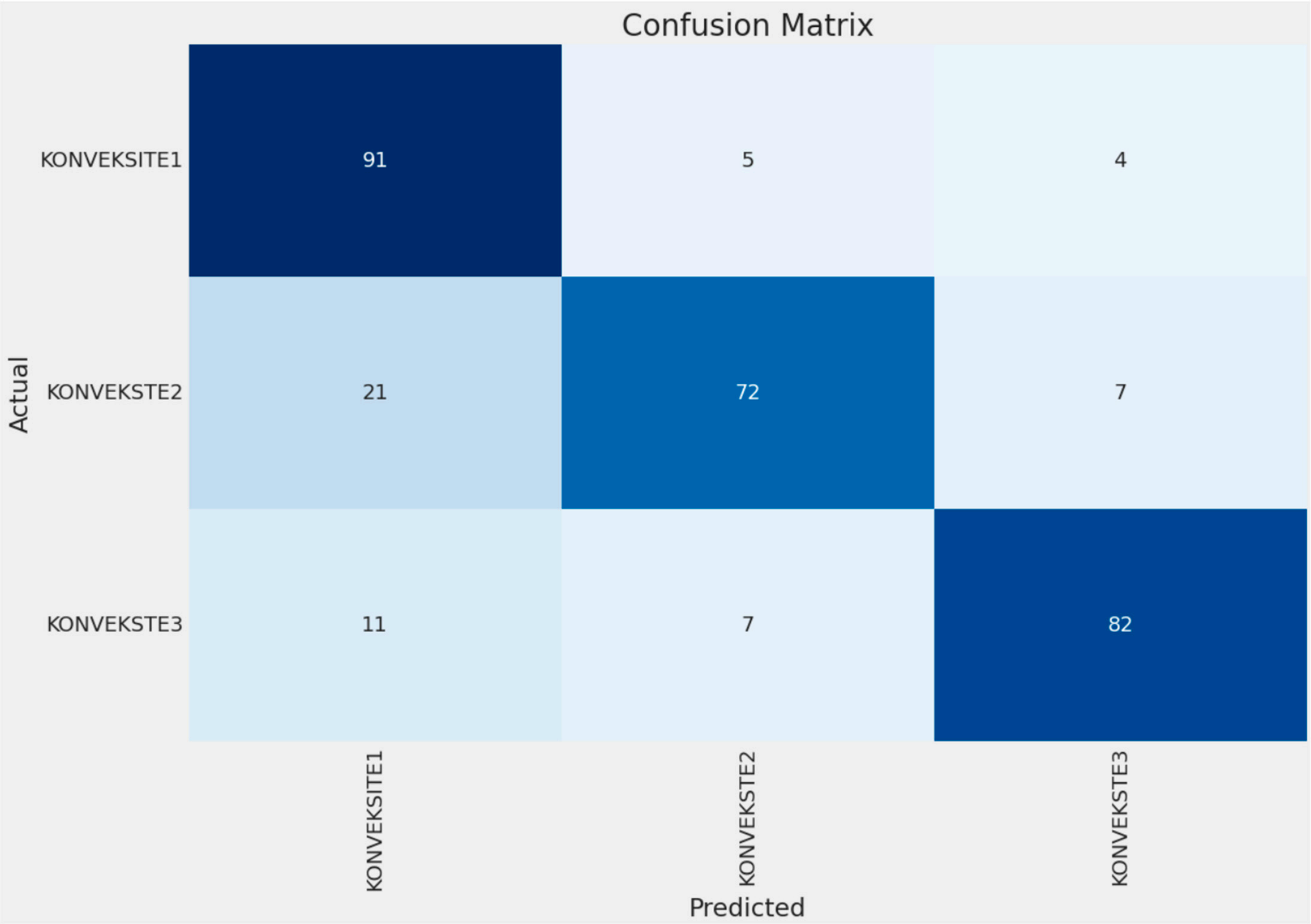

there were 55 errors in 300 tests for an accuracy of 81.67

Classification Report:
----

	precision	recall	f1-score	support
KONVEKSITE1	0.7398	0.9100	0.8161	100
KONVEKSTE2	0.8571	0.7200	0.7826	100
KONVEKSTE3	0.8817	0.8200	0.8497	100
accuracy			0.8167	300
macro avg	0.8262	0.8167	0.8162	300
weighted avg	0.8262	0.8167	0.8162	300

RESNET 101

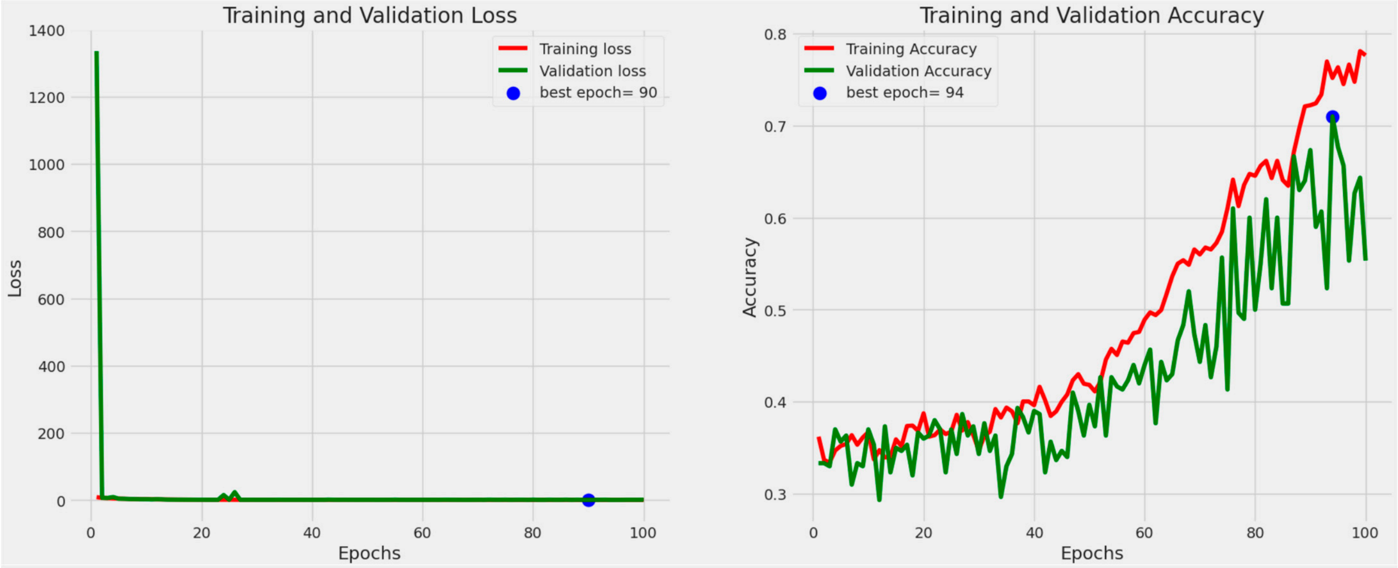
Training and Validation Loss
Training loss
Validation loss
best epoch= 90
Loss
Epochs
Training and Validation Accuracy
Training Accuracy
Validation Accuracy
best epoch= 94
Accuracy
Epochs

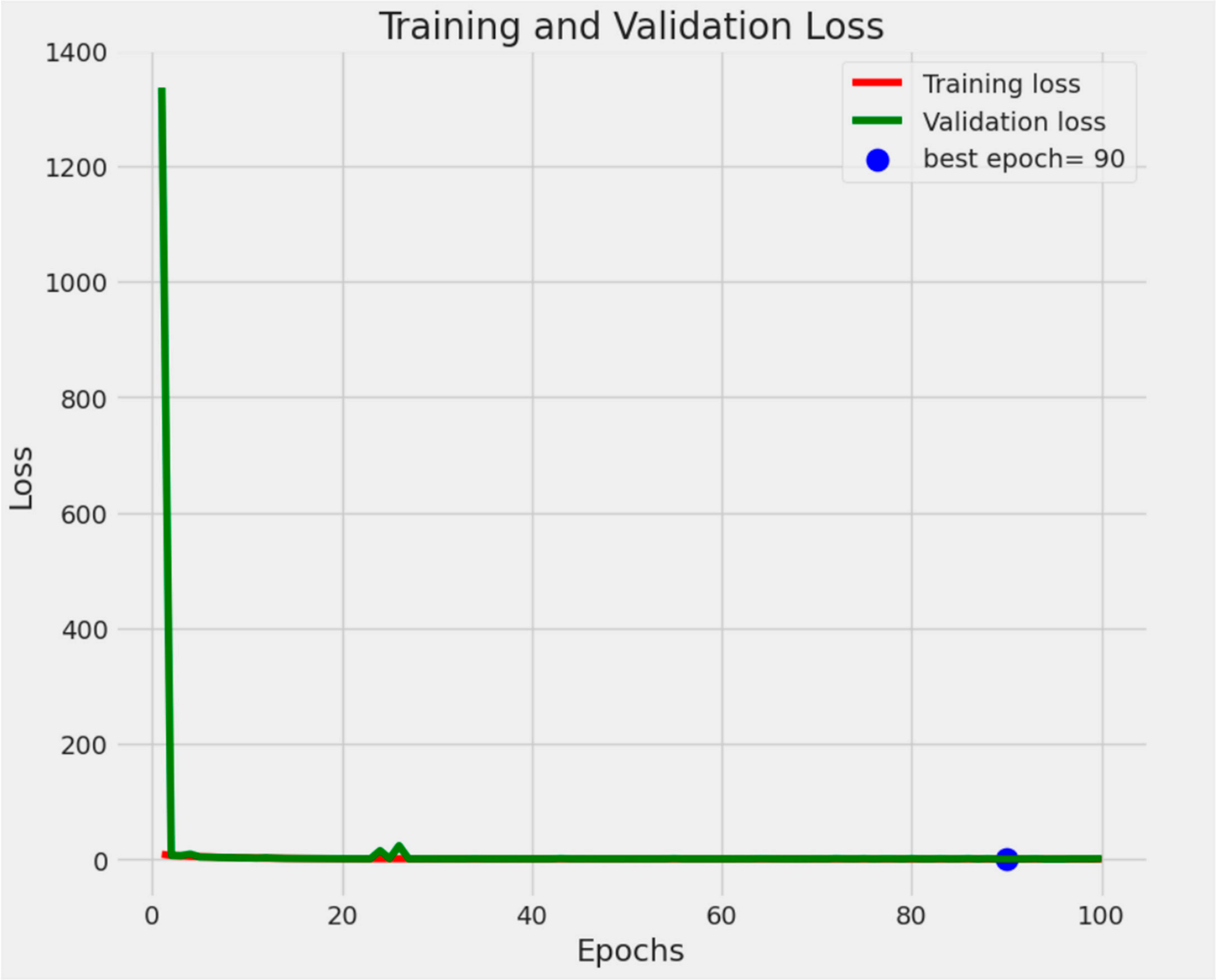
Training and Validation Loss
Training loss
Validation loss
best epoch= 90
Loss
Epochs

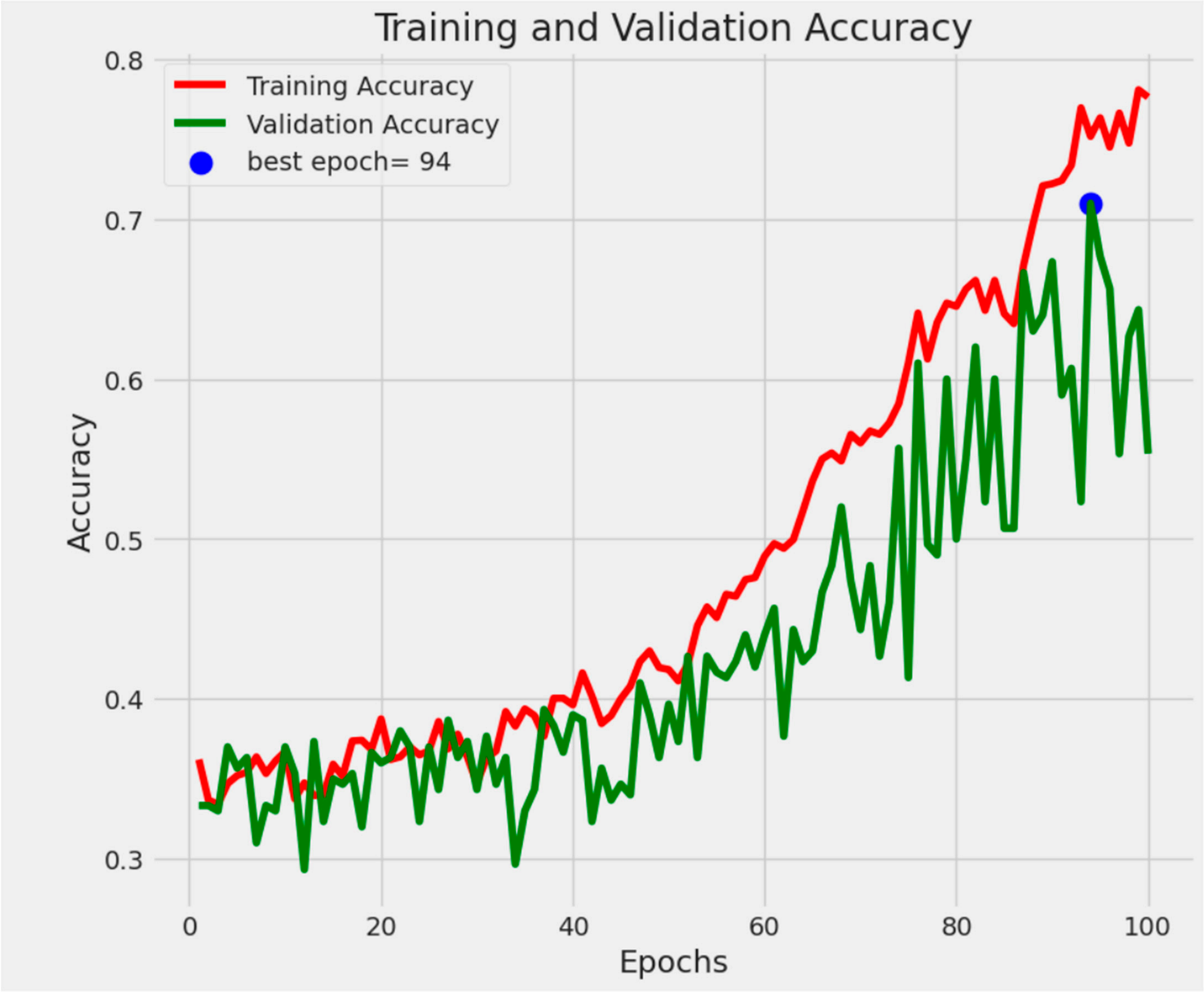

Training and Validation Accuracy
Training Accuracy
Validation Accuracy
best epoch= 94
Accuracy
Epochs
0.3
0.4
0.5
0.6
0.7
0.8
0
20
40
60
80
100

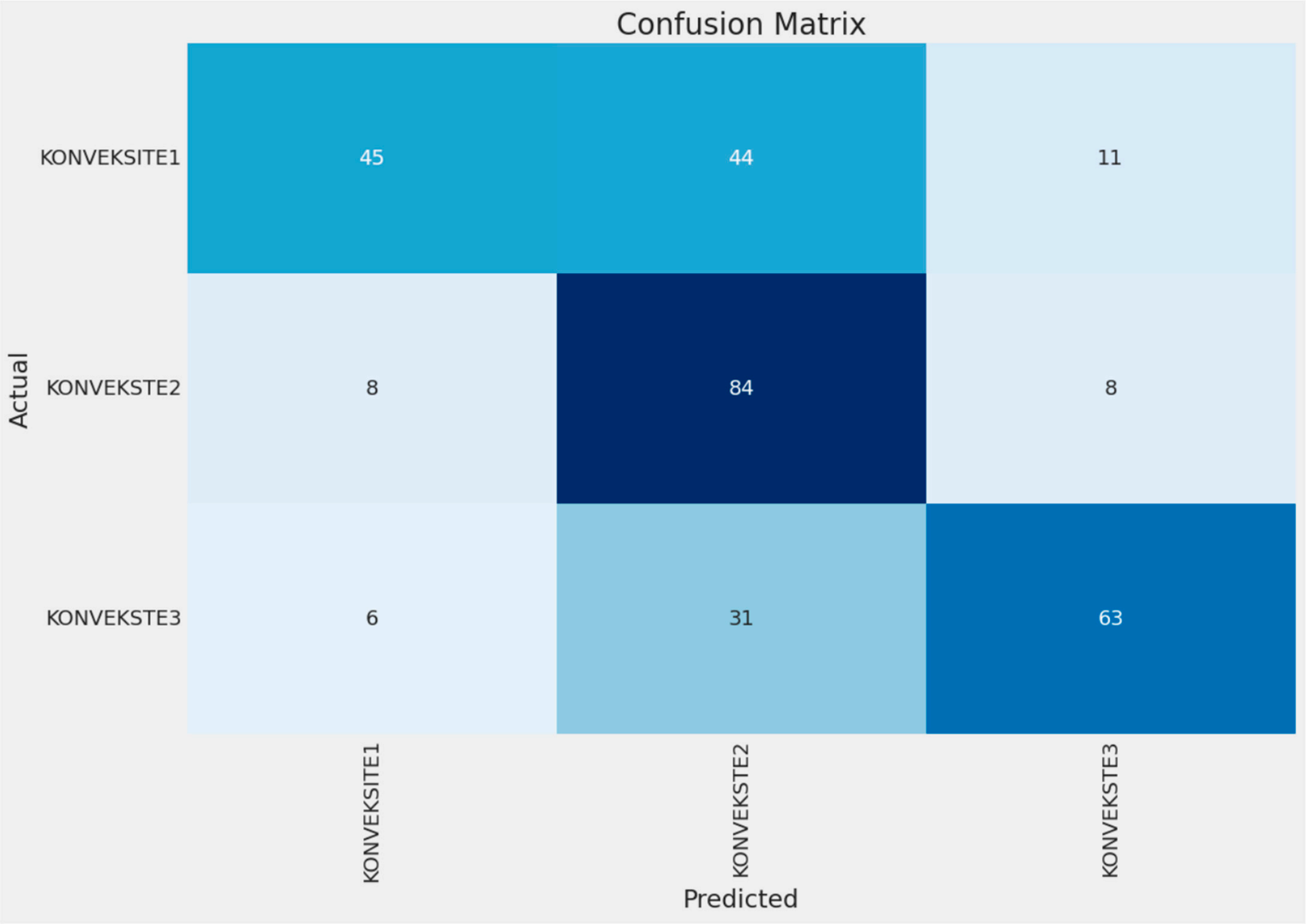

there were 108 errors in 300 tests for an accuracy of 64.00

Classification Report:
----

	precision	recall	f1-score	support
KONVEKSITE1	0.7627	0.4500	0.5660	100
KONVEKSTE2	0.5283	0.8400	0.6486	100
KONVEKSTE3	0.7683	0.6300	0.6923	100
accuracy			0.6400	300
macro avg	0.6864	0.6400	0.6357	300
weighted avg	0.6864	0.6400	0.6357	300

RESNET152

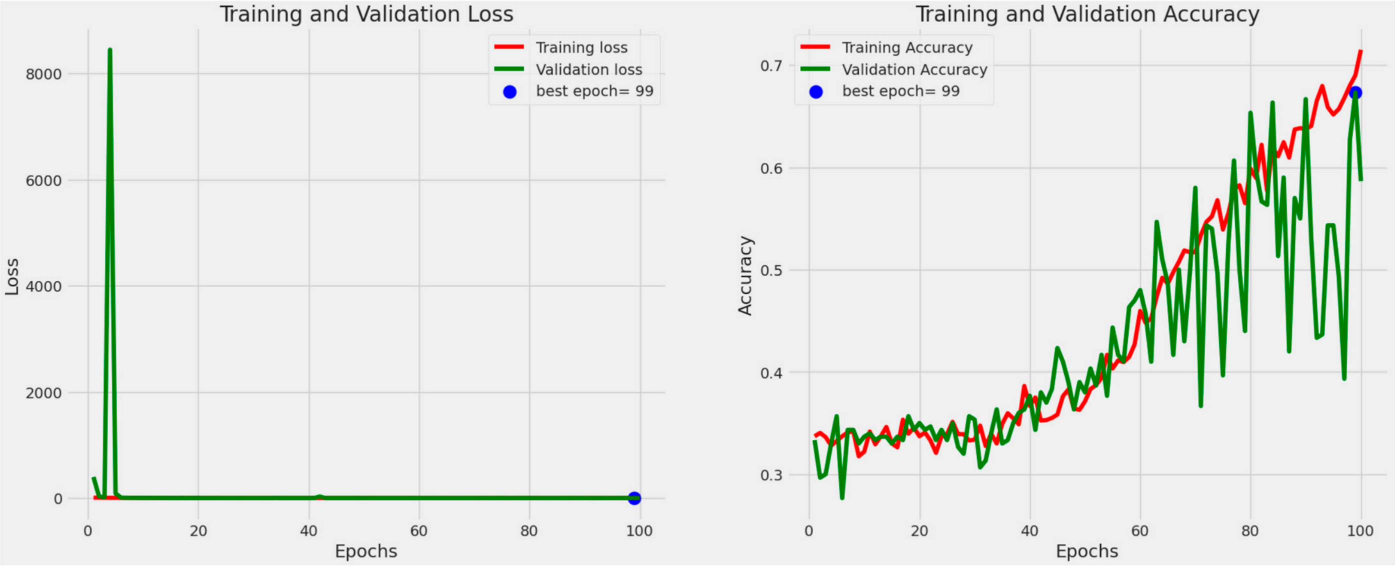
Training and Validation Loss
Training loss
Validation loss
best epoch= 99
Loss
Epochs
0
2000
4000
6000
8000
0
20
40
60
80
100
Training and Validation Accuracy
Training Accuracy
Validation Accuracy
best epoch= 99
Accuracy
Epochs
0.3
0.4
0.5
0.6
0.7
0
20
40
60
80
100

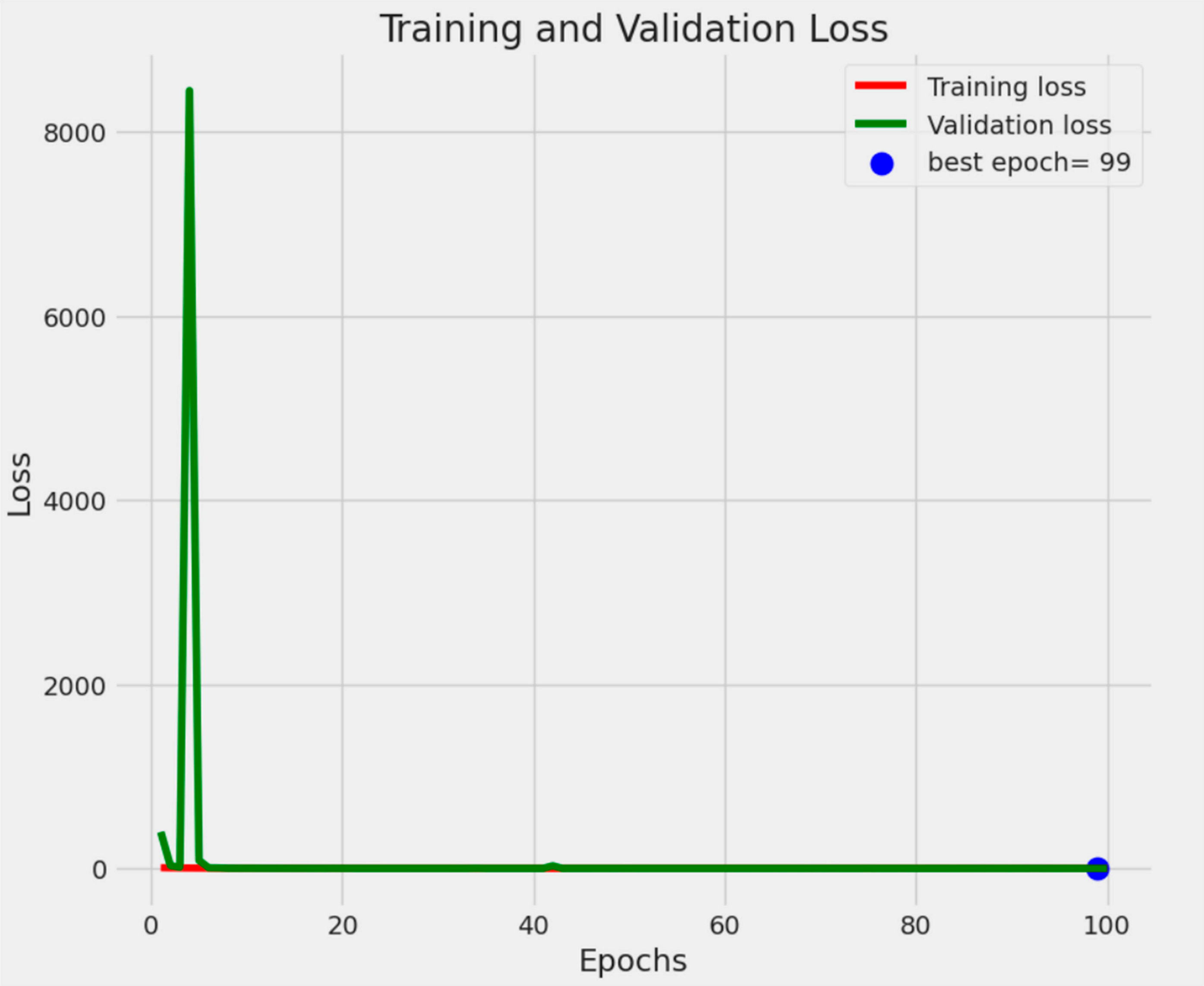
Training and Validation Loss
Training loss
Validation loss
best epoch= 99
Loss
Epochs
0
2000
4000
6000
8000
0
20
40
60
80
100

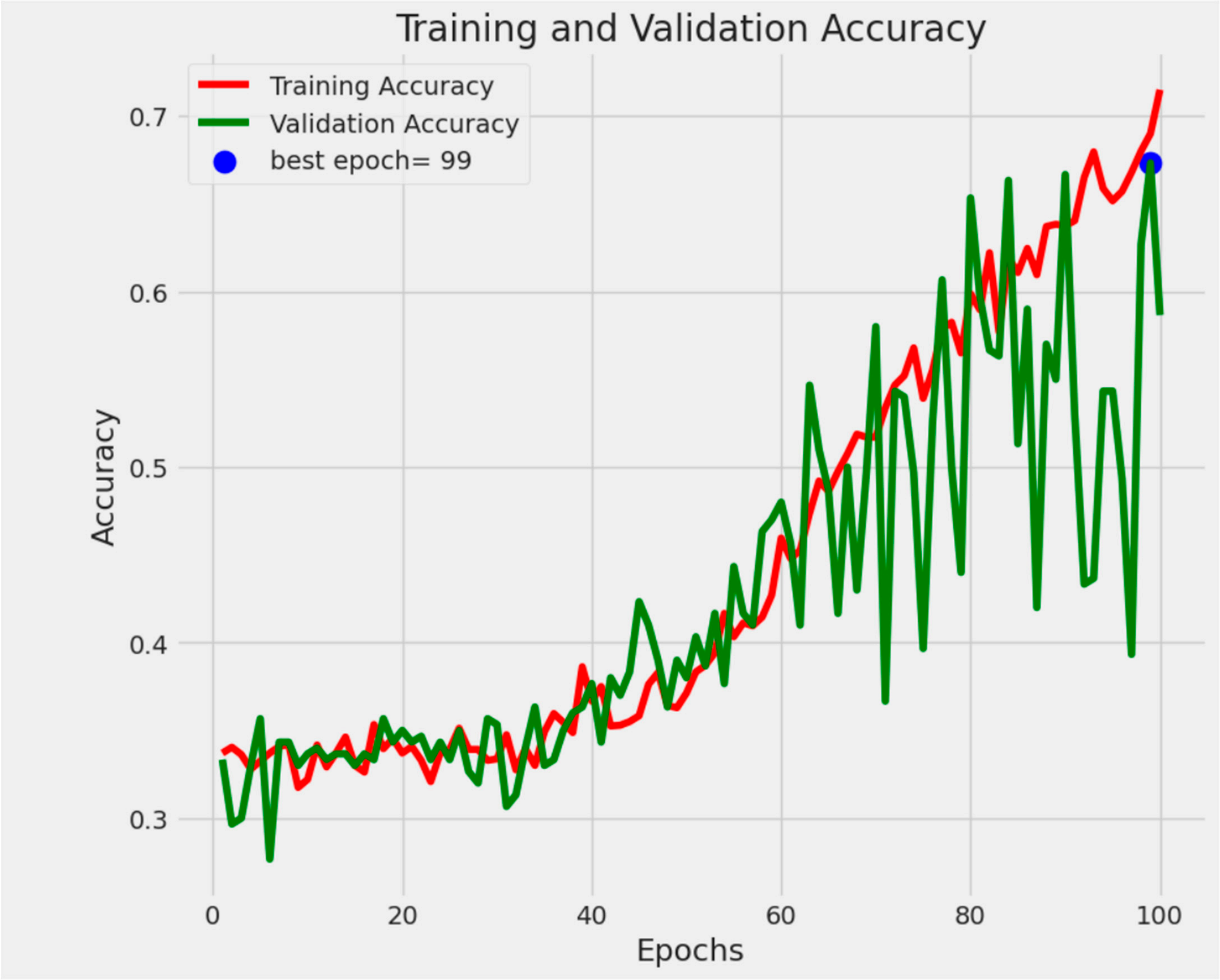
Training and Validation Accuracy
Training Accuracy
Validation Accuracy
best epoch= 99
Accuracy
Epochs
0.7
0.6
0.5
0.4
0.3
0
20
40
60
80
100

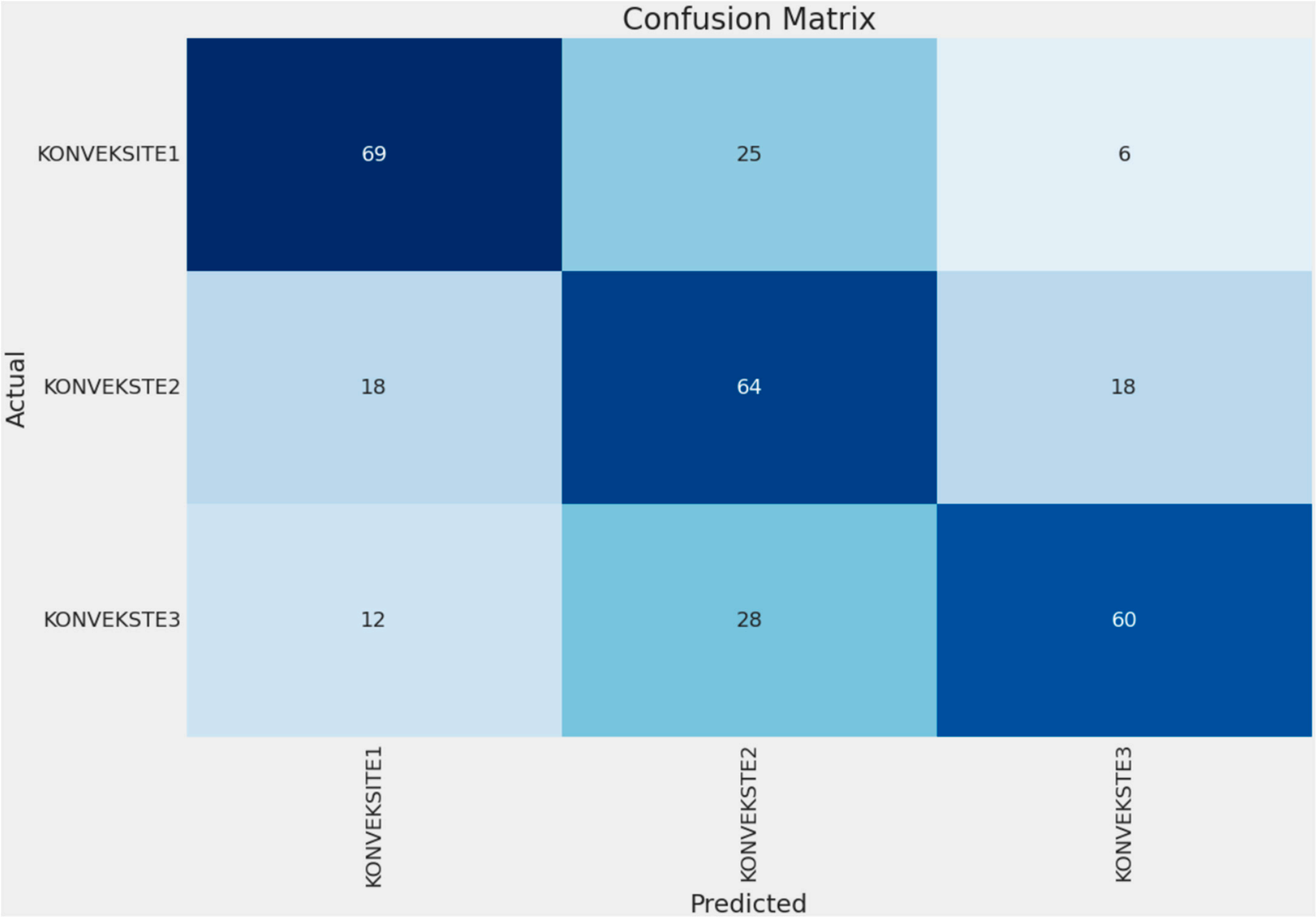

there were 107 errors in 300 tests for an accuracy of 64.33

Classification Report:
----

	precision	recall	f1-score	support
KONVEKSITE1	0.6970	0.6900	0.6935	100
KONVEKSTE2	0.5470	0.6400	0.5899	100
KONVEKSTE3	0.7143	0.6000	0.6522	100
accuracy			0.6433	300
macro avg	0.6528	0.6433	0.6452	300
weighted avg	0.6528	0.6433	0.6452	300

RESNET50

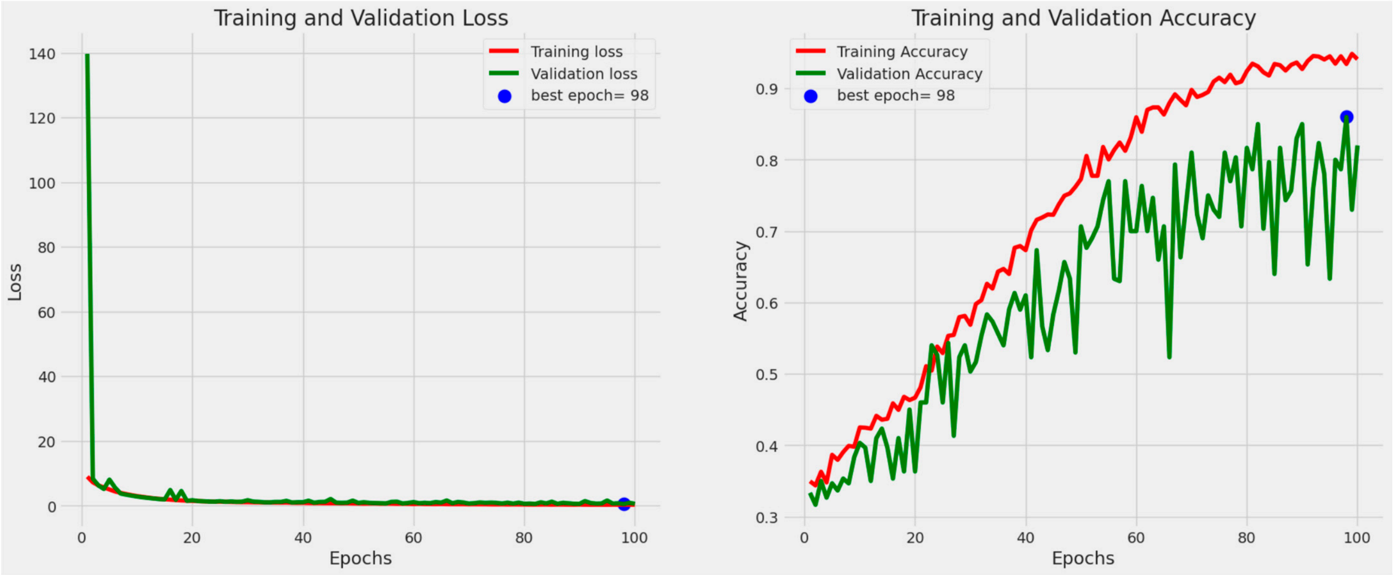

Training and Validation Loss
Training loss
Validation loss
best epoch= 98
Loss
Epochs
Training and Validation Accuracy
Training Accuracy
Validation Accuracy
best epoch= 98
Accuracy
Epochs

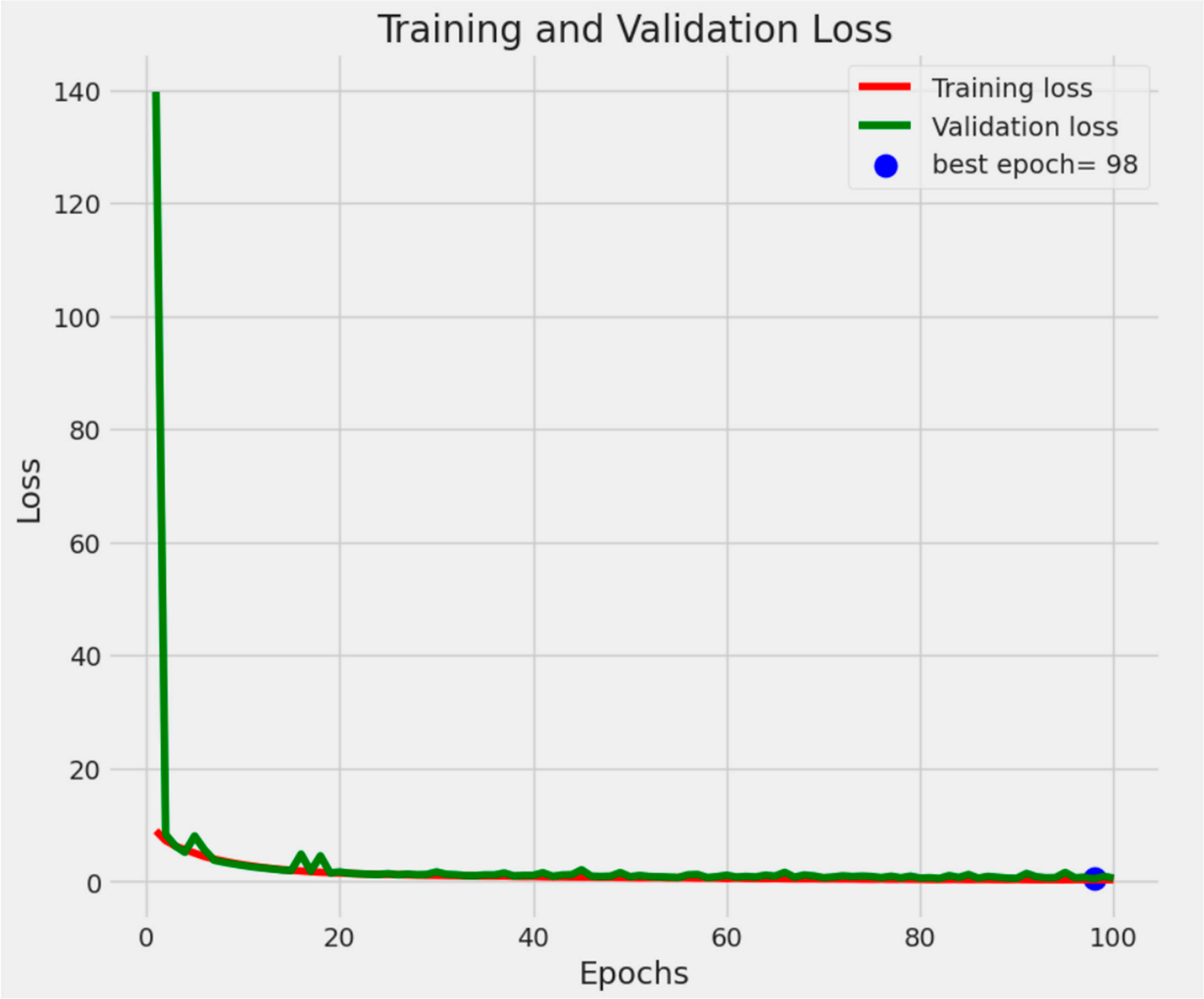

Training and Validation Loss
Training loss
Validation loss
best epoch= 98
Loss
Epochs

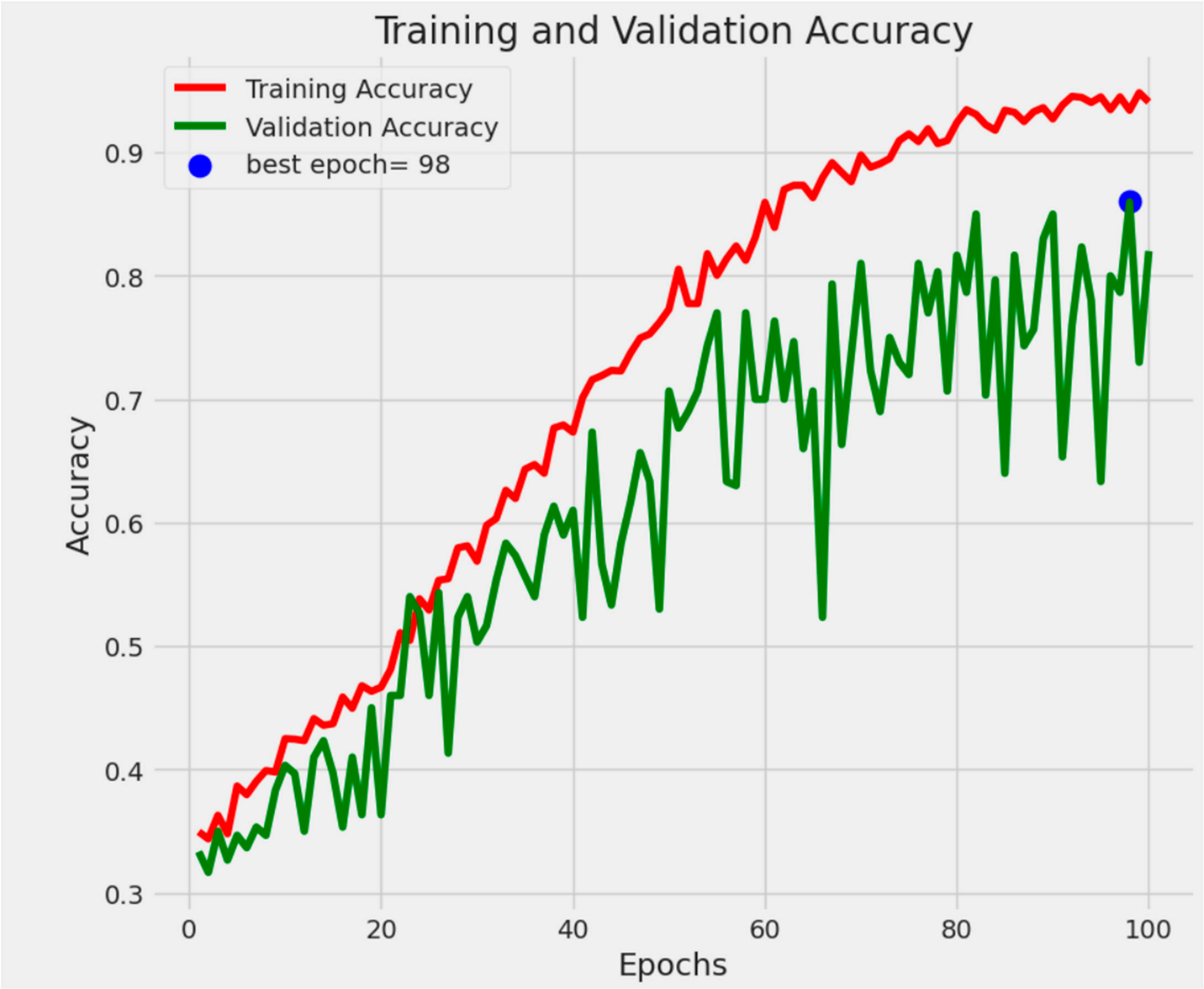
Training and Validation Accuracy
Training Accuracy
Validation Accuracy
best epoch= 98
Accuracy
Epochs
0.3
0.4
0.5
0.6
0.7
0.8
0.9
0
20
40
60
80
100

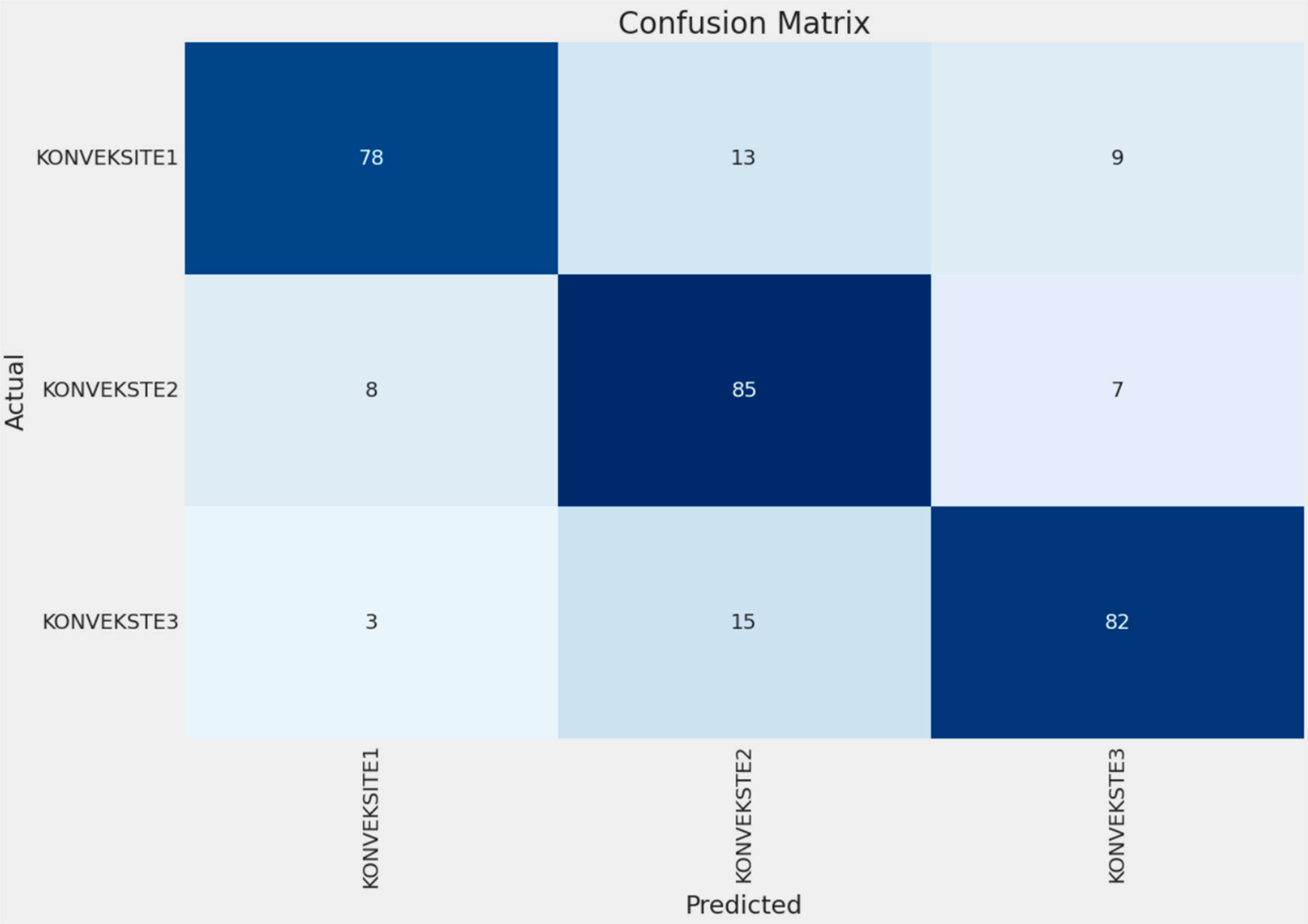

there were 55 errors in 300 tests for an accuracy of 81.67

Classification Report:
----

	precision	recall	f1-score	support
KONVEKSITE1	0.8764	0.7800	0.8254	100
KONVEKSTE2	0.7522	0.8500	0.7981	100
KONVEKSTE3	0.8367	0.8200	0.8283	100
accuracy			0.8167	300
macro avg	0.8218	0.8167	0.8173	300
weighted avg	0.8218	0.8167	0.8173	300

EFFICIENTNET V2

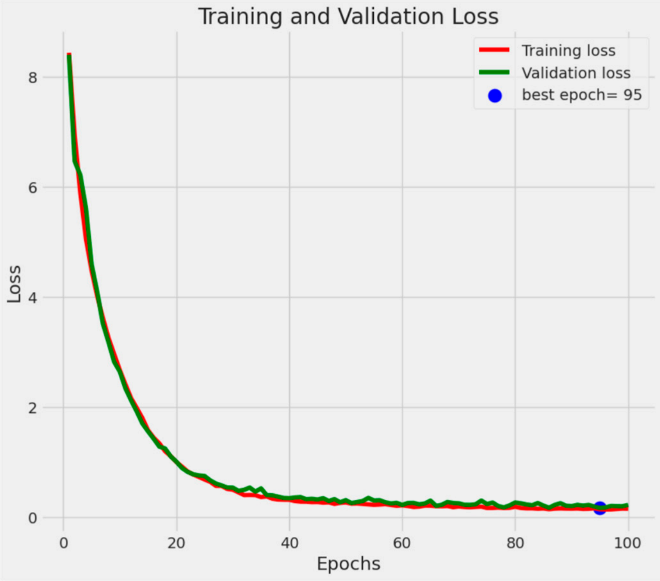

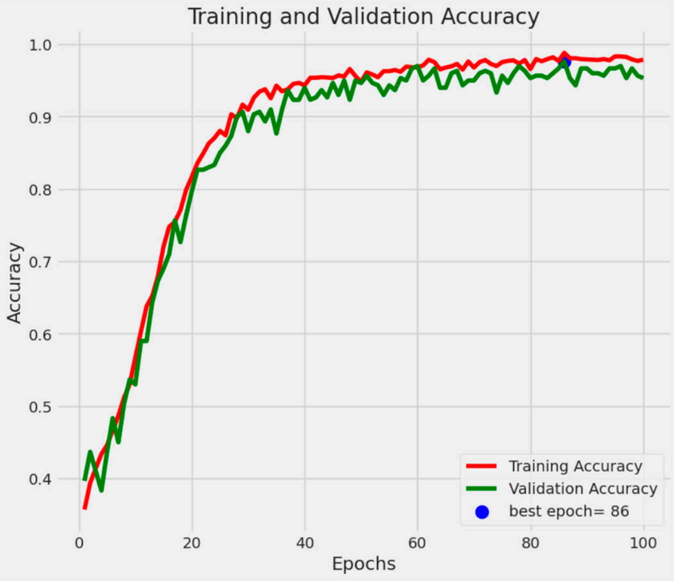

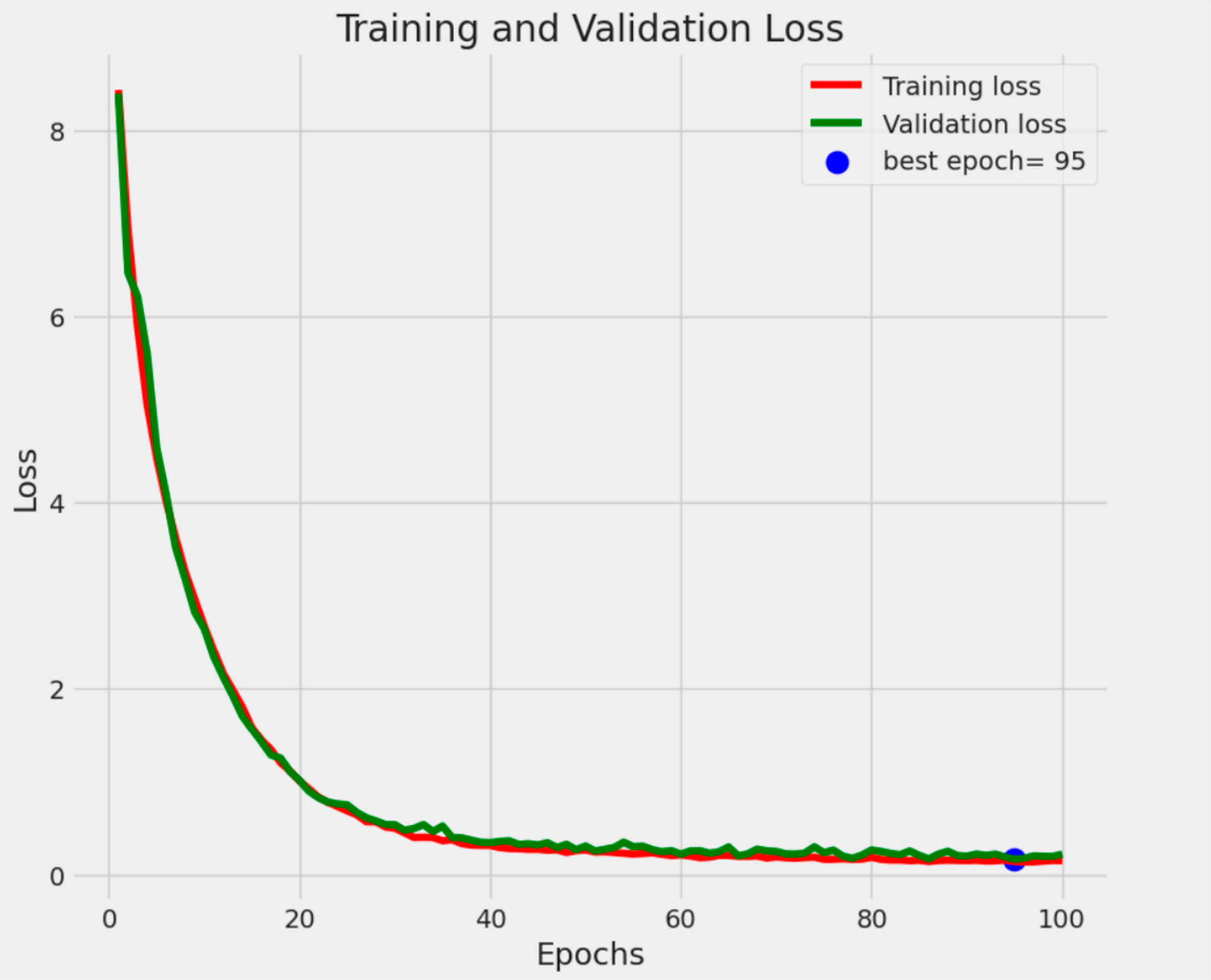

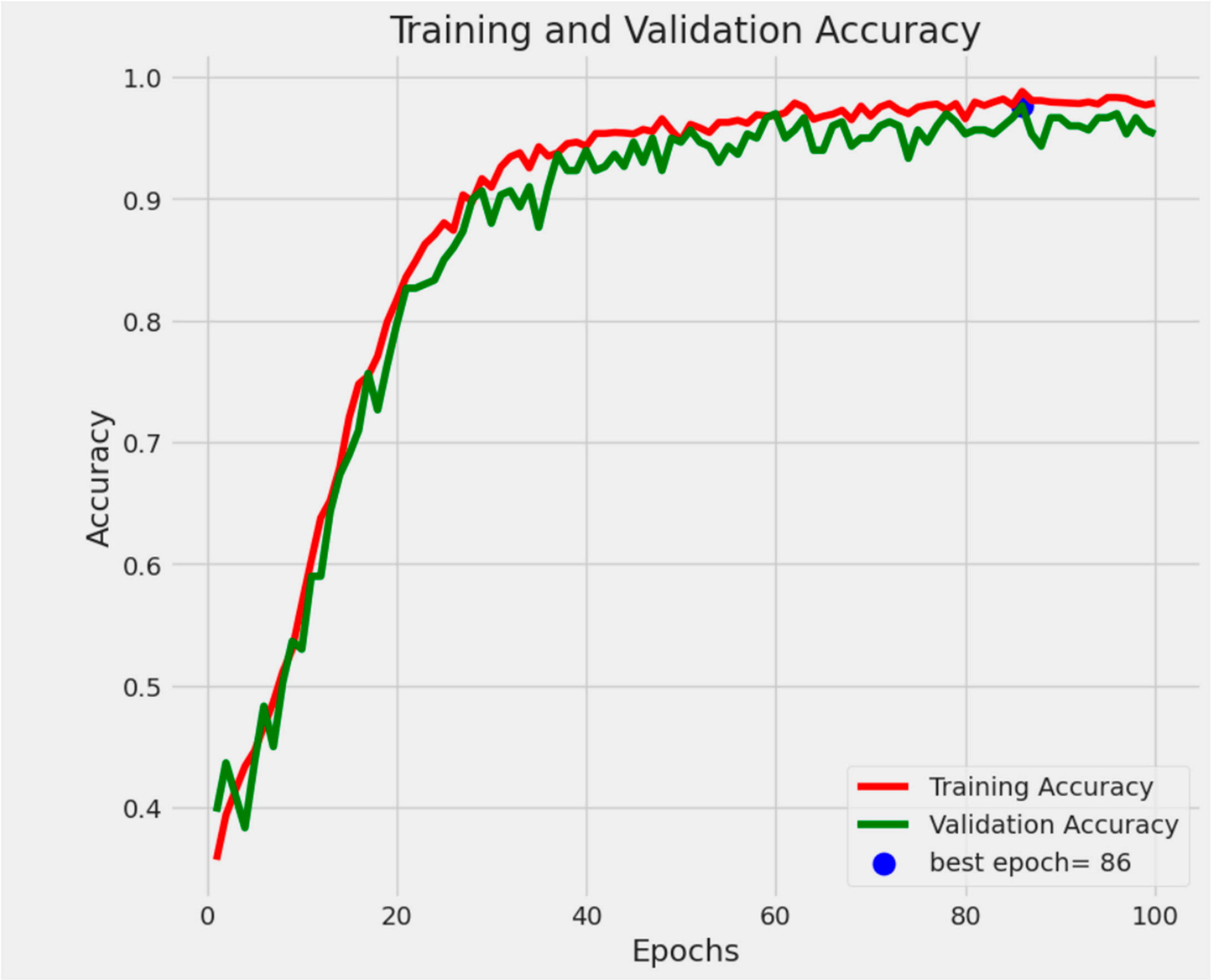
Training and Validation Accuracy
Accuracy
Epochs
1.0
0.9
0.8
0.7
0.6
0.5
0.4
0
20
40
60
80
100
Training Accuracy
Validation Accuracy
best epoch= 86

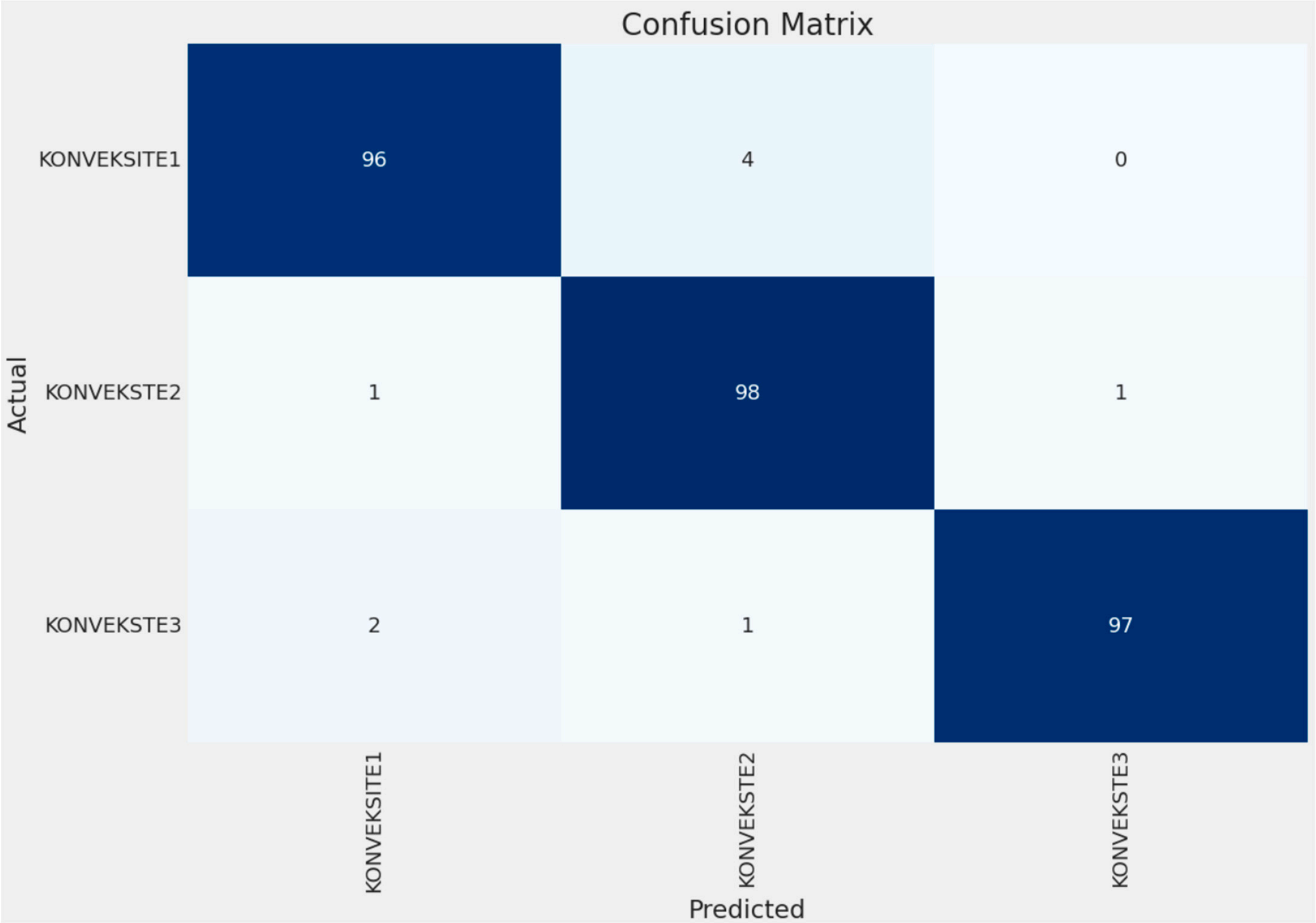

there were 9 errors in 300 tests for an accuracy of 97.00

Classification Report:

----

	precision	recall	f1-score	support
KONVEKSITE1	0.9697	0.9600	0.9648	100
KONVEKSTE2	0.9515	0.9800	0.9655	100
KONVEKSTE3	0.9898	0.9700	0.9798	100
accuracy			0.9700	300
macro avg	0.9703	0.9700	0.9700	300
weighted avg	0.9703	0.9700	0.9700	300
